# Comparison of Cardiovascular and Safety Outcomes of Chlorthalidone vs Hydrochlorothiazide to Treat Hypertension

**DOI:** 10.1001/jamainternmed.2019.7454

**Published:** 2020-02-17

**Authors:** George Hripcsak, Marc A. Suchard, Steven Shea, RuiJun Chen, Seng Chan You, Nicole Pratt, David Madigan, Harlan M. Krumholz, Patrick B. Ryan, Martijn J. Schuemie

**Affiliations:** 1 Department of Biomedical Informatics, Columbia University Medical Center, New York, New York; 2 Medical Informatics Services, NewYork-Presbyterian Hospital, New York; 3 Observational Health Data Sciences and Informatics, New York, New York; 4 Fielding School of Public Health, Department of Biostatistics, University of California, Los Angeles, Los Angeles; 5 David Geffen School of Medicine, Department of Biomathematics, University of California, Los Angeles, Los Angeles; 6 Department of Medicine, Columbia University, New York, New York; 7 Department of Medicine, Weill Cornell Medical College, New York, New York; 8 Department of Biomedical Informatics, Ajou University School of Medicine, Suwon, Korea; 9 Quality Use of Medicines and Pharmacy Research Centre, School of Pharmacy and Medical Sciences, University of South Australia, Adelaide, South Australia, Australia; 10 Department of Statistics, Columbia University, New York, New York; 11 Section of Cardiovascular Medicine, Department of Internal Medicine, Yale University School of Medicine, New Haven, Connecticut; 12 Department of Health Policy and Management, Yale School of Public Health, New Haven, Connecticut; 13 Center for Outcomes Research and Evaluation, Yale-New Haven Hospital, New Haven, Connecticut; 14 Epidemiology Analytics, Janssen Research and Development, Titusville, New Jersey

## Abstract

**Importance:**

Chlorthalidone is currently recommended as the preferred thiazide diuretic to treat hypertension, but no trials have directly compared risks and benefits.

**Objective:**

To compare the effectiveness and safety of chlorthalidone and hydrochlorothiazide as first-line therapies for hypertension in real-world practice.

**Design, Setting, and Participants:**

This is a Large-Scale Evidence Generation and Evaluation in a Network of Databases (LEGEND) observational comparative cohort study with large-scale propensity score stratification and negative-control and synthetic positive-control calibration on databases spanning January 2001 through December 2018. Outpatient and inpatient care episodes of first-time users of antihypertensive monotherapy in the United States based on 2 administrative claims databases and 1 collection of electronic health records were analyzed. Analysis began June 2018.

**Exposures:**

Chlorthalidone and hydrochlorothiazide.

**Main Outcomes and Measures:**

The primary outcomes were acute myocardial infarction, hospitalization for heart failure, ischemic or hemorrhagic stroke, and a composite cardiovascular disease outcome including the first 3 outcomes and sudden cardiac death. Fifty-one safety outcomes were measured.

**Results:**

Of 730 225 individuals (mean [SD] age, 51.5 [13.3] years; 450 100 women [61.6%]), 36 918 were dispensed or prescribed chlorthalidone and had 149 composite outcome events, and 693 337 were dispensed or prescribed hydrochlorothiazide and had 3089 composite outcome events. No significant difference was found in the associated risk of myocardial infarction, hospitalized heart failure, or stroke, with a calibrated hazard ratio for the composite cardiovascular outcome of 1.00 for chlorthalidone compared with hydrochlorothiazide (95% CI, 0.85-1.17). Chlorthalidone was associated with a significantly higher risk of hypokalemia (hazard ratio [HR], 2.72; 95% CI, 2.38-3.12), hyponatremia (HR, 1.31; 95% CI, 1.16-1.47), acute renal failure (HR, 1.37; 95% CI, 1.15-1.63), chronic kidney disease (HR, 1.24; 95% CI, 1.09-1.42), and type 2 diabetes mellitus (HR, 1.21; 95% CI, 1.12-1.30). Chlorthalidone was associated with a significantly lower risk of diagnosed abnormal weight gain (HR, 0.73; 95% CI, 0.61-0.86).

**Conclusions and Relevance:**

This study found that chlorthalidone use was not associated with significant cardiovascular benefits when compared with hydrochlorothiazide, while its use was associated with greater risk of renal and electrolyte abnormalities. These findings do not support current recommendations to prefer chlorthalidone vs hydrochlorothiazide for hypertension treatment in first-time users was found. We used advanced methods, sensitivity analyses, and diagnostics, but given the possibility of residual confounding and the limited length of observation periods, further study is warranted.

## Key Points

**Question:**

What are the relative effectiveness and safety of chlorthalidone and hydrochlorothiazide?

**Findings:**

In this comparative cohort study of 730 255 individuals from multiple large databases, no difference in association with effectiveness between the 2 drugs was found, but a significantly worse safety profile for chlorthalidone was observed.

**Meaning:**

Recommendations to prefer chlorthalidone to hydrochlorothiazide are not supported by real-world evidence.

## Teaser

This cohort study compares cardiovascular and safety outcomes of chlorthalidone and hydrochlorothiazide as first-line therapies for adults with hypertension.

## Introduction

The 2017 American College of Cardiology/American Heart Association hypertension guideline^1^ recommends thiazide and thiazidelike diuretics as one of the first-line treatment classes. Hydrochlorothiazide is the most commonly prescribed member of the class, but the guideline states that chlorthalidone is preferred on the basis of longer half-life and proven trial reduction of cardiovascular disease. However, to our knowledge there are no large, completed randomized clinical trials comparing these medications, although one is in progress.^2^ Indirect network meta-analyses showed superior effectiveness with chlorthalidone,^3,4^ but a large observational study showed approximately equal effectiveness.^5^ Short-term, small randomized clinical trials^6,7^ showed only nominal differences in safety issues such as hypokalemia, but the observational study^5^ showed a worse safety profile for chlorthalidone with higher rates of hypokalemia and hyponatremia.

Both indirect network meta-analyses and traditional observational studies are vulnerable to bias, but recent analytic methods are providing improved strategies to mitigate this risk. These strategies include the balancing of large numbers of baseline patient covariates to make comparison groups more equivalent,^8^ using many negative controls to detect and correct residual bias^9,10^ and testing of the consistency among heterogeneous data sources.^11^ We used these techniques to compare chlorthalidone and hydrochlorothiazide on 55 outcomes in 3 large observational databases of patients from the United States.

## Methods

This multicenter controlled cohort study was part of the Observational Health Data Sciences and Informatics (OHDSI)^12^ Large-Scale Evidence Generation and Evaluation in a Network of Databases for Hypertension initiative.^13^ The research was approved by the Columbia University institutional review board as an OHDSI network study. The use of databases was reviewed by the New England Institution Review Board and was determined to be exempt from broad institutional review board approval because this research project did not involve human subjects research. Informed consent was also waived for this reason.

### Data Sources

We included the 3 OHDSI databases that had at least 2500 patients with exposures to each drug who met the eligibility criteria enumerated below. The MarketScan Commercial Claims and Encounters database (CCAE) (IBM Watson Health; 2001 to 2018) database includes adjudicated health insurance claims and enrollment data from individuals enrolled in US employer-sponsored insurance health plans. The deidentified Clinformatics Data Mart Database (ie, Optum) (OptumInsight; 2001 to 2017) is an adjudicated US administrative health claims database with commercial and Medicare claims from inpatient and outpatient medical services, prescriptions as dispensed, and outpatient laboratory test results processed by participating large national laboratory vendors. The Optum deidentified Electronic Health Record Dataset (ie, PanTher) (Optum; 2007 to 2017) database comprises deidentified electronic health record data including prescriptions as prescribed and administered, laboratory results, vital signs, body measurements, diagnoses, procedures, and information derived from clinical notes using natural language processing. The databases were encoded in OHDSI’s Observational Medical Outcomes Partnership common data model version 5.^12,14,15^ The 3 databases were deidentified.

### Study Design

This study follows a retrospective, observational, comparative cohort design.^16^ We included all patients initiating antihypertensive treatment with chlorthalidone or hydrochlorothiazide, and we defined the index time as the first observed exposure to either drug, including only patients with a prior or concurrent diagnosis of hypertension. We excluded patients having known prior exposure to any hypertension therapies (eAppendix in the Supplement) and those initiating another hypertension treatment within 7 days after starting chlorthalidone or hydrochlorothiazide; however, a patient remained in the cohort if they initiated another hypertension treatment after the 7 days. We required that patients have continuous observation in the database for at least 365 days before treatment initiation. We excluded patients with known prior outcome events and less than 1 day at risk. Full cohort details are provided in the eAppendix in the Supplement.

We used more than 60 000 patient features per database, including demographics (age, sex, index year, index month), all other drugs in the 365 days before the index date, all diagnoses in the 365 days before the index date, and the Charlson Comorbidity Index score,^17^ as baseline covariates for balancing cohorts. Table 1 shows a sample of covariates.

**Table 1.** Selected Baseline Characteristics for CCAE^a^

Characteristic	Before Stratification			After Stratification		
	No. (%)^b^		Standard Difference	No. (%)^b^		Standard Difference
	Chlorthalidone	Hydrochlorothiazide		Chlorthalidone	Hydrochlorothiazide	
Age, mean (SD), y	49.0 (10.4)	48.2 (10.6)	0.05	48.7 (10.4)	48.2 (10.6)	0.03
Age, y						
15-19	60 (0.4)	1700 (0.6)	−0.03	60 (0.4)	1700 (0.6)	−0.03
20-24	230 (1.6)	4600 (1.6)	0	200 (1.4)	4600 (1.6)	−0.02
25-29	420 (3.0)	10 300 (3.6)	−0.03	480 (3.4)	10 100 (3.5)	−0.01
30-34	850 (6.0)	19 000 (6.6)	−0.02	850 (6.0)	19 000 (6.6)	−0.02
35-39	1260 (8.9)	28 500 (9.9)	−0.03	1340 (9.5)	28 500 (9.9)	−0.01
40-44	1850 (13.1)	38 500 (13.4)	−0.01	1860 (13.2)	38 500 (13.4)	0
45-49	2190 (15.5)	47 100 (16.4)	−0.02	2260 (16.0)	47 100 (16.4)	−0.01
50-54	2600 (18.4)	50 600 (17.6)	0.02	2550 (18.1)	50 900 (17.7)	0.01
55-59	2430 (17.2)	46 000 (16.0)	0.03	2400 (17.0)	46 300 (16.1)	0.02
60-64	2050 (14.5)	37 600 (13.1)	0.04	1950 (13.8)	37 600 (13.1)	0.02
65-69	180 (1.3)	3200 (1.1)	0.02	160 (1.1)	3200 (1.1)	0
Female	7310 (51.8)	175 600 (61.1)	−0.19	8590 (60.9)	174 200 (60.6)	0
**Medical History**						
General						
Acute respiratory disease	3300 (23.4)	75 600 (26.3)	−0.07	3640 (25.8)	75 000 (26.1)	−0.01
Attention-deficit/hyperactivity disorder	210 (1.5)	3200 (1.1)	0.04	140 (1.0)	3200 (1.1)	−0.01
Chronic liver disease	160 (1.1)	3200 (1.1)	0	140 (1.0)	3200 (1.1)	−0.01
Chronic obstructive lung disease	170 (1.2)	4000 (1.4)	−0.02	200 (1.4)	4000 (1.4)	0
Dementia	10 (0.1)	300 (0.1)	−0.02	10 (0.1)	300 (0.1)	−0.01
Depressive disorder	1170 (8.3)	23 300 (8.1)	0	1090 (7.7)	23 300 (8.1)	−0.02
Diabetes mellitus	630 (4.5)	13 200 (4.6)	0	660 (4.7)	12 900 (4.5)	0.01
Gastroesophageal reflux disease	1140 (8.1)	21 600 (7.5)	0.02	1020 (7.2)	21 600 (7.5)	−0.01
Gastrointestinal hemorrhage	210 (1.5)	4600 (1.6)	−0.01	230 (1.6)	4600 (1.6)	0
HIV infection	40 (0.3)	900 (0.3)	0.01	30 (0.2)	900 (0.3)	−0.02
Hyperlipidemia	3810 (27.0)	72 700 (25.3)	0.04	3740 (26.5)	72 700 (25.3)	0.03
Lesion of liver	30 (0.2)	600 (0.2)	0	10 (0.1)	600 (0.2)	−0.01
Obesity	1930 (13.7)	28 500 (9.9)	0.12	1380 (9.8)	28 700 (10.0)	−0.01
Osteoarthritis	1590 (11.3)	30 500 (10.6)	0.02	1520 (10.8)	30 500 (10.6)	0
Pneumonia	200 (1.4)	4000 (1.4)	−0.01	230 (1.6)	4000 (1.4)	0.02
Psoriasis	140 (1.0)	2600 (0.9)	0.02	100 (0.7)	2600 (0.9)	−0.02
Renal impairment	140 (1.0)	1400 (0.5)	0.06	80 (0.6)	1400 (0.5)	0.02
Rheumatoid arthritis	130 (0.9)	2300 (0.8)	0.01	160 (1.1)	2300 (0.8)	0.03
Ulcerative colitis	40 (0.3)	600 (0.2)	0.01	30 (0.2)	600 (0.2)	−0.01
Urinary tract infectious disease	750 (5.3)	18 400 (6.4)	−0.04	890 (6.3)	18 100 (6.3)	0
Viral hepatitis C	40 (0.3)	900 (0.3)	0	40 (0.3)	900 (0.3)	−0.01
Visual system disorder	2130 (15.1)	42 800 (14.9)	0	2060 (14.6)	42 500 (14.8)	−0.01
Cardiovascular disease						
Atrial fibrillation	40 (0.3)	600 (0.2)	0.02	30 (0.2)	600 (0.2)	0
Cerebrovascular disease	140 (1.0)	2900 (1.0)	0	110 (0.8)	2300 (0.8)	0.01
Coronary arteriosclerosis	160 (1.1)	2900 (1.0)	0.02	160 (1.1)	2600 (0.9)	0.02
Heart disease	1020 (7.2)	18 400 (6.4)	0.03	900 (6.4)	17 500 (6.1)	0.01
Heart failure	60 (0.4)	900 (0.3)	0.01	30 (0.2)	600 (0.2)	−0.01
Ischemic heart disease	110 (0.8)	2600 (0.9)	−0.01	110 (0.8)	2300 (0.8)	0
Peripheral vascular disease	550 (3.9)	9200 (3.2)	0.04	440 (3.1)	8600 (3.0)	0
Pulmonary embolism	30 (0.2)	600 (0.2)	0	10 (0.1)	600 (0.2)	−0.01
Neoplasms						
Hematologic neoplasm	70 (0.5)	1100 (0.4)	0.01	60 (0.4)	1100 (0.4)	−0.01
Malignant lymphoma	30 (0.2)	600 (0.2)	0.02	40 (0.3)	600 (0.2)	0.02
Malignant neoplastic disease	550 (3.9)	10 900 (3.8)	0.01	560 (4.0)	10 900 (3.8)	0.01
Malignant tumor of breast	130 (0.9)	2900 (1.0)	−0.01	170 (1.2)	2900 (1.0)	0.02
Malignant tumor of colon	10 (0.1)	600 (0.2)	−0.01	10 (0.1)	600 (0.2)	−0.02
Malignant tumor of urinary bladder	10 (0.1)	300 (0.1)	0	10 (0.1)	300 (0.1)	0.01
Primary malignant neoplasm of prostate	60 (0.4)	900 (0.3)	0	60 (0.4)	900 (0.3)	0
Medication use						
Antibacterials for systemic use	6500 (46.1)	146 300 (50.9)	−0.10	7180 (50.9)	145 400 (50.6)	0
Antidepressants	2500 (17.7)	55 200 (19.2)	−0.04	2650 (18.8)	54 900 (19.1)	−0.01
Antiepileptics	860 (6.1)	17 200 (6.0)	0	860 (6.1)	17 000 (5.9)	0.01
Anti-inflammatory and antirheumatic products	3470 (24.6)	75 900 (26.4)	−0.04	3720 (26.4)	75 300 (26.2)	0
Antineoplastic agents	210 (1.5)	4300 (1.5)	0.01	240 (1.7)	4300 (1.5)	0.02
Antipsoriatics	60 (0.4)	1100 (0.4)	0	60 (0.4)	1100 (0.4)	0
Antithrombotic agents	380 (2.7)	6300 (2.2)	0.03	340 (2.4)	5700 (2.0)	0.02
Drugs for acid-related disorders	1890 (13.4)	40 200 (14.0)	−0.02	1960 (13.9)	39 900 (13.9)	0
Drugs for obstructive airway diseases	2960 (21.0)	58 300 (20.3)	0.02	2910 (20.6)	58 300 (20.3)	0.01
Drugs used in diabetes	410 (2.9)	8900 (3.1)	−0.01	450 (3.2)	8600 (3.0)	0.01
Immunosuppressants	240 (1.7)	4300 (1.5)	0.02	200 (1.4)	4300 (1.5)	−0.01
Lipid modifying agents	2000 (14.2)	38 800 (13.5)	0.02	1990 (14.1)	38 500 (13.4)	0.02
Opioids	2200 (15.6)	46 000 (16.0)	−0.01	2260 (16.0)	45 400 (15.8)	0
Psycholeptics	2580 (18.3)	52 300 (18.2)	0	2620 (18.6)	52 000 (18.1)	0.01

^a^ Values are rounded, with prestratification counts estimated from percentages and totals and poststratification counts showing estimated effective numbers.

^b^ The Commercial Claims and Encounters Database (CCAE) chlorthalidone group had 14 104 patients, and the hydrochlorothiazide group had 287 390.

The primary outcomes, which we prespecified, were hospitalization for acute myocardial infarction, heart failure, ischemic or hemorrhagic stroke, and a composite cardiovascular disease outcome including the first 3 outcomes and sudden cardiac death (eAppendix in the Supplement). International Society for Pharmacoeconomics and Outcomes Research (ISPOR) reporting guideline was followed.

The 51 safety outcomes, which we prespecified, included electrolyte disorders, such as hypokalemia and hyponatremia, acute and chronic kidney disease, and gout. They were assembled from safety concerns reported on hypertension drug product labels, and they are defined in the eAppendix in the Supplement. We began the outcome risk window 1 day after treatment initiation and used 2 design choices to define the window end. First, we ended the outcome time-at-risk window at first cessation of continuous drug exposure, analogous to an on-treatment design. Second, we ended the outcome time-at-risk window when the patient was no longer in the database or the outcome occurred, analogous to an intention-to-treat design. Continuous drug exposures were constructed from the available longitudinal data by considering sequential dispensing or prescriptions with gaps less than 30 days. We show on-treatment results in this article and intention-to-treat results in the eAppendix in the Supplement.

### Statistical Analysis

Analysis began June 2018. We conducted our cohort study using the open-source OHDSI CohortMethod R package,^18^ with large-scale analytics from the Cyclops R package.^19^ We used propensity scores^20^ to balance the chlorthalidone and hydrochlorothiazide cohorts with respect to measured confounding variables, with a separate model developed for each database. Propensity scores estimated the treatment exposure probability conditional on 60 535 to 70 072 pretreatment baseline covariates in the 1 year prior to treatment initiation. We performed propensity score stratification and then estimated comparative chlorthalidone-vs-hydrochlorothiazide hazard ratios (HRs) using a Cox proportional hazards model, accounting for time on therapy and censoring; we used Kaplan-Meier survival plots to assess the assumption of proportionality. Detailed covariate and methods information is provided in the eAppendix in the Supplement. We used propensity score and covariate balance metrics to assess the success of measured confounding control, defined as all covariates having a standardized difference of the mean less than 0.1. We used preference score distributions to judge equipoise, defined as having most patients within 0.25 to 0.75 propensity score–based preference scores.^21^ The HRs and associated SEs from each of the 3 database analyses were then combined through a random-effect meta-analysis to produce a composite effect estimate.

We estimated residual bias using 76 negative control outcomes^11^ (eAppendix in the Supplement) (ie, outcomes believed to be caused by neither chlorthalidone nor hydrochlorothiazide, which therefore have an assumed HR of 1) identified through a data-rich algorithm,^22^ and we augmented the set by injecting events into the negative controls to create synthetic positive controls^9^ (ie, outcomes where the true HR is assumed known and greater than 1). We measured how often the true relative risks for controls were inside of their CIs (it should be 95% of the time for 95% CIs), and we calibrated all HR estimates, their 95% CIs, and their 2-sided *P* values so that approximate 95% coverage was achieved for the controls.

To address multiplicity concerns, we indicate which estimates remain statistically significant after a Bonferroni correction for 55 hypotheses. However, we report all differences.

### Time at Risk, Baseline Blood Pressure, Dose, and Potassium

Outcomes may differ in timing: electrolyte imbalances may occur quickly while cardiovascular outcomes may take longer to occur, issues that were identified after the prespecified analyses. We therefore performed a post hoc analysis with risk period starting 91 days after the first drug exposure. This both ensured that all recorded days at risk had at least a 91-day exposure to the drug and shifted the median time at risk to longer time than for the primary analysis.

Only the electronic health record database, PanTher, had systolic and diastolic blood pressure readings recorded to assess baseline blood pressure. We repeated the analysis on that database with last systolic and last diastolic blood pressures in the year prior to index treatment included in the propensity score model using cubic splines.

PanTher also had appropriately timed blood potassium levels for some patients. We compared the last potassium level up to a year before first drug dose with the last potassium level 30 to 90 days after the first dose. In the largest database, CCAE, we addressed differences in dosing and potency by restricting the analysis to patients whose dose was 12.5 mg of chlorthalidone or 25 mg of hydrochlorothiazide for the entire on-treatment period.

## Results

### Balance Between Cohorts

**Figure 1.**
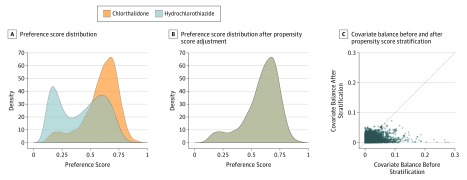
Comparability of the Populations for Commercial Claims and Encounters Database (CCAE) A, The preference score is a transformation of the propensity score that adjusts for differences in the sizes of the 2 treatment groups. A higher overlap indicates individuals in the 2 groups were more similar in terms of their predicted probability of receiving 1 treatment over the other. This plot shows sufficient equipoise (majority of both distributions being between 0.25 and 0.75) in CCAE that propensity score stratification should be able to create balance without discounting a large proportion of the population, but it shows sufficient difference (nonoverlap) that propensity score stratification is necessary. B, Same plot as panel A but showing essentially perfect overlap after adjustment (matching shown here). This illustrates the success of the adjustment in achieving balance. C, Each dot represents the standardized difference of the means for a single covariate before and after stratification on the propensity score. The panel shows poor balance before but excellent balance after stratification, with all 63 069 under 0.1 and most under 0.05. All measured variables were successfully balanced by the adjustment, and the 2 cohorts were in fact similar on all measured aspects.

For CCAE, there were 14 104 individuals receiving chlorthalidone and 287 390 individuals receiving hydrochlorothiazide; for Optum there were 7696 and 189 834, respectively; and for PanTher there were 15 118 and 216 113, respectively. Table 1 summarizes a selection of baseline characteristics before and after propensity score stratification for CCAE (eAppendix, eTables 3-8 in the Supplement), with substantial prestratification differences in sex, obesity, and several other variables but with all of the covariates having small (less than 0.1) standardized differences of the mean after propensity score stratification. Figure 1A and B shows the preference score distribution^21^ for CCAE, demonstrating sufficient between-group equipoise. Figure 1C shows the standardized differences of the means of all covariates before and after propensity score stratification. Before stratification, variables differed by up to almost 0.3, but after stratification all differed by substantially less than 0.1 and most less than 0.05, indicating excellent balance on all variables (eAppendix in the Supplement, section 2.4, reports the other databases, which had similar results; eFigure 4 in the Supplement).

### Effectiveness

**Figure 2.**
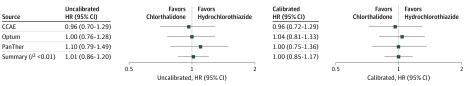
Homogeneity on Effectiveness Hazard ratios (HRs) and forest plot of the 3 databases and the meta-analysis for chlorthalidone vs hydrochlorothiazide on the composite cardiovascular disease outcome. The 3 databases showed excellent agreement. CCAE indicates Commercial Claims and Encounters Database.

We found no statistically significant differences in risk of acute myocardial infarction, hospitalized heart failure, stroke, or the composite cardiovascular outcome between individuals receiving chlorthalidone and hydrochlorothiazide (Table 2; eTables 9-12 in the Supplement). The calibrated and uncalibrated HRs were very close, and this similarity indicated that the 76 negative controls and the synthetic positive controls revealed little evidence of residual confounding (in the form of false-positive or skewed results in the controls). The HR for the composite cardiovascular outcome for patients receiving chlorthalidone compared with patients receiving hydrochlorothiazide was 1.00 (95% CI, 0.85-1.17). Figure 2 shows the estimate to be consistent across databases (eFigure 5 in the Supplement).

**Table 2.** Effectiveness by Outcome (Propensity Score Stratification, On-Treatment)

Outcome	Chlorthalidone, Total No.		Hydrochlorothiazide, No. (%)		Hazard Ratio (95% CI)^a^	
	Events	Patients^b^	Events	Patients^b^	Uncalibrated	Calibrated
Acute myocardial infarction	41	36 859	952	692 371	0.93 (0.63-1.36)	0.92 (0.64-1.31)
Hospitalization for heart failure	62	36 833	1248	691 409	1.07 (0.82-1.39)	1.05 (0.82-1.34)
Stroke	60	36 755	1141	689 698	1.13 (0.86-1.47)	1.10 (0.86-1.41)
Composite cardiovascular disease^c^	149	36 628	3089	687 106	1.01 (0.86-1.20)	1.00 (0.85-1.17)

^a^ Hazard ratio for chlorthalidone vs hydrochlorothiazide (lower hazard ratio favors chlorthalidone).

^b^ Number of patients exposed varies by outcome owing to differences in whether database has hospitalization information and outcome-specific preexposure exclusions.

^c^ Composite cardiovascular disease includes the first 3 outcomes and sudden cardiac death.

### Safety

**Figure 3.**
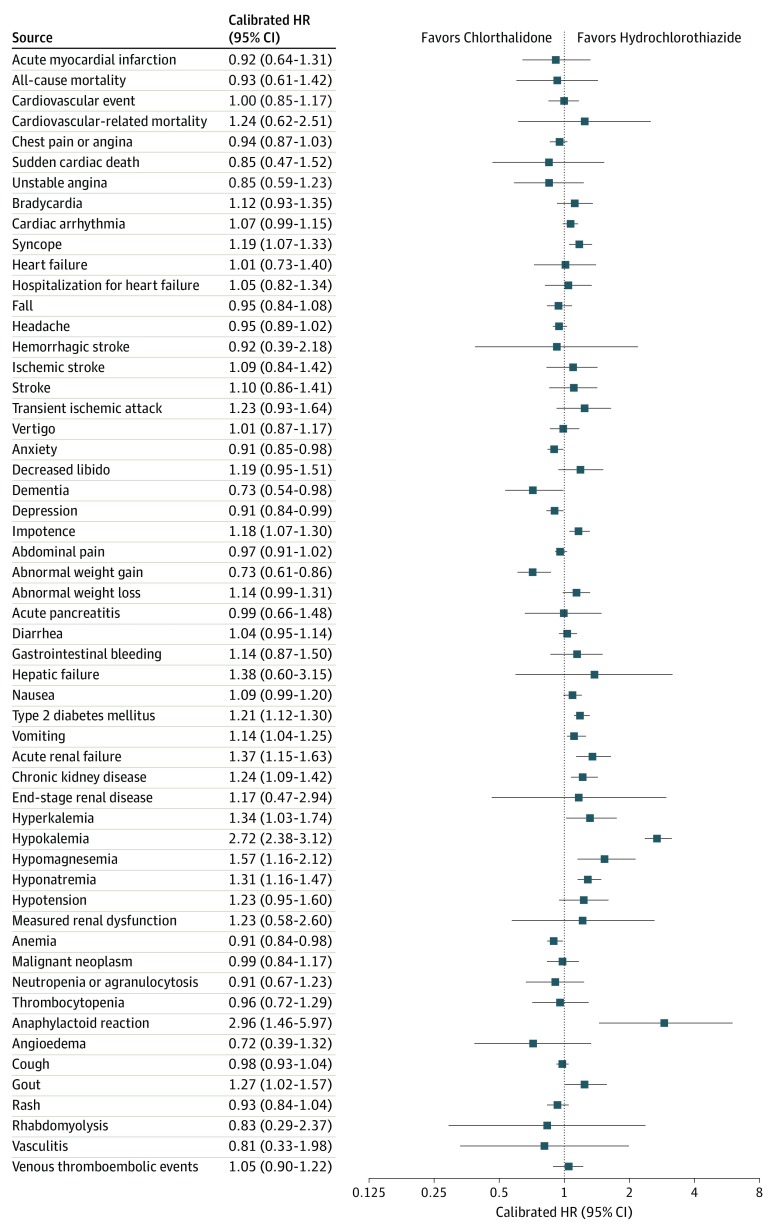
Forest Plot of Safety and Effectiveness Outcomes Forest plot of hazard ratio estimates and calibrated 95% CIs for chlorthalidone vs hydrochlorothiazide for 55 safety and effectiveness outcomes. The safety signals predominantly favored hydrochlorothiazide.

Figure 3 shows the comparative safety profile for the 2 drugs (eAppendix, eFigure 6, and eTables 9-12 in the Supplement). Chlorthalidone shows a different safety profile compared with hydrochlorothiazide, with the following outcomes different after correction for multiple hypotheses: chlorthalidone was associated with an increased risk for hypokalemia, hyponatremia, acute renal failure, chronic kidney disease, and type 2 diabetes mellitus. Chlorthalidone was associated with a decreased risk for diagnosed abnormal weight gain. Hypokalemia had an uncalibrated HR of 2.99 (95% CI, 2.58-3.46) and calibrated HR of 2.72 (95% CI, 2.38-3.12) (eFigure 5 in the Supplement). The uncalibrated and calibrated HR for hyponatremia was 1.36 (95% CI, 1.20-1.53) and 1.31 (95% CI, 1.16-1.47), respectively; acute renal failure, 1.42 (95% CI, 1.18-1.72) and 1.37 (95% CI, 1.15-1.63), respectively; and abnormal weight gain, 0.72 (95% CI, 0.60-0.87) and 0.73 (95% CI, 0.61-0.86), respectively. The following findings had CIs that excluded 1 but did not surpass the Bonferroni threshold: chlorthalidone was associated with an increased risk of hypomagnesemia, hyperkalemia, vomiting, syncope, gout, impotence, and anaphylactoid reaction and associated with a decreased risk of anemia, depression, dementia, and anxiety.

Furthermore, the event rates for hypokalemia were substantial. In CCAE, the rates per patient were 6.3% and 1.9% for chlorthalidone and hydrochlorothiazide, respectively. The Kaplan-Meier curves (eAppendix and eFigure 7 in the Supplement) were consistent with our assumption of proportionality for our use of the Cox proportional hazards model.

### Sensitivity to Time at Risk

When we shifted the time at risk to begin 91 days after first drug exposure in the largest database, CCAE, the median end of the at-risk period shifted from 92 days to 267 days after the first drug exposure, and the upper quartile shifted from 425 days to 689 days. The HR estimates for the composite cardiovascular outcome was 0.94 (95% CI, 0.60-1.38) for the delayed risk period, which was similar to that for the original period, which was 0.96 (95% CI, 0.70-1.29).

### Sensitivity to Baseline Blood Pressure

We assessed balance on baseline blood pressure in the PanTher database. Before any stratification, the standardized difference of the mean for blood pressure was 0.200 for systolic and 0.168 for diastolic. After propensity score stratification, but without including blood pressure in the model, the differences were 0.126 and 0.094, respectively. Therefore, without knowing blood pressure, balancing on the other 60 535 PanTher covariates resulted in marked improvement in balance on blood pressure. When we included blood pressure in the propensity model, the differences improved to 0.046 and less than 0.001 for systolic and diastolic blood pressure, respectively, with good balance among all other covariates (eFigure 1 in the Supplement). Furthermore, there were no major shifts in any of the effectiveness or safety outcomes (eFigure 2 in the Supplement) between propensity models with and without blood pressure, pointing to low sensitivity to slight imbalance in baseline blood pressure.

### Sensitivity to Dose

The subgroup receiving 12.5 mg of chlorthalidone vs 25 mg of hydrochlorothiazide had an uncalibrated HR for hypokalemia of 1.71 (95% CI, 1.37-2.11) and calibrated HR of 1.57 (95% CI, 1.25-2.01), passing the Bonferroni threshold (eTables 1-2 in the Supplement). No other outcomes passed the threshold.

### Change in Measured Potassium

PanTher showed greater reduction in blood potassium level in the chlorthalidone group than in the hydrochlorothiazide group (chlorthalidone, 0.22 mEq/L; hydrochlorothiazide, 0.12 mEq/L; *P* = .03; 95% CI, 0.01-0.18). Visualization of the changes reveals a greater downward shift in chlorthalidone (eFigure 3 in the Supplement).

## Discussion

Our observational analysis across 3 large and disparate databases showed no significant difference in the effectiveness of chlorthalidone compared with hydrochlorothiazide for a range of cardiovascular outcomes, but chlorthalidone had a worse safety profile, including an association with an increased risk of hypokalemia with an HR of 2.72 (95% CI, 2.38-3.12). Other electrolyte abnormalities were also more frequent.

Our study is the largest multisite analysis of real-world evidence to address this comparison, with 36 918 records of individuals prescribed chlorthalidone and 693 337 prescribed hydrochlorothiazide across the 3 databases. We found consistent results across our 3 databases, excellent balance on more than 60 000 covariates after stratification, little bias based on our controls, little sensitivity to changes in time at risk, to inclusion of baseline blood pressure or to initial dose, and confirmation of differences in potassium by laboratory measurement.

Chlorthalidone use was associated with a higher rate of electrolyte and renal disorders, with an increase in hypokalemia, hyponatremia, acute renal failure, and chronic kidney disease. Based on the electrolyte findings, chlorthalidone’s association with an increase in rate of type II diabetes may be associated with potassium depletion or to dehydration. Chlorthalidone’s lower rate of abnormal weight gain may be associated with more effective diuresis.

To our knowledge, there have been no completed large head-to-head randomized clinical trials comparing chlorthalidone and hydrochlorothiazide on cardiovascular effectiveness. An indirect meta-analysis by Thomopoulos et al^23^ looked at cardiovascular outcomes vs placebo for low-dose diuretics; there were nominal differences, but the 2018 European Society of Cardiology/European Society of Hypertension guideline^24^ interpreted the results as roughly equivalent for the 2 drugs. The indirect meta-analysis by Roush et al^4^ showed an improved relative risk for composite cardiovascular events for chlorthalidone compared with hydrochlorothiazide of 0.79 (95% CI, 0.72-0.88). The real-world evidence study by Dhalla et al^5^ estimated an HR for chlorthalidone vs hydrochlorothiazide of 0.93 (95% CI, 0.81-1.06), although 1 dose subgroup did reach statistical significance without adjustment for multiple hypotheses. An observational study of the MRFIT cohort by Dorsch et al^25^ showed a relative HR for chlorthalidone vs hydrochlorothiazide of 0.79 (95% CI, 0.68-0.92), but the doses for both drugs were high. A randomized clinical trial comparing hydrochlorothiazide with chlorthalidone currently in progress^2^ may provide more definitive information to inform drug choice.

Several factors may be contributing to the discordance between our effectiveness results and those of previous indirect network analyses. First, because we focus on first-time use of antihypertension drugs, we likely have higher proportion of patients with milder disease with less baseline cardiovascular disease risk than the randomized clinical trials included in the network analyses. Second, our time-at-risk periods may be shorter, but our sensitivity analysis showed no difference as we increased time at risk, and our previous study of hypertension drug classes,^13^ which used the same methods and databases and had the same follow-up times, was able to discern differences in effectiveness. Third, indirect network analysis is subject to bias^26^ if the underlying trials differ in populations of patients, in physician behavior, or in study design, and, similarly, our observational study may be subject to residual bias. A fourth factor, failure to account for differences in baseline blood pressure between the 2 drugs, does not appear to be a source of the disagreement based on the PanTher database results. Fifth, our 95% CI for the composite cardiovascular disease HR is 0.85 to 1.17, which does not rule out some superiority in either direction, although it does exclude the previous indirect network analysis results.

A simple difference in effective dose, with chlorthalidone known to have a greater-per-milligram potency for lowering blood pressure levels than hydrochlorothiazide,^6,7,27,28^ could underlie some of the observed differences in toxicities, but our dose-sensitivity analysis still revealed higher hypokalemia for chlorthalidone at a 1:2 chlorthalidone:hydrochlorothiazide dose ratio. Furthermore, if clinicians treat to similar blood pressure levels, then they may titrate the doses to levels with similar blood pressure reduction, explaining our lack of differences in effectiveness but not our differences in safety. Ernst et al^6^ found better chlorthalidone nighttime blood pressure control at a 1:2 dose ratio, which could explain some increased safety signals.

The literature inconsistently points to a difference in hypokalemia between the 2 drugs. At a 1:2 dose ratio, Ernst et al^6^ found little difference in potassium. At a 1:1 dose ratio, Bakris et al^7^ found no statistically significant difference in the hypokalemia rate although with only 5 events, the study lacked power. The network meta-analysis by Ernst et al^29^ found a small difference in potassium reduction. Two analyses of the MRFIT cohort^25,30^ showed increased reduction of potassium for chlorthalidone when both drugs were used at high doses (50 mg-100 mg). A study of real-world evidence by Dhalla et al^5^ showed odds ratios around 3 for hypokalemia, matching our results well, even in the 2 strata in which chlorthalidone was half the dose of hydrochlorothiazide; it also found an HR of 1.68 for hyponatremia.

### Limitations

Our main limitation is the possibility of residual confounding including confounding by indication, differences in physician characteristics that may be associated with drug choice, concomitant use of other drugs started after the index date, differences in blood pressure measurement error, and informative censoring at the end of the on-treatment periods. To minimize this risk, we used newer methods to account for bias and to detect residual bias through our negative and positive controls.

## Conclusions

Our findings based on currently available data and the most recent advances in observational research do not support the use of chlorthalidone over hydrochlorothiazide. This study found that chlorthalidone use was not associated with significant cardiovascular benefits when compared with hydrochlorothiazide, while its use was associated with greater risk of renal and electrolyte abnormalities. We acknowledge the possibility of residual confounding despite our analytic approach and diagnostics and look forward to the results of the ongoing randomized clinical trial.

## Notes

Supplement. eAppendix. eFigure 1. Covariate balance after inclusion of baseline blood pressure in the PanTher database eFigure 2. Sensitivity to balancing on baseline blood pressure in the PanTher database eFigure 3. Changes in blood potassium level in the PanTher database eFigure 4. Comparability of the populations for the three databases eFigure 5. Homogeneity on effectiveness and hypokalemia across databases and analytic approaches eFigure 6. Forest plot of safety and effectiveness outcomes across analytic approaches eFigure 7. Kaplan-Meier plots for effectiveness and hypokalemia outcomes across databases and analytic approaches eTable 1. Effectiveness and safety for CCAE for stratified on-treatment for limited dosing: 12.5mg chlorthalidone versus 25mg hydrochlorothiazide eTable 2. Effectiveness and safety for CCAE for matching on-treatment for limited dosing: 12.5mg chlorthalidone versus 25mg hydrochlorothiazide eTable 3. Baseline characteristics for CCAE with stratification eTable 4. Baseline characteristics for Optum with stratification eTable 5. Baseline characteristics for PanTher with stratification eTable 6. Baseline characteristics for CCAE with matching eTable 7. Baseline characteristics for Optum with matching eTable 8. Baseline characteristics for PanTher with matching eTable 9. Effectiveness and safety for meta-analysis for stratified on-treatment (these are the primary results reported in the main paper) eTable 10. Effectiveness and safety for meta-analysis for stratified intent-to-treat eTable 11. Effectiveness and safety for meta-analysis for matching on-treatment eTable 12. Effectiveness and safety for meta-analysis for matching intent-to-treat eReferences
