# Supplementary material for: Comparison of Cardiovascular and Safety Outcomes of Chlorthalidone vs Hydrochlorothiazide to Treat Hypertension
Source: JAMA Intern Med. 2020 Feb 17;180(4):542–51. doi: 10.1001/jamainternmed.2019.7454 (PMC7042845; doi:10.1001/jamainternmed.2019.7454)
Supplement: Supplement. — eAppendix. eFigure 1. Covariate balance after inclusion of baseline blood pressure in the PanTher database eFigure 2. Sensitivity to balancing on baseline blood pressure in the PanTher database eFigure 3. Changes in blood potassium level in the PanTher database eFigure 4. Comparability of the populations for the three databases eFigure 5. Homogeneity on effectiveness and hypokalemia across databases and analytic approaches eFigure 6. Forest plot of safety and effectiveness outcomes across analytic approaches eFigure 7. Kaplan-Meier plots for effectiveness and hypokalemia outcomes across databases and analytic approaches eTable 1. Effectiveness and safety for CCAE for stratified on-treatment for limited dosing: 12.5mg chlorthalidone versus 25mg hydrochlorothiazide eTable 2. Effectiveness and safety for CCAE for matching on-treatment for limited dosing: 12.5mg chlorthalidone versus 25mg hydrochlorothiazide eTable 3. Baseline characteristics for CCAE with stratification eTable 4. Baseline characteristics for Optum with stratification eTable 5. Baseline characteristics for PanTher with stratification eTable 6. Baseline characteristics for CCAE with matching eTable 7. Baseline characteristics for Optum with matching eTable 8. Baseline characteristics for PanTher with matching eTable 9. Effectiveness and safety for meta-analysis for stratified on-treatment (these are the primary results reported in the main paper) eTable 10. Effectiveness and safety for meta-analysis for stratified intent-to-treat eTable 11. Effectiveness and safety for meta-analysis for matching on-treatment eTable 12. Effectiveness and safety for meta-analysis for matching intent-to-treat eReferences [file jamainternmed-180-542-s001.pdf]

Supplementary Online Content

Hripcsak G, Suchard MA, Shea S, et al. Comparison of cardiovascular and safety outcomes of chlorthalidone vs hydrochlorothiazide to treat hypertension. *JAMA Intern Med*. Published online February 17, 2020. doi:10.1001/jamainternmed.2019.7454

eAppendix.
eFigure 1. Covariate balance after inclusion of baseline blood pressure in the PanTher database
eFigure 2. Sensitivity to balancing on baseline blood pressure in the PanTher database
eFigure 3. Changes in blood potassium level in the PanTher database
eFigure 4. Comparability of the populations for the three databases
eFigure 5. Homogeneity on effectiveness and hypokalemia across databases and analytic approaches
eFigure 6. Forest plot of safety and effectiveness outcomes across analytic approaches
eFigure 7. Kaplan-Meier plots for effectiveness and hypokalemia outcomes across databases and analytic approaches
eTable 1. Effectiveness and safety for CCAE for stratified on-treatment for limited dosing: 12.5mg chlorthalidone versus 25mg hydrochlorothiazide
eTable 2. Effectiveness and safety for CCAE for matching on-treatment for limited dosing: 12.5mg chlorthalidone versus 25mg hydrochlorothiazide
eTable 3. Baseline characteristics for CCAE with stratification
eTable 4. Baseline characteristics for Optum with stratification
eTable 5. Baseline characteristics for PanTher with stratification
eTable 6. Baseline characteristics for CCAE with matching
eTable 7. Baseline characteristics for Optum with matching
eTable 8. Baseline characteristics for PanTher with matching
eTable 9. Effectiveness and safety for meta-analysis for stratified on-treatment (these are the primary results reported in the main paper)
eTable 10. Effectiveness and safety for meta-analysis for stratified intent-to-treat
eTable 11. Effectiveness and safety for meta-analysis for matching on-treatment
eTable 12. Effectiveness and safety for meta-analysis for matching intent-to-treat
eReferences

This supplementary material has been provided by the authors to give readers additional information about their work.

© 2020 Hripcsak G. et al. *JAMA Internal Medicine.*

eAppendix.

1 Research methods 4
1.1 Study Design 4
1.2 Data Source(s) 4
1.2.1 Truven MarketScan Commercial Claims and Encounters (CCAE) 4
1.2.2 Optum ClinFormatics (Optum) 4
1.2.3 Optum® de-identified Electronic Health Record Dataset (PanTher) 4
1.3 Study population 5
1.4 Exposures and outcomes 5
1.5 Negative control outcomes 6
1.6 Positive control outcomes 7
1.7 Covariates 8
1.7.1 Propensity score covariates 8
1.8 Index event and calculation of time-at risk 8
1.9 Model Specification 9
1.9.1 Pooling effect estimates across databases 10
1.10 Analyses 10
1.10.1 Comparative analyses 10
1.11 Output 10
1.12 Evidence Evaluation 10
1.13 Strengths and Limitations of the Research Methods 11
1.14 Protection of Human Subjects 11
1.15 Management and Reporting of Adverse Events and Adverse Reactions 11
1.16 Plans for Disseminating and Communicating Study Results 11
2 Supplementary results 11
2.1 Ad hoc sensitivity analyses (blood pressure, potassium, and dose) defined in the main report 11
2.2 Baseline characteristics for each database for stratification and matching 19
2.3 Effectiveness and safety results for the meta-analysis for each analytic approach (stratified vs. matching; on-treatment vs. intent-to-treat) 31
2.4 Comparability of the populations for the three databases 39
2.5 Homogeneity on effectiveness and hypokalemia across databases and analytic approaches 41
2.6 Forest plot of safety and effectiveness outcomes across analytic approaches 48
2.7 Kaplan-Meier plots for effectiveness and hypokalemia outcomes across databases and analytic approaches 51
3 Detailed definitions of exposures and outcomes 82
3.1 Drug exposures 82
3.2 Outcomes 83
3.2.1 Abdominal pain 87
3.2.2 Abnormal weight gain 87
3.2.3 Abnormal weight loss 88
3.2.4 Acute myocardial infarction 88
3.2.5 Acute pancreatitis 90
3.2.6 Acute renal failure 91
3.2.7 All-cause mortality 92
3.2.8 Anaphylactoid reaction 93
3.2.9 Anemia 95
3.2.10 Angioedema 96
3.2.11 Anxiety 97

© 2020 Hripcsak G. et al. *JAMA Internal Medicine.*

3.2.12 Bradycardia 101
3.2.13 Cardiac arrhythmia 102
3.2.14 Cardiovascular disease 104
3.2.15 Cardiovascular-related mortality 108
3.2.16 Chest pain or angina 111
3.2.17 Chronic kidney disease 112
3.2.18 Cough 115
3.2.19 Decreased libido 116
3.2.20 Dementia 116
3.2.21 Depression 117
3.2.22 Diarrhea 120
3.2.23 End stage renal disease 121
3.2.24 Fall 123
3.2.25 Gastrointestinal bleeding 123
3.2.26 Gout 128
3.2.27 Headache 128
3.2.28 Heart failure 129
3.2.29 Hemorrhagic stroke 130
3.2.30 Hepatic failure 131
3.2.31 Hospitalization with heart failure 132
3.2.32 Hospitalization with preinfarction syndrome 133
3.2.33 Hyperkalemia 134
3.2.34 Hypokalemia 135
3.2.35 Hypomagnesemia 136
3.2.36 Hyponatremia 137
3.2.37 Hypotension 137
3.2.38 Impotence 138
3.2.39 Ischemic stroke 139
3.2.40 Malignant neoplasm 140
3.2.41 Measured renal dysfunction 151
3.2.42 Nausea 152
3.2.43 Neutropenia or agranulocytosis 153
3.2.44 Rash 154
3.2.45 Rhabdomyolysis 155
3.2.46 Stroke 157
3.2.47 Sudden cardiac death 158
3.2.48 Syncope 160
3.2.49 Thrombocytopenia 161
3.2.50 Transient ischemic attack 161
3.2.51 Type 2 diabetes mellitus 163
3.2.52 Vasculitis 167
3.2.53 Venous thromboembolic events 169
3.2.54 Vertigo 171
3.2.55 Vomiting 172
4 References 173

© 2020 Hripcsak G. et al. *JAMA Internal Medicine.*

1 Research methods

1.1 Study Design

This study is a retrospective, observational, new-user cohort study. By 'retrospective' we mean the study uses data already collected at the start of the study. By 'observational' we mean no intervention takes place in the course of this study. By 'new-user' we mean we only analyze the first exposure of a subject to the treatment of interest. By 'cohort study' we mean two cohorts, a target and comparator cohort, are followed from index date (start of first exposure) to some end date, and assessed for the occurrence of the outcomes of interest.

1.2 Data Source(s)

The analyses are performed across a network of observational healthcare databases. All databases have been transformed into the OMOP Common Data Model, version 4 or OMOP Common Data Model, version 5. The complete specification for OMOP Common Data Model is available [2]. The following databases are included in this analysis:

- IBM MarketScan Commercial Claims and Encounters (CCAE)
- Optum ClinFormatics (Optum)
- Optum® de-identified Electronic Health Record Dataset (PanTher)

1.2.1 Truven MarketScan Commercial Claims and Encounters (CCAE)

Truven Health MarketScan® Commercial Claims and Encounters Database (CCAE) represent data from individuals enrolled in United States employer-sponsored insurance health plans. The data includes adjudicated health insurance claims (e.g. inpatient, outpatient, and outpatient pharmacy) as well as enrollment data from large employers and health plans who provide private healthcare coverage to employees, their spouses, and dependents. Additionally, it captures laboratory tests for a subset of the covered lives. This administrative claims database includes a variety of fee-for-service, preferred provider organizations, and capitated health plans.

1.2.2 Optum ClinFormatics (Optum)

Optum Clinformatics Extended DataMart is an adjudicated US administrative health claims database for members of private health insurance, who are fully insured in commercial plans or in administrative services only (ASOs), Legacy Medicare Choice Lives (prior to January 2006), and Medicare Advantage (Medicare Advantage Prescription Drug coverage starting January 2006). The population is primarily representative of commercial claims patients (0-65 years old) with some Medicare (65+ years old) however ages are capped at 90 years. It includes data captured from administrative claims processed from inpatient and outpatient medical services and prescriptions as dispensed, as well as results for outpatient lab tests processed by large national lab vendors who participate in data exchange with Optum. This dataset also provides date of death (month and year only) for members with both medical and pharmacy coverage from the Social Security Death Master File (however after 2011 reporting frequency changed due to changes in reporting requirements) and location information for patients is at the US state level.

1.2.3 Optum® de-identified Electronic Health Record Dataset (PanTher)

Optum© de-identified Electronic Health Record Dataset represents Humedica's Electronic Health Record data a medical records database. The medical record data includes clinical information, inclusive of prescriptions as prescribed and administered, lab results, vital signs, body measurements, diagnoses, procedures, and information derived from clinical Notes using Natural Language Processing (NLP).

© 2020 Hripcsak G. et al. *JAMA Internal Medicine.*

1.3 Study population

All subjects in the database will be included who meet the following criteria: (note: the index date is the start of the first treatment for hypertension)

- Exposure to one of the treatments of interest
- At least 365 days of observation time prior to the index date
- No exposure of any hypertension treatment before the index date
- A diagnosis of hypertensive disorder on or preceding the index date
- No diagnosis of the outcome of interest preceding the index date

For the purpose of determining previous hypertension treatment, the following medications were used:

Acebutolol	Felodipine	Nicardipine
Aliskiren	Fosinopril	Nifedipine
Amiloride	Furosemide	Nisoldipine
Amlodipine	Guanfacine	Olmesartan
Atenolol	Hydralazine	Penbutolol
Azilsartan	Hydrochlorothiazide	Perindopril
Benazepril	Indapamide	Pindolol
Betaxolol	Irbesartan	Prazosin
Bisoprolol	Isradipine	Propranolol
Bumetanide	Labetalol	Quinapril
Candesartan	Lisinopril	Ramipril
Captopril	Losartan	Spironolactone
Carvedilol	Methyldopa	Telmisartan
Chlorthalidone	Metolazone	Terazosin
Clonidine	Metoprolol	Torsemide
Diltiazem	Minoxidil	Trandolapril
Doxazosin	Moexipril	Triamterene
Enalapril	Nadolol	Valsartan
Eplerenone	Nebivolol	Verapamil
Eprosartan		

1.4 Exposures and outcomes

The two study exposures were chlorthalidone and hydrochlorothiazide in any dose. A detailed definition of the exposures is given in Section 3.

The outcomes for effectiveness and safety were selected based on their relevance to hypertension treatment (not limited to chlorthalidone and hydrochlorothiazide) as part of the OHDSI LEGEND study. The effectiveness outcomes were acute myocardial infarction (AMI) (hospitalized), hospitalization for heart failure (HF), ischemic or hemorrhagic stroke (also hospitalized), and a composite cardiovascular disease outcome including the first three outcomes and sudden cardiac death. Fifty-five safety outcomes were included. All outcomes are enumerated and defined in Section 3.

Outcomes were selected based on being initially identified from endpoints from clinical trials reporting in clinical guidelines and systematic reviews, and augmented by adverse events listed on US structured product labels of hypertension drugs [3]. Each outcome was then assessed to determine if it was evaluable in observational data by developing and evaluating an operational

© 2020 Hripcsak G. et al. *JAMA Internal Medicine.*

phenotype definition. All phenotypes were designed, implemented and evaluated using the same approach: Initial design was guided by literature review in PubMed of prior observational studies of administrative claims or electronic health records where the phenotype was used as a study outcome, and if available, validated through source record verification or other means. Literature review was complemented by review of eMERGE PheKB phenotype entries [4]. Clinical definitions of cardiovascular outcomes were determined from clinical guidelines and systematic review of clinical trials of hypertension treatments [5-7]. Where possible, concept sets originated with published code lists (e.g. ICD-9-CM and ICD-10) and were augmented through lexical search and semantic exploration of the OHDSI standardized vocabularies. Cohort definitions and associated concept sets were then reviewed by a clinical adjudicator. Cohort definitions were developed using the OHDSI open-source platform ATLAS [8]. These definitions were initially executed across seven databases (the three from this study, CCAE, Optum, and PanTher, and four others: IBM MarketScan Medicare Supplemental Database, IBM MarketScan Multi-State Medicaid Database, Japan Medical Data Center Claims Database, IQVIA IMS Germany) to identify qualifying patients. Because these databases do not all consistently contain laboratory values, outcomes involving electrolyte imbalance (hypokalemia, hypomagnesemia, hyponatremia) were defined by diagnosis records only. These cohorts were used to produce characterization of outcome incidence, stratified by age decile, gender, and index year, to determine if outcomes were being observed in a frequency that was generally consistent across data sources and sufficiently aligned with epidemiologic expectations of the disease. No source record verification or other methods for validation was performed.

1.5 Negative control outcomes

Negative controls are concepts known to not be associated with the target or comparator cohorts, such that we can assume the true relative risk between the two cohorts is 1. Negative controls are selected using a similar process to that outlined by Voss et al. [9]. Person counts of all potential drug-condition pairs are reviewed in observational data; this person count data helps determine which pairs are even probable for use in calibration. Given the list of potential drug-condition pairs, the concepts in the pairs must meet the following requirements to be considered as negative controls: (1) that there is no Medline abstract where the MeSH terms suggest an association between the drug and the condition [10], (2) that there is no mention of the drug-condition pair on a US Product Label in the “Adverse Drug Reactions” or “Postmarketing” section [11], (3) there are no US spontaneous reports suggesting that the pair is in an adverse event relationship [12,13], (4) that the OMOP Vocabulary does not suggest that the drug is indicated for the condition, (5) that the concepts are usable (i.e. not too broad, not suggestive of an adverse event relationship, not pregnancy related), and (6) the exact concept itself is utilized in patient level data (i.e. concepts that are not usually used within the data are usually indicative a broad concept that has a child that is more specific). The remaining concepts are “optimized”, meaning parent concepts remove children as defined by the OMOP Vocabulary (e.g. if both “Non-Hodgkin’s Lymphoma” and “B-Cell Lymphoma” we selected, child concept “B-Cell Lymphoma would be removed for its parent “Non-Hodgkin’s Lymphoma”). Once potential negative control candidates were selected, manual clinical review to exclude any pairs that may still be in a causal relationship or similar to the study outcome was be performed to select the top concepts by patient exposure. The final list can be found here:

Abnormal cervical smear	Homocystinuria
Abnormal pupil	Human papilloma virus infection
Abrasion and/or friction burn of trunk without infection	Ileostomy present
Absence of breast	Impacted cerumen

© 2020 Hripcsak G. et al. *JAMA Internal Medicine.*

Absent kidney	Impingement syndrome of shoulder region
Acid reflux	Ingrowing nail
Acquired hallux valgus	Injury of knee
Acquired keratoderma	Irregular periods
Acquired trigger finger	Kwashiorkor
Acute conjunctivitis	Late effect of contusion
Amputated foot	Late effect of motor vehicle accident
Anal and rectal polyp	Leukorrhea
Burn of forearm	Macular drusen
Calcaneal spur	Melena
Cannabis abuse	Nicotine dependence
Cervical somatic dysfunction	Noise effects on inner ear
Changes in skin texture	Nonspecific tuberculin test reaction
Chondromalacia of patella	Non-toxic multinodular goiter
Cocaine abuse	Onychomycosis due to dermatophyte
Colostomy present	Opioid abuse
Complication due to Crohn's disease	Passing flatus
Contact dermatitis	Postviral fatigue syndrome
Contusion of knee	Presbyopia
Crohn's disease	Problem related to lifestyle
Derangement of knee	Psychalgia
Difficulty sleeping	Ptotic breast
Disproportion of reconstructed breast	Regular astigmatism
Effects of hunger	Senile hyperkeratosis
Endometriosis	Somatic dysfunction of lumbar region
Epidermoid cyst	Splinter of face, without major open wound
Feces contents abnormal	Sprain of ankle
Foreign body in orifice	Strain of rotator cuff capsule
Ganglion cyst	Tear film insufficiency
Genetic predisposition	Tobacco dependence syndrome
Hammer toe	Vaginitis and vulvovaginitis
Hereditary thrombophilia	Verruca vulgaris
Herpes zoster without complication	Wrist joint pain
High risk sexual behavior	Wristdrop

For each negative control outcome, a patient enters the negative control outcome cohort at the occurrence of a diagnose code identified by the concepts listed above, or any one of its descendant codes.

1.6 Positive control outcomes

In addition to negative control outcomes, we will also include synthetic positive control outcomes. These are outcomes based on the real negative controls, but where the true effect size

© 2020 Hripcsak G. et al. *JAMA Internal Medicine.*

is artificially increased to a desired effect size by injection of additional, simulated outcomes [14]. To preserve confounding, these additional outcomes are sampled from predicted probabilities generated using a fitted predictive model. For each negative control outcome, three positive control outcomes are generated with true relative risk is 1.5, 2, and 4. Using both negative and positive controls, we fit a systematic error model and perform confidence interval calibration [14].

1.7 Covariates

1.7.1 Propensity score covariates

Propensity scores (PS) are used as an analytic strategy to reduce potential confounding due to imbalance between the target and comparator cohorts in baseline covariates. The propensity score is the probability of a patient being classified in the target cohort vs. the comparator cohort, given a set of observed covariates.

The types of baseline covariates used to fit the propensity score model will be:

- Demographics
    - Gender
    - Age group (5-year bands)
    - Index year
    - Index month
- Conditions
    - In prior 30d
    - In prior 365d
- Condition aggregation
    - SNOMED
- Drugs
    - In prior 30d
    - In prior 365d
    - Overlapping index date
- Drug aggregation
    - Ingredient
    - ATC Class
- Risk scores
    - Charlson comorbidity index

All covariates that occur in less than 0.1% of the persons between the target and comparator cohorts combined are excluded prior to model fitting for computational efficiency. Each condition, drug, class, etc. is counted as a separate covariate, resulting in over 60,000 covariates per database for this study.

1.8 Index event and calculation of time-at risk

The index event for this study is the first treatment with chlorthalidone or hydrochlorothiazide. It must be taken as a single anti-hypertensive agent, and no other anti-hypertensive agents may precede them. Subjects are thus new users of one of the two study drugs and new users of anti-hypertensive agents in general.

Each subject must be observed for at least 365 days before the index event. Baseline covariates are collected from this period up to but not including the index event.

Two time-at-risk periods are used:

© 2020 Hripcsak G. et al. *JAMA Internal Medicine.*

- **On-treatment**. Starting one day after the index event and stopping at the termination of the drug or on the addition of a second anti-hypertensive agent. A maximum gap of 30 days is allowed between prescriptions, and the treatment is considered to end after the last prescription runs out.

- **Intent-to-treat** (ITT): Starting one day after the index event and stopping at the end of observation in the database.

1.9 Model Specification

In this study, we compare the chlorthalidone (target) cohort with the hydrochlorothiazide (comparator) cohort for the hazards of outcome during the time-at-risk by applying a Cox proportional hazards model.

The time-to-event of outcome among patients in the target and comparator cohorts is determined by calculating the number of days from the start of the time-at-risk window (the cohort start date), until the earliest event among 1) the first occurrence of the outcome, 2) the end of the time-at-risk window as defined above (i.e. 'on-treatment' or 'intent-to-treat'), and 3) the end of the observation period that spans the time-at-risk start.

Patients with the outcome observed prior to target or comparator cohort entry are excluded from consideration.

We conduct our cohort study using the open-source OHDSI CohortMethod R package [15], whose large-scale analytics are achieved through the Cyclops R package [16]. We use propensity scores as an analytic strategy to reduce potential confounding due to imbalance between the target and comparator cohorts in baseline covariates [17]. The propensity score is the probability of a patient being classified in the target cohort vs. the comparator cohort, given a set of observed covariates. In this study, the propensity score is estimated for each patient, using the predicted probability from a regularized logistic regression model, fit with a Laplace prior (LASSO) and the regularization hyperparameter selected by optimizing the likelihood in a 10-fold cross, a starting variance of 0.01 and a tolerance of 2e-7. Covariates to be used in the propensity score model are listed above; they include patient demographics, drug, condition, and procedure covariates generated through the FeatureExtraction R package [18]. The target cohort and comparator cohorts are stratified into ten quantiles of the propensity score distribution. The final outcome model applies a conditional Cox proportional hazard model, conditioned on the propensity score strata. We present PS and covariate balance metrics to assess successful confounding control, and provide HR estimates and Kaplan-Meier survival plots for the outcomes.

Residual study bias from unmeasured and systematic sources can exist in observational studies after controlling for measured confounding [19,20]. To estimate such residual bias, we conduct negative control outcome experiments with 76 negative control outcomes identified through a data-rich algorithm [9]. We fit the negative control estimates to an empirical null distribution that characterizes the study residual bias and is an important artifact from which to assess the study design [21]. Using the empirical null distribution and synthetic positive controls [14], we additionally calibrate all HR estimates, their 95% confidence intervals (CIs) and the p-value to reject the null hypothesis of no differential effect (HR = 1). Empirical calibration serves as an important diagnostic tool to evaluate if residual systematic error is sufficient to cast doubt on the accuracy of the unknown effect estimates.

We compute incidence rates for each outcome in each exposure group, in both the on-treatment and intent-to-treat windows.

© 2020 Hripcsak G. et al. *JAMA Internal Medicine.*

1.9.1 Pooling effect estimates across databases

Effects are pooled across databases using a random-effects meta-analysis. Estimates for negative and positive controls are pooled before performing empirical calibration on the pooled estimates.

1.10 Analyses

1.10.1 Comparative analyses

The following comparative analyses are performed. The above three databases were chosen based on having at least 2,500 subjects observed in both target and comparator cohorts:

- 56 outcomes of interest
- 2 time-at-risk definitions: on-treatment and intent-to-treat

We also include 76 negative control outcomes, and 3 * 76 synthetic positive control outcomes, so 304 control outcomes.

1.11 Output

All output is stored in the LEGEND evidence model, available at the OHDSI LEGEND evidence web site [22].

1.12 Evidence Evaluation

We execute the following analysis diagnostics:

- Propensity score distribution, shown as a plot of the preference score distribution [23]
- Covariate balance before and after propensity score matching, with the standardized difference of the means of the covariate under 0.1 being considered adequate balance
- Estimation for negative and positive controls, to assess residual error
- Negative and positive control exposures and outcomes are used to evaluate the potential impact of residual systematic error in the study design, and to facilitate empirical calibration of the p-value and confidence interval for the exposures and outcome of interest.

Negative control outcomes in the context of this study are outcomes that are not believed to have been caused by either exposure in any comparison and where therefore the true hazard ratio is equal to 1. We will execute the same analysis used for the primary hypotheses to produce hazard ratio estimates for the negative controls. The distribution of effect estimates across all negative controls are used to fit an empirical null distribution, which models the observed residual systematic error. The empirical null distribution is then applied to the target exposures and outcome of interest to calibrate the p-value [19].

Positive control exposures and outcomes are pairs of exposures and outcomes where the hazard ratio is known to be of some magnitude greater than 1. We synthesize positive controls by starting with the negative controls defined earlier, and adding additional, simulated outcomes during the time-at-risk until the desired true hazard ratio is achieved. The target hazard ratios are 1.5, 2 and 4. The negative and positive controls together are used to estimate an empirical systematic error model, which inform whether systematic error changes as a function of true effect size. The empirical systematic error model is then applied to the target the target exposures and outcome of interest to calibrate the confidence interval [14].

© 2020 Hripcsak G. et al. *JAMA Internal Medicine.*

Empirical calibration serves as an important diagnostic tool to evaluate if the residual systematic error is sufficient to cast doubt on the accuracy of the unknown effect estimate. The calibration effect plot and calibration probability plots are generated for review. We report the traditional and empirically calibrated p-value and confidence interval for each negative control, as well as the hypothesis of interest.

1.13 Strengths and Limitations of the Research Methods

Strengths

- Cohort studies allow direct estimation of incidence rates following exposure of interest, and the new-user design can capture early events following treatment exposures while avoiding confounding from previous treatment effects. New use allows for a clear exposure index date.
- PS matching allows balancing on a large number of baseline potential confounders.
- Use of negative and positive control outcomes allows for evaluating the study design as a whole in terms of residual bias.

Limitations

- Even though many potential confounders will be included in this study, there may be residual bias due to unmeasured or misspecified confounders.

1.14 Protection of Human Subjects

The study uses only de-identified data. Confidentiality of patient records is maintained at all times. All study reports contain aggregate data only and do not identify individual patients or physicians.

1.15 Management and Reporting of Adverse Events and Adverse Reactions

This study uses coded data that already exist in an electronic database. In this type of database, it is not possible to link (i.e., identify a potential causal association between) a particular product and medical event for any individual. Thus, the minimum criteria for reporting an adverse event (i.e., identifiable patient, identifiable reporter, a suspect product, and event) are not available and adverse events are not reportable as individual adverse events reports. The study results will be assessed for medically important results.

1.16 Plans for Disseminating and Communicating Study Results

The study results are posted on the OHDSI LEGEND evidence web site [22].

2 Supplementary results

2.1 Ad hoc sensitivity analyses (blood pressure, potassium, and dose) defined in the main report

© 2020 Hripcsak G. et al. *JAMA Internal Medicine.*

eFigure 1. Covariate balance after inclusion of baseline blood pressure in the PanTher database. After inclusion of baseline blood pressure in the propensity model, all 60,535 covariates had a standardized difference of the mean less than 0.1.

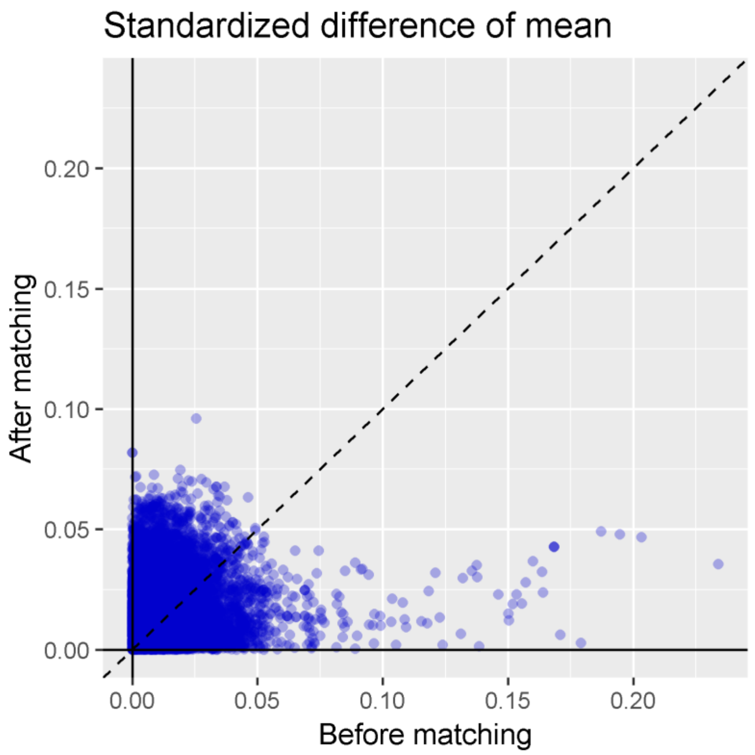

© 2020 Hripcsak G. et al. *JAMA Internal Medicine.*

eFigure 2. Sensitivity to balancing on baseline blood pressure in the PanTher database. We show effectiveness and safety outcomes for the PanTher database for propensity models that exclude (blue triangle) and include (red circle) baseline systolic and diastolic blood pressure in the propensity model. There are no major shifts in outcome.

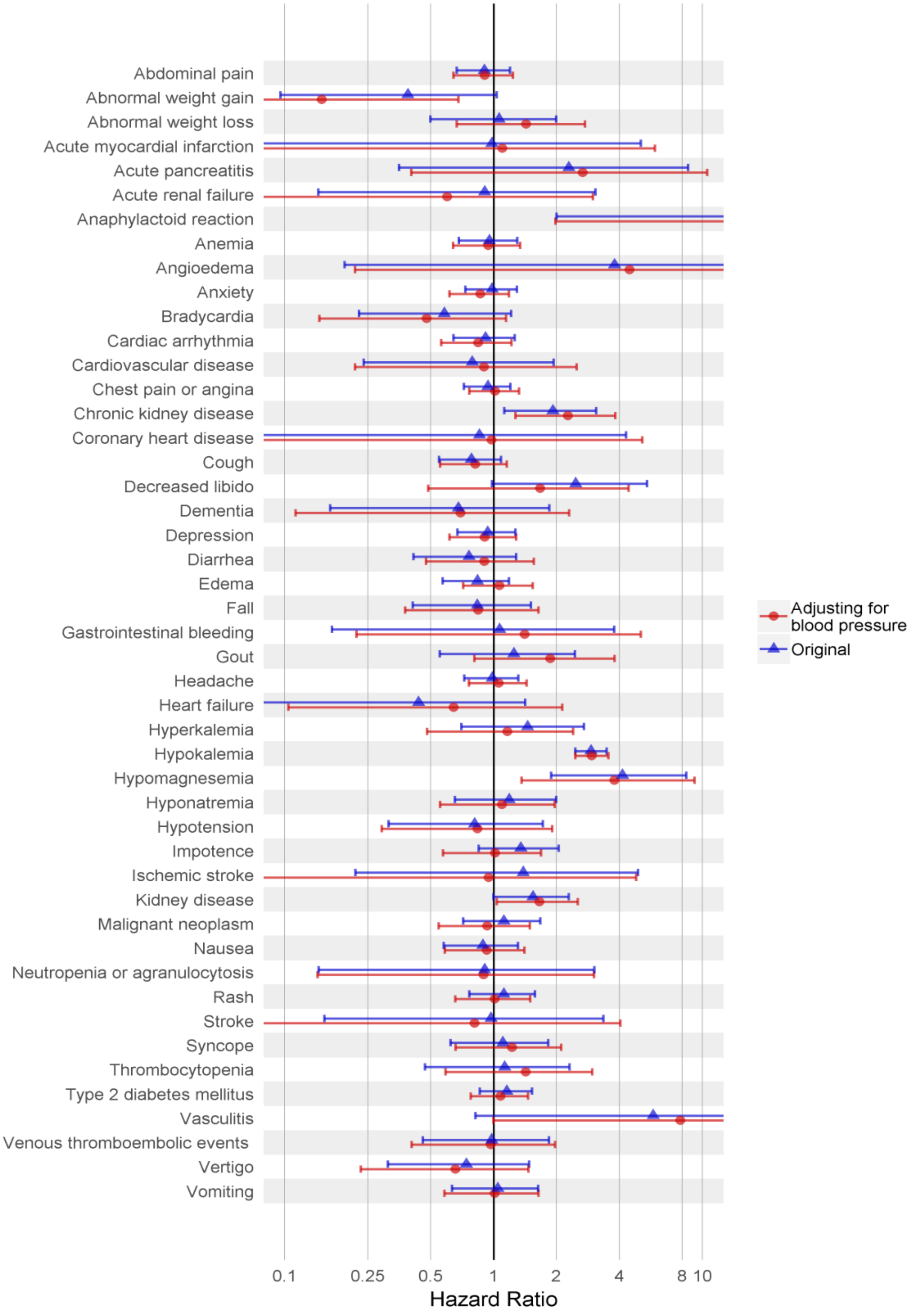

© 2020 Hripcsak G. et al. *JAMA Internal Medicine.*

eFigure 3. Changes in blood potassium level in the PanTher database. For each propensity score stratum, 1 to 10, the plots show the distribution of potassium levels for chlorthalidone (red) and hydrochlorothiazide (blue) before (lower row) and after (upper row) treatment. The sizes of the strata are shown at the bottom. Chlorthalidone shows a leftward (lower potassium) shift after treatment.

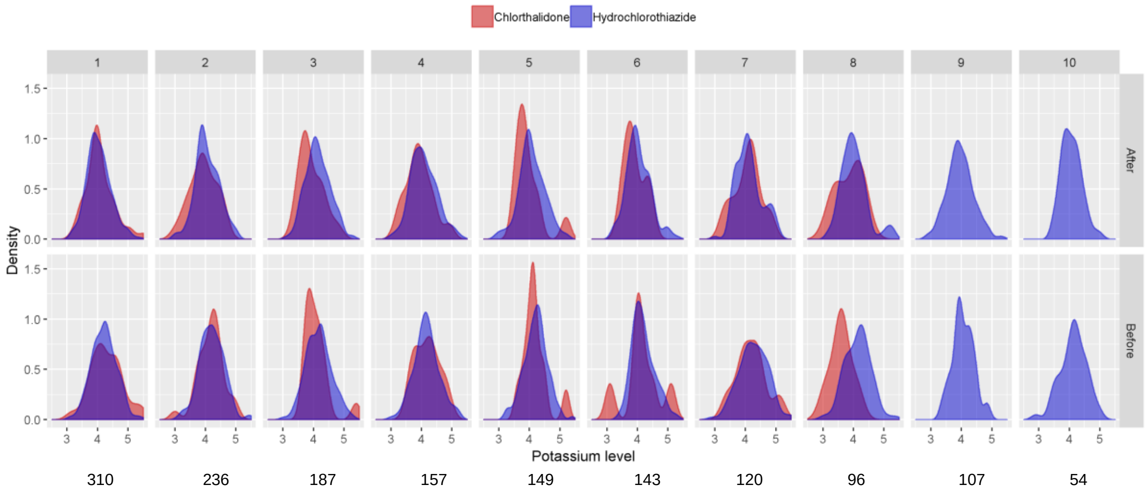

© 2020 Hripcsak G. et al. *JAMA Internal Medicine.*

eTable 1. Effectiveness and safety for CCAE for stratified on-treatment for limited dosing: 12.5mg chlorthalidone versus 25mg hydrochlorothiazide

	Chlorthalidone[#]		Hydrochloro-thiazide		Hazard ratio uncalibrated			Calibrated hazard ratio		
Outcome	Events	Subjects	Events	Subjects	HR	Lower CI	Upper CI	HR	Lower CI	Upper CI
Acute myocardial infarction	0	2555	169	160069	0.36	NA	1.17	NA	NA	NA
All-cause mortality	0	2559	38	160232	0.36	NA	5.63	NA	NA	NA
Composite cardiovascular disease	5	2543	490	159087	0.70	0.25	1.53	0.66	0.27	1.61
Cardiovascular-related mortality	0	2559	16	160232	0.36	NA	24.35	NA	NA	NA
Chest pain or angina	81	2014	6237	128478	1.03	0.82	1.27	0.96	0.76	1.22
Sudden cardiac death	<5	2559	42	160209	1.89	0.10	9.11	1.73	0.20	15.39
Hospitalization with preinfarction syndrome	0	2553	196	159771	0.36	NA	1.25	NA	NA	NA
Bradycardia	<5	2536	378	158857	0.39	0.06	1.20	0.37	0.09	1.55
Cardiac arrhythmia	35	2412	2045	152592	1.12	0.78	1.54	1.04	0.74	1.47
Syncope	12	2492	1238	156306	0.69	0.37	1.16	0.65	0.36	1.15
Heart failure	<5	2558	141	160059	1.47	0.36	3.97	1.36	0.42	4.42
Hospitalization with heart failure	<5	2555	143	159945	1.47	0.36	3.96	1.35	0.42	4.41
Fall	8	2516	553	157872	0.75	0.34	1.41	0.71	0.35	1.42
Headache	60	2098	3668	132551	1.23	0.94	1.58	1.14	0.87	1.51
Hemorrhagic stroke	0	2557	54	160067	0.36	NA	3.15	NA	NA	NA
Ischemic stroke	<5	2553	159	159648	0.81	0.13	2.57	0.76	0.18	3.24
Stroke	<5	2551	193	159512	0.70	0.12	2.21	0.66	0.15	2.79
Transient ischemic attack	<5	2543	160	159539	0.83	0.14	2.65	0.78	0.18	3.33
Vertigo	18	2506	676	157697	2.03	1.21	3.16	1.86	1.15	3.02
Anxiety	28	2311	2576	146132	0.67	0.45	0.96	0.63	0.43	0.92
Decreased libido	<5	2528	214	159398	0.99	0.30	2.35	0.92	0.34	2.52
Dementia	0	2558	74	159935	0.36	NA	3.52	NA	NA	NA
Depression	24	2233	2359	141473	0.77	0.50	1.13	0.72	0.48	1.09
Impotence	17	2471	961	156601	0.95	0.56	1.48	0.88	0.54	1.44
Abdominal pain	71	1895	5281	123593	1.13	0.89	1.42	1.05	0.82	1.36
Abnormal weight gain	11	2446	963	153751	0.82	0.42	1.42	0.77	0.42	1.40
Abnormal weight loss	<5	2524	483	158436	0.40	0.10	1.06	0.39	0.12	1.22
Acute pancreatitis	<5	2552	133	159838	0.54	0.03	2.45	0.51	0.06	4.35
Diarrhea	16	2373	1597	151894	0.76	0.44	1.20	0.71	0.43	1.17
Gastrointestinal bleeding	<5	2550	151	159520	0.55	0.03	2.50	0.52	0.06	4.44
Hepatic failure	<5	2558	43	160143	1.78	0.10	8.44	1.63	0.19	14.37
Nausea	39	2381	2031	150203	1.30	0.93	1.76	1.20	0.87	1.68
Type 2 diabetes mellitus	30	2445	2927	152869	0.81	0.55	1.14	0.76	0.52	1.09
Vomiting	20	2426	1476	152769	0.98	0.61	1.49	0.92	0.59	1.44
Acute renal failure	<5	2547	214	159909	0.76	0.19	2.02	0.72	0.22	2.29
Chronic kidney disease	7	2534	376	159699	1.15	0.49	2.26	1.07	0.50	2.27
End stage renal disease	0	2558	6	160220	0.36	NA	18.50	NA	NA	NA
Hyperkalemia	6	2546	336	159549	1.07	0.42	2.20	0.99	0.44	2.25
Hypokalemia	87	2523	3161	157974	1.71*	1.37	2.11	1.57*	1.25	2.01
Hypomagnesemia	<5	2554	150	159961	1.41	0.43	3.40	1.31	0.47	3.61
Hyponatremia	11	2550	471	159765	1.30	0.67	2.26	1.20	0.66	2.21
Hypotension	<5	2551	401	159496	0.53	0.13	1.38	0.50	0.16	1.59
Measured renal dysfunction	0	2556	30	160192	0.36	NA	3.97	NA	NA	NA
Anemia	28	2295	2919	144654	0.73	0.49	1.04	0.69	0.47	1.00

© 2020 Hripcsak G. et al. *JAMA Internal Medicine.*

Malignant neoplasm	14	2477	842	155680	1.31	0.73	2.14	1.21	0.71	2.08
Neutropenia or agranulocytosis	<5	2548	188	159576	0.46	0.03	2.09	0.44	0.05	3.73
Thrombocytopenia	<5	2530	222	159446	1.07	0.33	2.55	1.00	0.36	2.73
Anaphylactoid reaction	0	2558	20	160148	0.36	NA	7.73	NA	NA	NA
Angioedema	<5	2558	81	159977	0.83	0.05	3.82	0.78	0.09	6.67
Cough	60	2076	4513	137078	0.97	0.74	1.24	0.90	0.69	1.18
Gout	8	2527	688	157937	0.80	0.36	1.50	0.75	0.37	1.51
Rash	30	2334	2355	148737	0.85	0.58	1.20	0.79	0.55	1.15
Rhabdomyolysis	<5	2555	24	160162	1.86	0.10	9.17	1.71	0.19	15.37
Vasculitis	0	2558	11	160147	0.35	NA	9.94	NA	NA	NA
Venous thromboembolic events	10	2515	533	157665	1.40	0.69	2.49	1.29	0.69	2.44

[#] A "<" in front of a number implies a count less than 5.
* Statistically significant after Bonferroni correction for 55 hypotheses.
NA implies hazard ratio or bound could not be estimated

© 2020 Hripcsak G. et al. *JAMA Internal Medicine.*

eTable 2. Effectiveness and safety for CCAE for matching on-treatment for limited dosing: 12.5mg chlorthalidone versus 25mg hydrochlorothiazide

	Chlorthalidone#		Hydrochloro-thiazide		Hazard ratio uncalibrated			Calibrated hazard ratio		
Outcome	Events	Subjects	Events	Subjects	HR	Lower CI	Upper CI	HR	Lower CI	Upper CI
Acute myocardial infarction	0	2555	113	107259	0.34	NA	1.09	NA	NA	NA
All-cause mortality	0	2559	20	107334	0.35	NA	7.66	NA	NA	NA
Composite cardiovascular disease	5	2543	327	106680	0.60	0.21	1.39	0.58	0.23	1.53
Cardiovascular-related mortality	0	2559	11	107334	0.36	NA	44.00	NA	NA	NA
Chest pain or angina	81	2014	3963	86760	1.00	0.78	1.25	0.95	0.67	1.37
Sudden cardiac death	<5	2559	31	107322	1.70	0.09	9.62	1.60	0.16	15.63
Hospitalization with preinfarction syndrome	0	2553	113	107116	0.33	NA	1.20	NA	NA	NA
Bradycardia	<5	2536	244	106538	0.37	0.06	1.20	0.36	0.09	1.60
Cardiac arrhythmia	35	2412	1424	102396	1.09	0.75	1.52	1.04	0.67	1.60
Syncope	12	2492	829	104917	0.63	0.31	1.13	0.61	0.31	1.22
Heart failure	<5	2558	92	107215	1.42	0.33	4.10	1.34	0.39	4.61
Hospitalization with heart failure	<5	2555	96	107168	1.47	0.35	4.23	1.39	0.40	4.74
Fall	8	2516	445	105387	0.61	0.25	1.26	0.59	0.27	1.36
Headache	60	2098	2471	88796	1.10	0.82	1.44	1.05	0.71	1.54
Hemorrhagic stroke	0	2557	29	107273	0.34	NA	4.96	NA	NA	NA
Ischemic stroke	<5	2553	114	106951	0.71	0.11	2.43	0.69	0.15	3.09
Stroke	<5	2551	129	106905	0.61	0.10	2.11	0.59	0.13	2.69
Transient ischemic attack	<5	2543	111	106830	1.10	0.17	3.76	1.04	0.23	4.71
Vertigo	18	2506	446	105404	1.97	1.11	3.27	1.85	1.00	3.24
Anxiety	28	2311	1933	96925	0.78	0.52	1.12	0.75	0.48	1.20
Decreased libido	<5	2528	164	106510	0.93	0.27	2.36	0.88	0.30	2.59
Dementia	0	2558	40	107240	0.33	NA	3.13	NA	NA	NA
Depression	24	2233	1586	94091	0.81	0.52	1.20	0.78	0.48	1.28
Impotence	17	2471	723	104351	0.90	0.52	1.44	0.86	0.50	1.51
Abdominal pain	71	1895	3350	81681	1.17	0.90	1.48	1.11	0.76	1.59
Abnormal weight gain	11	2446	669	102539	1.01	0.52	1.76	0.96	0.50	1.84
Abnormal weight loss	<5	2524	345	105732	0.42	0.10	1.14	0.41	0.12	1.37
Acute pancreatitis	<5	2552	92	107044	1.08	0.06	5.16	1.02	0.12	8.98
Diarrhea	16	2373	1067	101107	0.78	0.44	1.28	0.75	0.43	1.36
Gastrointestinal bleeding	<5	2550	103	106887	0.31	0.02	1.62	0.31	0.03	2.86
Hepatic failure	<5	2558	30	107290	1.67	0.09	8.79	1.57	0.17	14.60
Nausea	39	2381	1458	100719	1.30	0.90	1.80	1.23	0.79	1.87
Type 2 diabetes mellitus	30	2445	1871	102419	0.76	0.50	1.10	0.73	0.47	1.18
Vomiting	20	2426	1002	102369	1.11	0.67	1.71	1.05	0.62	1.77
Acute renal failure	<5	2547	157	107105	0.56	0.09	1.82	0.55	0.13	2.37
Chronic kidney disease	7	2534	268	106825	1.16	0.48	2.34	1.10	0.49	2.45
End stage renal disease	0	2558	5	107325	0.36	NA	22.67	NA	NA	NA
Hyperkalemia	6	2546	240	106804	0.77	0.29	1.65	0.74	0.31	1.78
Hypokalemia	87	2523	2271	105879	1.73*	1.35	2.18	1.62*	1.11	2.25
Hypomagnesemia	<5	2554	112	107158	1.62	0.46	4.24	1.52	0.50	4.54
Hyponatremia	11	2550	345	107047	1.42	0.71	2.56	1.34	0.68	2.60
Hypotension	<5	2551	286	106687	0.56	0.14	1.50	0.54	0.17	1.78
Measured renal dysfunction	0	2556	27	107298	0.34	NA	3.46	NA	NA	NA
Anemia	28	2295	1923	95884	0.70	0.46	1.02	0.68	0.43	1.11

© 2020 Hripcsak G. et al. *JAMA Internal Medicine.*

Malignant neoplasm	14	2477	538	104209	1.45	0.79	2.47	1.37	0.74	2.50
Neutropenia or agranulocytosis	<5	2548	119	106816	0.63	0.04	2.99	0.61	0.07	5.32
Thrombocytopenia	<5	2530	166	106600	1.24	0.36	3.13	1.18	0.40	3.42
Anaphylactoid reaction	0	2558	14	107285	0.31	NA	4.23	NA	NA	NA
Angioedema	<5	2558	54	107210	0.97	0.05	4.84	0.92	0.10	8.32
Cough	60	2076	3062	88565	0.94	0.71	1.23	0.90	0.62	1.33
Gout	8	2527	476	105852	0.75	0.33	1.49	0.72	0.33	1.58
Rash	30	2334	1659	99292	0.79	0.52	1.15	0.76	0.48	1.23
Rhabdomyolysis	<5	2555	22	107286	2.71	0.15	14.31	2.52	0.27	23.41
Vasculitis	0	2558	7	107187	0.35	NA	23.87	NA	NA	NA
Venous thromboembolic events	10	2515	357	105710	1.29	0.62	2.40	1.22	0.60	2.45

A "<" in front of a number implies a count less than 5.

* Statistically significant after Bonferroni correction for 55 hypotheses.

NA implies hazard ratio or bound could not be estimated

© 2020 Hripcsak G. et al. *JAMA Internal Medicine.*

2.2 Baseline characteristics for each database for stratification and matching

eTable 3. Baseline characteristics for CCAE with stratification

Characteristic	Before stratification			After stratification		
	Chlor-thalidone (%)*	Hydro-chloro-thiazide (%)*	Std. diff	Chlor-thalidone (%)*	Hydro-chloro-thiazide (%)*	Std. diff
Age group						
05-09	0.0	0.0	0.00	0.0	0.0	0.00
10-14	0.0	0.1	-0.01	0.0	0.1	-0.02
15-19	0.4	0.6	-0.03	0.4	0.6	-0.03
20-24	1.6	1.6	0.00	1.4	1.6	-0.02
25-29	3.0	3.6	-0.03	3.4	3.5	-0.01
30-34	6.0	6.6	-0.02	6.0	6.6	-0.02
35-39	8.9	9.9	-0.03	9.5	9.9	-0.01
40-44	13.1	13.4	-0.01	13.2	13.4	0.00
45-49	15.5	16.4	-0.02	16.0	16.4	-0.01
50-54	18.4	17.6	0.02	18.1	17.7	0.01
55-59	17.2	16.0	0.03	17.0	16.1	0.02
60-64	14.5	13.1	0.04	13.8	13.1	0.02
65-69	1.3	1.1	0.02	1.1	1.1	0.00
Gender: female	51.8	61.1	-0.19	60.9	60.6	0.00
Medical history: General						
Acute respiratory disease	23.4	26.3	-0.07	25.8	26.1	-0.01
Attention deficit hyperactivity disorder	1.5	1.1	0.04	1.0	1.1	-0.01
Chronic liver disease	1.1	1.1	0.00	1.0	1.1	-0.01
Chronic obstructive lung disease	1.2	1.4	-0.02	1.4	1.4	0.00
Crohn's disease	0.3	0.3	0.01	0.3	0.3	0.00
Dementia	0.1	0.1	-0.02	0.1	0.1	-0.01
Depressive disorder	8.3	8.1	0.00	7.7	8.1	-0.02
Diabetes mellitus	4.5	4.6	0.00	4.7	4.5	0.01
Gastroesophageal reflux disease	8.1	7.5	0.02	7.2	7.5	-0.01
Gastrointestinal hemorrhage	1.5	1.6	-0.01	1.6	1.6	0.00
Human immunodeficiency virus infection	0.3	0.3	0.01	0.2	0.3	-0.02
Hyperlipidemia	27.0	25.3	0.04	26.5	25.3	0.03
Hypertensive disorder	100.0	100.0	0.00	100.0	100.0	0.00
Lesion of liver	0.2	0.2	0.00	0.1	0.2	-0.01
Obesity	13.7	9.9	0.12	9.8	10.0	-0.01
Osteoarthritis	11.3	10.6	0.02	10.8	10.6	0.00
Pneumonia	1.4	1.4	-0.01	1.6	1.4	0.02
Psoriasis	1.0	0.9	0.02	0.7	0.9	-0.02
Renal impairment	1.0	0.5	0.06	0.6	0.5	0.02
Rheumatoid arthritis	0.9	0.8	0.01	1.1	0.8	0.03
Schizophrenia	0.1	0.1	0.00	0.0	0.1	-0.01
Ulcerative colitis	0.3	0.2	0.01	0.2	0.2	-0.01
Urinary tract infectious disease	5.3	6.4	-0.04	6.3	6.3	0.00
Viral hepatitis C	0.3	0.3	0.00	0.3	0.3	-0.01
Visual system disorder	15.1	14.9	0.00	14.6	14.8	-0.01
Medical history: Cardiovascular disease						
Atrial fibrillation	0.3	0.2	0.02	0.2	0.2	0.00
Cerebrovascular disease	1.0	1.0	0.00	0.8	0.8	0.01
Coronary arteriosclerosis	1.1	1.0	0.02	1.1	0.9	0.02
Heart disease	7.2	6.4	0.03	6.4	6.1	0.01
Heart failure	0.4	0.3	0.01	0.2	0.2	-0.01
Ischemic heart disease	0.8	0.9	-0.01	0.8	0.8	0.00
Peripheral vascular disease	3.9	3.2	0.04	3.1	3.0	0.00
Pulmonary embolism	0.2	0.2	0.00	0.1	0.2	-0.01
Venous thrombosis	1.0	1.0	0.00	0.9	0.9	0.00
Medical history: Neoplasms						
Hematologic neoplasm	0.5	0.4	0.01	0.4	0.4	-0.01
Malignant lymphoma	0.2	0.2	0.02	0.3	0.2	0.02
Malignant neoplasm of anorectum	0.1	0.1	-0.01	0.1	0.1	0.00

© 2020 Hripcsak G. et al. *JAMA Internal Medicine.*

Malignant neoplastic disease	3.9	3.8	0.01	4.0	3.8	0.01
Malignant tumor of breast	0.9	1.0	-0.01	1.2	1.0	0.02
Malignant tumor of colon	0.1	0.2	-0.01	0.1	0.2	-0.02
Malignant tumor of lung	0.1	0.1	-0.01	0.0	0.1	-0.02
Malignant tumor of urinary bladder	0.1	0.1	0.00	0.1	0.1	0.01
Primary malignant neoplasm of prostate	0.4	0.3	0.00	0.4	0.3	0.00
Medication use						
Antibacterials for systemic use	46.1	50.9	-0.10	50.9	50.6	0.00
Antidepressants	17.7	19.2	-0.04	18.8	19.1	-0.01
Antiepileptics	6.1	6.0	0.00	6.1	5.9	0.01
Antiinflammatory and antirheumatic products	24.6	26.4	-0.04	26.4	26.2	0.00
Antineoplastic agents	1.5	1.5	0.01	1.7	1.5	0.02
Antipsoriatics	0.4	0.4	0.00	0.4	0.4	0.00
Antithrombotic agents	2.7	2.2	0.03	2.4	2.0	0.02
Beta blocking agents	0.5	0.4	0.01	0.4	0.4	-0.01
Calcium channel blockers	0.0	0.0	0.01	0.0	0.0	
Diuretics	0.0	0.0	0.01	0.0	0.0	0.00
Drugs for acid related disorders	13.4	14.0	-0.02	13.9	13.9	0.00
Drugs for obstructive airway diseases	21.0	20.3	0.02	20.6	20.3	0.01
Drugs used in diabetes	2.9	3.1	-0.01	3.2	3.0	0.01
Immunosuppressants	1.7	1.5	0.02	1.4	1.5	-0.01
Lipid modifying agents	14.2	13.5	0.02	14.1	13.4	0.02
Opioids	15.6	16.0	-0.01	16.0	15.8	0.00
Psycholeptics	18.3	18.2	0.00	18.6	18.1	0.01
Psychostimulants, agents used for adhd and nootropics	3.1	3.1	0.00	2.8	3.1	-0.02

© 2020 Hripcsak G. et al. *JAMA Internal Medicine.*

eTable 4. Baseline characteristics for Optum with stratification

Characteristic	Before stratification			After stratification		
	Chlor-thalidone (%)*	Hydro-chloro-thiazide (%)*	Std. diff	Chlor-thalidone (%)*	Hydro-chloro-thiazide (%)*	Std. diff
Age group						
10-14	0.0	0.0	-0.01	0.0	0.0	-0.02
15-19	0.2	0.4	-0.03	0.2	0.4	-0.04
20-24	0.7	1.1	-0.04	1.0	1.1	-0.01
25-29	2.3	2.9	-0.03	2.6	2.9	-0.02
30-34	5.4	5.5	0.00	4.8	5.6	-0.04
35-39	7.1	8.3	-0.04	7.4	8.3	-0.04
40-44	10.5	11.2	-0.02	11.1	11.2	-0.01
45-49	12.2	13.9	-0.05	12.6	13.9	-0.04
50-54	13.1	14.2	-0.03	13.2	14.3	-0.03
55-59	13.0	12.4	0.02	13.1	12.4	0.02
60-64	10.0	9.3	0.02	10.7	9.3	0.04
65-69	9.5	7.3	0.08	8.0	7.3	0.03
70-74	7.1	5.9	0.05	7.0	5.8	0.05
75-79	4.8	4.4	0.02	5.0	4.3	0.03
80-84	3.1	2.5	0.04	2.9	2.4	0.04
85-89	0.8	0.8	0.00	0.5	0.8	-0.03
Gender: female	51.0	60.2	-0.18	60.5	59.9	0.01
Medical history: General						
Acute respiratory disease	22.5	27.3	-0.11	26.7	27.1	-0.01
Attention deficit hyperactivity disorder	1.1	0.9	0.02	0.8	0.9	-0.01
Chronic liver disease	1.7	1.4	0.03	1.4	1.4	0.00
Chronic obstructive lung disease	3.7	3.7	0.00	3.2	3.4	-0.01
Crohn's disease	0.3	0.3	-0.01	0.3	0.3	-0.01
Dementia	0.6	0.7	-0.02	0.6	0.6	-0.01
Depressive disorder	9.7	10.6	-0.03	9.7	10.4	-0.02
Diabetes mellitus	8.2	7.8	0.02	8.5	7.6	0.03
Gastroesophageal reflux disease	11.0	10.7	0.01	10.6	10.6	0.00
Gastrointestinal hemorrhage	2.0	2.3	-0.02	2.1	2.2	-0.01
Human immunodeficiency virus infection	0.3	0.3	0.00	0.5	0.3	0.03
Hyperlipidemia	38.1	36.8	0.03	39.1	36.5	0.05
Hypertensive disorder	100.0	100.0	0.00	100.0	100.0	0.00
Lesion of liver	0.3	0.3	0.00	0.2	0.3	-0.02
Obesity	14.6	11.8	0.08	11.0	11.9	-0.03
Osteoarthritis	16.4	15.6	0.02	15.4	15.4	0.00
Pneumonia	1.8	2.0	-0.02	1.8	1.9	0.00
Psoriasis	1.2	1.0	0.01	1.1	1.1	0.00
Renal impairment	2.4	1.4	0.08	1.3	1.3	0.01
Rheumatoid arthritis	1.4	1.2	0.01	1.5	1.2	0.03
Schizophrenia	0.1	0.2	-0.01	0.2	0.2	0.01
Ulcerative colitis	0.4	0.3	0.01	0.4	0.3	0.01
Urinary tract infectious disease	6.9	7.9	-0.04	8.0	7.7	0.01
Viral hepatitis C	0.6	0.5	0.01	0.4	0.5	-0.01
Visual system disorder	25.3	24.0	0.03	26.4	23.8	0.06
Medical history: Cardiovascular disease						
Atrial fibrillation	1.2	0.9	0.03	1.3	0.8	0.05
Cerebrovascular disease	2.0	2.2	-0.01	1.5	1.6	-0.01
Coronary arteriosclerosis	2.8	2.6	0.01	2.4	2.3	0.01
Heart disease	11.9	11.2	0.02	11.8	10.4	0.04
Heart failure	0.9	0.9	0.00	0.5	0.5	-0.01
Ischemic heart disease	2.1	1.9	0.02	1.8	1.6	0.02
Peripheral vascular disease	7.3	6.1	0.05	6.5	5.7	0.03
Pulmonary embolism	0.3	0.3	-0.01	0.2	0.3	-0.02
Venous thrombosis	1.4	1.5	-0.01	1.4	1.4	0.00
Medical history: Neoplasms						
Hematologic neoplasm	0.6	0.7	-0.01	0.6	0.7	0.00
Malignant lymphoma	0.3	0.3	0.00	0.3	0.2	0.01
Malignant neoplasm of anorectum	0.2	0.2	0.00	0.2	0.2	0.01

© 2020 Hripcsak G. et al. *JAMA Internal Medicine.*

Malignant neoplastic disease	6.4	6.1	0.01	6.5	6.0	0.02
Malignant tumor of breast	1.2	1.3	-0.02	1.2	1.3	-0.01
Malignant tumor of colon	0.2	0.3	-0.03	0.3	0.3	-0.01
Malignant tumor of lung	0.1	0.2	-0.01	0.2	0.2	0.00
Malignant tumor of urinary bladder	0.2	0.2	0.01	0.3	0.2	0.02
Primary malignant neoplasm of prostate	1.0	0.8	0.02	1.0	0.8	0.03
Medication use						
Antibacterials for systemic use	42.4	46.7	-0.09	46.4	46.5	0.00
Antidepressants	16.4	17.8	-0.04	17.7	17.6	0.00
Antiepileptics	7.3	6.0	0.05	6.5	5.9	0.02
Antiinflammatory and antirheumatic products	21.1	22.4	-0.03	22.9	22.4	0.01
Antineoplastic agents	1.8	1.6	0.01	1.7	1.6	0.01
Antipsoriatics	0.3	0.4	-0.01	0.4	0.4	0.00
Antithrombotic agents	3.1	2.8	0.02	2.9	2.4	0.03
Beta blocking agents	0.9	0.9	0.00	1.0	0.8	0.01
Diuretics	-0.1	0.0	0.02	-0.1	0.0	0.00
Drugs for acid related disorders	12.0	12.6	-0.02	12.8	12.5	0.01
Drugs for obstructive airway diseases	18.3	17.6	0.02	17.7	17.6	0.00
Drugs used in diabetes	4.5	4.1	0.02	5.1	4.1	0.05
Immunosuppressants	1.8	1.4	0.03	1.9	1.4	0.03
Lipid modifying agents	18.2	16.6	0.04	17.1	16.4	0.02
Opioids	14.9	14.7	0.01	14.5	14.5	0.00
Psycholeptics	15.2	16.3	-0.03	16.7	16.1	0.02
Psychostimulants, agents used for adhd and nootropics	2.5	2.3	0.01	2.4	2.3	0.01

© 2020 Hripcsak G. et al. *JAMA Internal Medicine.*

eTable 5. Baseline characteristics for PanTher with stratification

Characteristic	Before stratification			After stratification		
	Chlor-thalidone (%)*	Hydro-chloro-thiazide (%)*	Std. diff	Chlor-thalidone (%)*	Hydro-chloro-thiazide (%)*	Std. diff
Age group						
10-14	0.0	0.1	-0.02	0.0	0.1	-0.03
15-19	0.3	0.4	-0.02	0.3	0.4	-0.02
20-24	1.3	1.3	-0.01	1.2	1.4	-0.02
25-29	3.0	3.0	0.00	3.0	3.0	0.00
30-34	5.0	5.2	-0.01	5.0	5.2	-0.01
35-39	7.3	7.2	0.00	7.1	7.3	0.00
40-44	9.6	9.7	0.00	9.1	9.7	-0.02
45-49	12.5	11.9	0.02	12.3	12.0	0.01
50-54	13.7	13.8	0.00	13.0	13.8	-0.02
55-59	13.6	13.1	0.01	13.4	13.2	0.01
60-64	11.5	10.8	0.02	11.1	10.8	0.01
65-69	8.7	8.2	0.02	8.5	8.3	0.01
70-74	5.6	5.8	-0.01	6.1	5.8	0.02
75-79	3.7	4.7	-0.05	5.0	4.6	0.02
80-84	3.2	3.8	-0.03	4.0	3.7	0.02
85-89	0.9	0.9	0.00	0.8	0.9	-0.01
Gender: female	56.4	65.0	-0.18	65.5	64.4	0.02
Race						
race = Asian	1.5	1.5	0.00	1.5	1.5	0.00
race = Black or African American	19.6	19.8	0.00	19.2	19.7	-0.01
race = White	72.0	71.9	0.00	72.9	71.9	0.02
Ethnicity						
ethnicity = Hispanic or Latino	4.2	5.2	-0.05	5.1	5.2	0.00
Medical history: General						
Acute respiratory disease	14.6	14.6	0.00	14.7	14.6	0.00
Attention deficit hyperactivity disorder	1.2	1.0	0.02	1.0	1.0	0.00
Chronic liver disease	1.4	1.2	0.02	1.2	1.2	0.00
Chronic obstructive lung disease	3.0	3.2	-0.01	3.3	3.1	0.01
Crohn's disease	0.3	0.3	0.00	0.3	0.3	0.00
Dementia	0.7	0.8	-0.01	0.8	0.7	0.01
Depressive disorder	10.6	10.8	0.00	10.8	10.7	0.00
Diabetes mellitus	6.5	7.5	-0.04	7.5	7.4	0.00
Gastroesophageal reflux disease	11.2	11.5	-0.01	11.4	11.5	0.00
Gastrointestinal hemorrhage	1.2	1.3	-0.01	1.2	1.3	0.00
Human immunodeficiency virus infection	0.4	0.3	0.01	0.3	0.3	0.00
Hyperlipidemia	30.5	31.1	-0.01	30.7	31.0	-0.01
Hypertensive disorder	95.2	95.4	-0.01	95.0	95.4	-0.02
Lesion of liver	0.3	0.3	-0.01	0.3	0.3	-0.01
Obesity	17.2	14.0	0.09	13.9	14.2	-0.01
Osteoarthritis	11.1	11.2	0.00	11.4	11.1	0.01
Pneumonia	1.5	1.6	-0.01	1.8	1.5	0.02
Psoriasis	0.9	0.8	0.01	0.8	0.8	-0.01
Renal impairment	2.1	1.5	0.04	1.8	1.5	0.02
Rheumatoid arthritis	1.0	1.0	0.00	1.0	1.0	0.00
Schizophrenia	0.3	0.4	-0.02	0.3	0.4	-0.02
Ulcerative colitis	0.3	0.2	0.01	0.2	0.2	0.00
Urinary tract infectious disease	3.4	4.3	-0.05	4.1	4.2	0.00
Viral hepatitis C	0.7	0.7	0.01	0.7	0.7	0.00
Visual system disorder	10.9	9.5	0.05	9.6	9.5	0.00
Medical history: Cardiovascular disease						
Atrial fibrillation	1.0	1.0	0.00	0.9	1.0	0.00
Cerebrovascular disease	1.1	1.1	0.00	1.0	1.0	0.00
Coronary arteriosclerosis	2.2	2.1	0.01	2.1	2.0	0.01
Heart disease	8.6	8.1	0.02	8.3	7.9	0.02
Heart failure	0.5	0.5	0.00	0.4	0.4	0.00
Ischemic heart disease	1.0	1.0	0.01	1.1	0.9	0.03
Peripheral vascular disease	4.7	4.2	0.03	4.3	4.1	0.01

© 2020 Hripcsak G. et al. *JAMA Internal Medicine.*

Pulmonary embolism	0.3	0.3	-0.01	0.2	0.3	-0.02
Venous thrombosis	0.9	0.9	0.00	0.8	0.9	-0.01
Medical history: Neoplasms						
Hematologic neoplasm	0.6	0.5	0.01	0.5	0.5	0.01
Malignant lymphoma	0.3	0.3	0.01	0.3	0.3	0.01
Malignant neoplasm of anorectum	0.1	0.1	0.00	0.2	0.1	0.01
Malignant neoplastic disease	5.5	5.1	0.01	5.5	5.1	0.02
Malignant tumor of breast	1.0	1.2	-0.02	1.3	1.2	0.01
Malignant tumor of colon	0.2	0.3	-0.01	0.3	0.3	0.00
Malignant tumor of lung	0.1	0.1	0.00	0.1	0.1	0.00
Malignant tumor of urinary bladder	0.1	0.1	0.01	0.2	0.1	0.01
Primary malignant neoplasm of prostate	0.6	0.5	0.01	0.6	0.5	0.00
Medication use						
Agents acting on the renin-angiotensin system	0.0	0.0	0.00	0.0	0.0	0.01
Antibacterials for systemic use	27.8	28.0	0.00	28.7	28.0	0.02
Antidepressants	20.1	21.0	-0.02	21.6	20.9	0.02
Antiepileptics	8.4	8.7	-0.01	9.0	8.6	0.01
Antiinflammatory and antirheumatic products	30.3	29.9	0.01	30.8	30.0	0.02
Antineoplastic agents	1.4	1.3	0.01	1.4	1.3	0.01
Antipsoriatics	0.2	0.2	0.01	0.2	0.2	0.00
Antithrombotic agents	17.0	16.7	0.01	17.7	16.6	0.03
Beta blocking agents	0.6	0.7	-0.01	0.6	0.7	-0.01
Calcium channel blockers	0.0	0.0	0.01	0.0	0.0	
Diuretics	0.1	0.0	0.03	0.1	0.0	0.03
Drugs for acid related disorders	21.2	22.1	-0.02	22.9	22.0	0.02
Drugs for obstructive airway diseases	27.9	27.1	0.02	27.7	27.2	0.01
Drugs used in diabetes	5.0	6.0	-0.04	6.2	5.9	0.01
Immunosuppressants	1.5	1.4	0.00	1.3	1.4	-0.01
Lipid modifying agents	18.9	19.2	-0.01	19.6	19.1	0.01
Opioids	16.6	17.9	-0.04	18.5	17.8	0.02
Psycholeptics	19.6	20.7	-0.03	21.4	20.5	0.02
Psychostimulants, agents used for adhd and nootropics	3.0	2.9	0.00	3.1	2.9	0.01

© 2020 Hripcsak G. et al. *JAMA Internal Medicine.*

eTable 6. Baseline characteristics for CCAE with matching

Characteristic	Before matching			After matching		
	Chlor-thalidone (%)*	Hydro-chloro-thiazide (%)*	Std. diff	Chlor-thalidone (%)*	Hydro-chloro-thiazide (%)*	Std. diff
Age group						
05-09	0.0	0.0	0.00	0.0	0.0	0.00
10-14	0.0	0.1	-0.01	0.0	0.1	-0.02
15-19	0.4	0.6	-0.03	0.4	0.6	-0.03
20-24	1.6	1.6	0.00	1.6	1.7	-0.01
25-29	3.0	3.6	-0.03	3.0	3.0	0.00
30-34	6.0	6.6	-0.02	6.0	6.2	-0.01
35-39	8.9	9.9	-0.03	9.0	9.1	0.00
40-44	13.1	13.4	-0.01	13.1	13.0	0.00
45-49	15.5	16.4	-0.02	15.6	15.4	0.00
50-54	18.4	17.6	0.02	18.4	18.3	0.00
55-59	17.2	16.0	0.03	17.2	17.0	0.00
60-64	14.5	13.1	0.04	14.4	14.4	0.00
65-69	1.3	1.1	0.02	1.3	1.1	0.02
Gender: female	51.8	61.1	-0.19	51.9	51.3	0.01
Medical history: General						
Acute respiratory disease	23.4	26.3	-0.07	23.3	23.1	0.00
Attention deficit hyperactivity disorder	1.5	1.1	0.04	1.4	1.4	0.00
Chronic liver disease	1.1	1.1	0.00	1.1	1.2	0.00
Chronic obstructive lung disease	1.2	1.4	-0.02	1.2	1.3	-0.01
Crohn's disease	0.3	0.3	0.01	0.3	0.3	0.00
Dementia	0.1	0.1	-0.02	0.1	0.1	-0.01
Depressive disorder	8.3	8.1	0.00	8.2	8.4	-0.01
Diabetes mellitus	4.5	4.6	0.00	4.4	4.6	0.00
Gastroesophageal reflux disease	8.1	7.5	0.02	8.0	8.2	-0.01
Gastrointestinal hemorrhage	1.5	1.6	-0.01	1.5	1.5	0.00
Human immunodeficiency virus infection	0.3	0.3	0.01	0.3	0.4	-0.02
Hyperlipidemia	27.0	25.3	0.04	26.9	27.2	-0.01
Hypertensive disorder	100.0	100.0	0.00	100.0	100.0	0.00
Lesion of liver	0.2	0.2	0.00	0.2	0.2	-0.01
Obesity	13.7	9.9	0.12	13.7	14.0	-0.01
Osteoarthritis	11.3	10.6	0.02	11.2	11.4	-0.01
Pneumonia	1.4	1.4	-0.01	1.3	1.2	0.01
Psoriasis	1.0	0.9	0.02	1.0	1.1	-0.01
Renal impairment	1.0	0.5	0.06	0.9	0.8	0.01
Rheumatoid arthritis	0.9	0.8	0.01	0.9	0.9	0.00
Schizophrenia	0.1	0.1	0.00	0.0	0.1	0.00
Ulcerative colitis	0.3	0.2	0.01	0.3	0.3	0.00
Urinary tract infectious disease	5.3	6.4	-0.04	5.2	5.2	0.00
Viral hepatitis C	0.3	0.3	0.00	0.3	0.3	0.00
Visual system disorder	15.1	14.9	0.00	15.0	14.8	0.00
Medical history: Cardiovascular disease						
Atrial fibrillation	0.3	0.2	0.02	0.3	0.3	0.01
Cerebrovascular disease	1.0	1.0	0.00	0.8	0.7	0.02
Coronary arteriosclerosis	1.1	1.0	0.02	1.0	1.1	0.00
Heart disease	7.2	6.4	0.03	6.9	6.9	0.00
Heart failure	0.4	0.3	0.01	0.2	0.2	0.01
Ischemic heart disease	0.8	0.9	-0.01	0.7	0.8	-0.01
Peripheral vascular disease	3.9	3.2	0.04	3.6	3.6	0.00
Pulmonary embolism	0.2	0.2	0.00	0.2	0.2	-0.01
Venous thrombosis	1.0	1.0	0.00	0.9	1.0	-0.01
Medical history: Neoplasms						
Hematologic neoplasm	0.5	0.4	0.01	0.5	0.5	0.00
Malignant lymphoma	0.2	0.2	0.02	0.2	0.2	0.00
Malignant neoplasm of anorectum	0.1	0.1	-0.01	0.1	0.1	0.00
Malignant neoplastic disease	3.9	3.8	0.01	3.9	4.0	-0.01
Malignant tumor of breast	0.9	1.0	-0.01	0.9	0.9	0.00
Malignant tumor of colon	0.1	0.2	-0.01	0.1	0.2	0.00

© 2020 Hripcsak G. et al. *JAMA Internal Medicine.*

Malignant tumor of lung	0.1	0.1	-0.01	0.1	0.1	-0.01
Malignant tumor of urinary bladder	0.1	0.1	0.00	0.1	0.1	0.00
Primary malignant neoplasm of prostate	0.4	0.3	0.00	0.4	0.4	0.00
Medication use						
Antibacterials for systemic use	46.1	50.9	-0.10	46.1	45.7	0.01
Antidepressants	17.7	19.2	-0.04	17.7	18.1	-0.01
Antiepileptics	6.1	6.0	0.00	5.9	6.1	0.00
Antiinflammatory and antirheumatic products	24.6	26.4	-0.04	24.7	24.5	0.00
Antineoplastic agents	1.5	1.5	0.01	1.5	1.6	-0.01
Antipsoriatics	0.4	0.4	0.00	0.4	0.4	0.01
Antithrombotic agents	2.7	2.2	0.03	2.5	2.4	0.00
Beta blocking agents	0.5	0.4	0.01	0.5	0.4	0.01
Diuretics	0.0	0.0	0.01	0.0	0.0	0.02
Drugs for acid related disorders	13.4	14.0	-0.02	13.3	13.3	0.00
Drugs for obstructive airway diseases	21.0	20.3	0.02	21.0	21.2	0.00
Drugs used in diabetes	2.9	3.1	-0.01	2.8	2.9	-0.01
Immunosuppressants	1.7	1.5	0.02	1.7	1.7	0.00
Lipid modifying agents	14.2	13.5	0.02	14.0	14.1	0.00
Opioids	15.6	16.0	-0.01	15.4	15.5	0.00
Psycholeptics	18.3	18.2	0.00	18.2	18.3	0.00
Psychostimulants, agents used for adhd and nootropics	3.1	3.1	0.00	3.1	3.1	0.

© 2020 Hripcsak G. et al. *JAMA Internal Medicine.*

eTable 7. Baseline characteristics for Optum with matching

Characteristic	Before matching			After matching		
	Chlor-thalidone (%)*	Hydro-chloro-thiazide (%)*	Std. diff	Chlor-thalidone (%)*	Hydro-chloro-thiazide (%)*	Std. diff
Age group						
10-14	0.0	0.0	-0.01	0.0	0.0	-0.01
15-19	0.2	0.4	-0.03	0.2	0.4	-0.04
20-24	0.7	1.1	-0.04	0.7	0.9	-0.02
25-29	2.3	2.9	-0.03	2.3	2.5	-0.01
30-34	5.4	5.5	0.00	5.5	5.2	0.01
35-39	7.1	8.3	-0.04	7.2	7.4	-0.01
40-44	10.5	11.2	-0.02	10.7	10.3	0.01
45-49	12.2	13.9	-0.05	12.3	12.4	0.00
50-54	13.1	14.2	-0.03	13.1	13.5	-0.01
55-59	13.0	12.4	0.02	13.0	12.8	0.00
60-64	10.0	9.3	0.02	10.1	9.9	0.01
65-69	9.5	7.3	0.08	9.5	9.5	0.00
70-74	7.1	5.9	0.05	7.0	6.8	0.01
75-79	4.8	4.4	0.02	4.7	4.5	0.01
80-84	3.1	2.5	0.04	3.0	2.7	0.01
85-89	0.8	0.8	0.00	0.7	1.0	-0.03
Gender: female	51.0	60.2	-0.18	51.1	50.3	0.02
Medical history: General						
Acute respiratory disease	22.5	27.3	-0.11	22.3	22.2	0.00
Attention deficit hyperactivity disorder	1.1	0.9	0.02	1.1	1.2	-0.01
Chronic liver disease	1.7	1.4	0.03	1.7	1.5	0.02
Chronic obstructive lung disease	3.7	3.7	0.00	3.5	3.6	-0.01
Crohn's disease	0.3	0.3	-0.01	0.3	0.3	0.00
Dementia	0.6	0.7	-0.02	0.5	0.7	-0.02
Depressive disorder	9.7	10.6	-0.03	9.6	9.6	0.00
Diabetes mellitus	8.2	7.8	0.02	8.0	8.2	-0.01
Gastroesophageal reflux disease	11.0	10.7	0.01	11.0	11.1	0.00
Gastrointestinal hemorrhage	2.0	2.3	-0.02	2.0	1.9	0.00
Human immunodeficiency virus infection	0.3	0.3	0.00	0.3	0.4	-0.01
Hyperlipidemia	38.1	36.8	0.03	37.7	37.5	0.00
Hypertensive disorder	100.0	100.0	0.00	100.0	100.0	0.00
Lesion of liver	0.3	0.3	0.00	0.3	0.3	-0.01
Obesity	14.6	11.8	0.08	14.7	14.4	0.01
Osteoarthritis	16.4	15.6	0.02	16.2	16.3	0.00
Pneumonia	1.8	2.0	-0.02	1.7	1.7	0.00
Psoriasis	1.2	1.0	0.01	1.2	1.2	0.00
Renal impairment	2.4	1.4	0.08	2.2	2.2	0.00
Rheumatoid arthritis	1.4	1.2	0.01	1.4	1.3	0.01
Schizophrenia	0.1	0.2	-0.01	0.1	0.2	-0.01
Ulcerative colitis	0.4	0.3	0.01	0.3	0.3	0.00
Urinary tract infectious disease	6.9	7.9	-0.04	6.8	6.7	0.00
Viral hepatitis C	0.6	0.5	0.01	0.6	0.6	0.00
Visual system disorder	25.3	24.0	0.03	25.2	24.9	0.01
Medical history: Cardiovascular disease						
Atrial fibrillation	1.2	0.9	0.03	1.1	0.9	0.02
Cerebrovascular disease	2.0	2.2	-0.01	1.4	1.4	0.00
Coronary arteriosclerosis	2.8	2.6	0.01	2.5	2.5	0.00
Heart disease	11.9	11.2	0.02	11.2	10.9	0.01
Heart failure	0.9	0.9	0.00	0.6	0.6	0.00
Ischemic heart disease	2.1	1.9	0.02	1.8	1.6	0.01
Peripheral vascular disease	7.3	6.1	0.05	6.7	6.6	0.01
Pulmonary embolism	0.3	0.3	-0.01	0.3	0.3	-0.01
Venous thrombosis	1.4	1.5	-0.01	1.3	1.4	-0.01
Medical history: Neoplasms						
Hematologic neoplasm	0.6	0.7	-0.01	0.6	0.7	-0.01
Malignant lymphoma	0.3	0.3	0.00	0.3	0.3	0.00
Malignant neoplasm of anorectum	0.2	0.2	0.00	0.2	0.2	0.00

© 2020 Hripcsak G. et al. *JAMA Internal Medicine.*

Malignant neoplastic disease	6.4	6.1	0.01	6.3	6.5	-0.01
Malignant tumor of breast	1.2	1.3	-0.02	1.2	1.1	0.00
Malignant tumor of colon	0.2	0.3	-0.03	0.2	0.3	-0.02
Malignant tumor of lung	0.1	0.2	-0.01	0.1	0.2	-0.01
Malignant tumor of urinary bladder	0.2	0.2	0.01	0.2	0.2	0.00
Primary malignant neoplasm of prostate	1.0	0.8	0.02	1.0	0.9	0.01
Medication use						
Antibacterials for systemic use	42.4	46.7	-0.09	42.3	42.4	0.00
Antidepressants	16.4	17.8	-0.04	16.4	16.4	0.00
Antiepileptics	7.3	6.0	0.05	7.3	7.1	0.01
Antiinflammatory and antirheumatic products	21.1	22.4	-0.03	21.2	21.1	0.00
Antineoplastic agents	1.8	1.6	0.01	1.7	1.8	-0.01
Antipsoriatics	0.3	0.4	-0.01	0.3	0.3	0.00
Antithrombotic agents	3.1	2.8	0.02	2.7	2.7	0.00
Beta blocking agents	0.9	0.9	0.00	0.9	1.0	-0.01
Diuretics	-0.1	0.0	0.02	-0.1	0.0	0.02
Drugs for acid related disorders	12.0	12.6	-0.02	11.9	12.1	-0.01
Drugs for obstructive airway diseases	18.3	17.6	0.02	18.3	18.4	0.00
Drugs used in diabetes	4.5	4.1	0.02	4.4	4.4	0.00
Immunosuppressants	1.8	1.4	0.03	1.8	1.7	0.01
Lipid modifying agents	18.2	16.6	0.04	17.8	18.3	-0.01
Opioids	14.9	14.7	0.01	14.8	14.6	0.00
Psycholeptics	15.2	16.3	-0.03	15.0	14.9	0.00
Psychostimulants, agents used for adhd and nootropics	2.5	2.3	0.01	2.4	2.3	0.01

© 2020 Hripcsak G. et al. *JAMA Internal Medicine.*

eTable 8. Baseline characteristics for PanTher with matching

Characteristic	Before matching			After matching		
	Chlor-thalidone (%)*	Hydro-chloro-thiazide (%)*	Std. diff	Chlor-thalidone (%)*	Hydro-chloro-thiazide (%)*	Std. diff
Age group						
10-14	0.0	0.1	-0.02	0.0	0.1	-0.03
15-19	0.3	0.4	-0.02	0.3	0.4	-0.02
20-24	1.3	1.3	-0.01	1.2	1.4	-0.01
25-29	3.0	3.0	0.00	3.0	3.0	0.00
30-34	5.0	5.2	-0.01	5.0	5.1	0.00
35-39	7.3	7.2	0.00	7.4	7.3	0.00
40-44	9.6	9.7	0.00	9.7	9.8	0.00
45-49	12.5	11.9	0.02	12.6	12.4	0.01
50-54	13.7	13.8	0.00	13.7	13.7	0.00
55-59	13.6	13.1	0.01	13.6	13.4	0.00
60-64	11.5	10.8	0.02	11.5	11.6	0.00
65-69	8.7	8.2	0.02	8.7	8.8	0.00
70-74	5.6	5.8	-0.01	5.6	5.4	0.01
75-79	3.7	4.7	-0.05	3.7	3.6	0.00
80-84	3.2	3.8	-0.03	3.2	3.1	0.01
85-89	0.9	0.9	0.00	0.9	0.9	-0.01
Gender: female	56.4	65.0	-0.18	56.5	55.8	0.02
Race						
race = Asian	1.5	1.5	0.00	1.5	1.7	-0.02
race = Black or African American	19.6	19.8	0.00	19.6	19.4	0.00
race = White	72.0	71.9	0.00	72.0	71.7	0.01
Ethnicity						
ethnicity = Hispanic or Latino	4.2	5.2	-0.05	4.2	4.2	0.00
Medical history: General						
Acute respiratory disease	14.6	14.6	0.00	14.6	14.8	0.00
Attention deficit hyperactivity disorder	1.2	1.0	0.02	1.2	1.2	0.00
Chronic liver disease	1.4	1.2	0.02	1.4	1.4	0.00
Chronic obstructive lung disease	3.0	3.2	-0.01	2.9	3.0	0.00
Crohn's disease	0.3	0.3	0.00	0.3	0.3	-0.01
Dementia	0.7	0.8	-0.01	0.7	0.6	0.00
Depressive disorder	10.6	10.8	0.00	10.6	10.5	0.00
Diabetes mellitus	6.5	7.5	-0.04	6.5	6.3	0.00
Gastroesophageal reflux disease	11.2	11.5	-0.01	11.2	11.3	0.00
Gastrointestinal hemorrhage	1.2	1.3	-0.01	1.1	1.2	-0.01
Human immunodeficiency virus infection	0.4	0.3	0.01	0.4	0.4	0.00
Hyperlipidemia	30.5	31.1	-0.01	30.4	30.4	0.00
Hypertensive disorder	95.2	95.4	-0.01	95.2	95.0	0.01
Lesion of liver	0.3	0.3	-0.01	0.3	0.3	-0.01
Obesity	17.2	14.0	0.09	17.2	17.5	-0.01
Osteoarthritis	11.1	11.2	0.00	11.0	11.0	0.00
Pneumonia	1.5	1.6	-0.01	1.5	1.4	0.00
Psoriasis	0.9	0.8	0.01	0.9	1.0	-0.01
Renal impairment	2.1	1.5	0.04	2.0	2.0	0.00
Rheumatoid arthritis	1.0	1.0	0.00	1.0	1.1	0.00
Schizophrenia	0.3	0.4	-0.02	0.3	0.3	-0.01
Ulcerative colitis	0.3	0.2	0.01	0.3	0.3	0.00
Urinary tract infectious disease	3.4	4.3	-0.05	3.3	3.4	0.00
Viral hepatitis C	0.7	0.7	0.01	0.7	0.7	0.00
Visual system disorder	10.9	9.5	0.05	10.9	10.9	0.00
Medical history: Cardiovascular disease						
Atrial fibrillation	1.0	1.0	0.00	1.0	0.9	0.00
Cerebrovascular disease	1.1	1.1	0.00	1.0	0.9	0.00
Coronary arteriosclerosis	2.2	2.1	0.01	2.1	2.0	0.01
Heart disease	8.6	8.1	0.02	8.4	8.3	0.00
Heart failure	0.5	0.5	0.00	0.4	0.4	0.01
Ischemic heart disease	1.0	1.0	0.01	1.0	0.8	0.01
Peripheral vascular disease	4.7	4.2	0.03	4.6	4.6	0.00

© 2020 Hripcsak G. et al. *JAMA Internal Medicine.*

Pulmonary embolism	0.3	0.3	-0.01	0.3	0.3	-0.02
Venous thrombosis	0.9	0.9	0.00	0.9	0.9	0.00
Medical history: Neoplasms						
Hematologic neoplasm	0.6	0.5	0.01	0.6	0.5	0.01
Malignant lymphoma	0.3	0.3	0.01	0.3	0.3	0.01
Malignant neoplasm of anorectum	0.1	0.1	0.00	0.1	0.2	-0.01
Malignant neoplastic disease	5.5	5.1	0.01	5.4	5.5	0.00
Malignant tumor of breast	1.0	1.2	-0.02	1.0	1.1	-0.01
Malignant tumor of colon	0.2	0.3	-0.01	0.2	0.3	-0.01
Malignant tumor of lung	0.1	0.1	0.00	0.1	0.1	0.00
Malignant tumor of urinary bladder	0.1	0.1	0.01	0.1	0.1	0.00
Primary malignant neoplasm of prostate	0.6	0.5	0.01	0.6	0.6	0.00
Medication use						
Agents acting on the renin-angiotensin system	0.0	0.0	0.00	0.0	0.0	0.01
Antibacterials for systemic use	27.8	28.0	0.00	27.8	27.8	0.00
Antidepressants	20.1	21.0	-0.02	20.1	20.0	0.00
Antiepileptics	8.4	8.7	-0.01	8.4	8.4	0.00
Antiinflammatory and antirheumatic products	30.3	29.9	0.01	30.3	30.3	0.00
Antineoplastic agents	1.4	1.3	0.01	1.4	1.4	0.00
Antipsoriatics	0.2	0.2	0.01	0.2	0.2	0.00
Antithrombotic agents	17.0	16.7	0.01	16.8	16.8	0.00
Beta blocking agents	0.6	0.7	-0.01	0.6	0.7	-0.01
Calcium channel blockers	0.0	0.0	0.01	0.0	0.0	
Diuretics	0.1	0.0	0.03	0.1	0.0	0.03
Drugs for acid related disorders	21.2	22.1	-0.02	21.2	21.0	0.00
Drugs for obstructive airway diseases	27.9	27.1	0.02	27.9	28.1	-0.01
Drugs used in diabetes	5.0	6.0	-0.04	5.0	5.0	0.00
Immunosuppressants	1.5	1.4	0.00	1.4	1.5	-0.01
Lipid modifying agents	18.9	19.2	-0.01	18.8	18.6	0.00
Opioids	16.6	17.9	-0.04	16.6	16.5	0.00
Psycholeptics	19.6	20.7	-0.03	19.5	19.2	0.01
Psychostimulants, agents used for adhd and nootropics	3.0	2.9	0.00	3.0	3.0	0.00

© 2020 Hripcsak G. et al. *JAMA Internal Medicine.*

2.3 Effectiveness and safety results for the meta-analysis for each analytic approach (stratified vs. matching; on-treatment vs. intent-to-treat)

(Only meta-analysis results are shown here. Effectiveness and safety results by individual database are available at http://data.ohdsi.org/LegendBasicViewer/ -> Specific Research Questions -> “Hypertension,” “Drug or procedure,” “Chlorthalidone,” “Hydrochlorothiazide,” <outcome of interest>.)

eTable 9. Effectiveness and safety for meta-analysis for stratified on-treatment (these are the primary results reported in the main paper)

	Chlorthalidone[#]		Hydrochloro-thiazide		Hazard ratio uncalibrated			Calibrated hazard ratio		
Outcome	Events	Subjects	Events	Subjects	HR	Lower CI	Upper CI	HR	Lower CI	Upper CI
Acute myocardial infarction	41	36859	952	692371	0.93	0.63	1.36	0.92	0.64	1.31
All-cause mortality	20	22814	463	405947	0.94	0.59	1.48	0.93	0.61	1.42
Composite cardiovascular disease	149	36628	3089	687106	1.01	0.86	1.20	1.00	0.85	1.17
Cardiovascular-related mortality	<11	22814	137	405947	1.28	0.60	2.73	1.24	0.62	2.51
Chest pain or angina	1211	29825	27487	557506	0.95	0.87	1.05	0.94	0.87	1.03
Sudden cardiac death	<16	36912	252	693210	0.85	0.45	1.60	0.85	0.47	1.52
Hospitalization with preinfarction syndrome	28	36831	940	691433	0.85	0.58	1.27	0.85	0.59	1.23
Bradycardia	104	36522	2067	686221	1.15	0.94	1.41	1.12	0.93	1.35
Cardiac arrhythmia	635	34296	11604	649112	1.09	1.01	1.19	1.07	0.99	1.15
Syncope	347	35881	6096	673888	1.23*	1.09	1.38	1.19	1.07	1.33
Heart failure	78	36800	1516	691182	1.03	0.73	1.46	1.01	0.73	1.40
Hospitalization with heart failure	62	36833	1248	691409	1.07	0.82	1.39	1.05	0.82	1.34
Fall	233	35664	4614	672215	0.97	0.84	1.10	0.95	0.84	1.08
Headache	771	30713	17177	572037	0.97	0.90	1.04	0.95	0.89	1.02
Hemorrhagic stroke	<15	36884	234	692586	0.93	0.37	2.35	0.92	0.39	2.18
Ischemic stroke	53	36778	999	690271	1.11	0.84	1.48	1.09	0.84	1.42
Stroke	60	36755	1141	689698	1.13	0.86	1.47	1.10	0.86	1.41
Transient ischemic attack	<47	36769	868	690106	1.27	0.94	1.73	1.23	0.93	1.64
Vertigo	158	36159	3397	680363	1.02	0.87	1.20	1.01	0.87	1.17
Anxiety	664	32558	13773	617896	0.92	0.85	1.00	0.91	0.85	0.98
Decreased libido	70	36635	931	689381	1.23	0.96	1.58	1.19	0.95	1.51
Dementia	<43	36753	1146	689502	0.72	0.52	1.00	0.73	0.54	0.98
Depression	513	31874	12262	600767	0.92	0.84	1.00	0.91	0.84	0.99
Impotence	369	35446	4751	674492	1.21*	1.09	1.35	1.18	1.07	1.30
Abdominal pain	1091	28496	24660	536678	0.98	0.92	1.04	0.97	0.91	1.02
Abnormal weight gain	140	35460	4303	664489	0.72*	0.60	0.87	0.73*	0.61	0.86
Abnormal weight loss	191	36319	3356	682277	1.17	1.01	1.36	1.14	0.99	1.31
Acute pancreatitis	23	36827	523	691623	1.01	0.65	1.55	0.99	0.66	1.48
Diarrhea	463	34415	9263	650702	1.06	0.97	1.17	1.04	0.95	1.14
Gastrointestinal bleeding	50	36761	975	689923	1.17	0.88	1.57	1.14	0.87	1.50
Hepatic failure	<19	36900	231	692854	1.43	0.59	3.49	1.38	0.60	3.15
Nausea	613	34300	11373	643933	1.12	1.01	1.23	1.09	0.99	1.20
Type 2 diabetes mellitus	725	34535	12002	648258	1.25*	1.15	1.35	1.21*	1.12	1.30
Vomiting	438	35003	7997	657171	1.17	1.06	1.29	1.14	1.04	1.25
Acute renal failure	125	36759	1556	691023	1.42*	1.18	1.72	1.37*	1.15	1.63
Chronic kidney	213	36388	3108	686812	1.28*	1.11	1.48	1.24*	1.09	1.42

© 2020 Hripcsak G. et al. *JAMA Internal Medicine.*

disease										
End stage renal disease	<15	36907	86	693239	1.21	0.45	3.25	1.17	0.47	2.94
Hyperkalemia	150	36664	2049	688845	1.39	1.04	1.84	1.34	1.03	1.74
Hypokalemia	2358	35759	15514	676942	2.99*	2.58	3.46	2.72*	2.38	3.12
Hypomagnesemia	112	36760	1144	691375	1.65	1.20	2.28	1.57	1.16	2.12
Hyponatremia	281	36677	3749	688957	1.36*	1.20	1.53	1.31*	1.16	1.47
Hypotension	180	36639	2952	688351	1.27	0.97	1.68	1.23	0.95	1.60
Measured renal dysfunction	<10	21789	87	477115	1.26	0.56	2.85	1.23	0.58	2.60
Anemia	670	33097	15653	619347	0.92	0.85	0.99	0.91	0.84	0.98
Malignant neoplasm	264	35392	5207	665994	1.00	0.84	1.20	0.99	0.84	1.17
Neutropenia or agranulocytosis	40	36771	987	690275	0.92	0.66	1.27	0.91	0.67	1.23
Thrombocytopenia	90	36577	1617	688395	0.98	0.71	1.34	0.96	0.72	1.29
Anaphylactoid reaction	<15	36899	74	693034	3.26	1.53	6.96	2.96	1.46	5.97
Angioedema	<17	36875	318	692371	0.71	0.37	1.37	0.72	0.39	1.32
Cough	1085	30570	22417	587562	1.00	0.94	1.06	0.98	0.93	1.04
Gout	207	36130	3045	682066	1.31	1.04	1.65	1.27	1.02	1.57
Rash	574	33846	11787	640868	0.94	0.84	1.06	0.93	0.84	1.04
Rhabdomyolysis	<15	36883	160	692902	0.84	0.27	2.57	0.83	0.29	2.37
Vasculitis	<15	36875	139	692816	0.81	0.31	2.12	0.81	0.33	1.98
Venous thromboembolic events	154	36250	3036	681014	1.07	0.90	1.26	1.05	0.90	1.22

A "<" in front of a number implies one of the databases had a count less than 5, so the sum is some number less than the printed number.
* Statistically significant after Bonferroni correction for 55 hypotheses.

© 2020 Hripcsak G. et al. *JAMA Internal Medicine.*

eTable 10. Effectiveness and safety for meta-analysis for stratified intent-to-treat

	Chlorthalidone		Hydrochloro-thiazide		Hazard ratio uncalibrated			Calibrated hazard ratio		
Outcome	Events	Subjects	Events	Subjects	HR	Lower CI	Upper CI	HR	Lower CI	Upper CI
Acute myocardial infarction	275	36885	6293	693725	1.05	0.93	1.18	1.04	0.91	1.18
All-cause mortality	324	36944	8523	694693	1.08	0.96	1.22	1.07	0.95	1.21
Composite cardiovascular disease	892	36654	20824	688446	1.05	0.98	1.13	1.04	0.96	1.13
Cardiovascular-related mortality	79	36944	2241	694693	1.02	0.81	1.28	1.01	0.80	1.27
Chest pain or angina	4429	29844	105920	558591	0.98	0.95	1.01	0.97	0.92	1.02
Sudden cardiac death	58	36938	1795	694566	0.78	0.59	1.01	0.77	0.59	1.01
Hospitalization with preinfarction syndrome	153	36857	4728	692782	0.99	0.84	1.17	0.98	0.83	1.16
Bradycardia	472	36548	10597	687560	1.13	1.03	1.24	1.11	1.01	1.24
Cardiac arrhythmia	2250	34320	48995	650388	1.04	1.00	1.09	1.03	0.98	1.10
Syncope	1336	35904	28944	675215	1.12	1.04	1.21	1.11	1.02	1.21
Heart failure	529	36826	11711	692532	1.06	0.97	1.16	1.05	0.95	1.16
Hospitalization with heart failure	440	36859	10512	692757	1.06	0.97	1.17	1.05	0.95	1.17
Fall	1420	35690	30793	673546	1.02	0.97	1.08	1.01	0.95	1.08
Headache	2968	30735	72670	573143	0.97	0.92	1.02	0.96	0.90	1.03
Hemorrhagic stroke	53	36910	1558	693939	0.96	0.72	1.27	0.95	0.72	1.25
Ischemic stroke	294	36804	6651	691623	1.11	0.99	1.25	1.10	0.97	1.25
Stroke	326	36781	7541	691047	1.10	0.98	1.23	1.09	0.97	1.23
Transient ischemic attack	202	36795	5125	691455	1.08	0.89	1.31	1.07	0.88	1.30
Vertigo	708	36185	15800	681693	1.06	0.99	1.15	1.05	0.97	1.15
Anxiety	2522	32580	56346	619141	0.97	0.93	1.01	0.96	0.91	1.02
Decreased libido	230	36661	4759	690733	0.95	0.83	1.09	0.94	0.82	1.08
Dementia	343	36779	8461	690854	1.02	0.91	1.14	1.01	0.90	1.13
Depression	1854	31894	48829	601976	0.94	0.89	0.98	0.93	0.87	0.99
Impotence	1311	35471	20221	675811	1.13	1.04	1.24	1.12	1.02	1.24
Abdominal pain	4499	28515	107522	537736	0.99	0.95	1.03	0.98	0.93	1.04
Abnormal weight gain	767	35484	20547	665794	0.88	0.77	1.01	0.88	0.76	1.01
Abnormal weight loss	887	36344	19205	683615	1.09	1.01	1.19	1.08	0.99	1.19
Acute pancreatitis	119	36853	2974	692977	1.02	0.85	1.23	1.01	0.84	1.22
Diarrhea	2192	34440	49136	651983	1.05	1.00	1.10	1.04	0.98	1.10
Gastrointestinal bleeding	271	36787	6551	691273	1.07	0.94	1.21	1.06	0.93	1.20
Hepatic failure	69	36926	1653	694210	0.97	0.70	1.35	0.97	0.70	1.34
Nausea	2555	34324	57279	645214	1.05	1.00	1.10	1.04	0.97	1.11
Type 2 diabetes mellitus	2036	34561	41763	649517	1.15*	1.10	1.20	1.14*	1.07	1.21
Vomiting	1808	35027	41914	658466	1.04	0.99	1.09	1.03	0.97	1.10
Acute renal failure	584	36785	12084	692376	1.09	1.00	1.19	1.08	0.98	1.19
Chronic kidney disease	877	36414	16743	688163	1.20*	1.11	1.28	1.18*	1.09	1.29
End stage renal disease	60	36933	1065	694595	1.27	0.97	1.66	1.25	0.96	1.64
Hyperkalemia	584	36689	11336	690194	1.18	1.04	1.34	1.16	1.02	1.33
Hypokalemia	5033	35782	52039	678276	2.28*	1.98	2.62	2.23*	1.92	2.59
Hypomagnesemia	492	36786	7504	692729	1.39*	1.24	1.56	1.38*	1.22	1.56
Hyponatremia	931	36703	17263	690307	1.19	1.05	1.36	1.18	1.03	1.35
Hypotension	845	36664	17644	689696	1.15	1.05	1.27	1.14	1.03	1.27
Measured renal dysfunction	27	21815	627	478442	1.02	0.69	1.51	1.01	0.68	1.49
Anemia	2814	33120	68523	620564	0.98	0.95	1.02	0.98	0.92	1.03
Malignant	1038	35418	22238	667299	1.04	0.98	1.11	1.03	0.96	1.11

© 2020 Hripcsak G. et al. *JAMA Internal Medicine.*

neoplasm										
Neutropenia or agranulocytosis	153	36797	4909	691626	0.78	0.63	0.95	0.77	0.63	0.95
Thrombocytopenia	470	36603	10058	689746	0.97	0.88	1.06	0.96	0.87	1.06
Anaphylactoid reaction	27	36925	435	694390	1.69	1.11	2.57	1.66	1.09	2.53
Angioedema	60	36901	1604	693723	0.95	0.73	1.24	0.94	0.73	1.23
Cough	4713	30591	10548<br>4	588731	1.00	0.96	1.03	0.99	0.94	1.04
Gout	840	36156	14904	683405	1.21*	1.13	1.30	1.20*	1.11	1.31
Rash	2600	33870	56767	642140	0.99	0.95	1.03	0.98	0.93	1.04
Rhabdomyolysis	41	36909	1099	694257	0.89	0.65	1.23	0.88	0.65	1.22
Vasculitis	30	36901	752	694172	0.96	0.66	1.40	0.95	0.66	1.39
Venous thromboembolic events	665	36276	16128	682348	1.00	0.93	1.09	0.99	0.91	1.09

* Statistically significant after Bonferroni correction for 55 hypotheses.

© 2020 Hripcsak G. et al. *JAMA Internal Medicine.*

eTable 11. Effectiveness and safety for meta-analysis for matching on-treatment

	Chlorthalidone[#]		Hydrochloro-thiazide		Hazard ratio uncalibrated			Calibrated hazard ratio		
Outcome	Events	Subjects	Events	Subjects	HR	Lower CI	Upper CI	HR	Lower CI	Upper CI
Acute myocardial infarction	41	36858	910	665080	0.85	0.53	1.36	0.86	0.56	1.33
All-cause mortality	20	22814	443	393740	0.92	0.55	1.55	0.92	0.57	1.49
Composite cardiovascular disease	149	36626	2948	659729	0.96	0.79	1.16	0.96	0.80	1.14
Cardiovascular-related mortality	<11	22814	133	393740	1.14	0.47	2.77	1.13	0.50	2.54
Chest pain or angina	1211	29824	26150	535929	0.93	0.82	1.05	0.93	0.83	1.04
Sudden cardiac death	<16	36911	244	665822	0.77	0.37	1.60	0.78	0.40	1.54
Hospitalization with preinfarction syndrome	28	36830	883	664099	0.82	0.54	1.26	0.83	0.57	1.23
Bradycardia	104	36521	1982	658944	1.05	0.84	1.32	1.05	0.85	1.29
Cardiac arrhythmia	635	34294	11140	623002	1.09	1.00	1.19	1.08	0.99	1.17
Syncope	347	35880	5809	646737	1.24	1.05	1.46	1.22	1.05	1.41
Heart failure	78	36799	1459	663646	1.00	0.77	1.29	0.99	0.78	1.26
Hospitalization with heart failure	62	36832	1194	664002	1.01	0.72	1.42	1.01	0.74	1.38
Fall	233	35662	4458	645578	0.95	0.80	1.14	0.96	0.82	1.12
Headache	771	30712	16358	552070	0.93	0.86	1.01	0.93	0.87	1.01
Hemorrhagic stroke	<15	36883	223	665084	0.70	0.24	2.08	0.72	0.27	1.95
Ischemic stroke	53	36776	945	662992	1.09	0.79	1.50	1.08	0.81	1.45
Stroke	60	36753	1081	662265	1.03	0.76	1.39	1.03	0.78	1.35
Transient ischemic attack	<47	36767	827	662847	1.22	0.87	1.70	1.20	0.88	1.63
Vertigo	158	36158	3237	653538	1.01	0.84	1.21	1.01	0.85	1.19
Anxiety	664	32557	13256	593650	0.91	0.83	0.99	0.91	0.84	0.99
Decreased libido	70	36633	911	661925	1.35	1.02	1.80	1.32	1.01	1.71
Dementia	<43	36752	1094	662231	0.62	0.43	0.90	0.65	0.46	0.90
Depression	513	31874	11732	578958	0.91	0.83	1.01	0.92	0.84	1.00
Impotence	369	35444	4697	646916	1.25*	1.10	1.41	1.22*	1.09	1.37
Abdominal pain	1091	28494	23477	516562	1.00	0.94	1.07	1.00	0.94	1.07
Abnormal weight gain	140	35458	4095	638051	0.67*	0.55	0.81	0.69*	0.58	0.83
Abnormal weight loss	191	36318	3230	655423	1.19	1.01	1.41	1.17	1.01	1.37
Acute pancreatitis	23	36826	493	664327	1.09	0.67	1.78	1.08	0.69	1.70
Diarrhea	463	34413	8847	625749	1.05	0.95	1.17	1.05	0.95	1.16
Gastrointestinal bleeding	50	36760	924	662966	1.27	0.92	1.76	1.24	0.92	1.67
Hepatic failure	<19	36899	225	665491	1.44	0.73	2.82	1.39	0.75	2.59
Nausea	613	34298	10866	619086	1.12	1.03	1.23	1.11	1.02	1.21
Type 2 diabetes mellitus	725	34532	11464	620660	1.20	1.06	1.36	1.18	1.05	1.32
Vomiting	438	35002	7623	631847	1.20	1.03	1.39	1.18	1.02	1.35
Acute renal failure	125	36757	1504	663662	1.30	1.05	1.61	1.27	1.04	1.55
Chronic kidney disease	213	36386	3013	659567	1.24	1.06	1.45	1.21	1.05	1.40
End stage renal disease	<15	36906	82	665828	1.51	0.55	4.17	1.46	0.57	NA
Hyperkalemia	150	36662	1986	661626	1.37	1.01	1.88	1.34	1.00	1.78
Hypokalemia	2358	35758	14903	649810	2.91*	2.51	3.39	2.66*	2.32	NA
Hypomagnesemia	112	36759	1116	663778	1.64*	1.23	2.19	1.57*	1.20	2.05
Hyponatremia	281	36676	3640	661783	1.36*	1.18	1.56	1.32*	1.16	1.50
Hypotension	180	36638	2828	661162	1.26	0.83	1.90	1.23	0.84	1.80
Measured renal dysfunction	<10	21788	85	452260	1.54	0.60	3.92	1.48	0.63	NA
Anemia	670	33095	14927	594523	0.93	0.85	1.01	0.93	0.86	1.01
Malignant neoplasm	264	35391	5017	638553	1.03	0.83	1.27	1.02	0.84	1.25

© 2020 Hripcsak G. et al. *JAMA Internal Medicine.*

Neutropenia or agranulocytosis	40	36770	940	662645	0.85	0.55	1.33	0.86	0.58	1.29
Thrombocytopenia	90	36576	1570	660936	0.95	0.70	1.28	0.95	0.72	1.25
Anaphylactoid reaction	<15	36898	70	665634	3.56	1.43	8.89	3.20	1.38	NA
Angioedema	<17	36874	307	664863	0.86	0.42	1.77	0.87	0.45	1.68
Cough	1085	30567	21470	563532	0.98	0.91	1.05	0.98	0.92	1.04
Gout	207	36130	2949	654661	1.41	1.14	1.74	1.37	1.13	1.66
Rash	574	33844	11290	616281	0.92	0.83	1.01	0.92	0.84	1.01
Rhabdomyolysis	<15	36882	155	665534	1.08	0.41	2.82	1.07	0.45	2.59
Vasculitis	<15	36874	135	665356	0.78	0.29	2.13	0.80	0.32	2.00
Venous thromboembolic events	154	36248	2898	654198	1.05	0.88	1.27	1.05	0.89	1.24

[#] A “<” in front of a number implies one of the databases had a count less than 5, so the sum is some number less than the printed number.

* Statistically significant after Bonferroni correction for 55 hypotheses.

© 2020 Hripcsak G. et al. *JAMA Internal Medicine.*

eTable 12. Effectiveness and safety for meta-analysis for matching intent-to-treat

	Chlorthalidone		Hydrochloro-thiazide		Hazard ratio uncalibrated			Calibrated hazard ratio		
Outcome	Events	Subjects	Events	Subjects	HR	Lower CI	Upper CI	HR	Lower CI	Upper CI
Acute myocardial infarction	275	36884	5991	666058	1.02	0.89	1.16	1.01	0.88	1.16
All-cause mortality	324	36943	7996	666902	1.07	0.92	1.25	1.06	0.91	1.24
Composite cardiovascular disease	892	36652	19756	660714	1.04	0.96	1.12	1.03	0.95	1.12
Cardiovascular-related mortality	79	36943	2100	666902	1.02	0.80	1.31	1.02	0.79	1.30
Chest pain or angina	4429	29843	99832	536652	0.98	0.95	1.01	0.97	0.93	1.02
Sudden cardiac death	58	36937	1703	666797	0.75	0.56	1.00	0.75	0.56	1.00
Hospitalization with preinfarction syndrome	153	36856	4418	665070	0.97	0.79	1.21	0.97	0.78	1.20
Bradycardia	472	36547	10024	659918	1.12	1.01	1.24	1.11	1.00	1.23
Cardiac arrhythmia	2250	34318	46486	623945	1.05	1.01	1.10	1.04	0.99	1.11
Syncope	1336	35903	27330	647682	1.12	1.02	1.23	1.11	1.01	1.22
Heart failure	529	36825	11149	664610	1.10	0.99	1.22	1.09	0.98	1.22
Hospitalization with heart failure	440	36858	9949	664983	1.09	0.98	1.22	1.08	0.97	1.21
Fall	1420	35688	29396	646523	1.02	0.96	1.08	1.01	0.95	1.08
Headache	2968	30734	68479	552809	0.97	0.91	1.02	0.96	0.90	1.03
Hemorrhagic stroke	53	36909	1461	666045	0.87	0.65	1.18	0.87	0.65	1.17
Ischemic stroke	294	36802	6296	663958	1.09	0.96	1.24	1.08	0.95	1.24
Stroke	326	36779	7131	663242	1.06	0.93	1.21	1.05	0.92	1.20
Transient ischemic attack	202	36793	4807	663835	1.04	0.83	1.31	1.03	0.82	1.30
Vertigo	708	36184	14919	654530	1.04	0.96	1.13	1.03	0.94	1.13
Anxiety	2522	32579	53459	594570	0.96	0.92	1.01	0.96	0.91	1.01
Decreased libido	230	36659	4568	662911	0.95	0.82	1.09	0.94	0.82	1.09
Dementia	343	36778	8040	663211	1.01	0.90	1.14	1.01	0.89	1.14
Depression	1854	31894	46033	579873	0.94	0.89	0.98	0.93	0.88	0.99
Impotence	1311	35469	19804	647877	1.12*	1.05	1.19	1.11	1.04	1.20
Abdominal pain	4499	28513	101606	517363	0.99	0.95	1.04	0.99	0.93	1.05
Abnormal weight gain	767	35482	19192	639008	0.86	0.74	1.01	0.86	0.74	1.01
Abnormal weight loss	887	36343	18262	656413	1.08	0.99	1.18	1.07	0.98	1.18
Acute pancreatitis	119	36852	2779	665315	1.04	0.85	1.26	1.03	0.84	1.25
Diarrhea	2192	34438	46441	626713	1.04	0.99	1.09	1.03	0.97	1.09
Gastrointestinal bleeding	271	36786	6168	663958	1.09	0.91	1.29	1.08	0.90	1.28
Hepatic failure	69	36925	1568	666462	0.96	0.73	1.25	0.95	0.73	1.24
Nausea	2555	34322	54010	620053	1.04	0.99	1.09	1.03	0.97	1.09
Type 2 diabetes mellitus	2036	34558	39222	621569	1.16*	1.10	1.21	1.15*	1.08	1.22
Vomiting	1808	35026	39420	632839	1.05	1.00	1.11	1.04	0.98	1.11
Acute renal failure	584	36783	11540	664622	1.09	0.99	1.19	1.08	0.98	1.19
Chronic kidney disease	877	36412	15999	660557	1.22*	1.14	1.32	1.21*	1.12	1.32
End stage renal disease	60	36932	1014	666804	1.33	0.99	1.79	1.32	0.98	1.77
Hyperkalemia	584	36687	10765	662617	1.21	1.07	1.37	1.20	1.06	1.37
Hypokalemia	5033	35781	49456	650770	2.32*	2.04	2.65	2.27*	1.97	2.63
Hypomagnesemia	492	36785	7207	664760	1.39*	1.18	1.64	1.37*	1.16	1.63
Hyponatremia	931	36702	16524	662752	1.21*	1.08	1.35	1.20	1.07	1.35
Hypotension	845	36663	16750	662149	1.15*	1.07	1.24	1.14	1.05	1.24
Measured renal dysfunction	27	21814	603	453206	1.07	0.69	1.67	1.07	0.69	1.64
Anemia	2814	33118	64525	595323	0.98	0.94	1.02	0.97	0.92	1.02
Malignant	1038	35417	21194	639526	1.06	0.99	1.13	1.05	0.97	1.13

© 2020 Hripcsak G. et al. *JAMA Internal Medicine.*

neoplasm										
Neutropenia or agranulocytosis	153	36796	4630	663630	0.75	0.59	0.95	0.75	0.59	0.95
Thrombocytopenia	470	36602	9648	661900	0.93	0.84	1.03	0.92	0.83	1.03
Anaphylactoid reaction	27	36924	404	666627	1.44	0.92	2.26	1.42	0.92	2.22
Angioedema	60	36900	1485	665839	0.96	0.72	1.26	0.95	0.72	1.25
Cough	4713	30588	99674	564417	1.00	0.96	1.03	0.99	0.95	1.04
Gout	840	36156	14266	655635	1.26*	1.16	1.35	1.24*	1.14	1.35
Rash	2600	33868	53900	617239	0.98	0.94	1.02	0.98	0.92	1.03
Rhabdomyolysis	41	36908	1048	666504	0.93	0.67	1.30	0.93	0.67	1.29
Vasculitis	30	36900	717	666344	1.02	0.68	1.51	1.01	0.68	1.50
Venous thromboembolic events	665	36274	15195	655191	1.01	0.90	1.12	1.00	0.89	1.12

* Statistically significant after Bonferroni correction for 55 hypotheses.

© 2020 Hripcsak G. et al. *JAMA Internal Medicine.*

2.4 Comparability of the populations for the three databases

eFigure 4a. Preference score distribution before propensity score adjustment. The preference score is a transformation of the propensity score that adjusts for differences in the sizes of the two treatment groups. A higher overlap indicates subjects in the two groups were more similar in terms of their predicted probability of receiving one treatment over the other. This plot shows sufficient equipoise (majority of both distributions being between 0.25 and 0.75) in all three databases that propensity score stratification should be able to create balance without discounting a large proportion of the population, but it shows sufficient difference (non-overlap) that propensity score stratification is necessary.

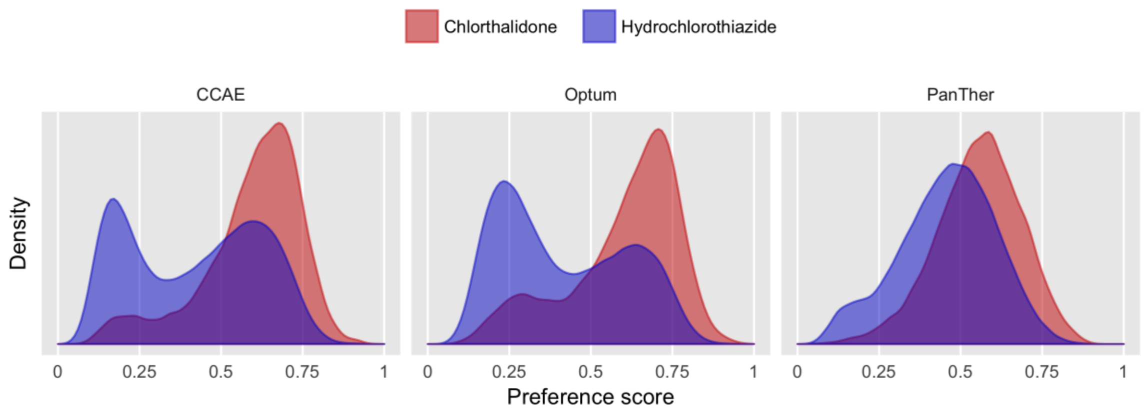

eFigure 4b. Preference score distribution after propensity score adjustment. Same plot as (a) but showing essentially perfect overlap after adjustment (matching shown here). This illustrates the success of the adjustment in achieving balance.

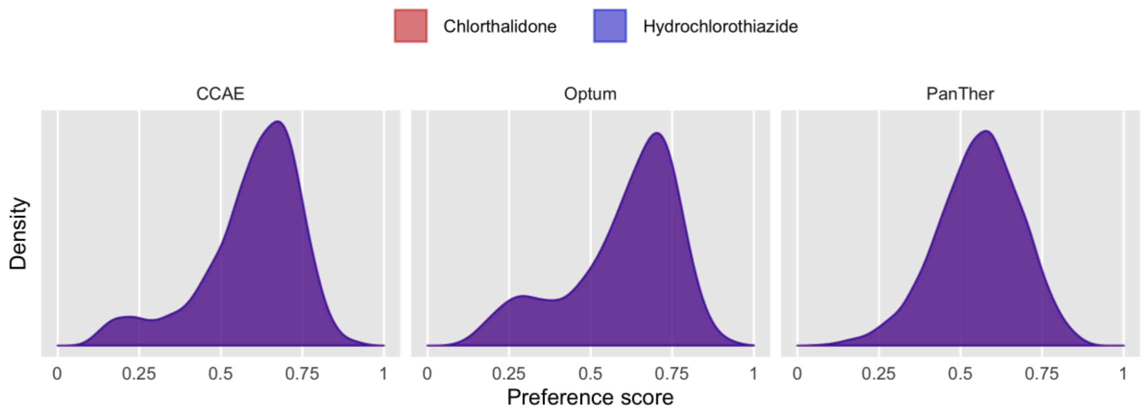

eFigure 4c. Covariate balance before and after propensity score stratification. Each dot represents the standardized difference of the means for a single covariate before and after stratification on the propensity score. The figure shows poor balance before but excellent balance after stratification, with all 60,535 to 70,072 covariates under 0.1 and most under 0.05. It demonstrates that all measured variables were successfully balanced by the adjustment and that the two cohorts were in fact similar on all measured aspects.

© 2020 Hripcsak G. et al. *JAMA Internal Medicine.*

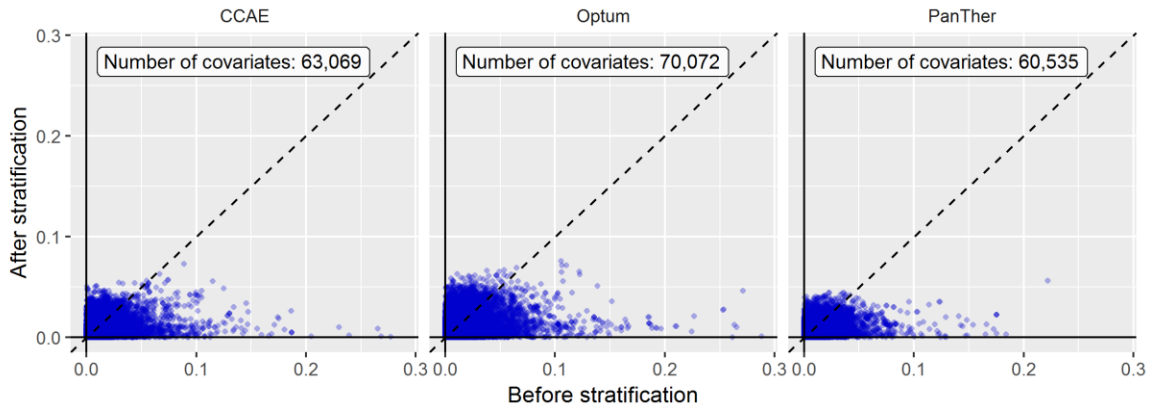

eFigure 4d. Covariate balance before and after propensity score matching. Each dot represents the standardized difference of the means for a single covariate before and after matching on the propensity score. The figure shows poor balance before but excellent balance after stratification, with all 60,405 to 69,356 covariates under 0.1 and most under 0.05. It demonstrates that all measured variables were successfully balanced by the adjustment and that the two cohorts were in fact similar on all measured aspects.

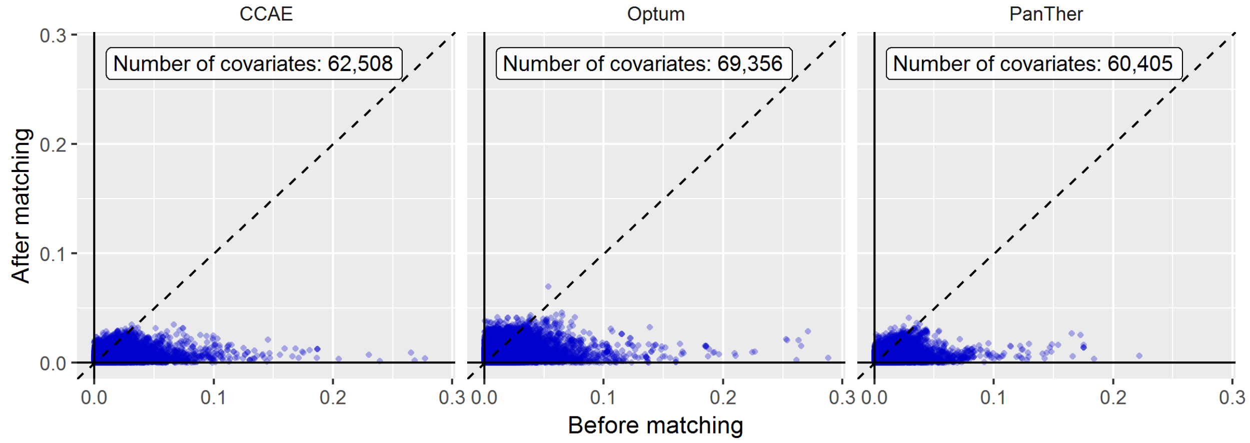

© 2020 Hripcsak G. et al. *JAMA Internal Medicine.*

2.5 Homogeneity on effectiveness and hypokalemia across databases and analytic approaches

(Only the primary effectiveness and hypokalemia results are shown here. Other outcomes can be seen summarized in the forest plots in the next section, and plots similar to these for all outcomes are available at http://data.ohdsi.org/LegendBasicViewer/ -> Specific Research Questions -> “Hypertension,” “Drug or procedure,” “Chlorthalidone,” “Hydrochlorothiazide,” <outcome of interest>.)

eFigure 5 (a-t). Homogeneity on effectiveness and hypokalemia by database and analytic approach. Hazard ratios and forest plot of the three databases and the meta-analysis for chlorthalidone versus hydrochlorothiazide for each combination. (Stratified on-treatment represents the paper’s primary results and duplicates Figure 2 in the main paper.)

e5a. Composite cardiovascular disease for stratified on-treatment

Source	HR (95% CI)	Calibrated HR (95% CI)
CCAE	0.96 (0.70-1.29)	0.96 (0.72-1.29)
Optum	1.00 (0.76-1.28)	1.04 (0.81-1.33)
Panther	1.10 (0.79-1.49)	1.00 (0.75-1.36)
Summary (I² = 0.00)	1.01 (0.86-1.20)	1.00 (0.85-1.17)

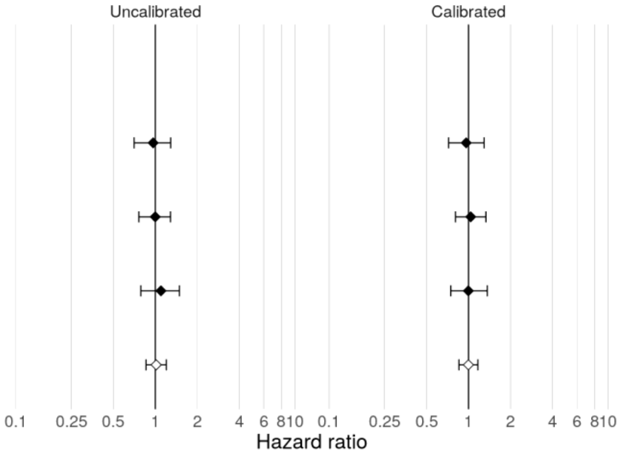

e5b. Composite cardiovascular disease for stratified intent-to-treat

Source	HR (95% CI)	Calibrated HR (95% CI)
CCAE	1.09 (0.95-1.25)	1.10 (0.95-1.29)
Optum	1.09 (0.97-1.23)	1.08 (0.92-1.27)
Panther	1.00 (0.90-1.11)	0.97 (0.87-1.10)
Summary (I² = 0.00)	1.05 (0.98-1.13)	1.04 (0.96-1.13)

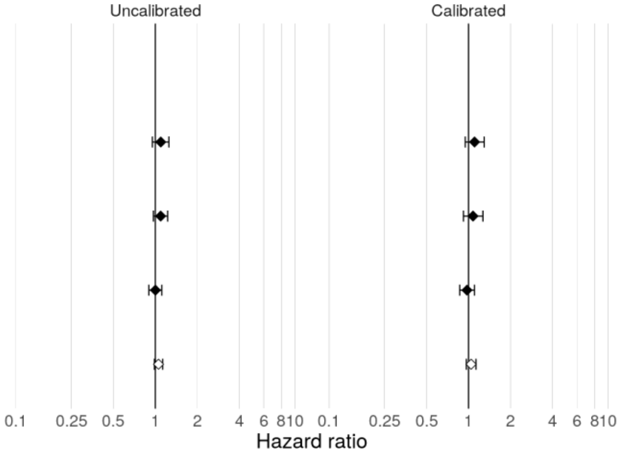

e5c. Composite cardiovascular disease for matching on-treatment

© 2020 Hripcsak G. et al. *JAMA Internal Medicine.*

Source	HR (95% CI)	Calibrated HR (95% CI)
CCAE	0.87 (0.60-1.22)	0.88 (0.63-1.25)
Optum	1.04 (0.77-1.37)	1.10 (0.83-1.46)
Panther	0.93 (0.64-1.32)	0.86 (0.62-1.20)
Summary (I² = 0.00)	0.96 (0.79-1.16)	0.96 (0.80-1.14)

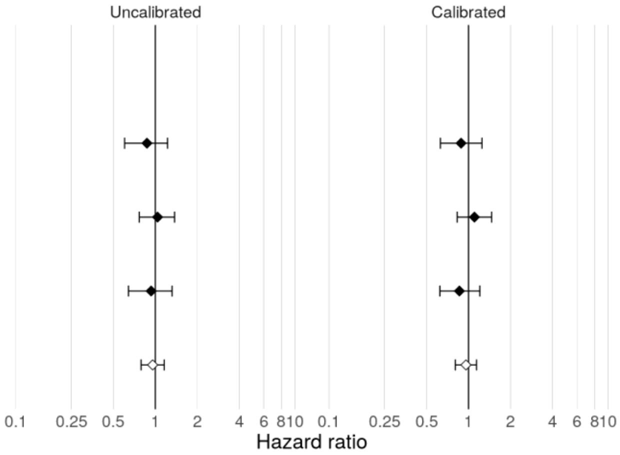

e5d. Composite cardiovascular disease for matching intent-to-treat

Source	HR (95% CI)	Calibrated HR (95% CI)
CCAE	1.03 (0.89-1.20)	1.05 (0.89-1.24)
Optum	1.11 (0.97-1.25)	1.10 (0.94-1.28)
Panther	0.99 (0.88-1.10)	0.96 (0.85-1.09)
Summary (I² = 0.00)	1.04 (0.96-1.12)	1.03 (0.95-1.12)

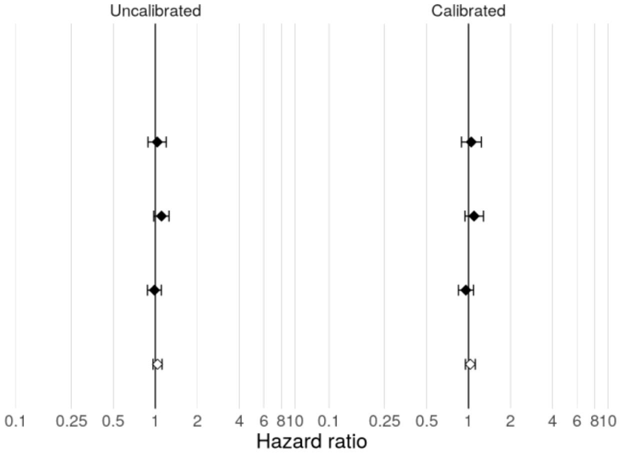

e5e. Acute myocardial infarction for stratified on-treatment

Source	HR (95% CI)	Calibrated HR (95% CI)
CCAE	0.65 (0.33-1.14)	0.66 (0.37-1.19)
Optum	0.90 (0.52-1.44)	0.93 (0.57-1.53)
Panther	1.32 (0.72-2.22)	1.16 (0.72-1.94)
Summary (I² = 0.29)	0.93 (0.63-1.36)	0.92 (0.64-1.31)

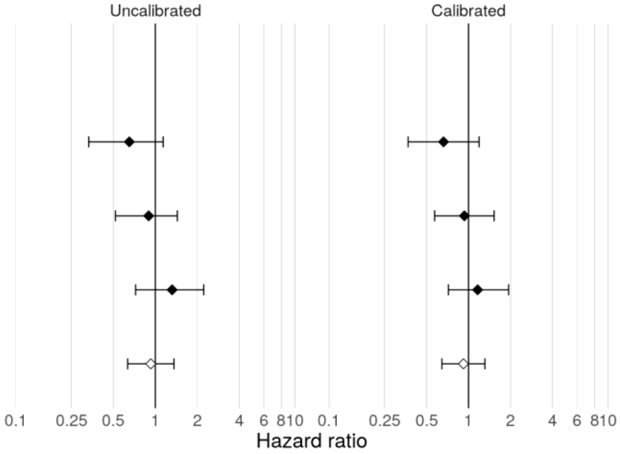

e5f. Acute myocardial infarction for stratified intent-to-treat

© 2020 Hripcsak G. et al. *JAMA Internal Medicine.*

Source	HR (95% CI)	Calibrated HR (95% CI)
CCAE	1.00 (0.78-1.26)	1.01 (0.79-1.30)
Optum	1.14 (0.91-1.39)	1.12 (0.89-1.43)
Panther	1.01 (0.82-1.22)	0.98 (0.81-1.20)
Summary (I² = 0.00)	1.05 (0.93-1.18)	1.04 (0.91-1.18)

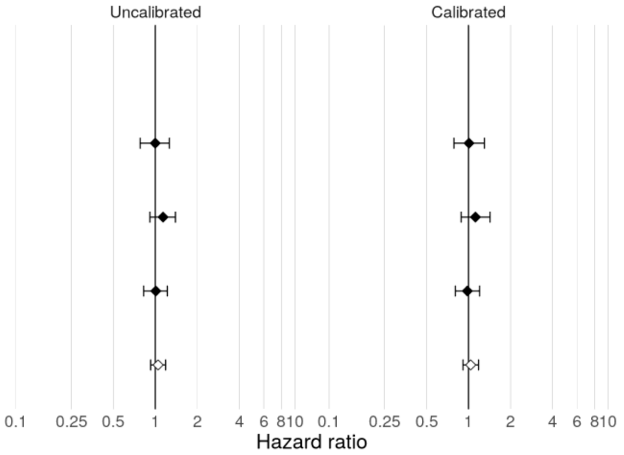

e5g. Acute myocardial infarction for matching on-treatment

Source	HR (95% CI)	Calibrated HR (95% CI)
CCAE	0.50 (0.22-0.99)	0.52 (0.25-1.07)
Optum	0.89 (0.49-1.52)	0.95 (0.55-1.66)
Panther	1.23 (0.62-2.26)	1.09 (0.63-1.92)
Summary (I² = 0.37)	0.85 (0.53-1.36)	0.86 (0.56-1.33)

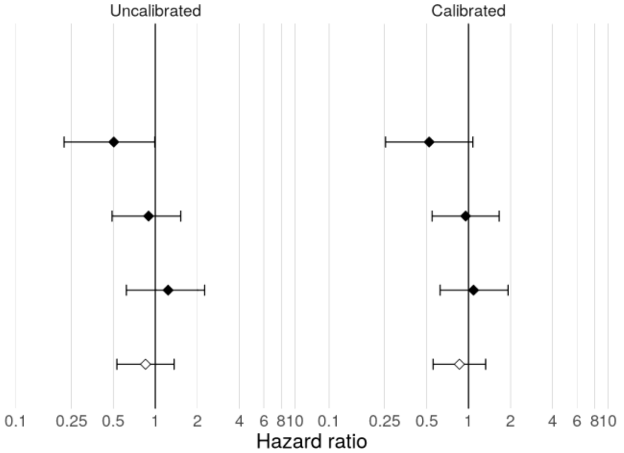

e5h. Acute myocardial infarction for matching intent-to-treat

Source	HR (95% CI)	Calibrated HR (95% CI)
CCAE	0.89 (0.67-1.15)	0.90 (0.68-1.19)
Optum	1.12 (0.88-1.40)	1.11 (0.87-1.42)
Panther	1.02 (0.82-1.25)	0.99 (0.80-1.22)
Summary (I² = 0.00)	1.02 (0.89-1.16)	1.01 (0.88-1.16)

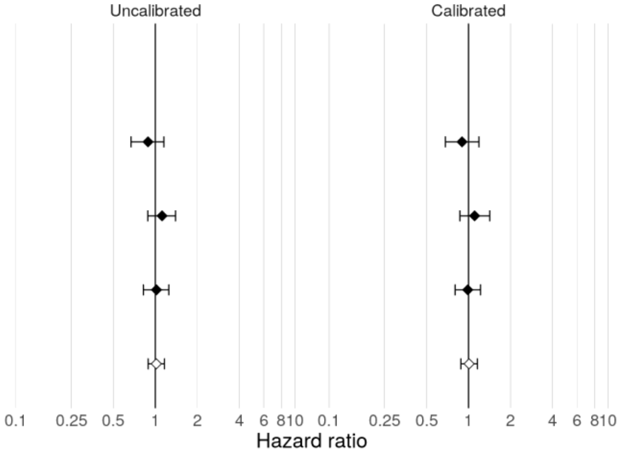

e5i. Hospitalized for heart failure for stratified on-treatment

© 2020 Hripcsak G. et al. *JAMA Internal Medicine.*

Source	HR (95% CI)	Calibrated HR (95% CI)
CCAE	1.23 (0.73-1.95)	1.21 (0.76-1.95)
Optum	1.14 (0.76-1.64)	1.18 (0.82-1.70)
Panther	0.80 (0.45-1.31)	0.77 (0.49-1.22)
Summary (I² = 0.00)	1.07 (0.82-1.39)	1.05 (0.82-1.34)

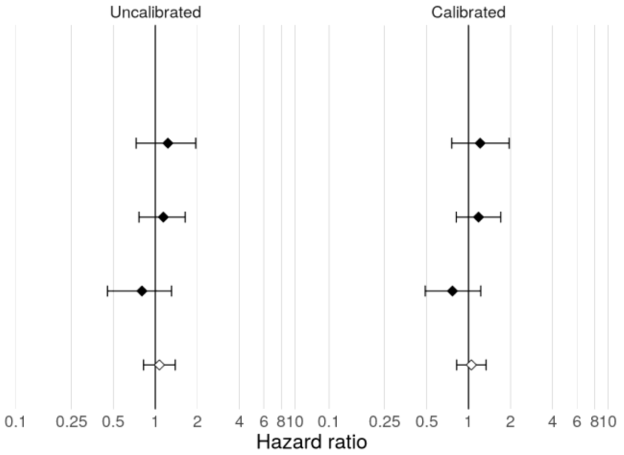

e5j. Hospitalized for heart failure for stratified intent-to-treat

Source	HR (95% CI)	Calibrated HR (95% CI)
CCAE	1.19 (0.95-1.48)	1.21 (0.95-1.53)
Optum	1.08 (0.92-1.27)	1.07 (0.88-1.31)
Panther	1.00 (0.86-1.15)	0.97 (0.84-1.14)
Summary (I² = 0.00)	1.06 (0.97-1.17)	1.05 (0.95-1.17)

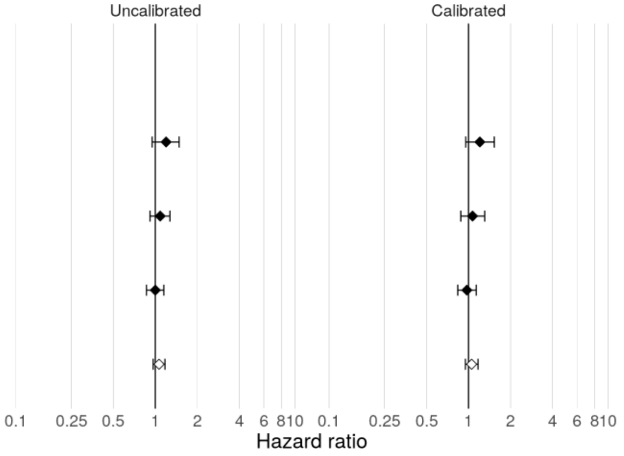

e5k. Hospitalized for heart failure for matching on-treatment

Source	HR (95% CI)	Calibrated HR (95% CI)
CCAE	1.17 (0.63-2.02)	1.18 (0.67-2.08)
Optum	1.19 (0.76-1.79)	1.26 (0.83-1.92)
Panther	0.68 (0.37-1.17)	0.66 (0.41-1.10)
Summary (I² = 0.21)	1.01 (0.72-1.42)	1.01 (0.74-1.38)

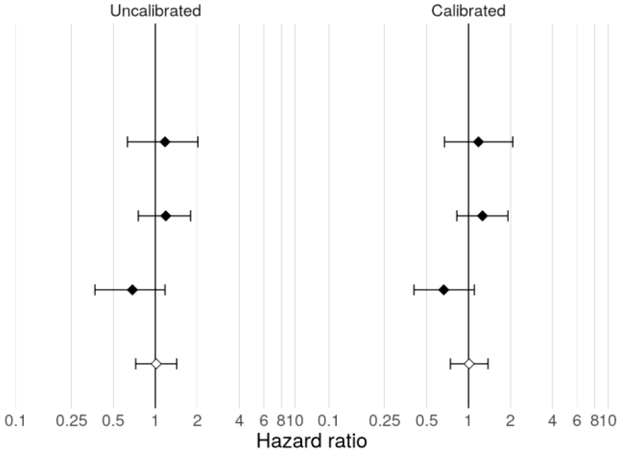

e5l. Hospitalized for heart failure for matching intent-to-treat

© 2020 Hripcsak G. et al. *JAMA Internal Medicine.*

Source	HR (95% CI)	Calibrated HR (95% CI)
CCAE	1.26 (0.98-1.59)	1.27 (0.99-1.64)
Optum	1.10 (0.92-1.31)	1.09 (0.90-1.34)
Panther	1.02 (0.87-1.18)	0.99 (0.85-1.16)
Summary (I² = 0.06)	1.09 (0.98-1.22)	1.08 (0.97-1.21)

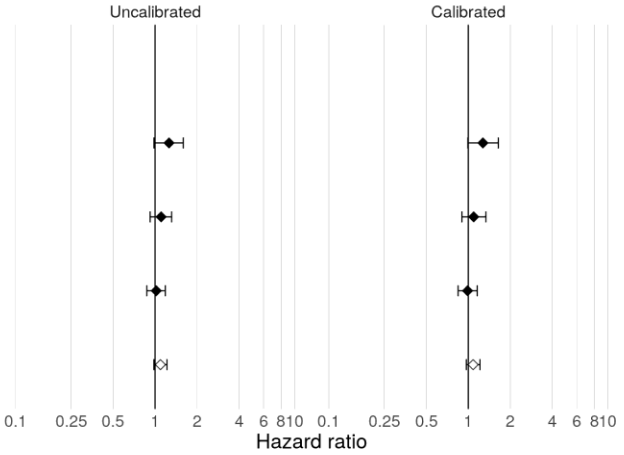

e5m. Stroke for stratified on-treatment

Source	HR (95% CI)	Calibrated HR (95% CI)
CCAE	1.18 (0.73-1.81)	1.17 (0.76-1.81)
Optum	0.96 (0.61-1.42)	0.99 (0.66-1.50)
Panther	1.36 (0.78-2.21)	1.19 (0.76-1.92)
Summary (I² = 0.00)	1.13 (0.86-1.47)	1.10 (0.86-1.41)

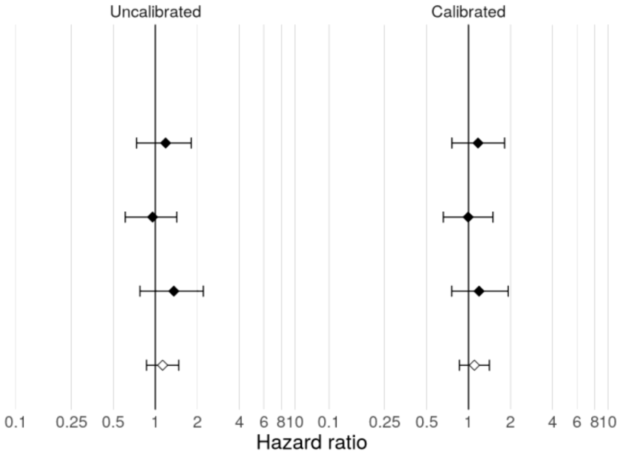

e5n. Stroke for stratified intent-to-treat

Source	HR (95% CI)	Calibrated HR (95% CI)
CCAE	1.07 (0.85-1.32)	1.08 (0.85-1.36)
Optum	1.15 (0.95-1.38)	1.14 (0.92-1.41)
Panther	1.08 (0.89-1.30)	1.05 (0.87-1.27)
Summary (I² = 0.00)	1.10 (0.98-1.23)	1.09 (0.97-1.23)

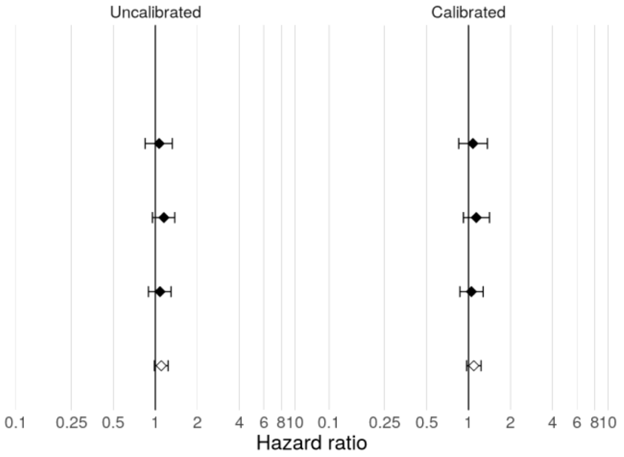

e5o. Stroke for matching on-treatment

© 2020 Hripcsak G. et al. *JAMA Internal Medicine.*

Source	HR (95% CI)	Calibrated HR (95% CI)
CCAE	0.97 (0.56-1.60)	0.98 (0.59-1.65)
Optum	0.97 (0.59-1.52)	1.04 (0.65-1.64)
Panther	1.21 (0.64-2.10)	1.07 (0.64-1.80)
Summary (I² = 0.00)	1.03 (0.76-1.39)	1.03 (0.78-1.35)

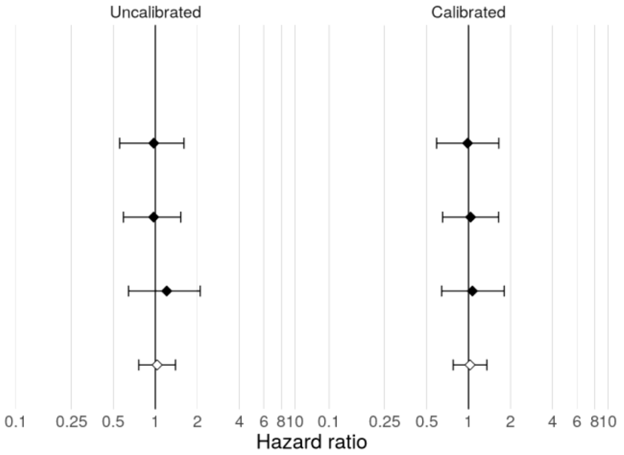

e5p. Stroke for matching intent-to-treat

Source	HR (95% CI)	Calibrated HR (95% CI)
CCAE	0.99 (0.77-1.25)	1.00 (0.78-1.29)
Optum	1.19 (0.97-1.45)	1.18 (0.95-1.48)
Panther	0.98 (0.80-1.20)	0.96 (0.78-1.17)
Summary (I² = 0.11)	1.06 (0.93-1.21)	1.05 (0.92-1.20)

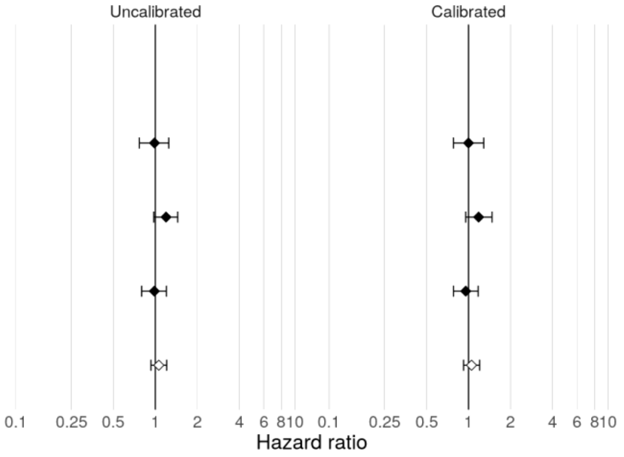

e5q. Hypokalemia for stratified on-treatment

Source	HR (95% CI)	Calibrated HR (95% CI)
CCAE	3.45 (3.20-3.71)	3.23 (2.77-3.91)
Optum	2.78 (2.53-3.05)	2.78 (2.54- NA)
Panther	2.78 (2.59-2.98)	2.16 (1.77-2.80)
Summary (I² = 0.90)	2.99 (2.58-3.46)	2.72 (2.38-3.12)

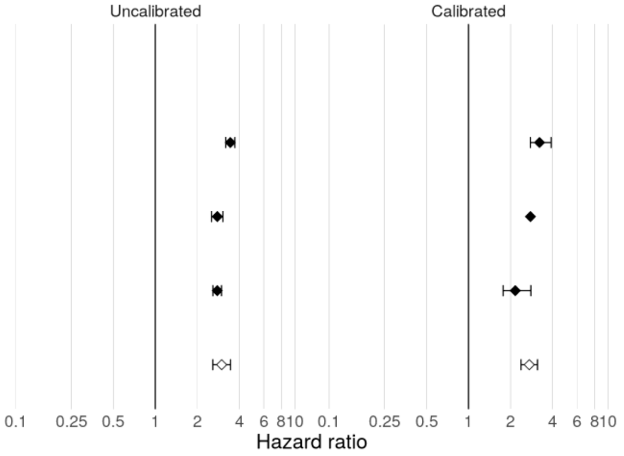

e5r. Hypokalemia for stratified intent-to-treat

© 2020 Hripcsak G. et al. *JAMA Internal Medicine.*

Source	HR (95% CI)	Calibrated HR (95% CI)
CCAE	2.60 (2.45-2.74)	2.61 (2.31-2.98)
Optum	2.19 (2.05-2.34)	2.16 (1.88-2.52)
Panther	2.08 (2.00-2.17)	1.97 (1.80-2.18)
Summary (I² = 0.95)	2.28 (1.98-2.62)	2.23 (1.92-2.59)

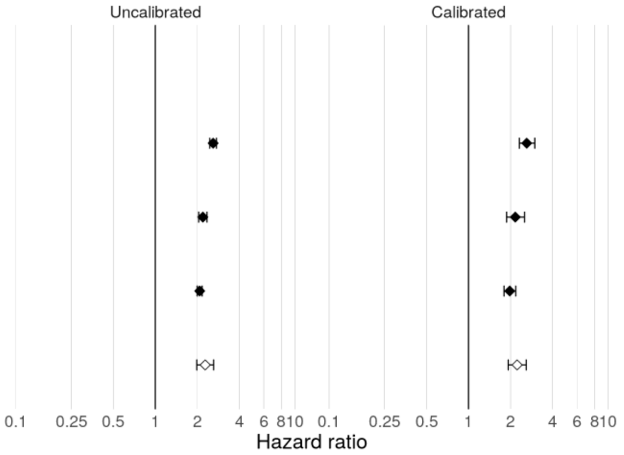

e5s. Hypokalemia for matching on-treatment

Source	HR (95% CI)	Calibrated HR (95% CI)
CCAE	3.38 (3.10-3.69)	3.26 (2.82-3.91)
Optum	2.61 (2.34-2.91)	2.72 (2.44- NA)
Panther	2.79 (2.57-3.01)	2.14 (1.77-2.68)
Summary (I² = 0.88)	2.91 (2.51-3.39)	2.66 (2.32- NA)

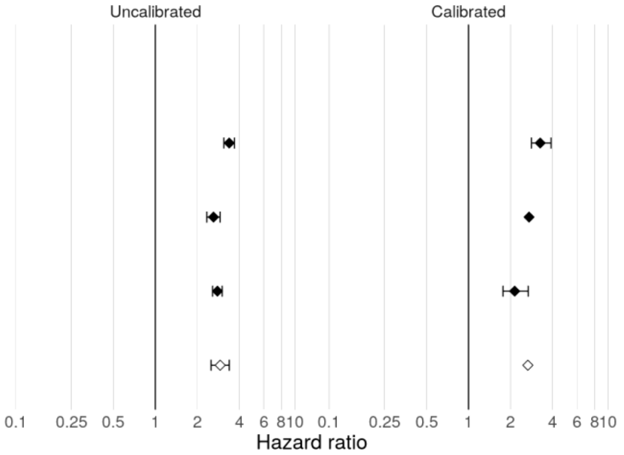

e5t. Hypokalemia for matching intent-to-treat

Source	HR (95% CI)	Calibrated HR (95% CI)
CCAE	2.63 (2.47-2.79)	2.64 (2.35-3.01)
Optum	2.24 (2.09-2.41)	2.24 (1.96-2.60)
Panther	2.13 (2.03-2.22)	2.00 (1.82-2.20)
Summary (I² = 0.93)	2.32 (2.04-2.65)	2.27 (1.97-2.63)

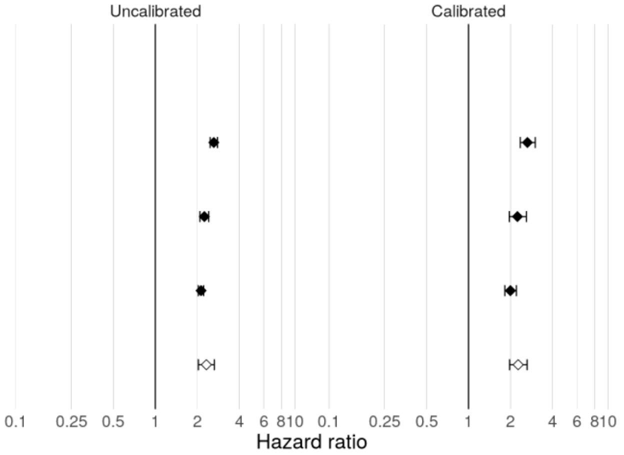

© 2020 Hripcsak G. et al. *JAMA Internal Medicine.*

2.6 Forest plot of safety and effectiveness outcomes across analytic approaches

eFigure 6(a-d). Forest plot of safety and effectiveness across analyses. Forest plot of hazard ratio estimates and calibrated 95% confidence intervals for chlorthalidone versus hydrochlorothiazide for 55 safety and effectiveness outcomes for three databases and the meta-analysis. Confidence intervals that include 1 (no effect) are greyed. (The forest plot for stratification on-treatment for the meta-analysis is shown in Figure 3 of the main paper).

eFigure 6a. Forest plot of stratification on-treatment

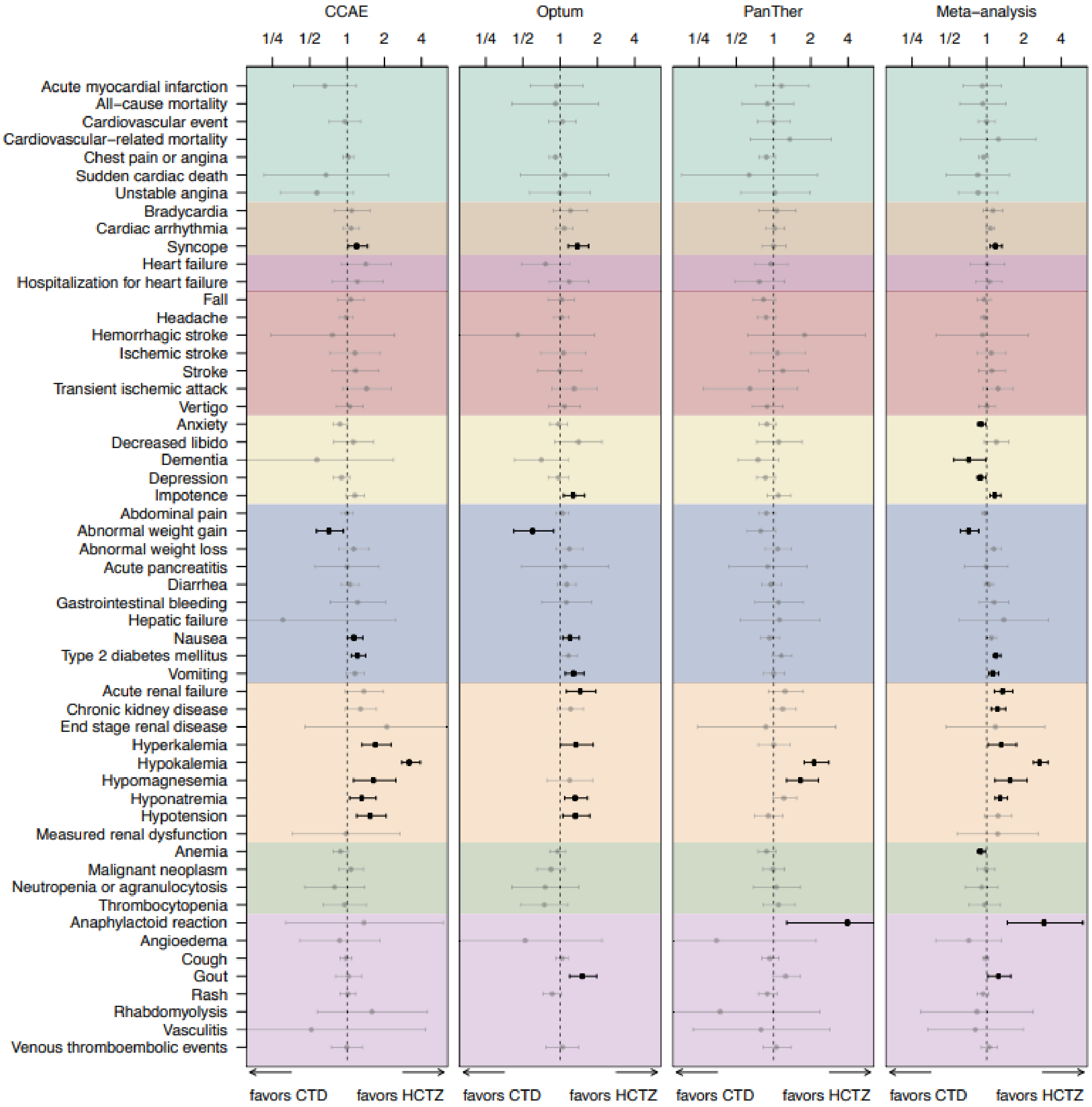

© 2020 Hripcsak G. et al. *JAMA Internal Medicine.*

eFigure 6b. Forest plot for stratification intent-to-treat

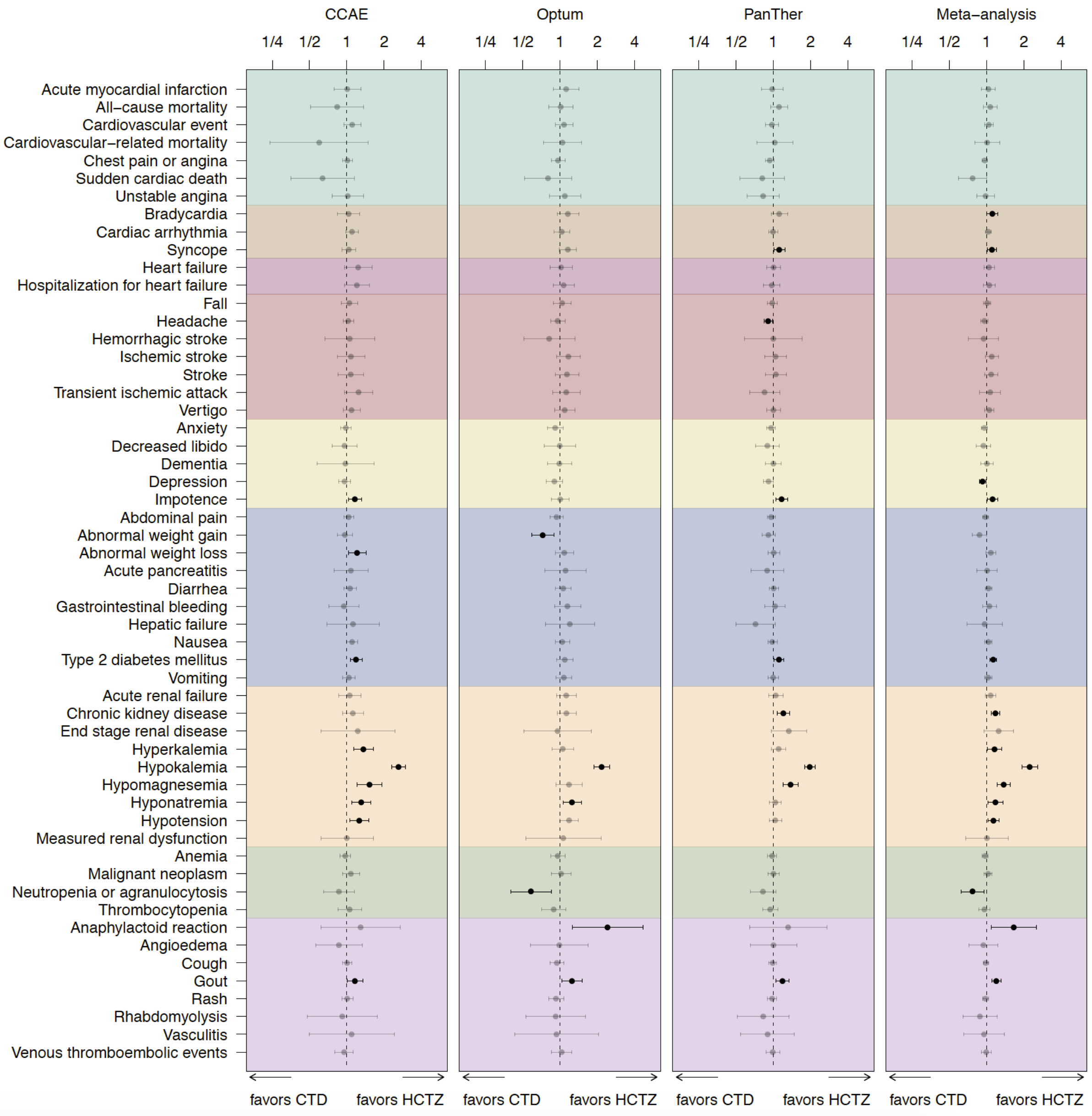

© 2020 Hripcsak G. et al. *JAMA Internal Medicine.*

eFigure 6c. Forest plot for matching on-treatment

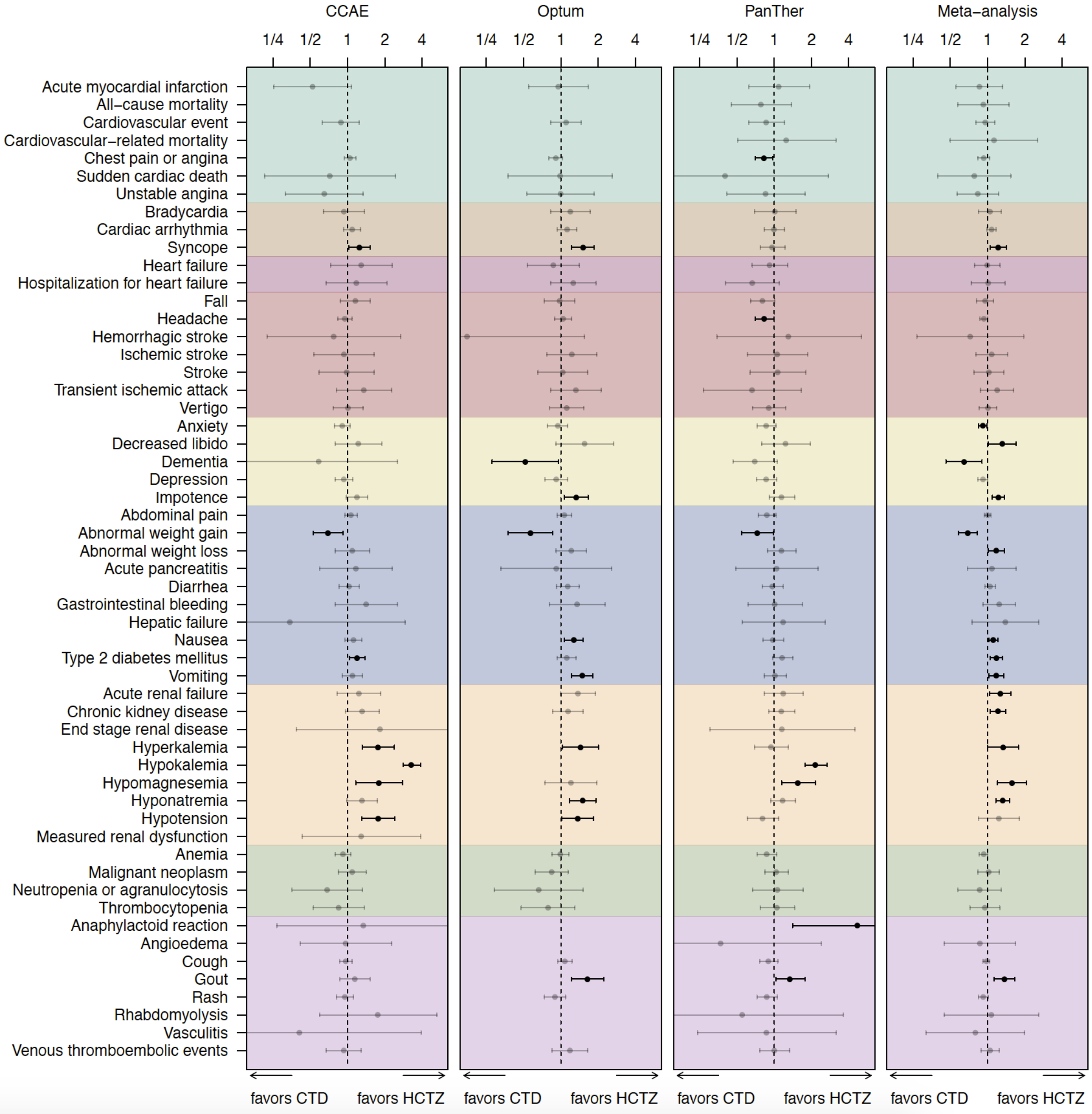

© 2020 Hripcsak G. et al. *JAMA Internal Medicine.*

eFigure 6d. Forest plot for matching intent-to-treat

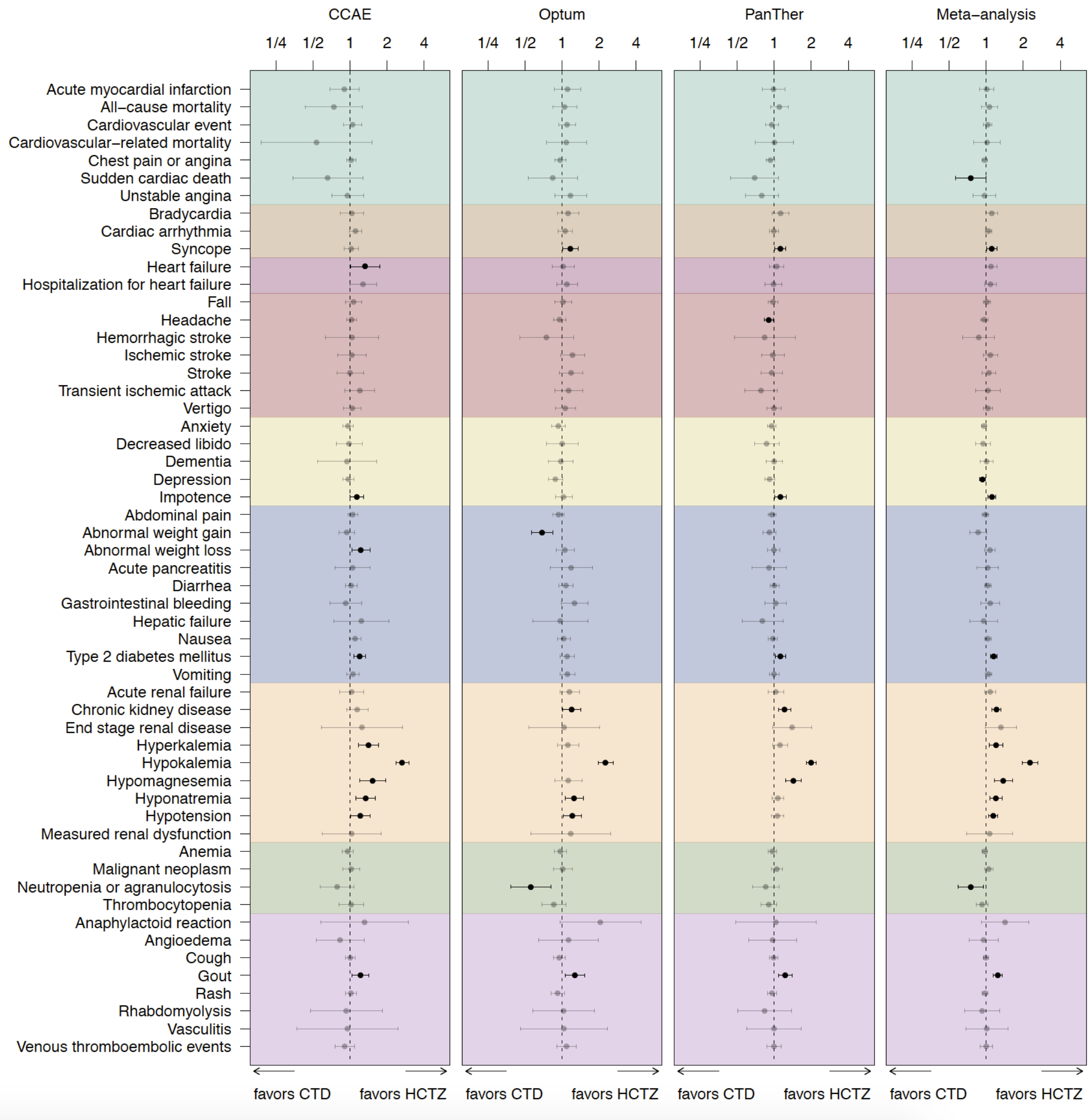

2.7 Kaplan-Meier plots for effectiveness and hypokalemia outcomes across databases and analytic approaches

eFigure 7. Kaplan-Meier plots for effectiveness and hypokalemia. (Only the primary effectiveness and hypokalemia results are shown here. Kaplan-Meier plots for other outcomes are available at http://data.ohdsi.org/LegendBasicViewer/ -> Specific Research Questions -> "Hypertension," "Drug or procedure," "Chlorthalidone," "Hydrochlorothiazide," <outcome of interest>.)

© 2020 Hripcsak G. et al. *JAMA Internal Medicine.*

Kaplan-Meier plot for composite cardiovascular disease in CCAE for stratified on-treatment

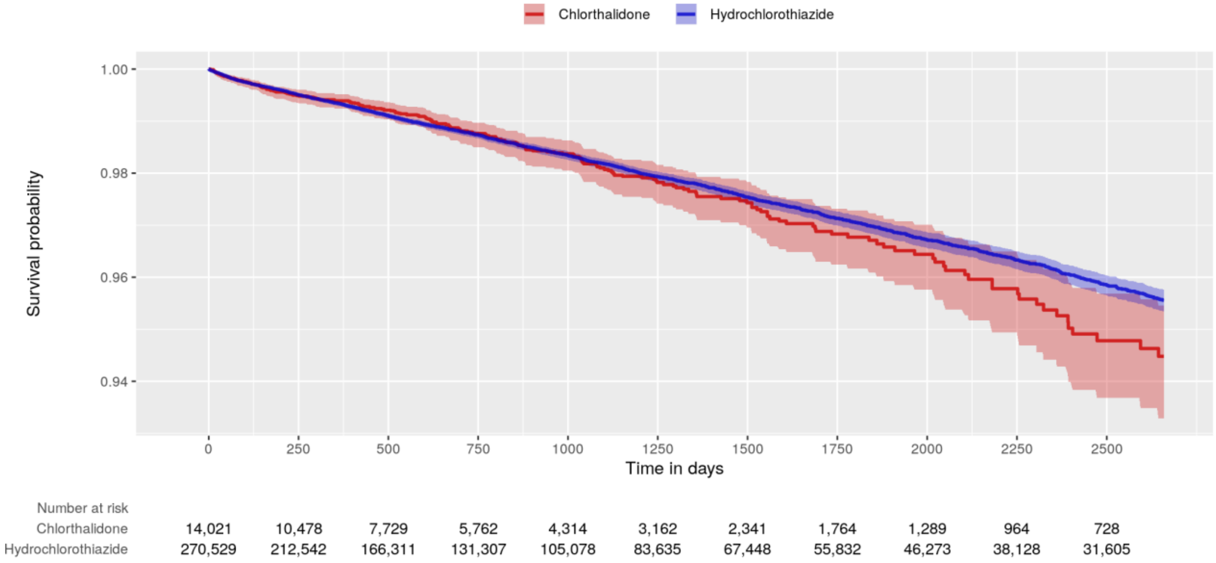

Kaplan-Meier plot for composite cardiovascular disease in Optum for stratified on-treatment

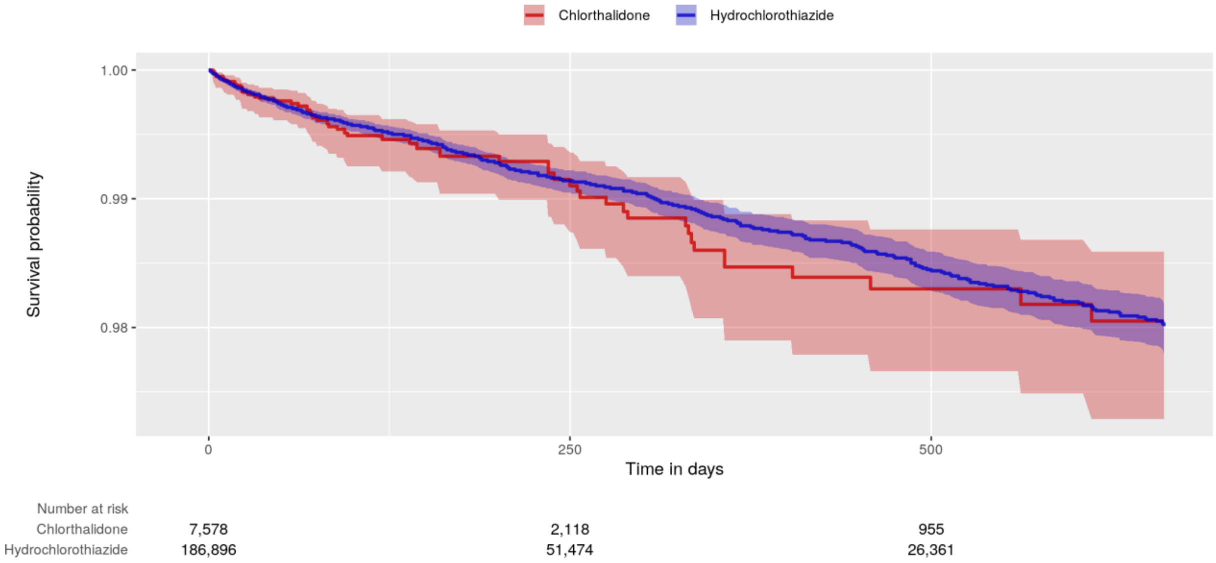

© 2020 Hripcsak G. et al. *JAMA Internal Medicine.*

Kaplan-Meier plot for composite cardiovascular disease in Panther for stratified on-treatment

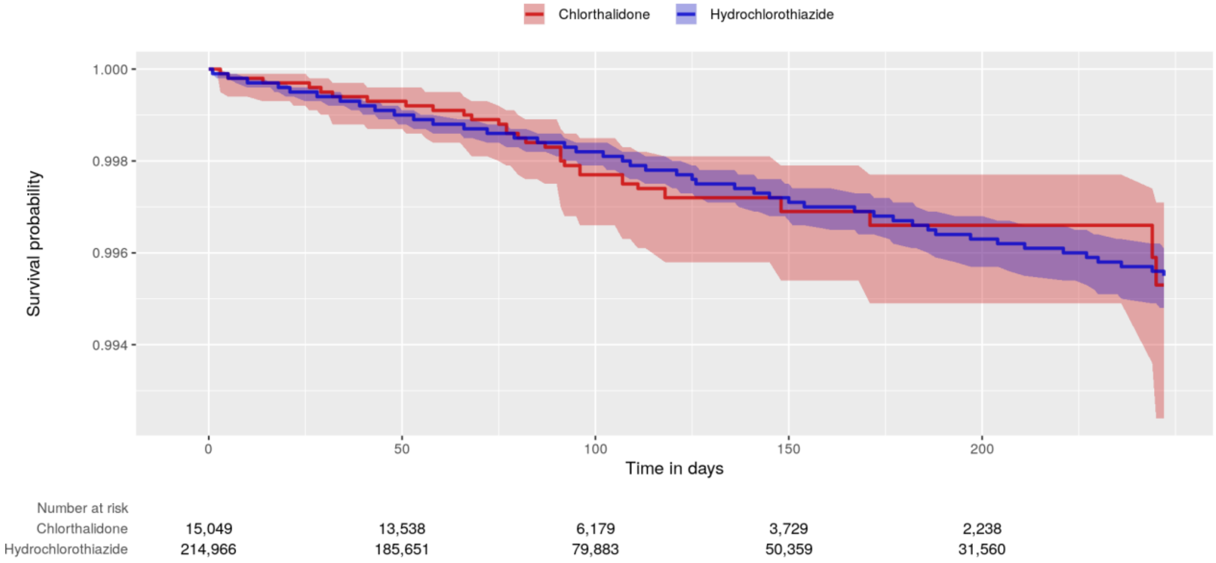

Kaplan-Meier plot for composite cardiovascular disease in CCAE for stratified intent-to-treat

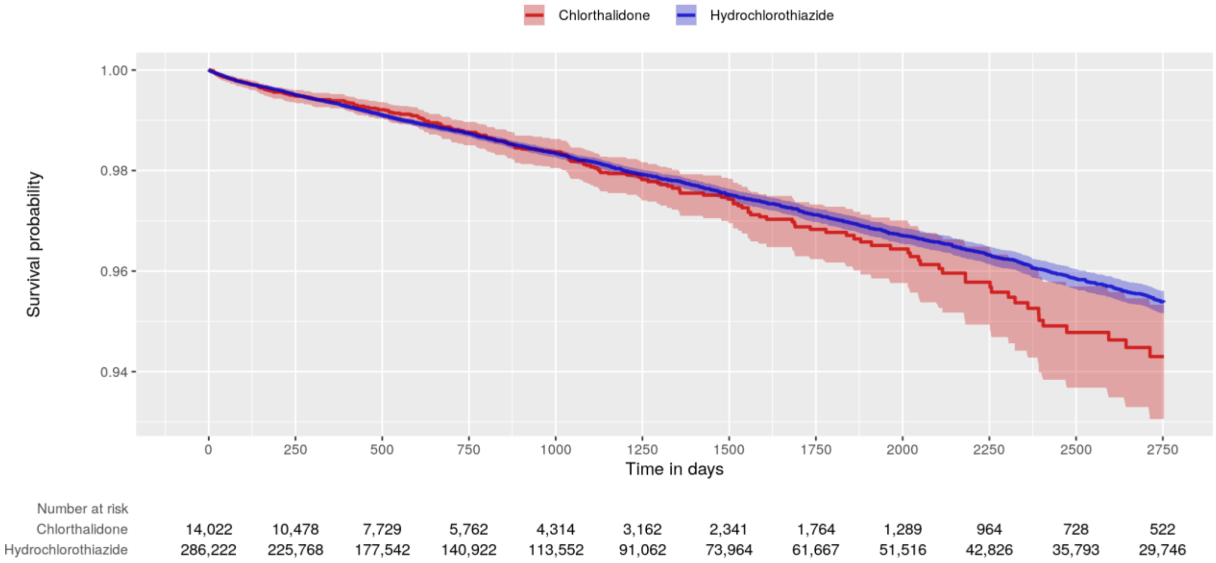

© 2020 Hripcsak G. et al. *JAMA Internal Medicine.*

Kaplan-Meier plot for composite cardiovascular disease in Optum for stratified intent-to-treat

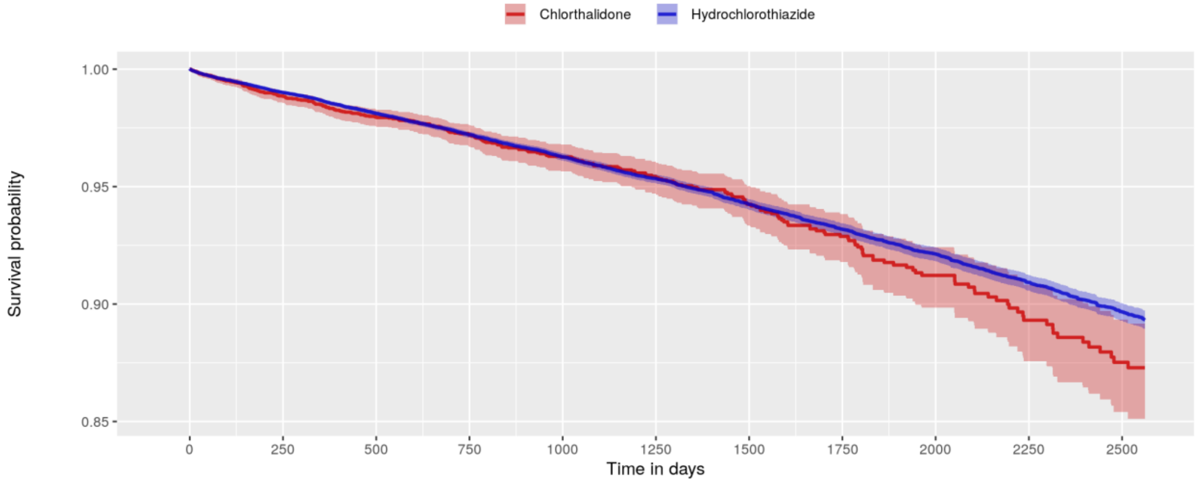

Number at risk											
Chlorthalidone	7,583	5,776	4,233	3,229	2,510	1,947	1,469	1,081	774	508	382
Hydrochlorothiazide	187,232	147,040	115,097	91,334	74,271	60,460	49,053	40,073	32,517	24,963	20,307

Kaplan-Meier plot for composite cardiovascular disease in Panther for stratified intent-to-treat

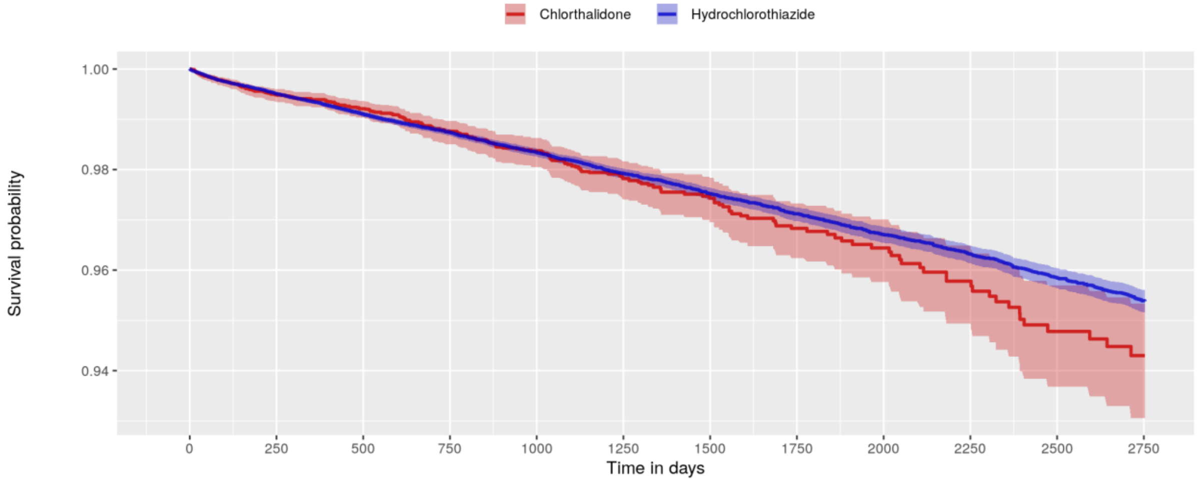

Number at risk												
Chlorthalidone	14,022	10,478	7,729	5,762	4,314	3,162	2,341	1,764	1,289	964	728	522
Hydrochlorothiazide	286,222	225,768	177,542	140,922	113,552	91,062	73,964	61,667	51,516	42,826	35,793	29,746

© 2020 Hripcsak G. et al. *JAMA Internal Medicine.*

Kaplan-Meier plot for composite cardiovascular disease in CCAE for matching on-treatment

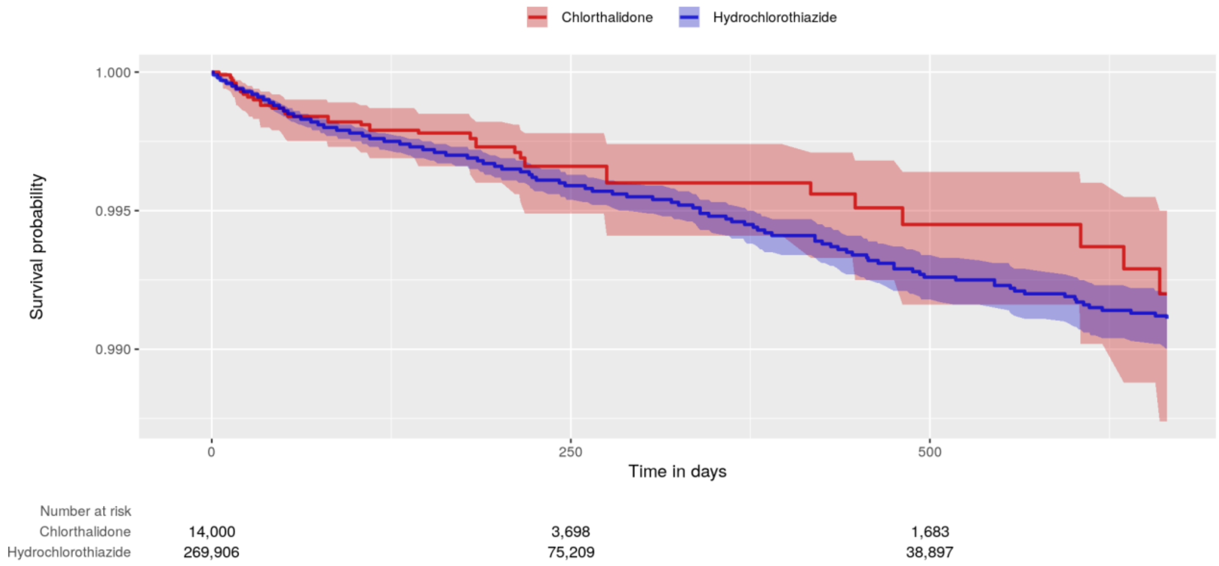

Kaplan-Meier plot for composite cardiovascular disease in Optum for matching on-treatment

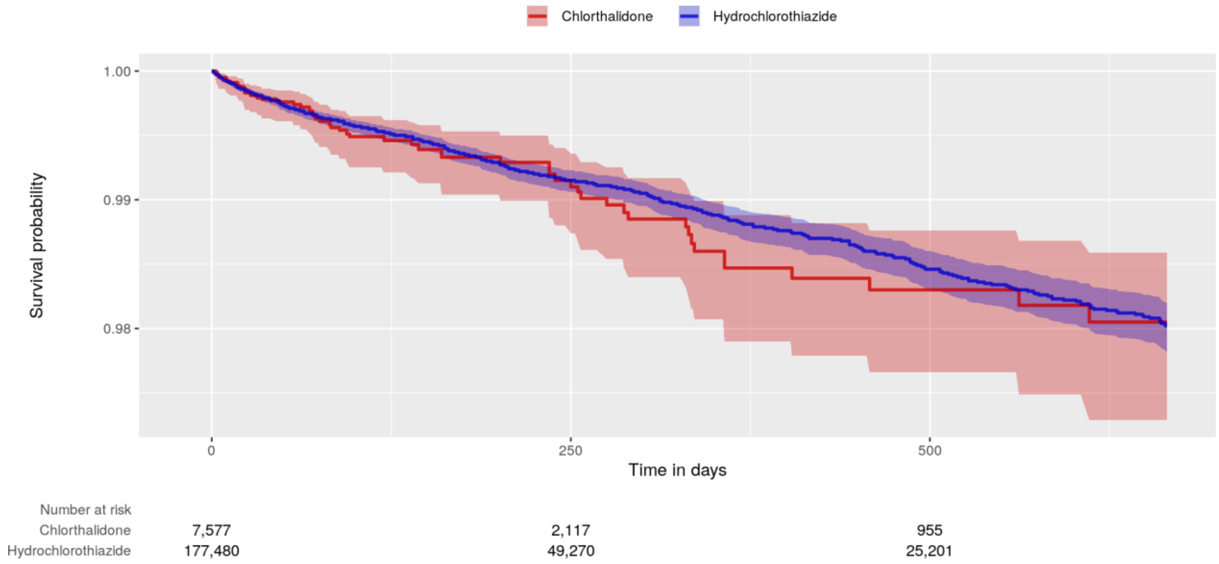

© 2020 Hripcsak G. et al. *JAMA Internal Medicine.*

Kaplan-Meier plot for composite cardiovascular disease in Panther for matching on-treatment

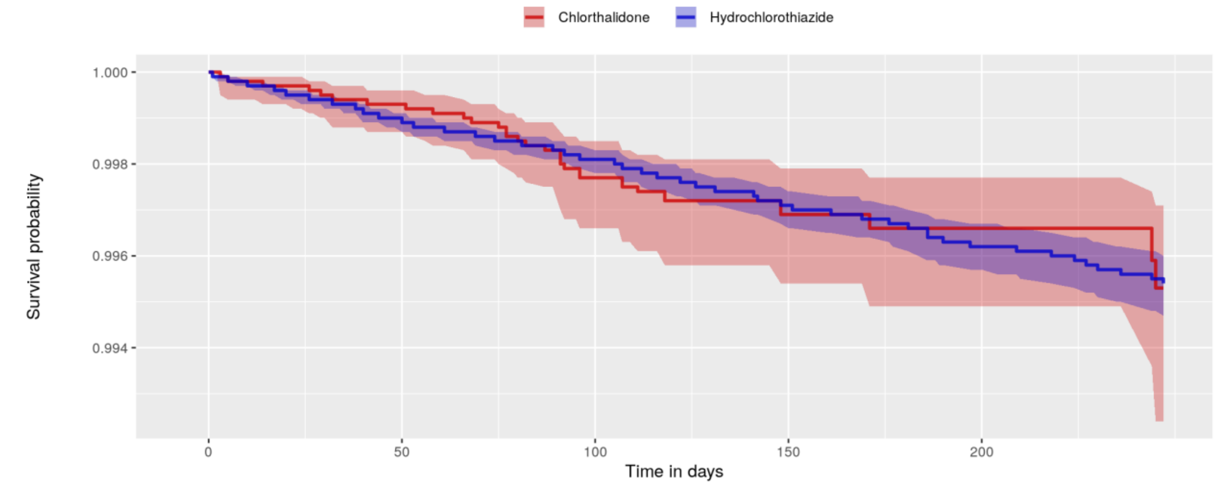

Number at risk					
Chlorthalidone	15,049	13,538	6,179	3,729	2,238
Hydrochlorothiazide	212,343	183,357	79,078	49,834	31,234

Kaplan-Meier plot for composite cardiovascular disease in CCAE for matching intent-to-treat

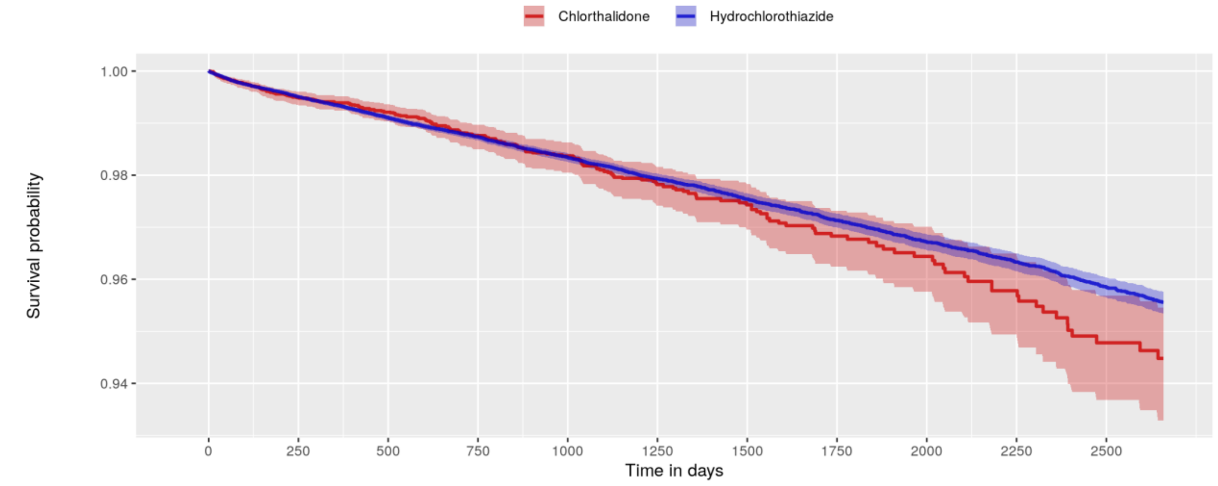

Number at risk											
Chlorthalidone	14,021	10,478	7,729	5,762	4,314	3,162	2,341	1,764	1,289	964	728
Hydrochlorothiazide	270,529	212,542	166,311	131,307	105,078	83,635	67,448	55,832	46,273	38,128	31,605

© 2020 Hripcsak G. et al. *JAMA Internal Medicine.*

Kaplan-Meier plot for composite cardiovascular disease in Optum for matching intent-to-treat

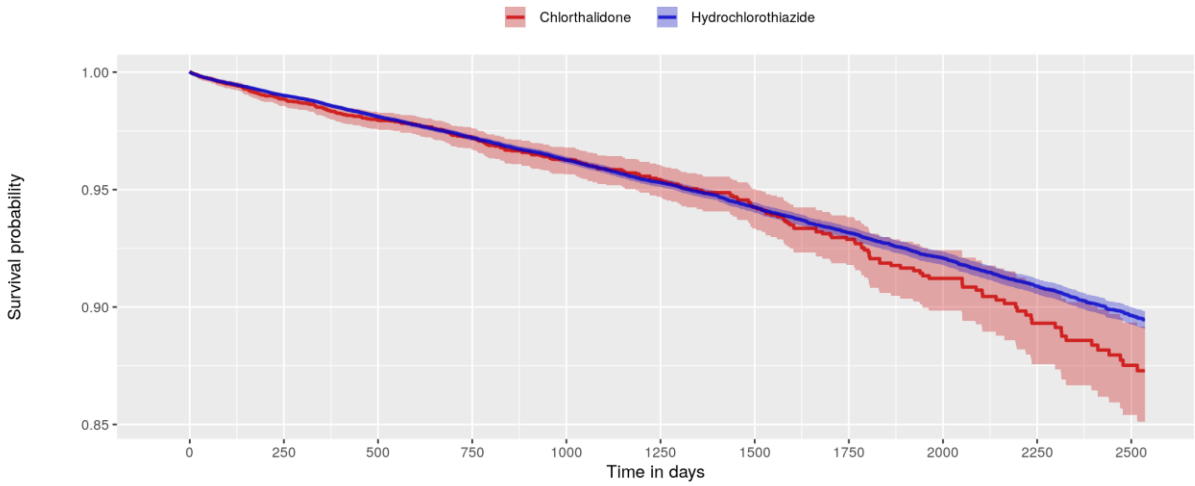

Number at risk											
Chlorthalidone	7,582	5,775	4,233	3,229	2,510	1,947	1,469	1,081	774	508	382
Hydrochlorothiazide	177,816	139,675	109,172	86,477	70,242	57,059	46,201	37,609	30,430	23,247	18,829

Kaplan-Meier plot for composite cardiovascular disease in Panther for matching intent-to-treat

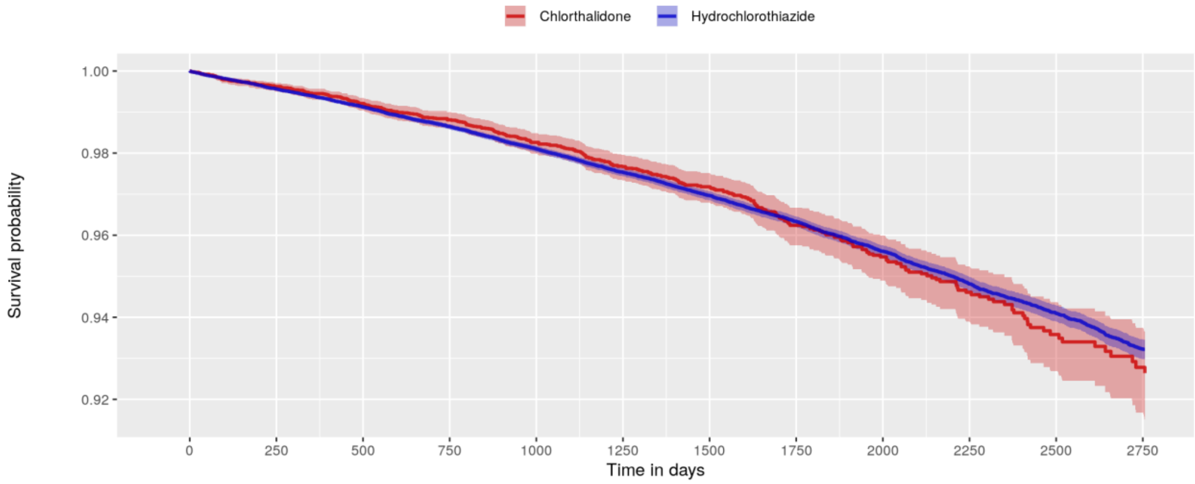

Number at risk												
Chlorthalidone	15,049	12,384	10,691	9,174	7,578	6,067	4,799	3,611	2,499	1,730	1,037	648
Hydrochlorothiazide	212,369	181,722	161,947	142,728	123,156	103,935	86,760	71,371	56,612	44,389	32,638	22,392

© 2020 Hripcsak G. et al. *JAMA Internal Medicine.*

Kaplan-Meier plot for acute myocardial infarction in CCAE for stratified on-treatment

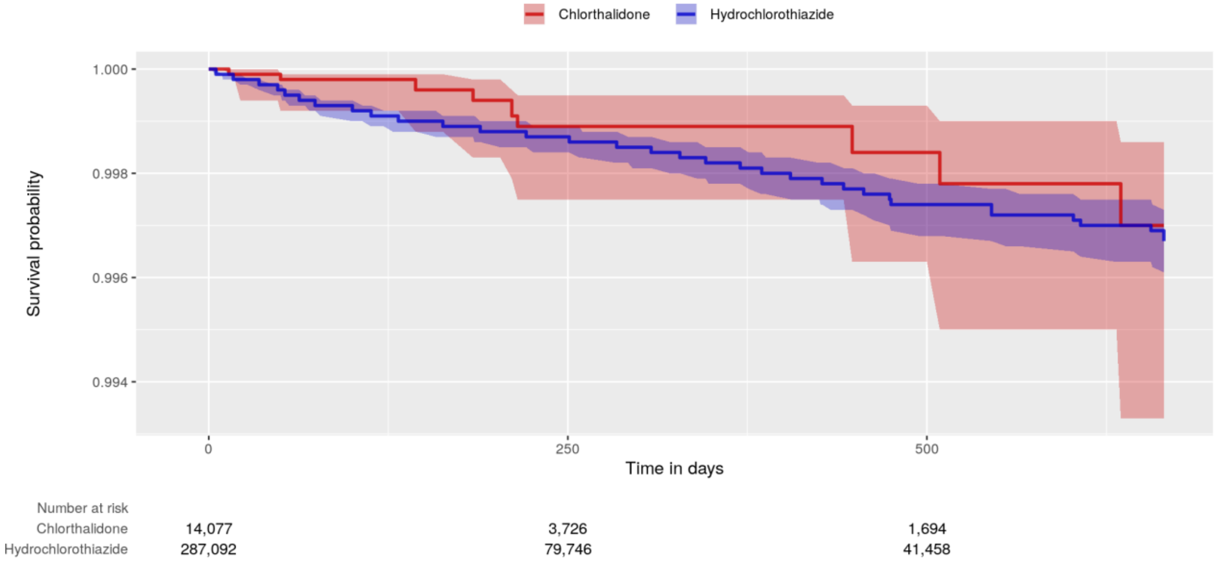

Kaplan-Meier plot for acute myocardial infarction in Optum for stratified on-treatment

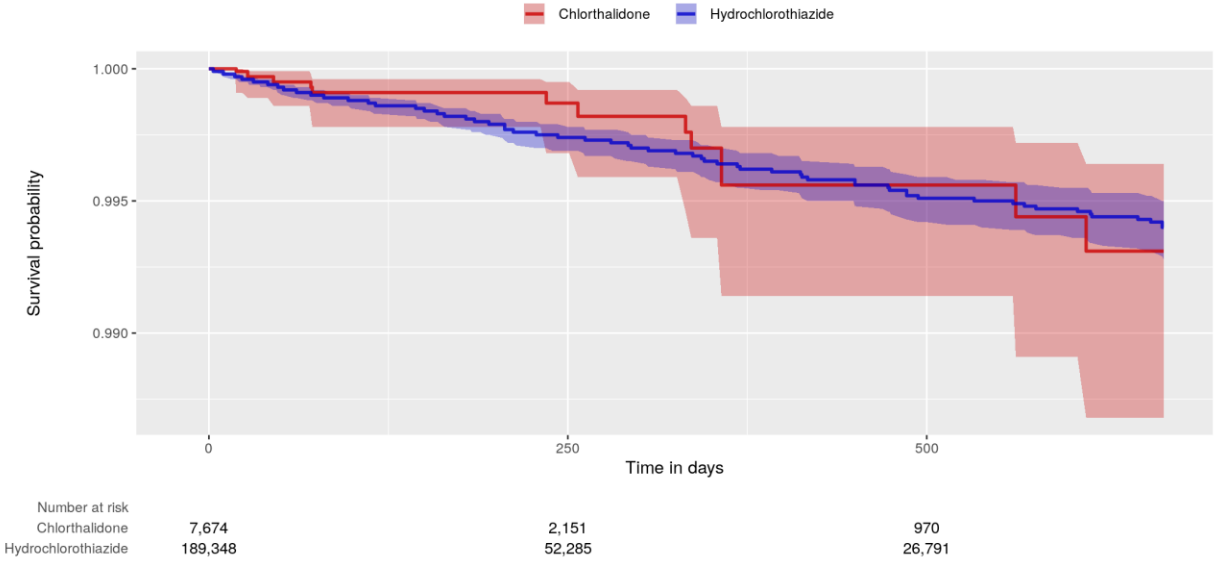

© 2020 Hripcsak G. et al. *JAMA Internal Medicine.*

Kaplan-Meier plot for acute myocardial infarction in Panther for stratified on-treatment

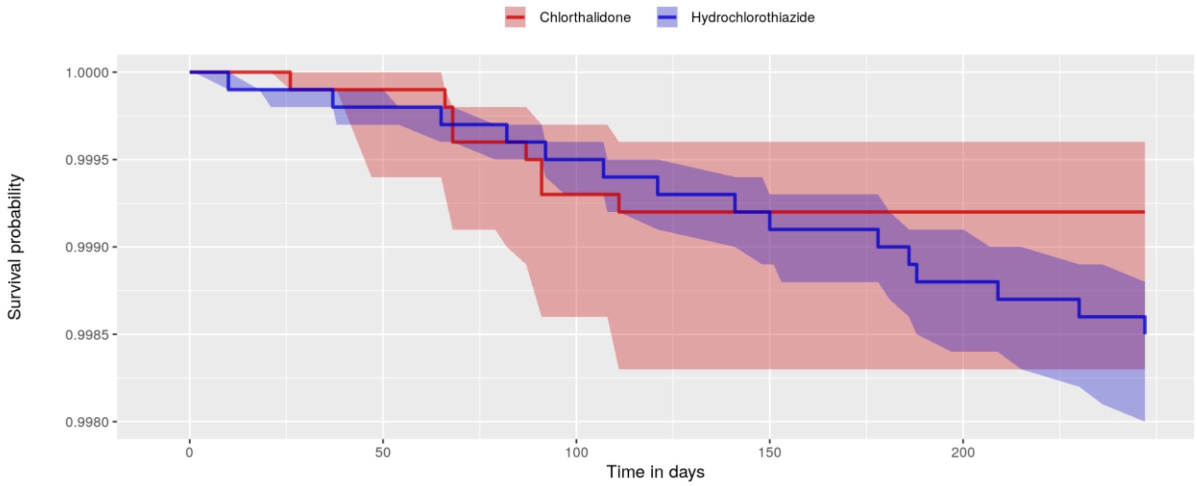

Number at risk					
Chlorthalidone	15,108	13,599	6,215	3,749	2,252
Hydrochlorothiazide	215,931	186,560	80,308	50,661	31,755

Kaplan-Meier plot for acute myocardial infarction in CCAE for stratified intent-to-treat

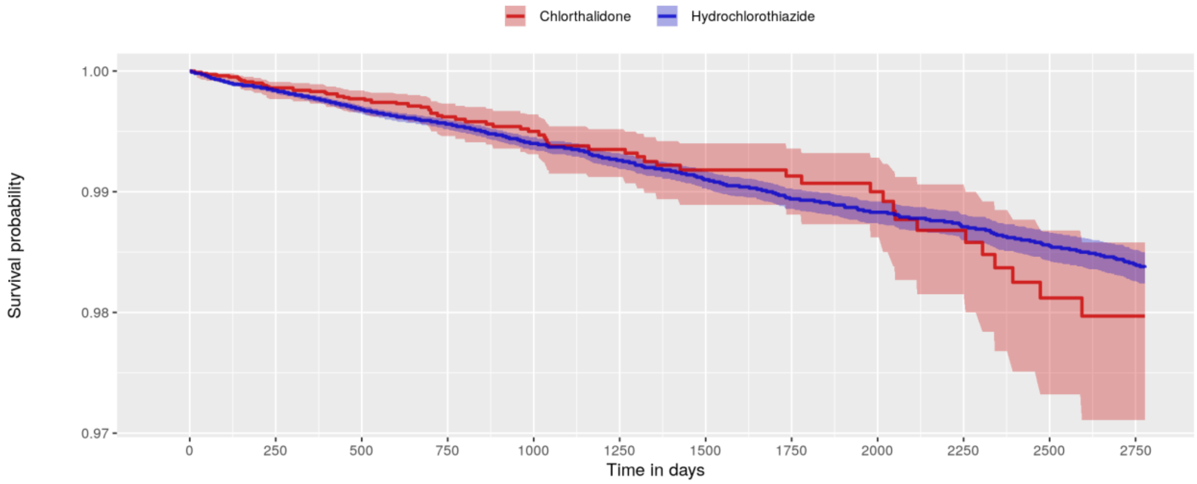

Number at risk												
Chlorthalidone	14,098	10,563	7,804	5,840	4,387	3,235	2,392	1,809	1,329	990	747	537
Hydrochlorothiazide	288,073	227,706	179,330	142,550	115,008	92,329	75,069	62,682	52,460	43,673	36,564	30,419

© 2020 Hripcsak G. et al. *JAMA Internal Medicine.*

Kaplan-Meier plot for acute myocardial infarction in Optum for stratified intent-to-treat

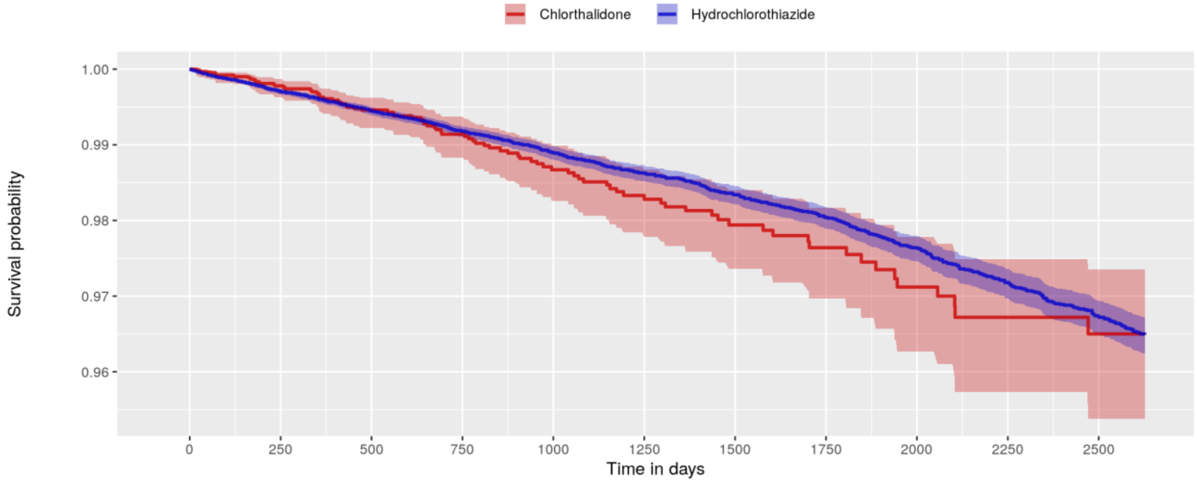

Number at risk											
Chlorthalidone	7,679	5,898	4,334	3,324	2,597	2,020	1,536	1,133	818	543	413
Hydrochlorothiazide	189,692	149,810	117,857	93,967	76,810	62,904	51,329	42,204	34,429	26,603	21,786

Kaplan-Meier plot for acute myocardial infarction in Panther for stratified intent-to-treat

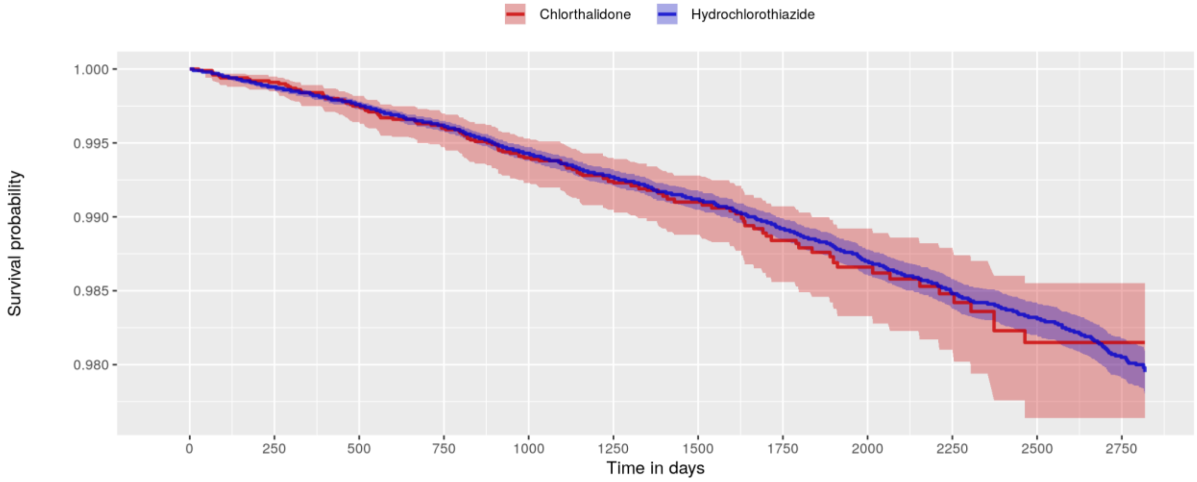

Number at risk												
Chlorthalidone	15,108	12,468	10,785	9,277	7,682	6,177	4,900	3,715	2,588	1,801	1,078	679
Hydrochlorothiazide	215,960	185,605	166,078	147,018	127,521	108,302	91,031	75,445	60,437	47,988	35,783	25,045

© 2020 Hripcsak G. et al. *JAMA Internal Medicine.*

Kaplan-Meier plot for acute myocardial infarction in CCAE for matching on-treatment

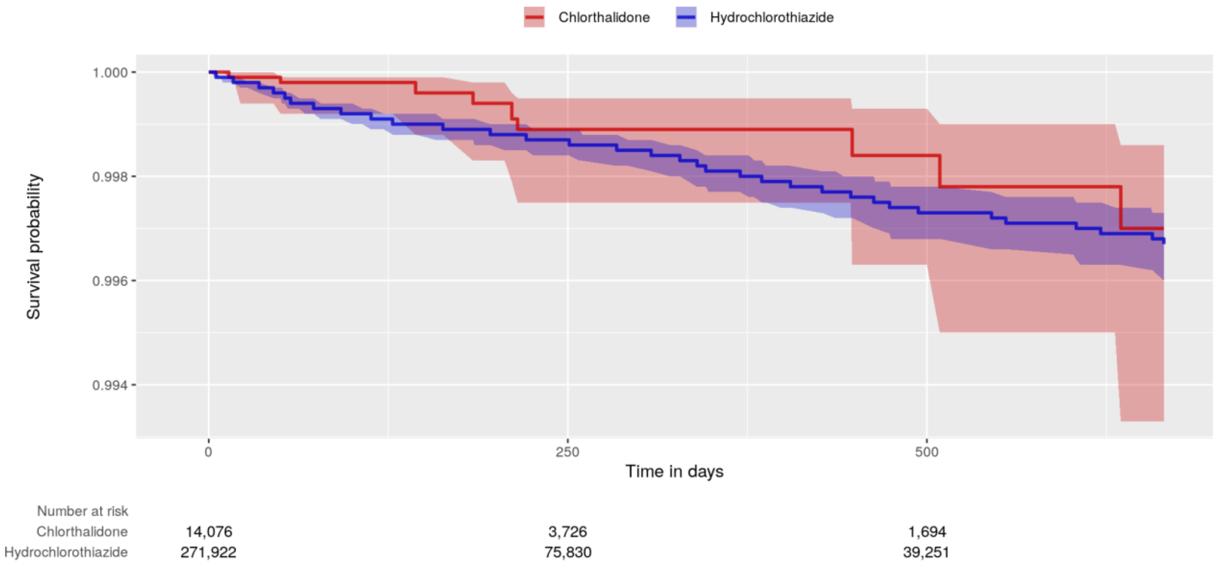

Kaplan-Meier plot for acute myocardial infarction in Optum for matching on-treatment

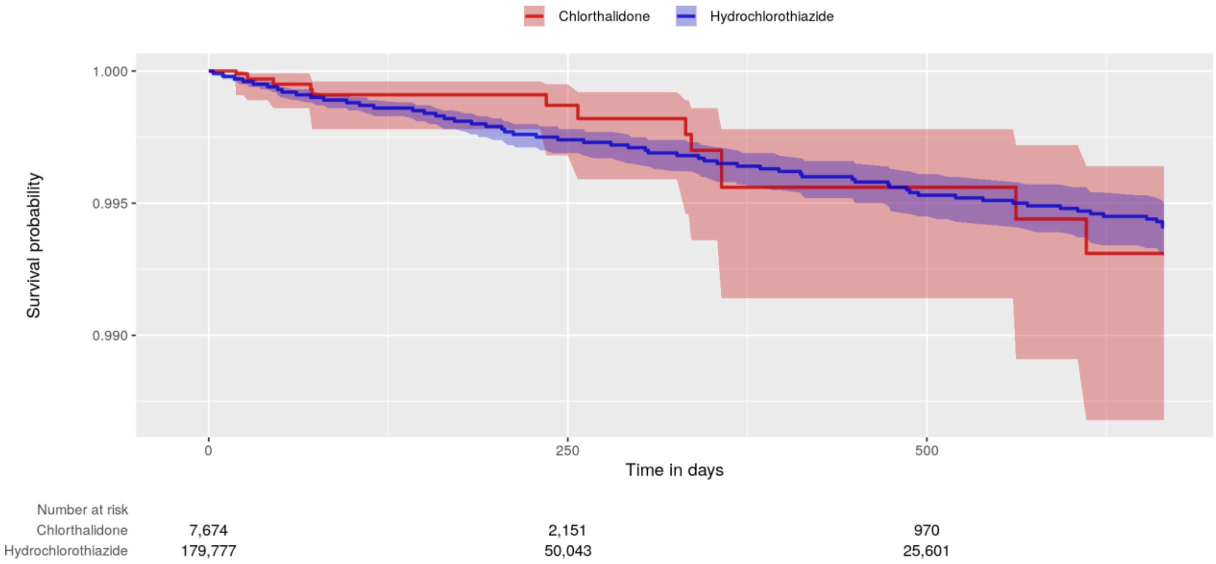

© 2020 Hripcsak G. et al. *JAMA Internal Medicine.*

Kaplan-Meier plot for acute myocardial infarction in Panther for matching on-treatment

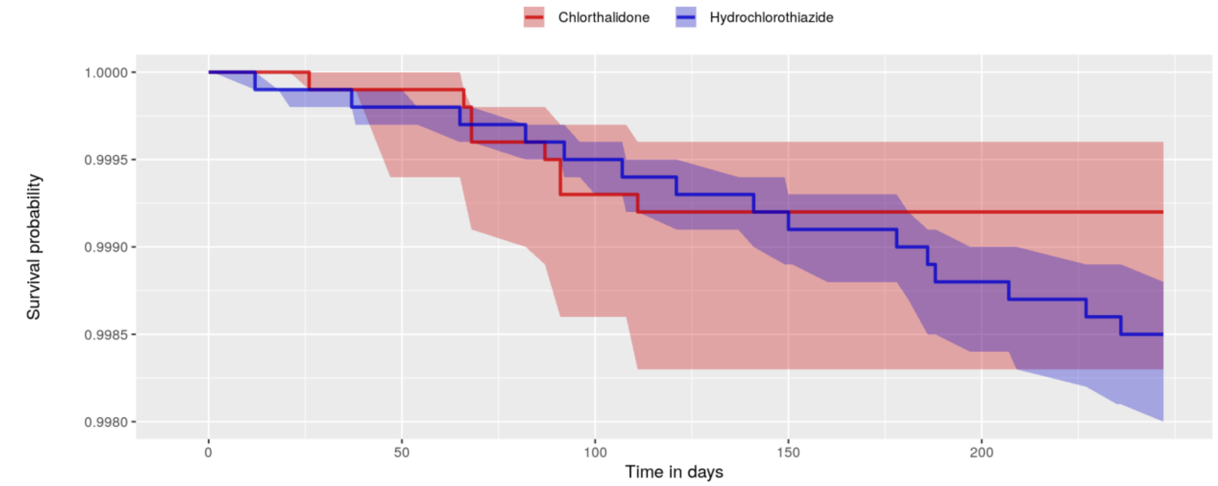

Number at risk					
Chlorthalidone	15,108	13,599	6,215	3,749	2,252
Hydrochlorothiazide	213,381	184,329	79,528	50,153	31,437

Kaplan-Meier plot for acute myocardial infarction in CCAE for matching intent-to-treat

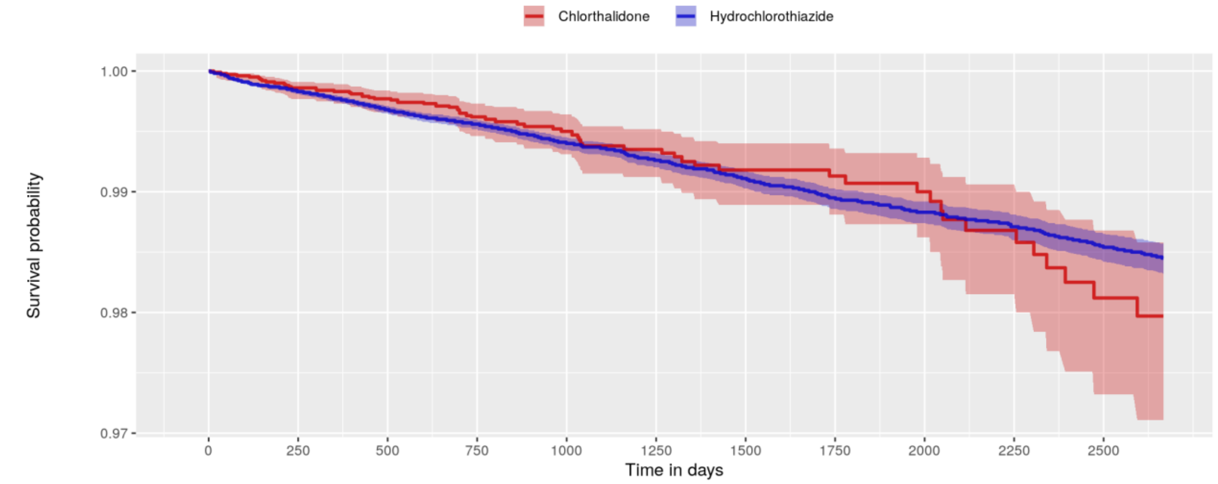

Number at risk											
Chlorthalidone	14,097	10,563	7,804	5,840	4,387	3,235	2,392	1,809	1,329	990	747
Hydrochlorothiazide	272,529	214,574	168,159	132,960	106,542	84,919	68,557	56,830	47,194	38,955	32,353

© 2020 Hripcsak G. et al. *JAMA Internal Medicine.*

Kaplan-Meier plot for acute myocardial infarction in Optum for matching intent-to-treat

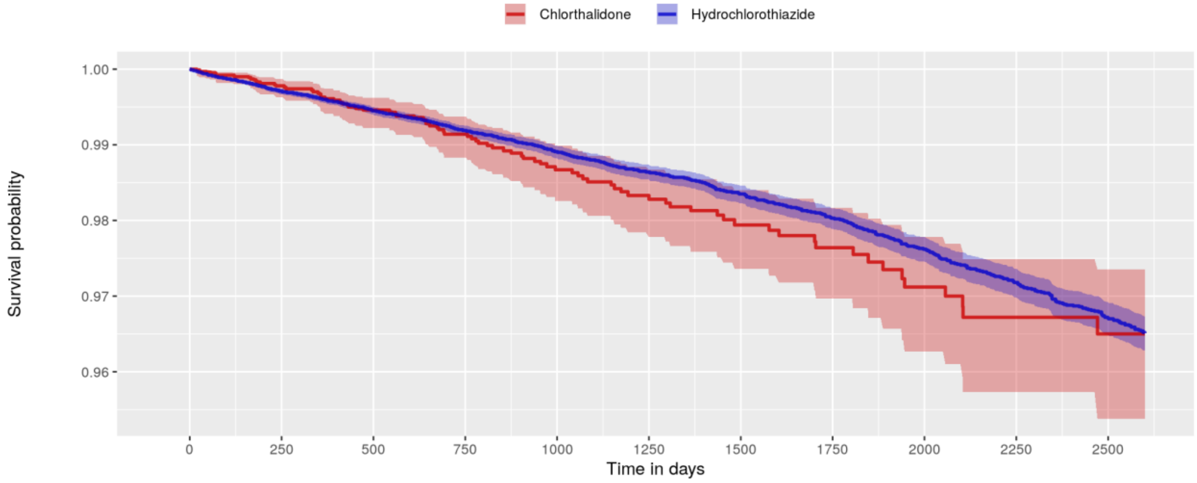

Number at risk											
Chlorthalidone	7,679	5,898	4,334	3,324	2,597	2,020	1,536	1,133	818	543	413
Hydrochlorothiazide	180,119	142,287	111,778	88,975	72,649	59,371	48,356	39,625	32,229	24,782	20,195

Kaplan-Meier plot for acute myocardial infarction in Panther for matching intent-to-treat

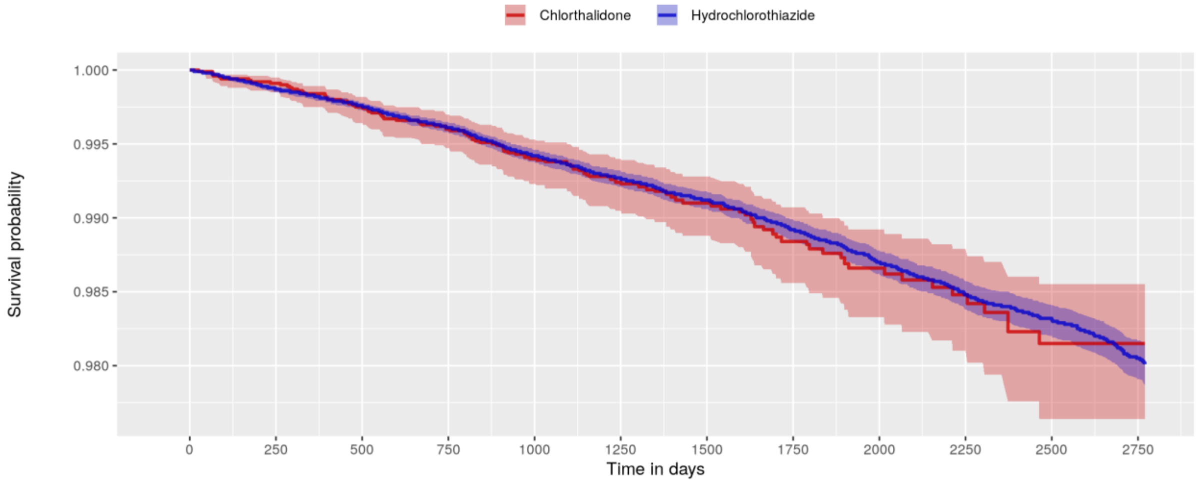

Number at risk												
Chlorthalidone	15,108	12,468	10,785	9,277	7,682	6,177	4,900	3,715	2,588	1,801	1,078	679
Hydrochlorothiazide	213,410	183,118	163,636	144,611	125,164	105,991	88,772	73,239	58,296	45,913	33,835	23,256

© 2020 Hripcsak G. et al. *JAMA Internal Medicine.*

Kaplan-Meier plot for hospitalized for heart failure in CCAE for stratified on-treatment

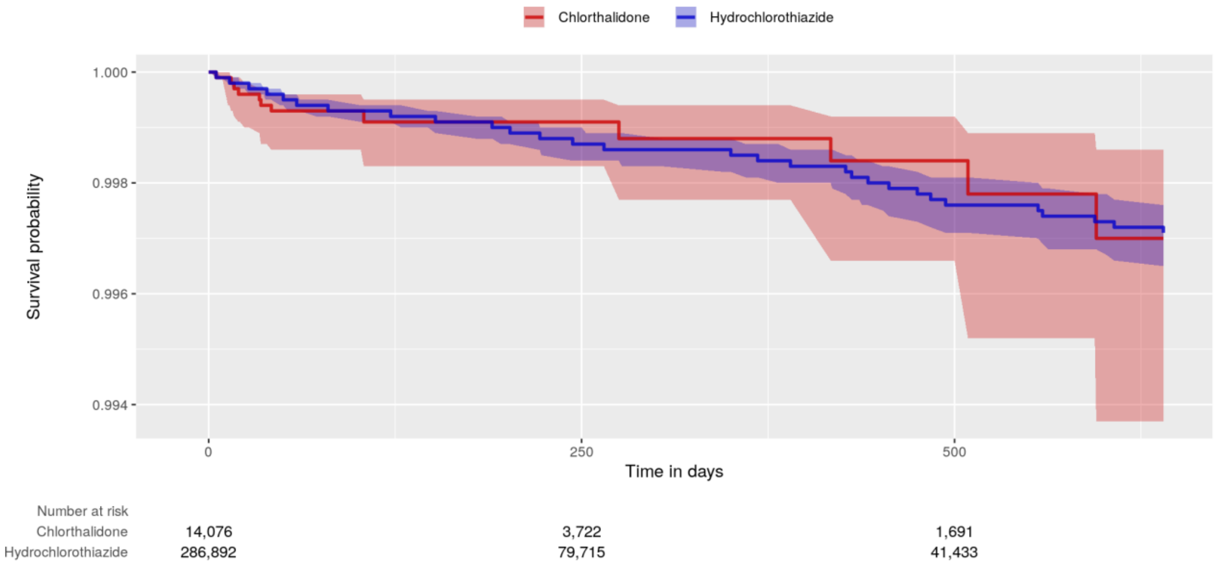

Kaplan-Meier plot for hospitalized for heart failure in Optum for stratified on-treatment

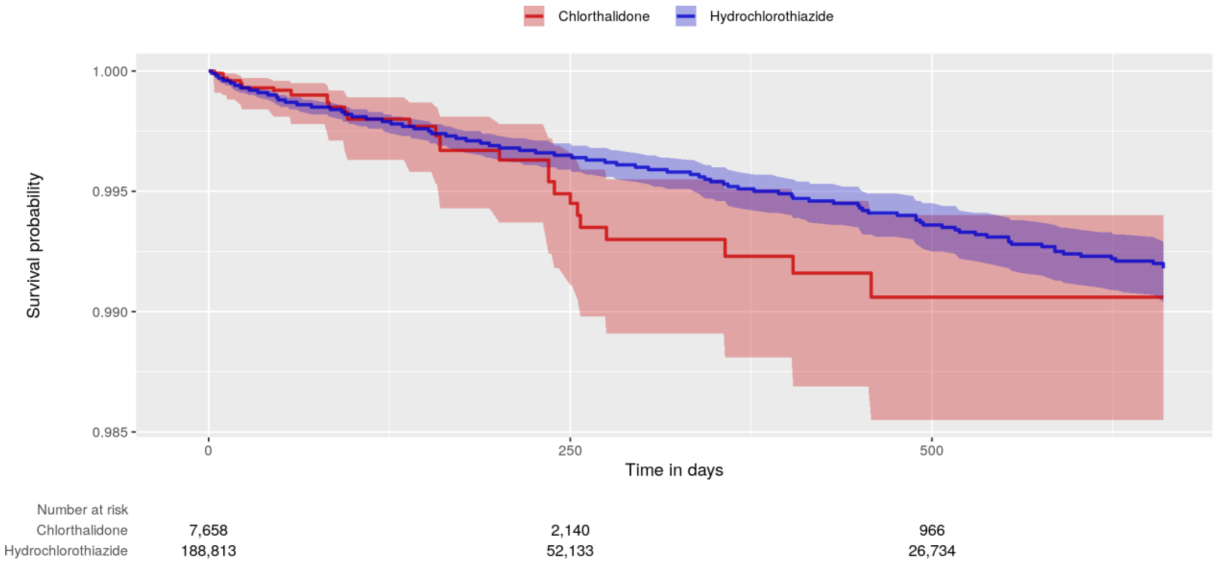

© 2020 Hripcsak G. et al. *JAMA Internal Medicine.*

Kaplan-Meier plot for hospitalized for heart failure in Panther for stratified on-treatment

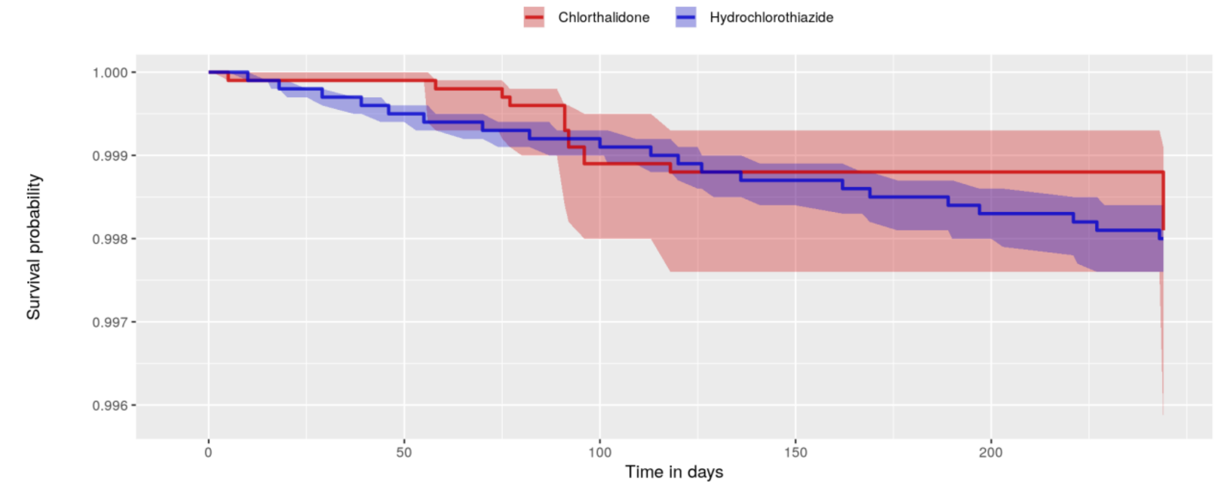

Number at risk					
Chlorthalidone	15,099	13,590	6,208	3,746	2,250
Hydrochlorothiazide	215,704	186,323	80,179	50,570	31,703

Kaplan-Meier plot for hospitalized for heart failure in CCAE for stratified intent-to-treat

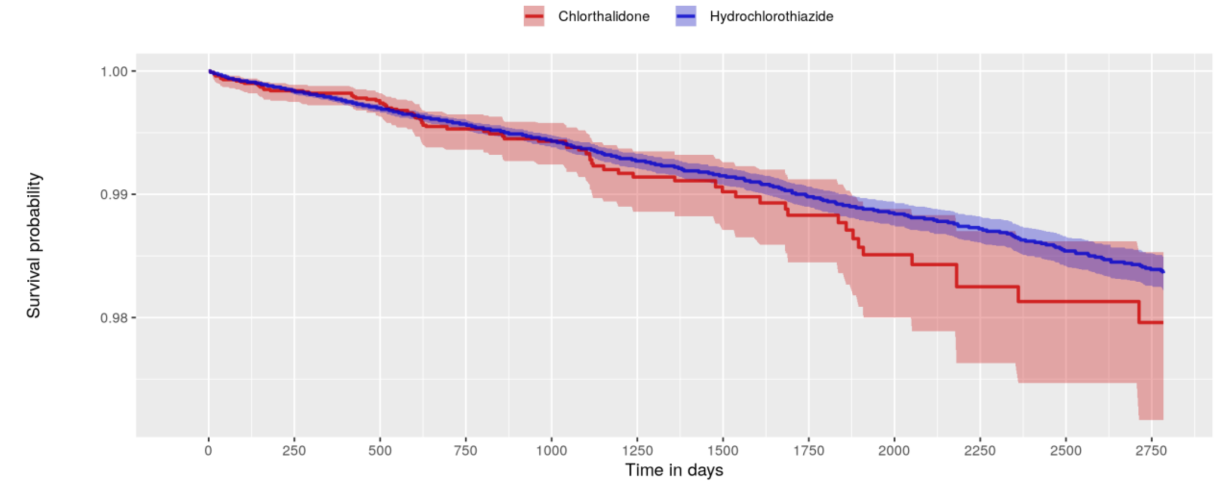

Number at risk												
Chlorthalidone	14,097	10,563	7,801	5,830	4,376	3,213	2,380	1,796	1,314	983	745	535
Hydrochlorothiazide	287,873	227,608	179,306	142,542	115,021	92,379	75,127	62,719	52,493	43,717	36,620	30,479

© 2020 Hripcsak G. et al. *JAMA Internal Medicine.*

Kaplan-Meier plot for hospitalized for heart failure in Optum for stratified intent-to-treat

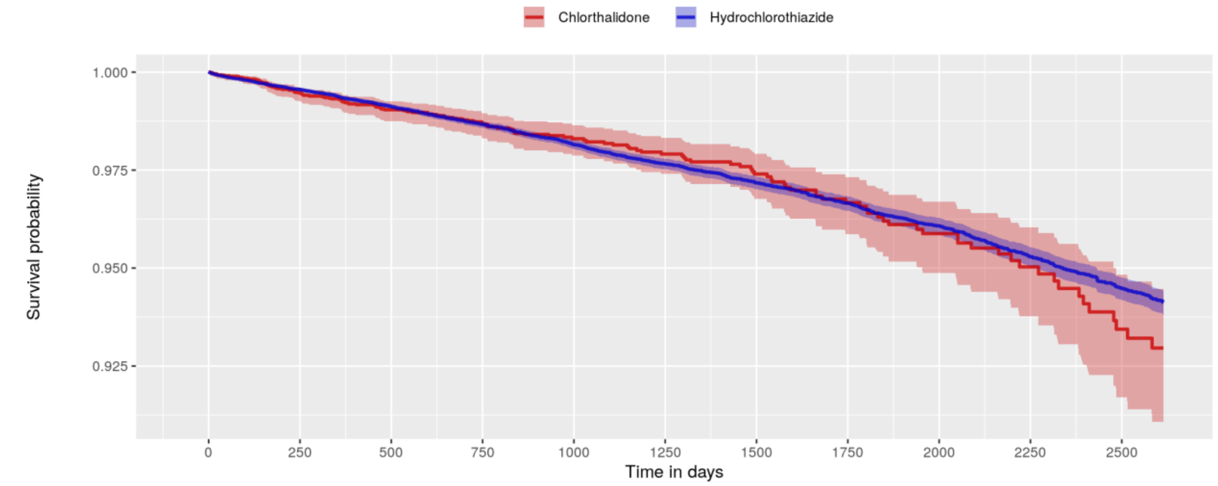

Number at risk											
Chlorthalidone	7,663	5,868	4,309	3,304	2,580	2,009	1,527	1,127	818	546	413
Hydrochlorothiazide	189,152	149,242	117,237	93,365	76,170	62,273	50,730	41,646	33,917	26,153	21,366

Kaplan-Meier plot for hospitalized for heart failure in Panther for stratified intent-to-treat

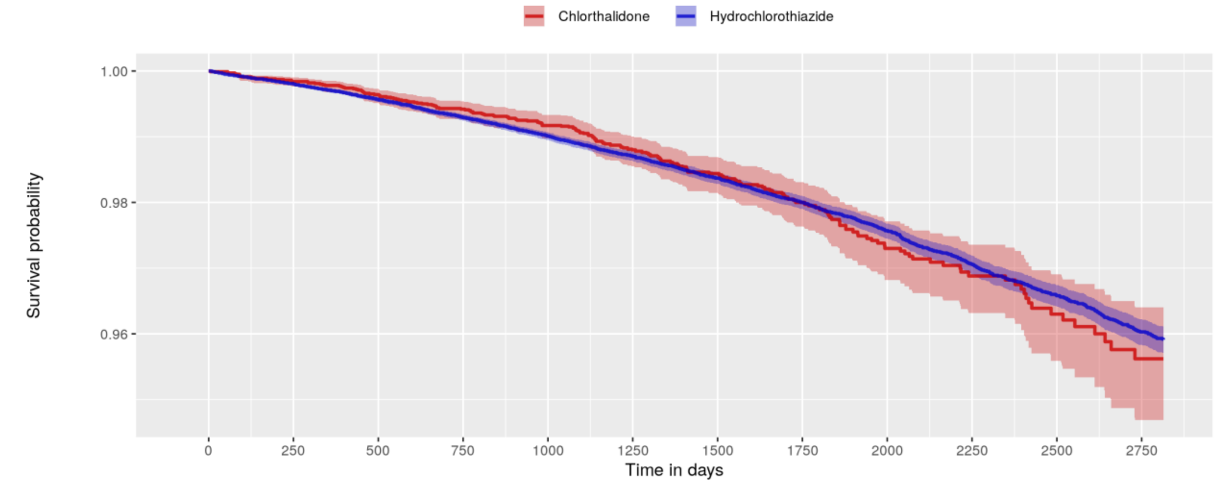

Number at risk												
Chlorthalidone	15,099	12,450	10,769	9,257	7,665	6,144	4,864	3,677	2,546	1,766	1,057	662
Hydrochlorothiazide	215,732	185,258	165,565	146,394	126,886	107,614	90,311	74,752	59,824	47,414	35,333	24,734

© 2020 Hripcsak G. et al. *JAMA Internal Medicine.*

Kaplan-Meier plot for hospitalized for heart failure in CCAE for matching on-treatment

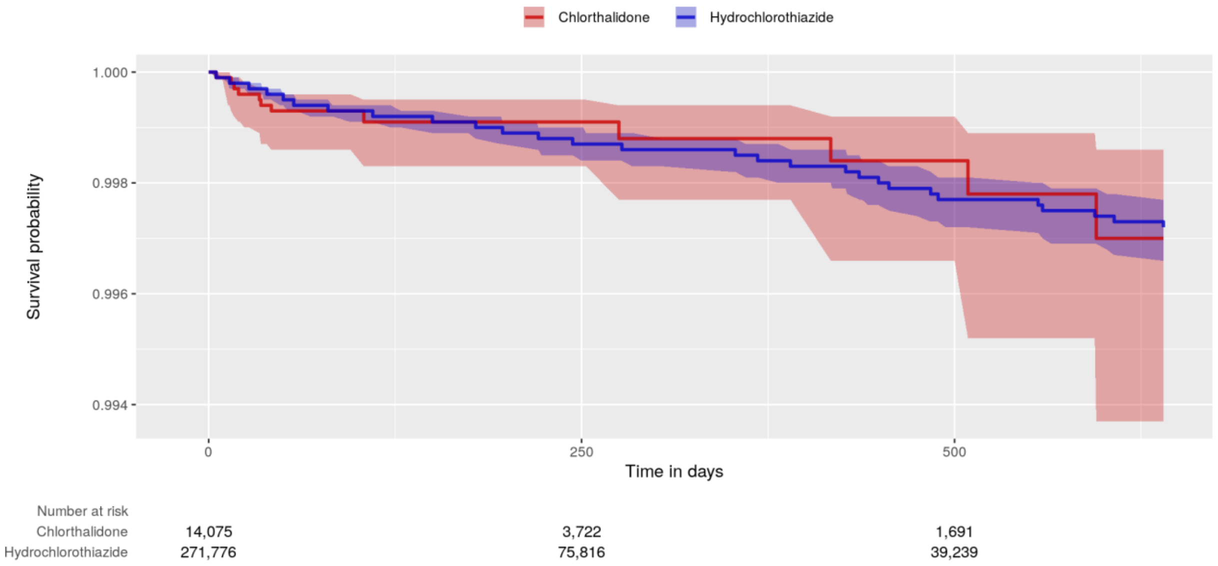

Kaplan-Meier plot for hospitalized for heart failure in Optum for matching on-treatment

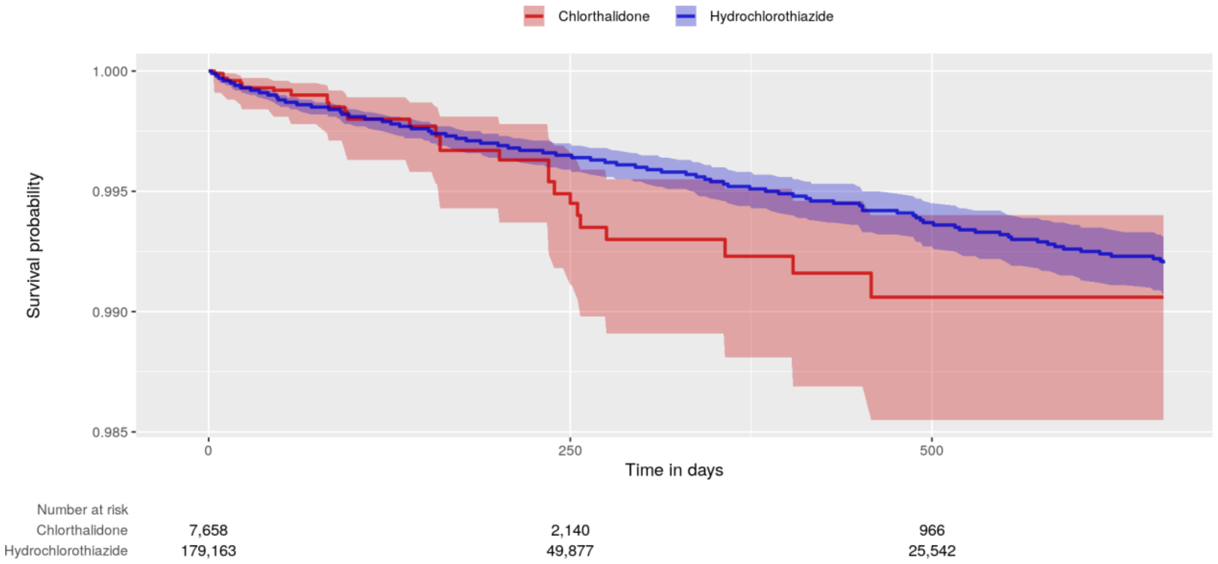

© 2020 Hripcsak G. et al. *JAMA Internal Medicine.*

Kaplan-Meier plot for hospitalized for heart failure in Panther for matching on-treatment

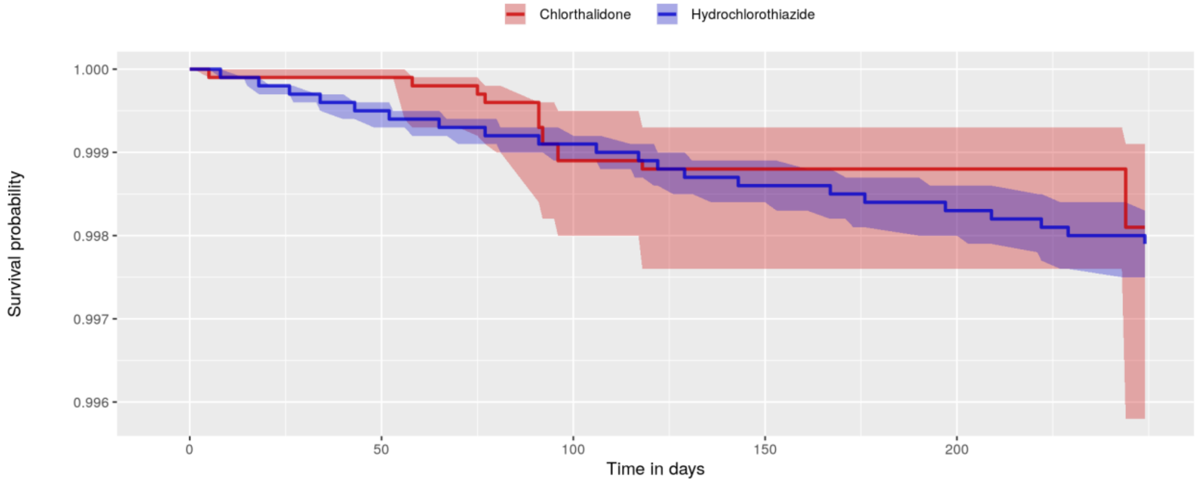

Number at risk					
Chlorthalidone	15,099	13,590	6,208	3,746	2,250
Hydrochlorothiazide	213,063	184,013	79,367	50,041	31,375

Kaplan-Meier plot for hospitalized for heart failure in CCAE for matching intent-to-treat

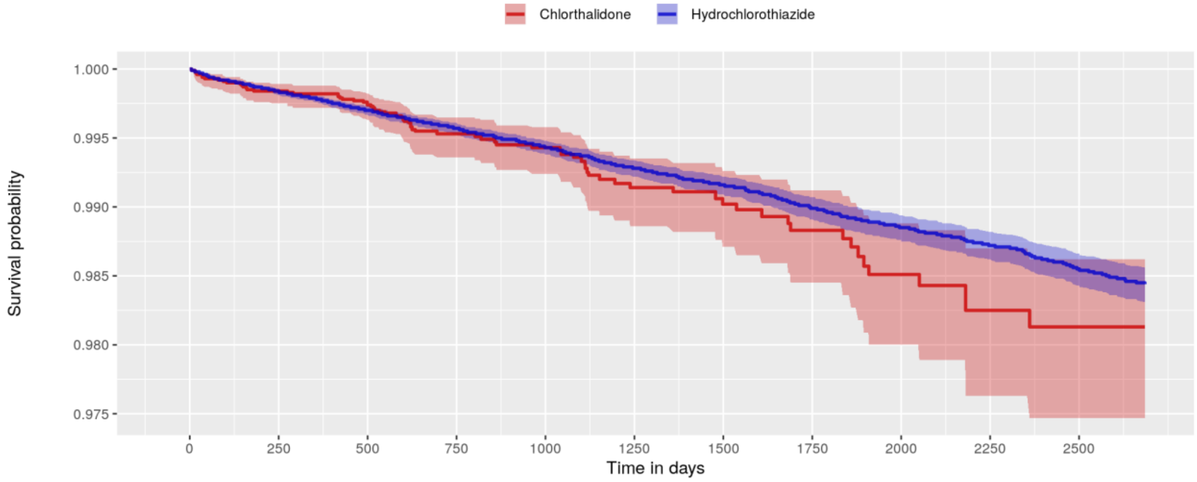

Number at risk											
Chlorthalidone	14,096	10,563	7,801	5,830	4,376	3,213	2,380	1,796	1,314	983	745
Hydrochlorothiazide	272,384	214,529	168,185	132,994	106,606	84,998	68,639	56,895	47,261	39,021	32,432

© 2020 Hripcsak G. et al. *JAMA Internal Medicine.*

Kaplan-Meier plot for hospitalized for heart failure in Optum for matching intent-to-treat

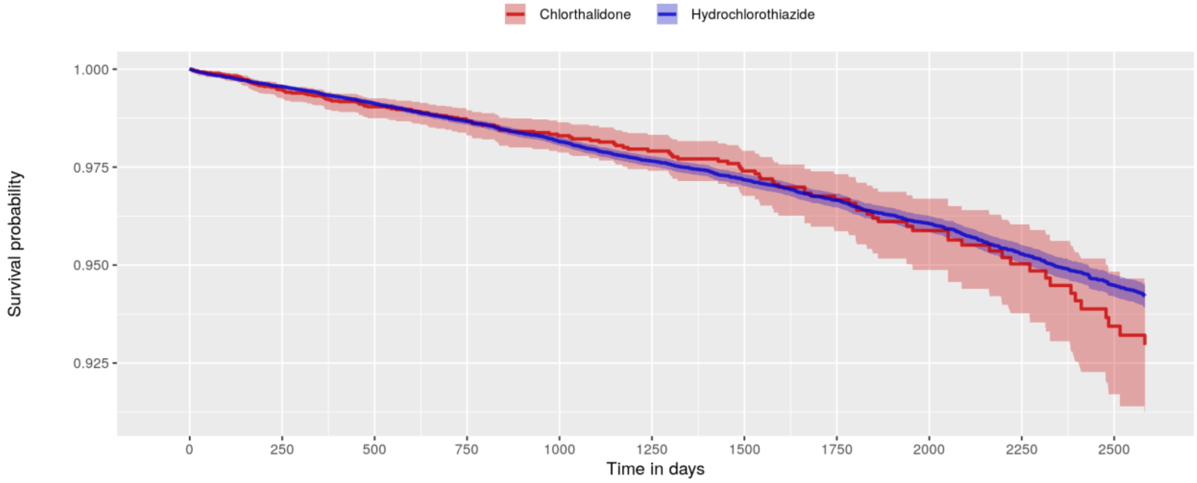

Number at risk											
Chlorthalidone	7,663	5,868	4,309	3,304	2,580	2,009	1,527	1,127	818	546	413
Hydrochlorothiazide	179,508	141,668	111,129	88,345	71,983	58,729	47,745	39,060	31,724	24,339	19,793

Kaplan-Meier plot for hospitalized for heart failure in Panther for matching intent-to-treat

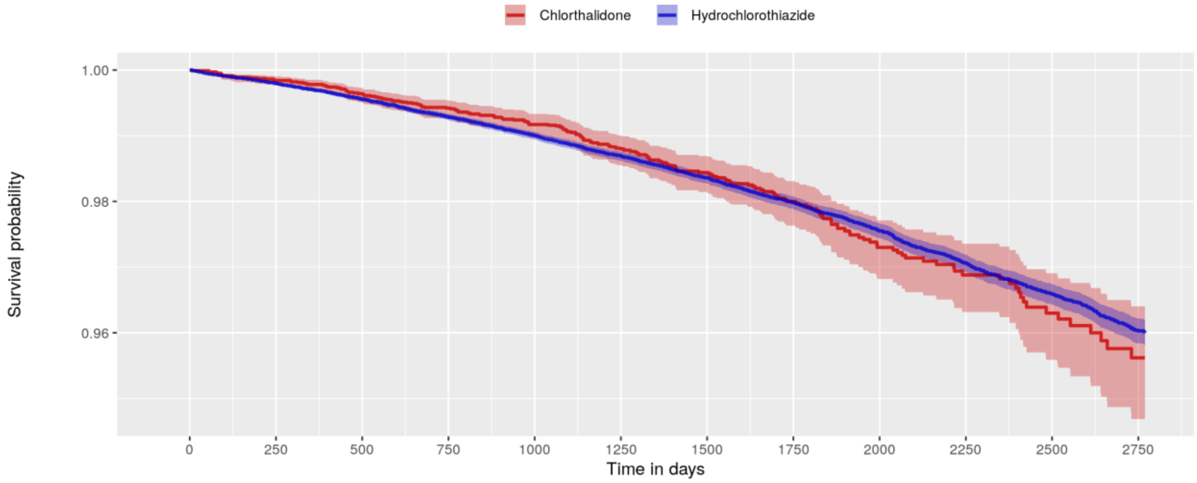

Number at risk												
Chlorthalidone	15,099	12,450	10,769	9,257	7,665	6,144	4,864	3,677	2,546	1,766	1,057	662
Hydrochlorothiazide	213,091	182,685	163,045	143,912	124,454	105,230	87,987	72,486	57,628	45,290	33,341	22,907

© 2020 Hripcsak G. et al. *JAMA Internal Medicine.*

Kaplan-Meier plot for stroke in CCAE for stratified on-treatment

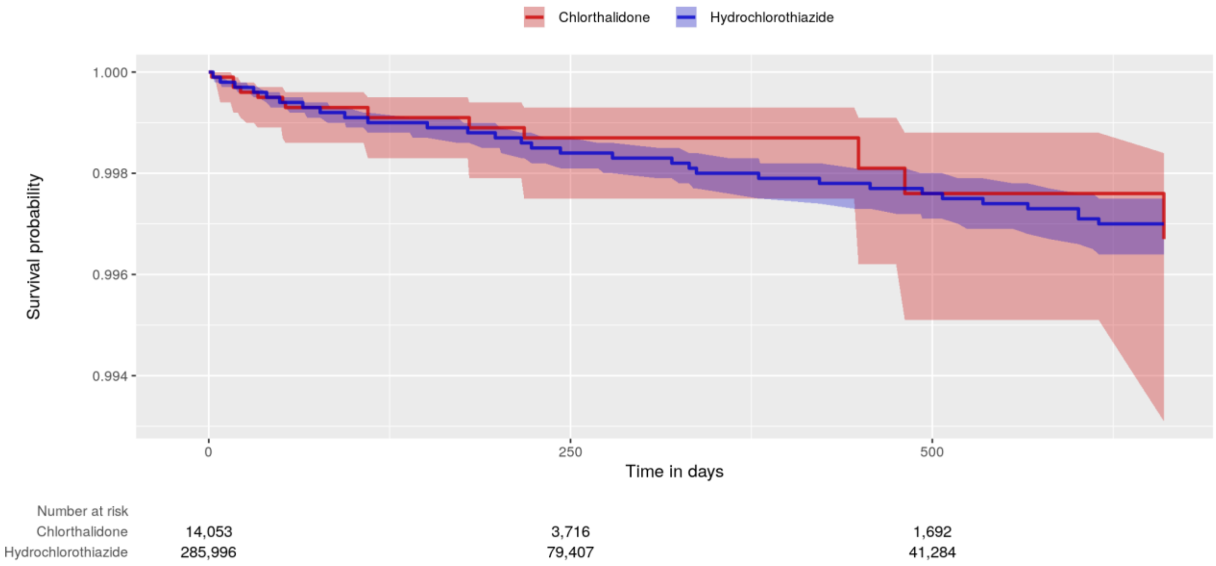

Kaplan-Meier plot for stroke in Optum for stratified on-treatment

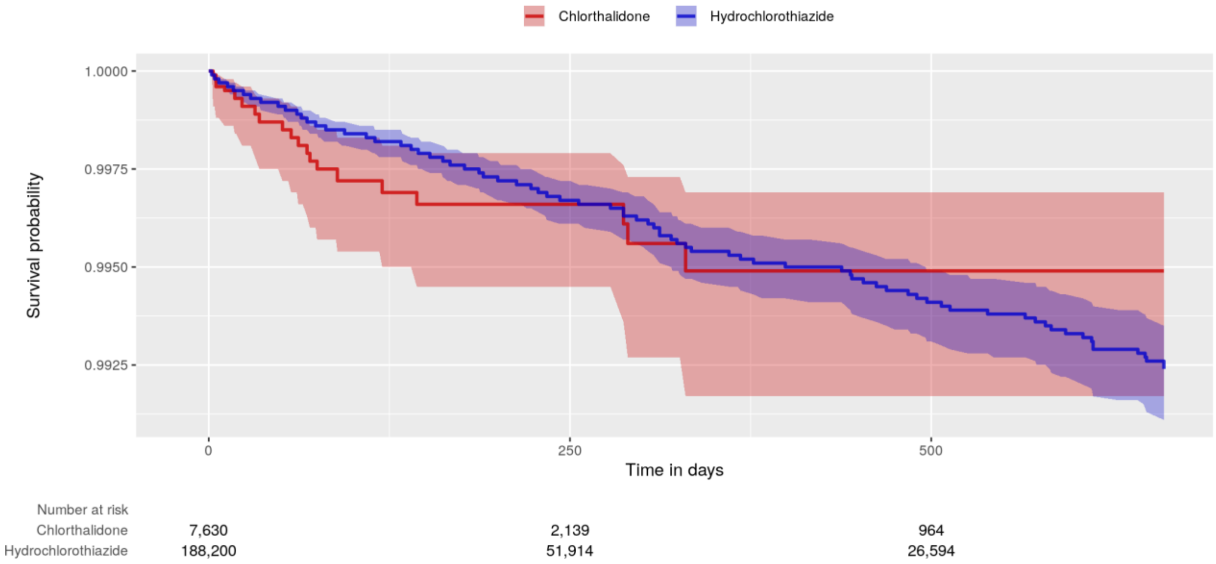

© 2020 Hripcsak G. et al. *JAMA Internal Medicine.*

Kaplan-Meier plot for stroke in Panther for stratified on-treatment

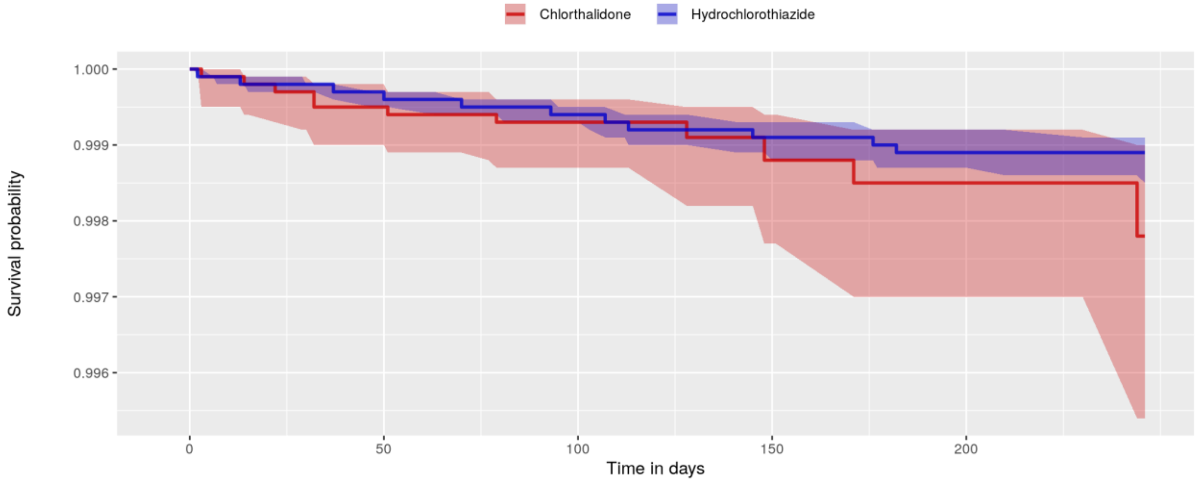

Number at risk					
Chlorthalidone	15,072	13,561	6,198	3,740	2,242
Hydrochlorothiazide	215,502	186,194	80,135	50,556	31,690

Kaplan-Meier plot for stroke in CCAE for stratified intent-to-treat

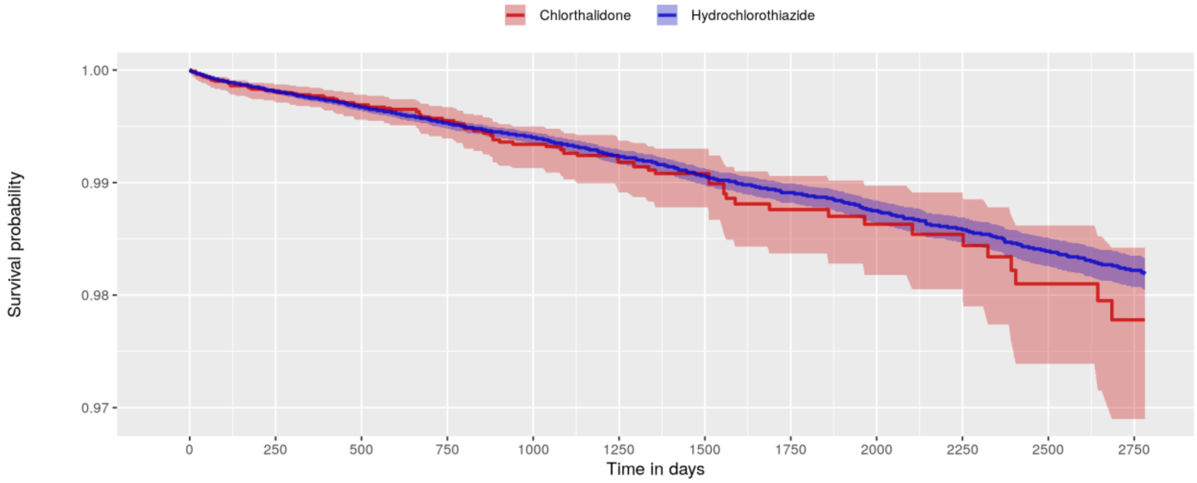

Number at risk												
Chlorthalidone	14,074	10,545	7,795	5,831	4,373	3,213	2,384	1,801	1,321	990	752	542
Hydrochlorothiazide	286,975	226,907	178,795	142,118	114,702	92,115	74,911	62,570	52,344	43,593	36,513	30,388

© 2020 Hripcsak G. et al. *JAMA Internal Medicine.*

Kaplan-Meier plot for stroke in Optum for stratified intent-to-treat

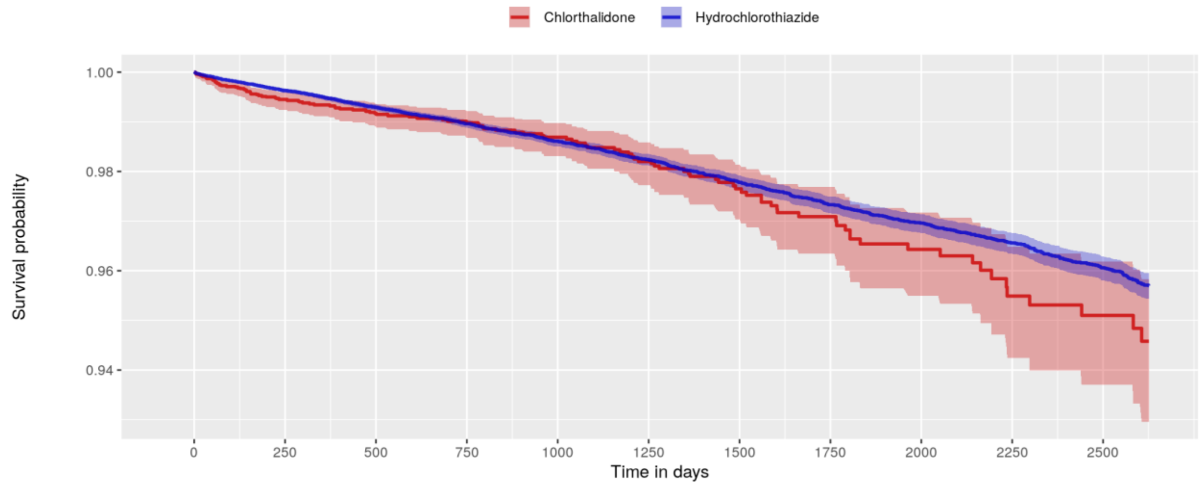

Number at risk											
Chlorthalidone	7,635	5,843	4,306	3,307	2,582	2,011	1,523	1,123	811	538	413
Hydrochlorothiazide	188,543	148,851	117,036	93,246	76,205	62,363	50,815	41,734	34,064	26,298	21,530

Kaplan-Meier plot for stroke in Panther for stratified intent-to-treat

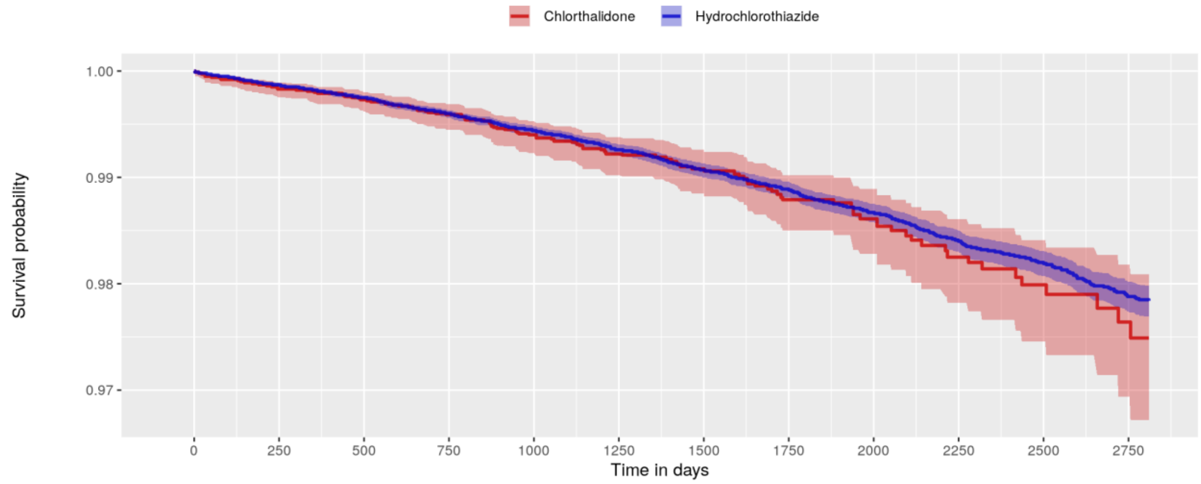

Number at risk											
Chlorthalidone	15,072	12,427	10,755	9,251	7,664	6,160	4,880	3,698	2,574	1,784	1,074	673
Hydrochlorothiazide	215,529	185,236	165,749	146,707	127,270	108,045	90,737	75,235	60,254	47,827	35,658	24,980

© 2020 Hripcsak G. et al. *JAMA Internal Medicine.*

Kaplan-Meier plot for stroke in CCAE for matching on-treatment

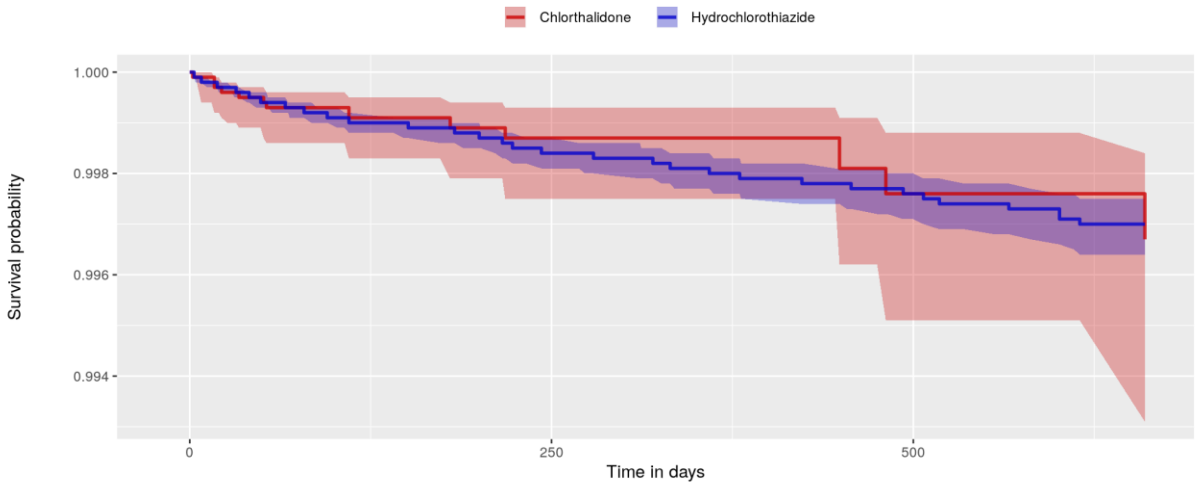

Number at risk			
Chlorthalidone	14,052	3,716	1,692
Hydrochlorothiazide	270,560	75,408	39,030

Kaplan-Meier plot for stroke in Optum for matching on-treatment

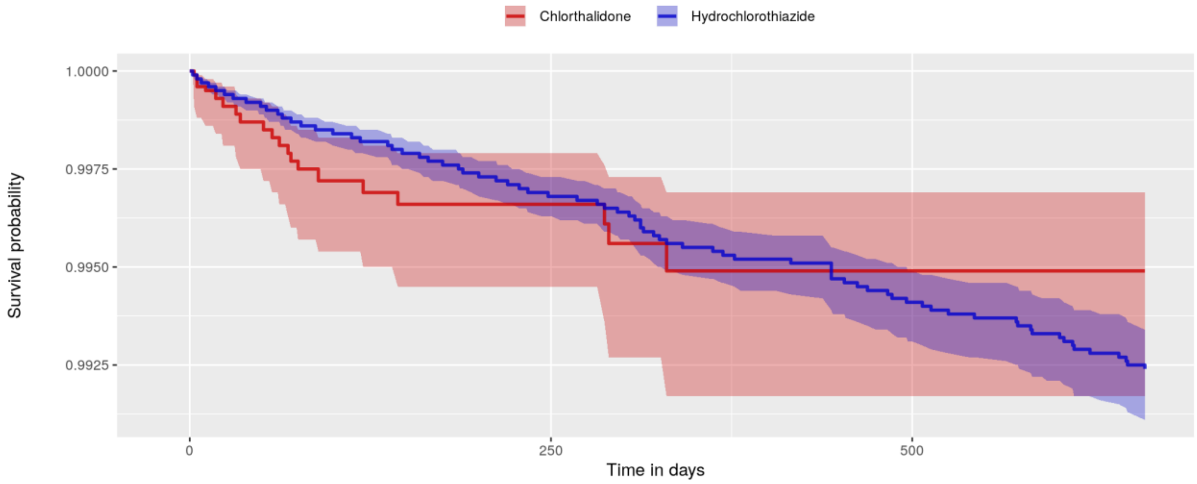

Number at risk			
Chlorthalidone	7,629	2,138	964
Hydrochlorothiazide	178,742	49,702	25,424

© 2020 Hripcsak G. et al. *JAMA Internal Medicine.*

Kaplan-Meier plot for stroke in Panther for matching on-treatment

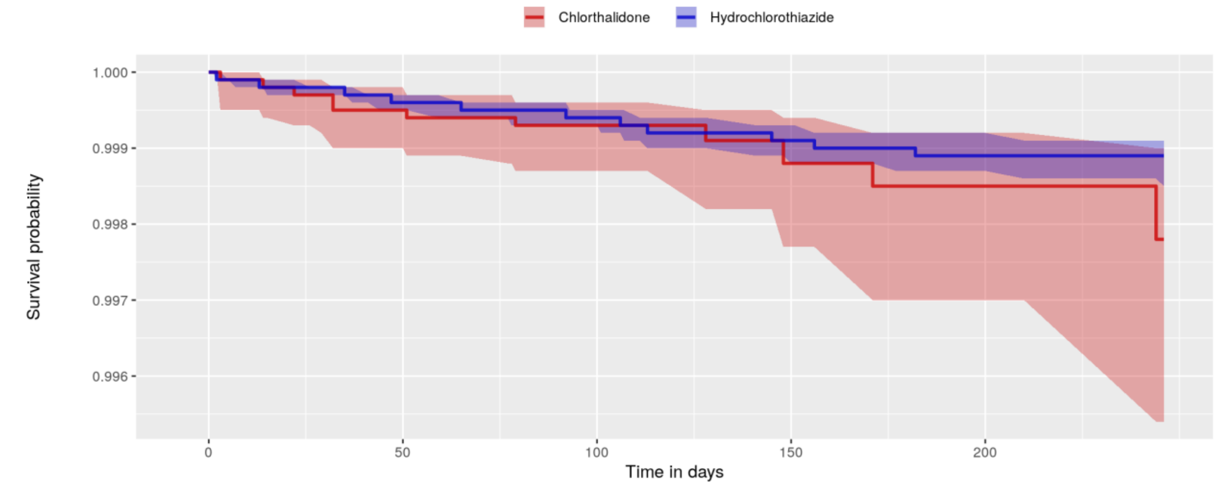

Number at risk					
Chlorthalidone	15,072	13,561	6,198	3,740	2,242
Hydrochlorothiazide	212,963	183,972	79,360	50,050	31,373

Kaplan-Meier plot for stroke in CCAE for matching intent-to-treat

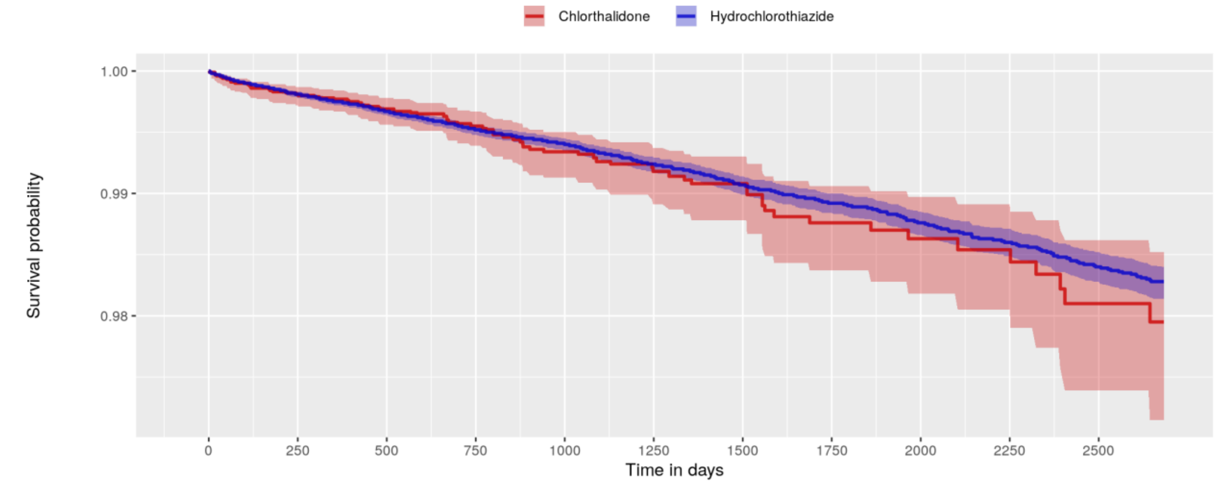

Number at risk											
Chlorthalidone	14,073	10,545	7,795	5,831	4,373	3,213	2,384	1,801	1,321	990	752
Hydrochlorothiazide	271,167	213,549	167,429	132,370	106,088	84,563	68,271	56,613	46,986	38,792	32,229

© 2020 Hripcsak G. et al. *JAMA Internal Medicine.*

Kaplan-Meier plot for stroke in Optum for matching intent-to-treat

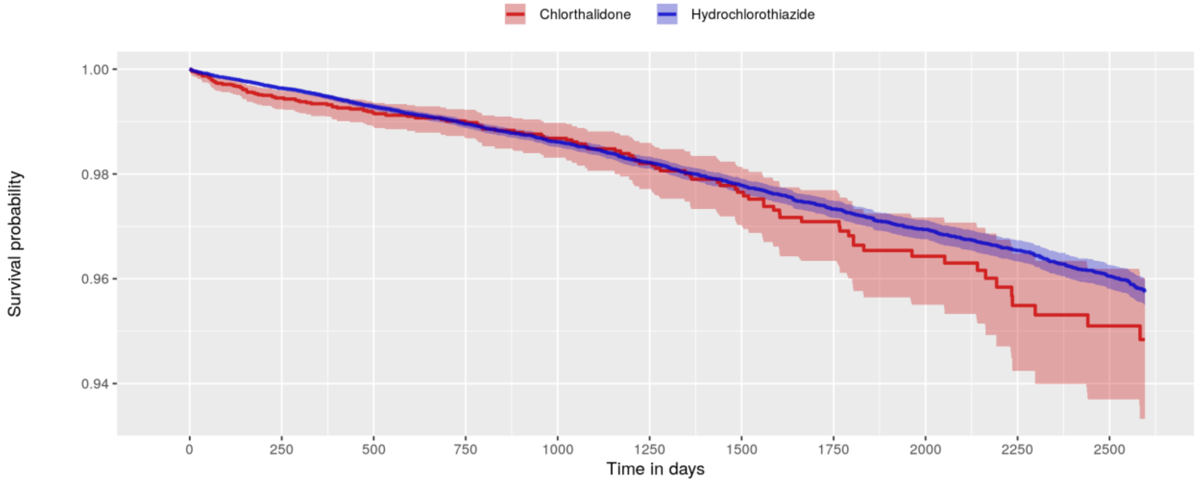

Number at risk											
Chlorthalidone	7,634	5,842	4,306	3,307	2,582	2,011	1,523	1,123	811	538	413
Hydrochlorothiazide	179,085	141,425	111,030	88,310	72,083	58,869	47,879	39,190	31,895	24,503	19,966

Kaplan-Meier plot for stroke in Panther for matching intent-to-treat

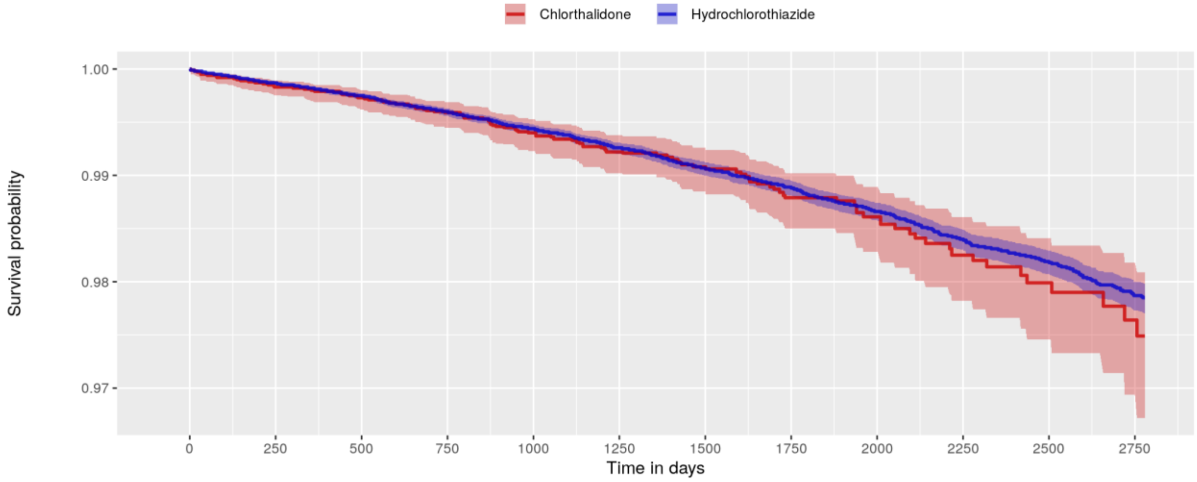

Number at risk												
Chlorthalidone	15,072	12,427	10,755	9,251	7,664	6,160	4,880	3,698	2,574	1,784	1,074	673
Hydrochlorothiazide	212,990	182,765	163,322	144,319	124,927	105,751	88,497	73,050	58,133	45,771	33,724	23,207

© 2020 Hripcsak G. et al. *JAMA Internal Medicine.*

Kaplan-Meier plot for hypokalemia in CCAE for stratified on-treatment

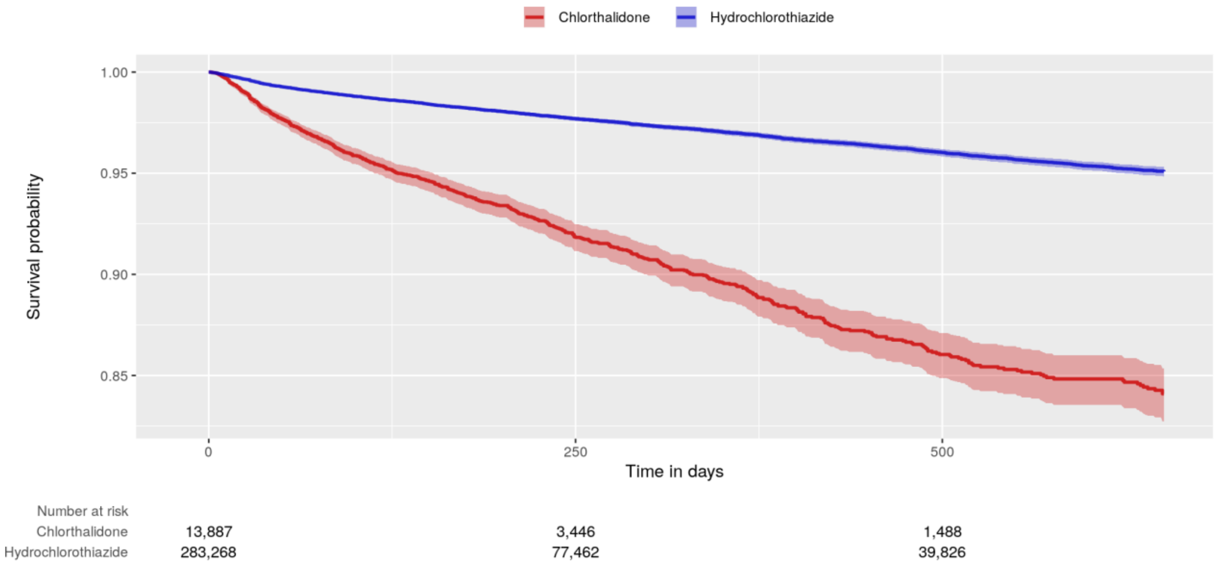

Kaplan-Meier plot for hypokalemia in Optum for stratified on-treatment

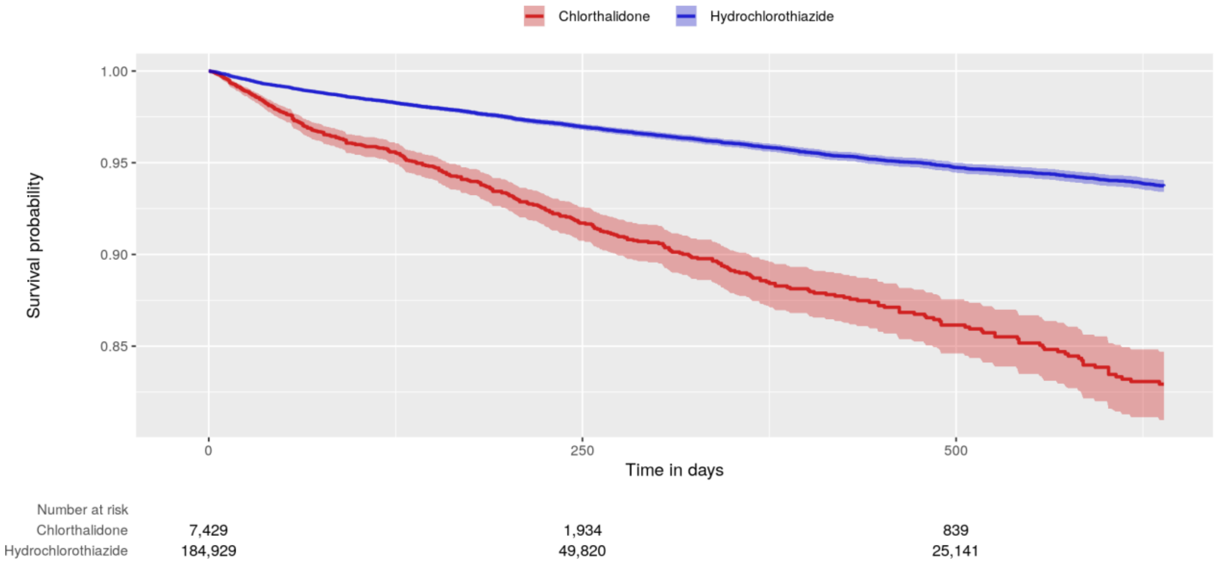

© 2020 Hripcsak G. et al. *JAMA Internal Medicine.*

Kaplan-Meier plot for hypokalemia in Panther for stratified on-treatment

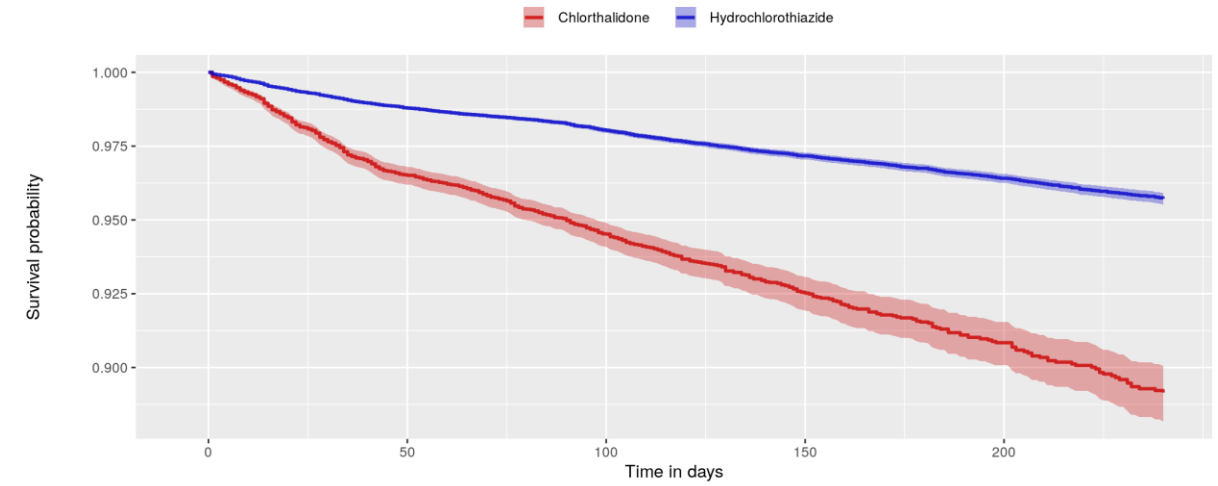

Number at risk					
Chlorthalidone	14,443	12,574	5,590	3,302	1,940
Hydrochlorothiazide	208,745	178,306	76,071	47,484	29,463

Kaplan-Meier plot for hypokalemia in CCAE for stratified intent-to-treat

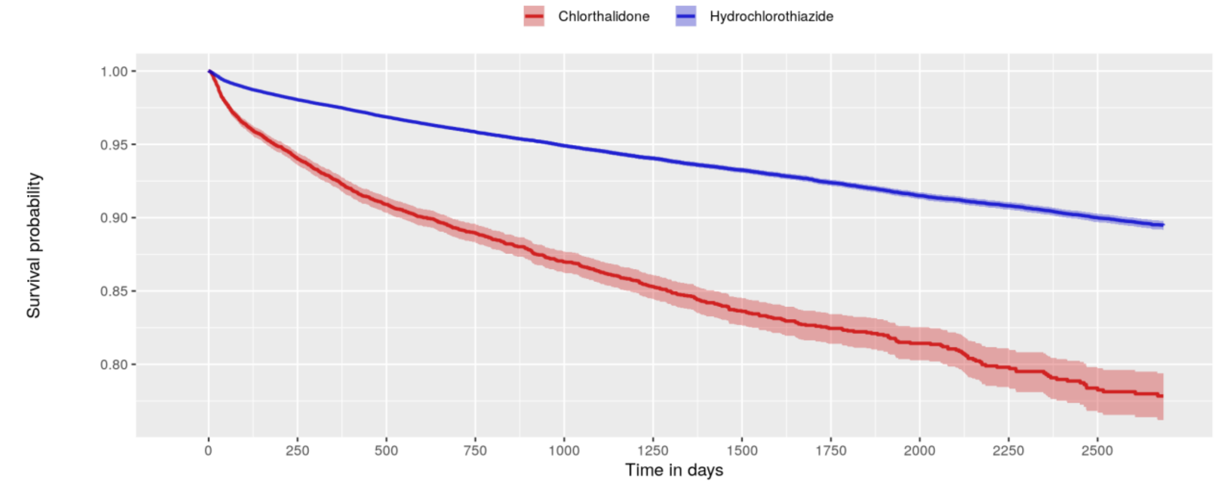

Number at risk											
Chlorthalidone	13,906	9,824	7,034	5,175	3,828	2,778	2,040	1,535	1,131	840	635
Hydrochlorothiazide	284,237	221,178	172,834	136,422	109,401	87,410	70,772	58,815	48,929	40,564	33,793

© 2020 Hripcsak G. et al. *JAMA Internal Medicine.*

Kaplan-Meier plot for hypokalemia in Optum for stratified intent-to-treat

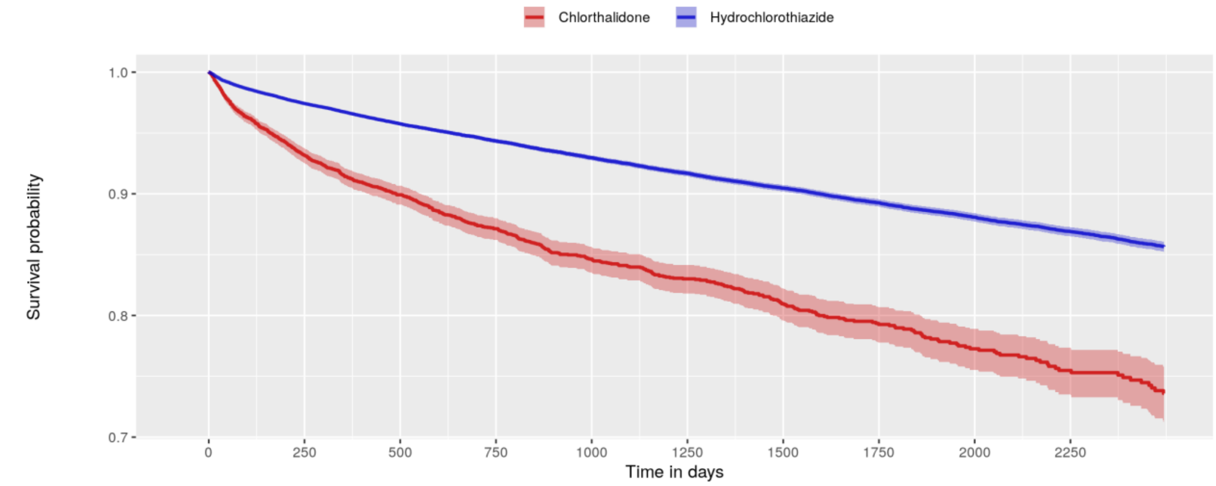

Number at risk										
Chlorthalidone	7,433	5,326	3,796	2,824	2,158	1,655	1,227	890	637	420
Hydrochlorothiazide	185,266	143,056	111,065	87,440	70,543	57,099	46,062	37,431	30,212	23,237

Kaplan-Meier plot for hypokalemia in Panther for stratified intent-to-treat

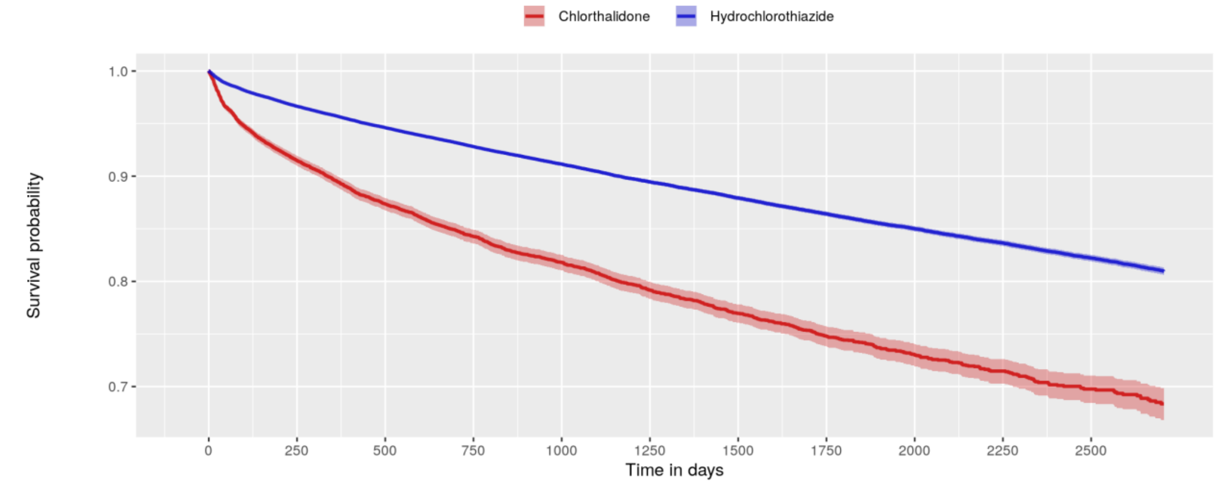

Number at risk											
Chlorthalidone	14,443	10,952	9,056	7,527	6,070	4,742	3,641	2,681	1,810	1,230	737
Hydrochlorothiazide	208,773	174,127	152,781	132,921	113,533	94,931	78,651	64,357	51,004	40,074	29,505

© 2020 Hripcsak G. et al. *JAMA Internal Medicine.*

Kaplan-Meier plot for hypokalemia in CCAE for matching on-treatment

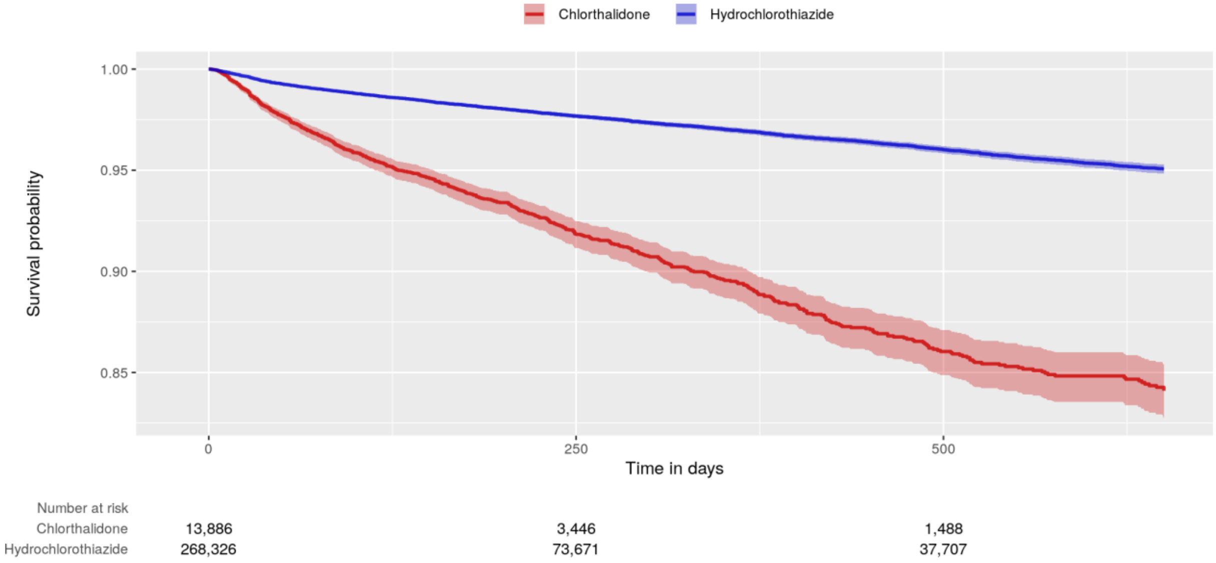

Kaplan-Meier plot for hypokalemia in Optum for matching on-treatment

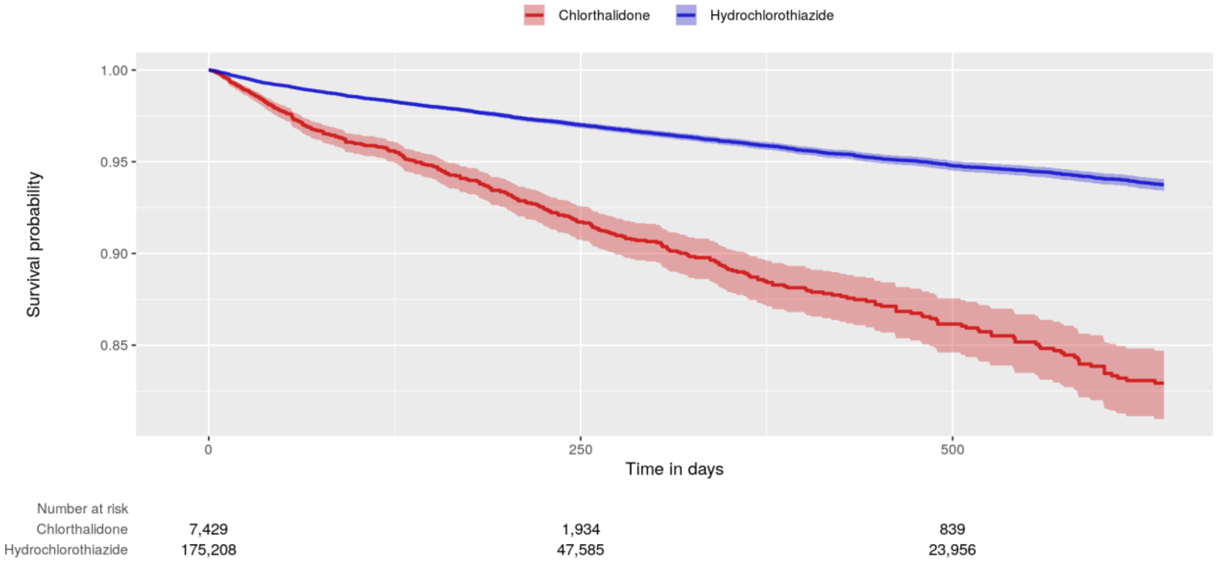

© 2020 Hripcsak G. et al. *JAMA Internal Medicine.*

Kaplan-Meier plot for hypokalemia in Panther for matching on-treatment

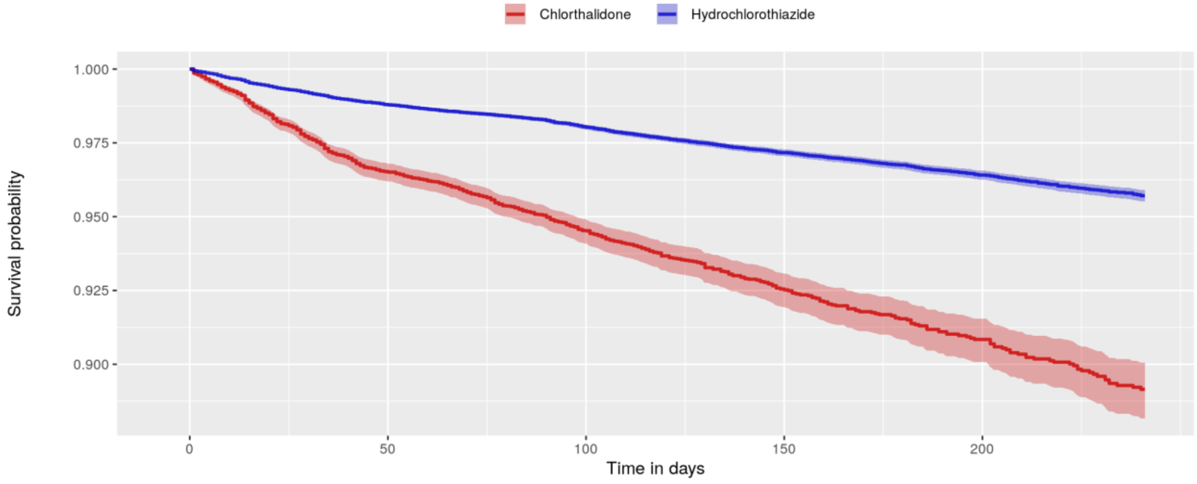

Number at risk					
Chlorthalidone	14,443	12,574	5,590	3,302	1,940
Hydrochlorothiazide	206,276	176,163	75,331	47,007	29,165

Kaplan-Meier plot for hypokalemia in CCAE for matching intent-to-treat

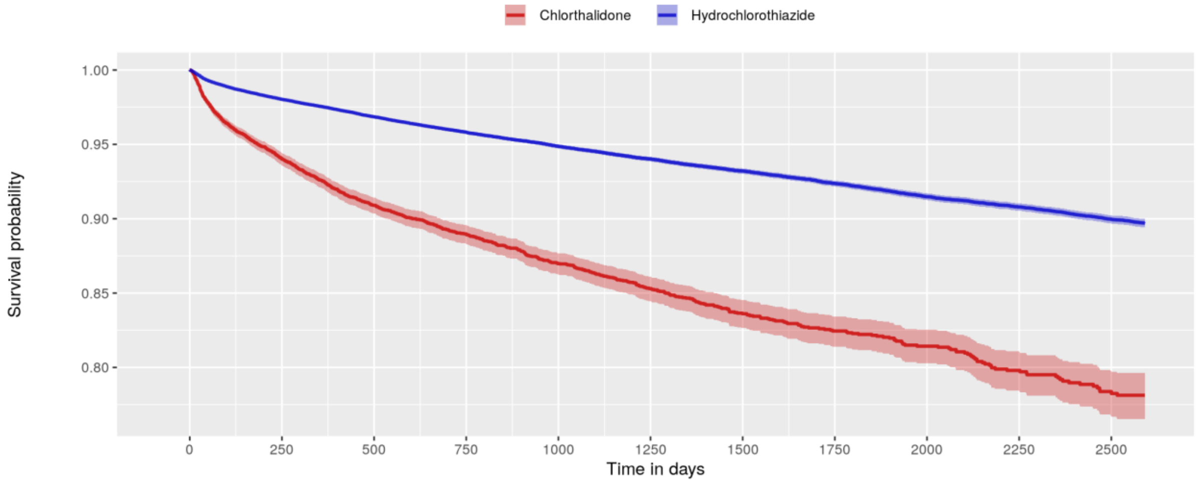

Number at risk											
Chlorthalidone	13,905	9,824	7,034	5,175	3,828	2,778	2,040	1,535	1,131	840	635
Hydrochlorothiazide	268,911	208,415	162,087	127,254	101,356	80,395	64,633	53,330	44,035	36,199	29,928

© 2020 Hripcsak G. et al. *JAMA Internal Medicine.*

Kaplan-Meier plot for hypokalemia in Optum for matching intent-to-treat

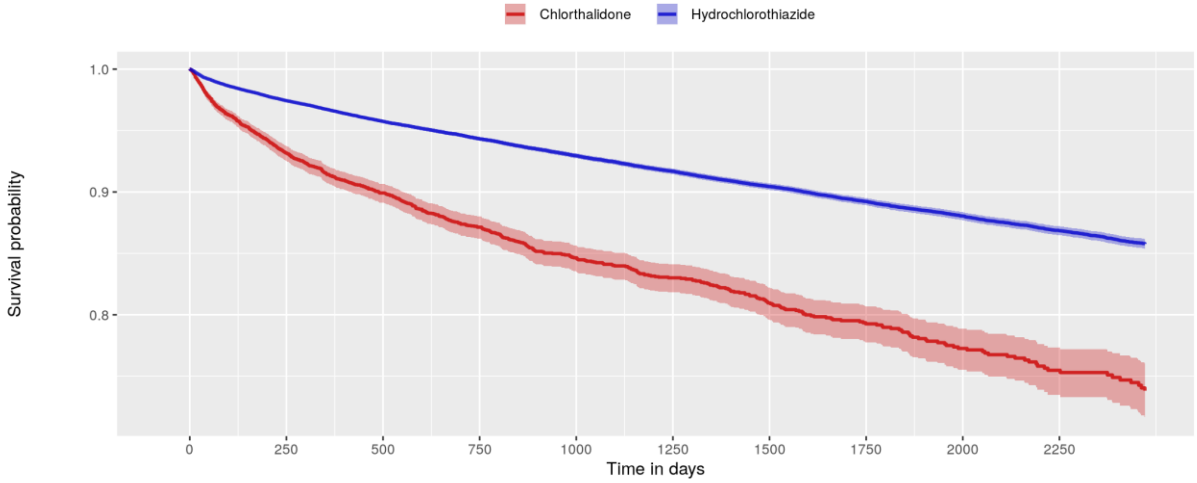

Number at risk										
Chlorthalidone	7,433	5,326	3,796	2,824	2,158	1,655	1,227	890	637	420
Hydrochlorothiazide	175,555	135,605	105,121	82,598	66,563	53,776	43,306	35,083	28,231	21,616

Kaplan-Meier plot for hypokalemia in Panther for matching intent-to-treat

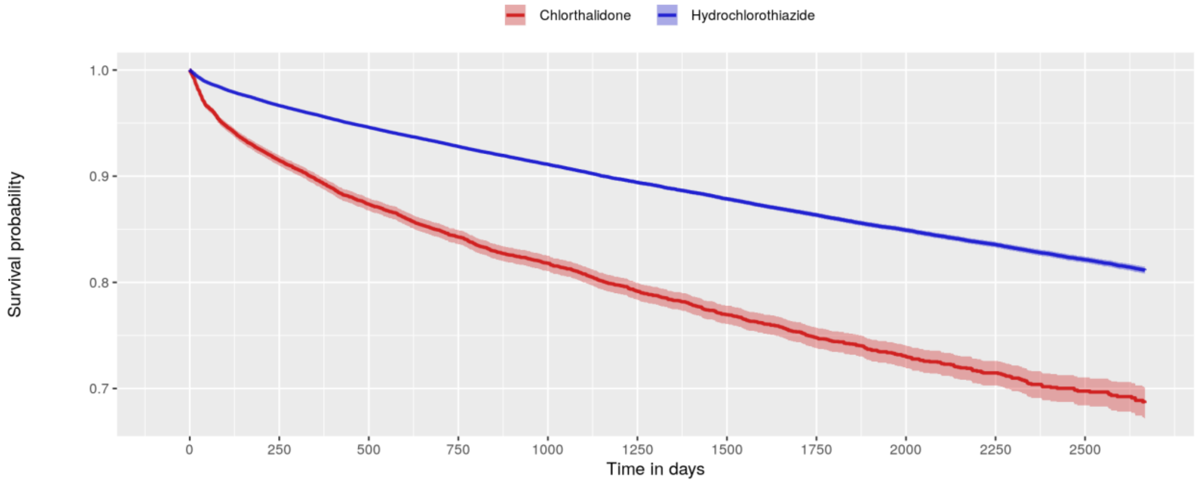

Number at risk											
Chlorthalidone	14,443	10,952	9,056	7,527	6,070	4,742	3,641	2,681	1,810	1,230	737
Hydrochlorothiazide	206,304	171,765	150,514	130,726	111,402	92,888	76,703	62,484	49,214	38,361	27,930

© 2020 Hripcsak G. et al. *JAMA Internal Medicine.*

3 Detailed definitions of exposures and outcomes

We provide here a formal definition for each exposure and outcome. The definitions are based on the OHDSI OMOP data model [24]. They contain logic and concept set expressions, which are lists of codes used to query the database. Each concept set expression is defined in one or more tables below each definition. In each table, the "concept ID" is the unique OHDSI OMOP code for the term, the "domain" tells what kind of data are coded, and the "vocabulary" and "concept name" identify the meaning of the code. If the term is an exclusion for the outcome (include only persons without that code), then "excluded" is set to "yes." If "descendants" is "yes," then all the terms in that vocabulary that are more specific (i.e., descendants) are included in the query. "Mapped" implies that terms from other vocabularies are included. OHDSI maps data from existing vocabularies like ICD9-CM and ICD10-CM to its standard vocabularies like SNOMED CT, so diagnoses in queries are defined by with SNOMED CT codes rather than ICD codes.

For the effectiveness and safety outcomes, we also include a table with a logical description, computer-executable code, and relevant references.

3.1 Drug exposures

In this study, we are interested in two exposures, chlorthalidone and hydrochlorothiazide. They were formally defined as follows:

Index rule defining the index date:

- First exposure to any drug or combination of drugs containing the RxNorm ingredient(s) of interest.

Inclusion rules based on the index date:

- At least 365 days of observation time prior to the index date
- No exposure to the target or comparator ingredient(s) before the index date
- No exposure to any other hypertension treatment on or before the index date
- A diagnosis of hypertension on or preceding the index date

Note that no prior exposure to other hypertension treatments prior to or on the index date is allowed. For example, when comparing chlorthalidone to hydrochlorothiazide, no prior exposure to lisinopril or any other hypertension treatment is allowed.
The end of the exposure cohort is defined as the end of the first exposure, allowing for 30-day gaps between consecutive prescriptions.

Chlorthalidone

Concept Id	Concept Name	Domain	Vocabulary	Exclude	Descendants	Mapped
1395058	Chlorthalidone	Drug	RxNorm	NO	YES	NO

Hydrochlorothiazide

Concept id	Concept Name	Domain	Vocabulary	Exclude	Descendants	Mapped
974166	Hydrochlorothiazide	Drug	RxNorm	NO	YES	NO

© 2020 Hripcsak G. et al. *JAMA Internal Medicine.*

3.2 Outcomes

We first summarize the effectiveness and safety outcomes in a table and then show each detailed definition (including codes used) as described above.

Phenotype name	Logical description	Computer-executable code	Supporting references
Abdominal pain	Abdominal pain condition record of any type; successive records with > 90 day gap are considered independent episodes	https://github.com/OHDSI/Legend/blob/master/inst/cohorts/Abdominal%20pain.json	[25-27]
Abnormal weight gain	Abnormal weight gain record of any type; successive records with > 90 day gap are considered independent episodes; note, weight measurements not used	https://github.com/OHDSI/Legend/blob/master/inst/cohorts/Abnormal%20weight%20gain.json	[28]
Abnormal weight loss	Abnormal weight loss record of any type; successive records with > 90 day gap are considered independent episodes; note, weight measurements not used	https://github.com/OHDSI/Legend/blob/master/inst/cohorts/Abnormal%20weight%20loss.json	[29]
Acute myocardial infarction	Acute myocardial infarction condition record during an inpatient or ER visit; successive records with > 180 day gap are considered independent episodes	https://github.com/OHDSI/Legend/blob/master/inst/cohorts/Acute%20myocardial%20infarction.json	[30-35]
Acute pancreatitis	Acute pancreatitis condition record during an inpatient or ER visit; successive records with >30 day gap are considered independent episodes	https://github.com/OHDSI/Legend/blob/master/inst/cohorts/Acute%20pancreatitis.json	[36-39]
Acute renal failure	A diagnosis of 'acute renal failure' in an inpatient or ER setting; must be at least 30d between inpatient/ER visits to be considered separate episodes	https://github.com/OHDSI/Legend/blob/master/inst/cohorts/Acute%20renal%20failure.json	[40-47]
All-cause mortality	Death record of any type	https://github.com/OHDSI/Legend/blob/master/inst/cohorts/All-cause%20mortality.json	[33, 48, 49]
Anaphylactoid reaction	Anaphylactoid reaction condition record during an inpatient or ER visit; successive records with >7 day gap are considered independent episodes	https://github.com/OHDSI/Legend/blob/master/inst/cohorts/Anaphylactoid%20reaction.json	[50,51]
Anemia	The first condition record of anemia	https://github.com/OHDSI/Legend/blob/master/inst/cohorts/Anemia.json	[52-54]
Angioedema	Angioedema condition record during an inpatient or ER visit; successive records with >7 day gap are considered independent episodes	https://github.com/OHDSI/Legend/blob/master/inst/cohorts/Angioedema.json	[50,55]
Anxiety	The first condition record of anxiety, which is followed by another anxiety condition record or a drug used to treat anxiety	https://github.com/OHDSI/Legend/blob/master/inst/cohorts/Anxiety.json	[56-59]
Bradycardia	The first condition record of bradycardia, which is followed by another bradycardia condition record	https://github.com/OHDSI/Legend/blob/master/inst/cohorts/Bradycardia.json	[60,61]
Cardiac arrhythmia	The first condition record of cardiac arrhythmia, which is followed by another cardiac arrhythmia condition record, at least two drug records for a drug used to treat arrhythmias, or a procedure to treat	https://github.com/OHDSI/Legend/blob/master/inst/cohorts/Cardiac%20arrhythmia.json	[62-68]

© 2020 Hripcsak G. et al. *JAMA Internal Medicine.*

	arrhythmias		
Cardiovascular disease	A condition record of ischemic stroke, hemorrhagic stroke, heart failure, acute myocardial infarction or sudden cardiac death during an inpatient or ER visit; successive records with > 180 day gap are considered independent episodes	https://github.com/OHDSI/Legend/blob/master/inst/cohorts/Cardiovascular%20disease.json	[30-35,62]
Cardiovascular-related mortality	Death record with at least 1 cardiovascular-related condition record (myocardial infarction, ischemic stroke, intracranial hemorrhage, sudden cardiac death, hospitalization for heart failure) in 30 days prior to death	https://github.com/OHDSI/Legend/blob/master/inst/cohorts/Cardiovascular-related%20mortality.json	[33]
Chest pain or angina	The first condition record of chest pain or angina	https://github.com/OHDSI/Legend/blob/master/inst/cohorts/Chest%20pain%20or%20angina.json	[69]
Chronic kidney disease	The first condition record of chronic kidney disease, which is followed by either another chronic kidney disease condition record or a dialysis procedure or observation	https://github.com/OHDSI/Legend/blob/master/inst/cohorts/Chronic%20kidney%20disease.json	[42,70-77]
Cough	Cough condition record of any type; successive records with > 90 day gap are considered independent episodes	https://github.com/OHDSI/Legend/blob/master/inst/cohorts/Cough.json	[78-80]
Decreased libido	The first condition record of decreased libido	https://github.com/OHDSI/Legend/blob/master/inst/cohorts/Decreased%20libido.json	[81]
Dementia	The first condition record of dementia	https://github.com/OHDSI/Legend/blob/master/inst/cohorts/Dementia.json	[82-89]
Depression	The first condition record of depression, which is followed by another depression condition record, at least two drugs used to treat depression without another indication, or two psychotherapy procedures	https://github.com/OHDSI/Legend/blob/master/inst/cohorts/Depression.json	[57,58,88, 90-93]
Diarrhea	Diarrhea condition record of any type; successive records with > 30 day gap are considered independent episodes	https://github.com/OHDSI/Legend/blob/master/inst/cohorts/Diarrhea.json	[94-96]
End stage renal disease	End stage renal disease (ESRD) is defined by at least one diagnosis in any setting, followed by at least one additional diagnosis or a dialysis-related procedure within 90 days	https://github.com/OHDSI/Legend/blob/master/inst/cohorts/End%20stage%20renal%20disease.json	[39,75,97]
Fall	Fall condition record of any type; successive records with > 180 day gap are considered independent episodes	https://github.com/OHDSI/Legend/blob/master/inst/cohorts/Fall.json	[98-100]
Gastrointestinal bleeding	Gastrointestinal hemorrhage condition record during an inpatient or ER visit; successive records with > 30 day gap are considered independent episodes	https://github.com/OHDSI/Legend/blob/master/inst/cohorts/Gastrointestinal%20bleeding.json	[25,34, 101-105]
Gout	The first condition record of gout	https://github.com/OHDSI/Legend/blob/master/inst/cohorts/Gout.json	[106-109]
Headache	Headache condition record of any type;	https://github.com/OHDSI/Legen	[110,111]

© 2020 Hripcsak G. et al. *JAMA Internal Medicine.*

	successive records with > 30 day gap are considered independent episodes	d/blob/master/inst/cohorts/Headache.json	
Heart failure	The first condition record of heart failure, which is followed by at least 1 heart failure condition record in the following year	https://github.com/OHDSI/Legend/blob/master/inst/cohorts/Heart%20failure.json	[31,112-121]
Hemorrhagic stroke	Intracranial, cerebral or subarachnoid hemorrhage condition record during an inpatient or ER visit; successive records with > 180 day gap are considered independent episodes	https://github.com/OHDSI/Legend/blob/master/inst/cohorts/Hemorrhagic%20stroke.json	[122-126]
Hepatic failure	The first condition record of hepatic failure, necrosis, or coma	https://github.com/OHDSI/Legend/blob/master/inst/cohorts/Hepatic%20failure.json	[40, 127-113]
Hospitalization with heart failure	Inpatient or ER visits with heart failure condition record; all qualifying inpatient visits occurring > 7 days apart are considered independent episodes	https://github.com/OHDSI/Legend/blob/master/inst/cohorts/Hospitalization%20with%20heart%20failure.json	[114,119 120,135,136]
Hospitalization with preinfarction syndrome	Inpatient or ER visits with preinfarction syndrome condition record; all qualifying inpatient visits occurring > 7 days apart are considered independent episodes	https://github.com/OHDSI/Legend/blob/master/inst/cohorts/Hospitalization%20with%20preinfarction%20syndrome.json	[69,137,138]
Hyperkalemia	Condition record for hyperkalemia or potassium measurements > 5.6 mmol/L; successive records with >90 day gap are considered independent episodes	https://github.com/OHDSI/Legend/blob/master/inst/cohorts/Hyperkalemia.json	[139-141]
Hypokalemia	Hypokalemia condition record of any type; successive records with > 90 day gap are considered independent episodes	https://github.com/OHDSI/Legend/blob/master/inst/cohorts/Hypokalemia.json	[142]
Hypomagnesemia	Hypomagnesemia condition record of any type; successive records with > 90 day gap are considered independent episodes	https://github.com/OHDSI/Legend/blob/master/inst/cohorts/Hypomagnesemia.json	[143,144]
Hyponatremia	Hyponatremia condition record of any type; successive records with > 90 day gap are considered independent episodes	https://github.com/OHDSI/Legend/blob/master/inst/cohorts/Hyponatremia.json	[145,146]
Hypotension	Hypotension condition record of any type; successive records with > 90 day gap are considered independent episodes	https://github.com/OHDSI/Legend/blob/master/inst/cohorts/Hypotension.json	[147]
Impotence	The first condition record of impotence	https://github.com/OHDSI/Legend/blob/master/inst/cohorts/Impotence.json	[148-151]
Ischemic stroke	Ischemic stroke condition record during an inpatient or ER visit; successive records with > 180 day gap are considered independent episodes	https://github.com/OHDSI/Legend/blob/master/inst/cohorts/Ischemic%20stroke.json	[31,33,34, 122,152]
Malignant neoplasm	First occurrence of malignant neoplasm, followed by at least one additional diagnosis of the same type (melanoma, bladder, brain, breast, colon and rectum, kidney, leukemia, liver, lung, lymphoma, multiple myeloma, ovary, pancreas, prostate, thyroid, uterus, myelodysplastic syndrome)	https://github.com/OHDSI/Legend/blob/master/inst/cohorts/Malignant%20neoplasm.json	[153-168]

© 2020 Hripcsak G. et al. *JAMA Internal Medicine.*

Measured renal dysfunction	The first creatinine measurement with value > 3 mg/dL	https://github.com/OHDSI/Legend/blob/master/inst/cohorts/Measured%20renal%20dysfunction.json	[47]
Nausea	Nausea condition record of any type; successive records with > 30 day gap are considered independent episodes	https://github.com/OHDSI/Legend/blob/master/inst/cohorts/Nausea.json	[25,169,170]
Neutropenia or agranulocytosis	The first condition record of neutropenia or agranulocytosis	https://github.com/OHDSI/Legend/blob/master/inst/cohorts/Neutropenia%20or%20agranulocytosis.json	[171, 172]
Rash	Rash condition record of any type; successive records with > 90 day gap are considered independent episodes	https://github.com/OHDSI/Legend/blob/master/inst/cohorts/Rash.json	[173]
Rhabdomyolysis	Rhabdomyolysis condition record or muscle disorder condition record with creatine measurement 5*ULN during an inpatient or ER visit; successive records with >90 day gap are considered independent episodes	https://github.com/OHDSI/Legend/blob/master/inst/cohorts/Rhabdomyolysis.json	[174,175]
Stroke	Stroke (ischemic or hemorrhagic) condition record during an inpatient or ER visit; successive records with > 180 day gap are considered independent episodes	https://github.com/OHDSI/Legend/blob/master/inst/cohorts/Stroke.json	[31,33,34,64,122-126,152]
Sudden cardiac death	Sudden cardiac death condition record during an inpatient or ER visit; successive records with > 180 day gap are considered independent episodes	https://github.com/OHDSI/Legend/blob/master/inst/cohorts/Sudden%20cardiac%20death.json	[33,62]
Syncope	Syncope condition record of any type; successive records with > 180 day gap are considered independent episodes	https://github.com/OHDSI/Legend/blob/master/inst/cohorts/Syncope.json	[147]
Thrombocytopenia	The first condition record of thrombocytopenia	https://github.com/OHDSI/Legend/blob/master/inst/cohorts/Thrombocytopenia.json	[169,176,177]
Transient ischemic attack	Transient ischemic attack condition record during an inpatient or ER visit; successive records with > 30 day gap are considered independent episodes	https://github.com/OHDSI/Legend/blob/master/inst/cohorts/Transient%20ischemic%20attack.json	[122,152]
Type 2 diabetes mellitus	The first condition record of Type 2 Diabetes Mellitus, which is followed by another Type 2 Diabetes Mellitus condition record, at least 2 drugs used to treat Type 2 diabetes, or at least 2 HbA1c measurements with value > 6.5%	https://github.com/OHDSI/Legend/blob/master/inst/cohorts/Type%202%20diabetes%20mellitus.json	[88,178-180]
Vasculitis	The first condition record of vasculitis, which is followed by another vasculitis condition record or drug to treat vasculitis	https://github.com/OHDSI/Legend/blob/master/inst/cohorts/Vasculitis.json	[181,182]
Venous thromboembolic events	Venous thromboembolism condition record of any type; successive records with > 180 day gap are considered independent episodes	https://github.com/OHDSI/Legend/blob/master/inst/cohorts/Venous%20thromboembolic%20events%20.json	[183-186]
Vertigo	The first condition record of vertigo	https://github.com/OHDSI/Legend/blob/master/inst/cohorts/Vertig	[187]

© 2020 Hripcsak G. et al. *JAMA Internal Medicine.*

		0.json	
Vomiting	Vomiting condition record of any type; successive records with > 30 day gap are considered independent episodes	https://github.com/OHDSI/Legend/blob/master/inst/cohorts/Vomiting.json	[25,169,170]

3.2.1 Abdominal pain

Abdominal pain events

Abdominal pain condition record of any type; successive records with > 90 day gap are considered independent episodes

Initial Event Cohort

People having any of the following:

- a condition occurrence of Abdominal pain[1]

with continuous observation of at least 0 days prior and 0 days after event index date, and limit initial events to: **all events per person.**

Limit qualifying cohort to: **all events per person.**

End Date Strategy

Date Offset Exit Criteria

This cohort definition end date will be the index event's start date plus 1 days

Cohort Collapse Strategy:

Collapse cohort by era with a gap size of 90 days.

1. Abdominal pain

Concept Id	Concept Name	Domain	Vocabulary	Excluded	Descendants	Mapped
200219	Abdominal pain	Condition	SNOMED	NO	YES	NO

3.2.2 Abnormal weight gain

Abnormal weight gain events

Abnormal weight gain record of any type; successive records with > 90 day gap are considered independent episodes

Initial Event Cohort

People having any of the following:

- an observation of Abnormal weight gain[1]

with continuous observation of at least 0 days prior and 0 days after event index date, and limit initial events to: **all events per person.**

Limit qualifying cohort to: **all events per person.**

End Date Strategy

Date Offset Exit Criteria

This cohort definition end date will be the index event's start date plus 1 days
Cohort Collapse Strategy:
Collapse cohort by era with a gap size of 90 days.

1. Abnormal weight gain

Concept Id	Concept Name	Domain	Vocabulary	Excluded	Descendants	Mapped
439141	Abnormal weight gain	Observation	SNOMED	NO	YES	NO

3.2.3 Abnormal weight loss

Abnormal weight loss events
Abnormal weight loss observation record of any type; successive records with > 90 day gap are considered independent episodes
Initial Event Cohort
People having any of the following:

- an observation of Abnormal weight loss[1]

with continuous observation of at least 0 days prior and 0 days after event index date, and limit initial events to: **all events per person.**

Limit qualifying cohort to: **all events per person.**
End Date Strategy
Date Offset Exit Criteria
This cohort definition end date will be the index event's start date plus 1 days
Cohort Collapse Strategy:
Collapse cohort by era with a gap size of 90 days.

1. Abnormal weight loss

Concept Id	Concept Name	Domain	Vocabulary	Excluded	Descendants	Mapped
435928	Abnormal weight loss	Observation	SNOMED	NO	YES	NO

3.2.4 Acute myocardial infarction

Acute myocardial infarction events
Acute myocardial infarction condition record during an inpatient or ER visit; successive records with > 180 day gap are considered independent episodes
Initial Event Cohort
People having any of the following:

© 2020 Hripcsak G. et al. *JAMA Internal Medicine.*

- a condition occurrence of Acute myocardial Infarction[2]

with continuous observation of at least 0 days prior and 0 days after event index date, and limit initial events to: **all events per person.**

For people matching the Primary Events, include:
Having any of the following criteria:

- at least 1 occurrences of a visit occurrence of Inpatient or ER visit[1]

  where event starts between all days Before and 0 days After index start date and event ends between 0 days Before and all days After index start date

Limit cohort of initial events to: **all events per person.**
Limit qualifying cohort to: **all events per person.**
End Date Strategy
Date Offset Exit Criteria
This cohort definition end date will be the index event's start date plus 7 days
Cohort Collapse Strategy:
Collapse cohort by era with a gap size of 180 days.

1. Inpatient or ER visit

Concept Id	Concept Name	Domain	Vocabulary	Excluded	Descendants	Mapped
262	Emergency Room and Inpatient Visit	Visit	Visit	NO	YES	NO
9203	Emergency Room Visit	Visit	Visit	NO	YES	NO
9201	Inpatient Visit	Visit	Visit	NO	YES	NO

2. Acute myocardial Infarction

Concept Id	Concept Name	Domain	Vocabulary	Excluded	Descendants	Mapped
4329847	Myocardial infarction	Condition	SNOMED	NO	YES	NO

© 2020 Hripcsak G. et al. *JAMA Internal Medicine.*

Concept Id	Concept Name	Domain	Vocabulary	Excluded	Descendants	Mapped
314666	Old myocardial infarction	Condition	SNOMED	YES	YES	NO

3.2.5 Acute pancreatitis

Acute pancreatitis events

Acute pancreatitis condition record during an inpatient or ER visit; successive records with >30 day gap are considered independent episodes

Initial Event Cohort

People having any of the following:

- a condition occurrence of Acute pancreatitis[2]

with continuous observation of at least 0 days prior and 0 days after event index date, and limit initial events to: **all events per person.**

For people matching the Primary Events, include:
Having any of the following criteria:

- at least 1 occurrences of a visit occurrence of Inpatient or ER visit[1]

  where event starts between all days Before and 0 days After index start date and event ends between 0 days Before and all days After index start date

Limit cohort of initial events to: **all events per person.**
Limit qualifying cohort to: **all events per person.**
End Date Strategy

Date Offset Exit Criteria

This cohort definition end date will be the index event's start date plus 7 days
Cohort Collapse Strategy:

Collapse cohort by era with a gap size of 30 days.

1. Inpatient or ER visit

Concept Id	Concept Name	Domain	Vocabulary	Excluded	Descendants	Mapped
262	Emergency Room and Inpatient Visit	Visit	Visit	NO	YES	NO

© 2020 Hripcsak G. et al. *JAMA Internal Medicine.*

Concept Id	Concept Name	Domain	Vocabulary	Excluded	Descendants	Mapped
9203	Emergency Room Visit	Visit	Visit	NO	YES	NO
9201	Inpatient Visit	Visit	Visit	NO	YES	NO

2. Acute pancreatitis

Concept Id	Concept Name	Domain	Vocabulary	Excluded	Descendants	Mapped
199074	Acute pancreatitis	Condition	SNOMED	NO	YES	NO

3.2.6 Acute renal failure

Acute renal failure events

A diagnosis of 'acute renal failure' in an inpatient or ER setting; must be at least 30d between inpatient/ER visits to be considered separate episodes

Initial Event Cohort

People having any of the following:

- a condition occurrence of Acute Renal Failure[2]

with continuous observation of at least 0 days prior and 0 days after event index date, and limit initial events to: **all events per person.**

For people matching the Primary Events, include:
Having any of the following criteria:

- at least 1 occurrences of a visit occurrence of Inpatient or ER visit[1]

  where event starts between all days Before and 0 days After index start date and event ends between 0 days Before and all days After index start date

Limit cohort of initial events to: **all events per person.**
Limit qualifying cohort to: **all events per person.**

End Date Strategy

Date Offset Exit Criteria

This cohort definition end date will be the index event's start date plus 30 days

Cohort Collapse Strategy:

Collapse cohort by era with a gap size of 30 days.

© 2020 Hripcsak G. et al. *JAMA Internal Medicine.*

1. Inpatient or ER visit

Concept Id	Concept Name	Domain	Vocabulary	Excluded	Descendants	Mapped
262	Emergency Room and Inpatient Visit	Visit	Visit	NO	YES	NO
9203	Emergency Room Visit	Visit	Visit	NO	YES	NO
9201	Inpatient Visit	Visit	Visit	NO	YES	NO

2. Acute Renal Failure

Concept Id	Concept Name	Domain	Vocabulary	Excluded	Descendants	Mapped
197320	Acute renal failure syndrome	Condition	SNOMED	NO	YES	NO
432961	Acute renal papillary necrosis with renal failure	Condition	SNOMED	NO	YES	NO
444044	Acute tubular necrosis	Condition	SNOMED	NO	YES	NO

3.2.7 All-cause mortality

All-cause mortality

Death record of any type

Initial Event Cohort

People having any of the following:

- a death occurrence from Any Death

with continuous observation of at least 0 days prior and 0 days after event index date, and limit initial events to: **earliest event per person.**

Limit qualifying cohort to: **earliest event per person.**

End Date Strategy

© 2020 Hripcsak G. et al. *JAMA Internal Medicine.*

No end date strategy selected. By default, the cohort end date will be the end of the observation period that contains the index event.
Cohort Collapse Strategy:
Collapse cohort by era with a gap size of 0 days.

3.2.8 Anaphylactoid reaction

Anaphylactoid reaction events
Anaphylactoid reaction condition record during an inpatient or ER visit; successive records with >7 day gap are considered independent episodes
Initial Event Cohort
People having any of the following:

- a condition occurrence of Anaphylactoid reaction[2]

with continuous observation of at least 0 days prior and 0 days after event index date, and limit initial events to: **all events per person.**

For people matching the Primary Events, include:
Having any of the following criteria:

- at least 1 occurrences of a visit occurrence of Inpatient or ER visit[1]

  where event starts between all days Before and 0 days After index start date and event ends between 0 days Before and all days After index start date

Limit cohort of initial events to: **all events per person.**
Limit qualifying cohort to: **all events per person.**
End Date Strategy
Date Offset Exit Criteria
This cohort definition end date will be the index event's start date plus 7 days
Cohort Collapse Strategy:
Collapse cohort by era with a gap size of 7 days.

1. Inpatient or ER visit

Concept Id	Concept Name	Domain	Vocabulary	Excluded	Descendants	Mapped
262	Emergency Room and Inpatient Visit	Visit	Visit	NO	YES	NO
9203	Emergency Room Visit	Visit	Visit	NO	YES	NO
9201	Inpatient Visit	Visit	Visit	NO	YES	NO

© 2020 Hripcsak G. et al. *JAMA Internal Medicine.*

2. Anaphylactoid reaction

Concept Id	Concept Name	Domain	Vocabulary	Excluded	Descendants	Mapped
441202	Anaphylaxis	Condition	SNOMED	NO	YES	NO
4330225	Anaphylaxis due to hymenoptera venom	Condition	SNOMED	YES	YES	NO
40479646	Anaphylaxis due to latex	Condition	SNOMED	YES	YES	NO
4299299	Anaphylaxis secondary to bite and/or sting	Condition	SNOMED	YES	YES	NO
4084633	Bee sting-induced anaphylaxis	Condition	SNOMED	YES	YES	NO
434219	Food anaphylaxis	Condition	SNOMED	YES	YES	NO
4086737	Insulin-induced anaphylaxis	Condition	SNOMED	YES	YES	NO
4084634	Wasp sting-induced anaphylaxis	Condition	SNOMED	YES	YES	NO

3. Angioedema

Concept Id	Concept Name	Domain	Vocabulary	Excluded	Descendants	Mapped
432791	Angioedema	Condition	SNOMED	NO	YES	NO

© 2020 Hripcsak G. et al. *JAMA Internal Medicine.*

3.2.9 Anemia

Persons with anemia

The first condition record of anemia

Initial Event Cohort

People having any of the following:

- a condition occurrence of Anemia[1]
  - for the first time in the person's history

with continuous observation of at least 0 days prior and 0 days after event index date, and limit initial events to: **earliest event per person.**

Limit qualifying cohort to: **earliest event per person.**

End Date Strategy

No end date strategy selected. By default, the cohort end date will be the end of the observation period that contains the index event.

Cohort Collapse Strategy:

Collapse cohort by era with a gap size of 0 days.

1. Anemia

Concept Id	Concept Name	Domain	Vocabulary	Excluded	Descendants	Mapped
439777	Anemia	Condition	SNOMED	NO	YES	NO
137829	Aplastic anemia	Condition	SNOMED	NO	YES	NO
437090	Hemolytic disease of fetus OR newborn due to ABO immunization	Condition	SNOMED	YES	YES	NO
440218	Hemolytic disease of fetus OR newborn due to isoimmunization	Condition	SNOMED	YES	YES	NO
25518	Sickle cell trait	Condition	SNOMED	NO	YES	NO
24006	Sickle cell-hemoglobin C disease	Condition	SNOMED	NO	YES	NO

© 2020 Hripcsak G. et al. *JAMA Internal Medicine.*

Concept Id	Concept Name	Domain	Vocabulary	Excluded	Descendants	Mapped
4301602	Thrombotic thrombocytopenic purpura	Condition	SNOMED	YES	YES	NO

3.2.10 Angioedema

Angioedema events

Angioedema condition record during an inpatient or ER visit; successive records with >7 day gap are considered independent episodes

Initial Event Cohort

People having any of the following:

- a condition occurrence of Angioedema[2]

with continuous observation of at least 0 days prior and 0 days after event index date, and limit initial events to: **all events per person.**

For people matching the Primary Events, include:
Having any of the following criteria:

- at least 1 occurrences of a visit occurrence of Inpatient or ER visit[1]

  where event starts between all days Before and 0 days After index start date and event ends between 0 days Before and all days After index start date

Limit cohort of initial events to: **all events per person.**
Limit qualifying cohort to: **all events per person.**

End Date Strategy

Date Offset Exit Criteria

This cohort definition end date will be the index event's start date plus 7 days

Cohort Collapse Strategy:

Collapse cohort by era with a gap size of 30 days.

1. Inpatient or ER visit

Concept Id	Concept Name	Domain	Vocabulary	Excluded	Descendants	Mapped
262	Emergency Room and Inpatient Visit	Visit	Visit	NO	YES	NO

© 2020 Hripcsak G. et al. *JAMA Internal Medicine.*

Concept Id	Concept Name	Domain	Vocabulary	Excluded	Descendants	Mapped
9203	Emergency Room Visit	Visit	Visit	NO	YES	NO
9201	Inpatient Visit	Visit	Visit	NO	YES	NO

2. Angioedema

Concept Id	Concept Name	Domain	Vocabulary	Excluded	Descendants	Mapped
432791	Angioedema	Condition	SNOMED	NO	YES	NO

3.2.11 Anxiety

Persons with anxiety

The first condition record of anxiety, which is followed by another anxiety condition record or a drug used to treat anxiety

Initial Event Cohort

People having any of the following:

- a condition occurrence of Anxiety[1]
  - for the first time in the person's history

with continuous observation of at least 0 days prior and 0 days after event index date, and limit initial events to: **earliest event per person.**

For people matching the Primary Events, include:
Having any of the following criteria:

- at least 1 occurrences of a condition occurrence of Anxiety[1]

  where event starts between 1 days After and all days After index start date

- or at least 1 occurrences of a drug exposure of Drugs to treat anxiety[3]

  where event starts between 0 days Before and 30 days After index start date

Limit cohort of initial events to: **earliest event per person.**
Limit qualifying cohort to: **earliest event per person.**
End Date Strategy

© 2020 Hripcsak G. et al. *JAMA Internal Medicine.*

No end date strategy selected. By default, the cohort end date will be the end of the observation period that contains the index event.
Cohort Collapse Strategy:
Collapse cohort by era with a gap size of 0 days.

1. Anxiety

Concept Id	Concept Name	Domain	Vocabulary	Excluded	Descendants	Mapped
442077	Anxiety disorder	Condition	SNOMED	NO	NO	NO
37109206	Anxiety disorder caused by drug	Condition	SNOMED	NO	YES	NO
4199892	Anxiety disorder due to a general medical condition	Condition	SNOMED	NO	YES	NO
434613	Generalized anxiety disorder	Condition	SNOMED	NO	YES	NO
4338031	Mixed anxiety and depressive disorder	Condition	SNOMED	NO	YES	NO
381537	Organic anxiety disorder	Condition	SNOMED	NO	YES	NO
436074	Panic disorder	Condition	SNOMED	NO	YES	NO
4304010	Phobic disorder	Condition	SNOMED	YES	YES	NO

2. Depression

© 2020 Hripcsak G. et al. *JAMA Internal Medicine.*

Concept Id	Concept Name	Domain	Vocabulary	Excluded	Descendants	Mapped
442306	Adjustment disorder with depressed mood	Condition	SNOMED	NO	YES	NO
436665	Bipolar disorder	Condition	SNOMED	YES	YES	NO
440383	Depressive disorder	Condition	SNOMED	NO	YES	NO
4175329	Organic mood disorder of depressed type	Condition	SNOMED	NO	YES	NO

3. Drugs to treat anxiety

Concept Id	Concept Name	Domain	Vocabulary	Excluded	Descendants	Mapped
781039	Alprazolam	Drug	RxNorm	NO	YES	NO
733301	Buspirone	Drug	RxNorm	NO	YES	NO
990678	Chlordiazepoxide	Drug	RxNorm	NO	YES	NO
797617	Citalopram	Drug	RxNorm	NO	YES	NO
19050832	clobazam	Drug	RxNorm	NO	YES	NO
798874	Clonazepam	Drug	RxNorm	NO	YES	NO
790253	clorazepate	Drug	RxNorm	NO	YES	NO

© 2020 Hripcsak G. et al. *JAMA Internal Medicine.*

Concept Id	Concept Name	Domain	Vocabulary	Excluded	Descendants	Mapped
717607	Desvenlafaxine	Drug	RxNorm	NO	YES	NO
723013	Diazepam	Drug	RxNorm	NO	YES	NO
739323	Droperidol	Drug	RxNorm	NO	YES	NO
715259	duloxetine	Drug	RxNorm	NO	YES	NO
715939	Escitalopram	Drug	RxNorm	NO	YES	NO
755695	Fluoxetine	Drug	RxNorm	NO	YES	NO
751412	Fluvoxamine	Drug	RxNorm	NO	YES	NO
777221	Hydroxyzine	Drug	RxNorm	NO	YES	NO
791967	Lorazepam	Drug	RxNorm	NO	YES	NO
702865	Meprobamate	Drug	RxNorm	NO	YES	NO
724816	Oxazepam	Drug	RxNorm	NO	YES	NO
722031	Paroxetine	Drug	RxNorm	NO	YES	NO
739138	Sertraline	Drug	RxNorm	NO	YES	NO
743670	venlafaxine	Drug	RxNorm	NO	YES	NO

4. Drugs to treat depression

© 2020 Hripcsak G. et al. *JAMA Internal Medicine.*

Concept Id	Concept Name	Domain	Vocabulary	Excluded	Descendants	Mapped
21604686	ANTIDEPRESSANTS	Drug	ATC	NO	YES	NO

3.2.12 Bradycardia

Persons with bradycardia

Initial Event Cohort

People having any of the following:

- a condition occurrence of Bradycardia[1]
  - for the first time in the person's history

with continuous observation of at least 0 days prior and 0 days after event index date, and limit initial events to: **earliest event per person.**

For people matching the Primary Events, include:
Having all of the following criteria:

- at least 1 occurrences of a condition occurrence of Bradycardia[1]

  where event starts between 1 days After and all days After index start date

Limit cohort of initial events to: **earliest event per person.**
Limit qualifying cohort to: **earliest event per person.**
End Date Strategy

No end date strategy selected. By default, the cohort end date will be the end of the observation period that contains the index event.
Cohort Collapse Strategy:

Collapse cohort by era with a gap size of 0 days.

1. Bradycardia

Concept Id	Concept Name	Domain	Vocabulary	Excluded	Descendants	Mapped
4169095	Bradycardia	Condition	SNOMED	NO	YES	NO
316999	Conduction disorder of the heart	Condition	SNOMED	NO	NO	NO
4171683	Sinus bradycardia	Condition	SNOMED	NO	YES	NO

© 2020 Hripcsak G. et al. *JAMA Internal Medicine.*

Concept Id	Concept Name	Domain	Vocabulary	Excluded	Descendants	Mapped
317302	Sinus node dysfunction	Condition	SNOMED	NO	YES	NO

3.2.13 Cardiac arrhythmia

Person with cardiac arrhythmia

The first condition record of cardiac arrhythmia, which is followed by another cardiac arrhythmia condition record, at least two drug records for a drug used to treat arrhythmias, or a procedure to treat arrhythmias

Initial Event Cohort

People having any of the following:

- a condition occurrence of Cardiac arrhythmia[1]
  - for the first time in the person's history

with continuous observation of at least 0 days prior and 0 days after event index date, and limit initial events to: **earliest event per person.**

For people matching the Primary Events, include:
Having any of the following criteria:

- at least 1 occurrences of a condition occurrence of Cardiac arrhythmia[1]

  where event starts between 1 days After and all days After index start date

- or at least 2 occurrences of a drug exposure of Drugs used to treat cardiac arrhythmia[2]

  where event starts between 0 days Before and all days After index start date

- or at least 1 occurrences of a procedure of Procedures to treat cardiac arrhythmia[3]

  where event starts between 0 days Before and all days After index start date

Limit cohort of initial events to: **earliest event per person.**
Limit qualifying cohort to: **earliest event per person.**

End Date Strategy

No end date strategy selected. By default, the cohort end date will be the end of the observation period that contains the index event.

Cohort Collapse Strategy:

Collapse cohort by era with a gap size of 0 days.

1. Cardiac arrhythmia

© 2020 Hripcsak G. et al. *JAMA Internal Medicine.*

Concept Id	Concept Name	Domain	Vocabulary	Excluded	Descendants	Mapped
44784217	Cardiac arrhythmia	Condition	SNOMED	NO	YES	NO
38001137	Cardiac arrhythmia & conduction disorders w CC	Observation	DRG	NO	YES	NO
38001138	Cardiac arrhythmia & conduction disorders w/o CC/MCC	Observation	DRG	NO	YES	NO
315078	Palpitations	Condition	SNOMED	NO	YES	NO
444070	Tachycardia	Condition	SNOMED	NO	YES	NO

2. Drugs used to treat cardiac arrhythmia

Concept Id	Concept Name	Domain	Vocabulary	Excluded	Descendants	Mapped
21600248	ANTIARRHYTHMICS, CLASS I AND III	Drug	ATC	NO	YES	NO
43013024	apixaban	Drug	RxNorm	NO	YES	NO
40228152	dabigatran etexilate	Drug	RxNorm	NO	YES	NO
40241331	rivaroxaban	Drug	RxNorm	NO	YES	NO
1310149	Warfarin	Drug	RxNorm	NO	YES	NO

3. Procedures to treat cardiac arrhythmia

© 2020 Hripcsak G. et al. *JAMA Internal Medicine.*

Concept Id	Concept Name	Domain	Vocabulary	Excluded	Descendants	Mapped
45890325	Cardioversion, elective, electrical conversion of arrhythmia	Procedure	CPT4	NO	YES	NO
45890400	Operative tissue ablation and reconstruction of atria, extensive (eg, maze procedure)	Procedure	CPT4	NO	YES	NO
2107068	Operative tissue ablation and reconstruction of atria, performed at the time of other cardiac procedure(s), extensive (eg, maze procedure), with cardiopulmonary bypass (List separately in addition to code for primary procedure)	Procedure	CPT4	NO	YES	NO
4051932	Procedure for arrhythmia	Procedure	SNOMED	NO	YES	NO

3.2.14 Cardiovascular disease

Total cardiovascular disease events (ischemic stroke, hemorrhagic stroke, heart failure, acute myocardial infarction or sudden cardiac death)

A condition record of ischemic stroke, hemorrhagic stroke, heart failure, acute myocardial infarction or sudden cardiac death during an inpatient or ER visit; successive records with > 180 day gap are considered independent episodes

Initial Event Cohort

People having any of the following:

© 2020 Hripcsak G. et al. *JAMA Internal Medicine.*

- a condition occurrence of Acute myocardial Infarction[2]
- a condition occurrence of Sudden cardiac death[6]
- a condition occurrence of Ischemic stroke[5]
- a condition occurrence of intracranial bleed Hemorrhagic stroke[4]
- a condition occurrence of Heart Failure [3]

with continuous observation of at least 0 days prior and 0 days after event index date, and limit initial events to: **all events per person.**

For people matching the Primary Events, include:
Having all of the following criteria:

- at least 1 occurrences of a visit occurrence of Inpatient or ER visit[1]

  where event starts between all days Before and 0 days After index start date and event ends between 0 days Before and all days After index start date

Limit cohort of initial events to: **all events per person.**
Limit qualifying cohort to: **all events per person.**
End Date Strategy
Date Offset Exit Criteria
This cohort definition end date will be the index event's start date plus 7 days
Cohort Collapse Strategy:
Collapse cohort by era with a gap size of 180 days.

1. Inpatient or ER visit

Concept Id	Concept Name	Domain	Vocabulary	Excluded	Descendants	Mapped
262	Emergency Room and Inpatient Visit	Visit	Visit	NO	YES	NO
9203	Emergency Room Visit	Visit	Visit	NO	YES	NO
9201	Inpatient Visit	Visit	Visit	NO	YES	NO

2. Acute myocardial Infarction

© 2020 Hripcsak G. et al. *JAMA Internal Medicine.*

Concept Id	Concept Name	Domain	Vocabulary	Excluded	Descendants	Mapped
4329847	Myocardial infarction	Condition	SNOMED	NO	YES	NO
314666	Old myocardial infarction	Condition	SNOMED	YES	YES	NO

3. Heart Failure

Concept Id	Concept Name	Domain	Vocabulary	Excluded	Descendants	Mapped
315295	Congestive rheumatic heart failure	Condition	SNOMED	YES	YES	NO
316139	Heart failure	Condition	SNOMED	NO	YES	NO

4. Intracranial bleed Hemorrhagic stroke

Concept Id	Concept Name	Domain	Vocabulary	Excluded	Descendants	Mapped
376713	Cerebral hemorrhage	Condition	SNOMED	NO	NO	NO
439847	Intracranial hemorrhage	Condition	SNOMED	NO	NO	NO
432923	Subarachnoid hemorrhage	Condition	SNOMED	NO	NO	NO

5. Ischemic stroke

© 2020 Hripcsak G. et al. *JAMA Internal Medicine.*

Concept Id	Concept Name	Domain	Vocabulary	Excluded	Descendants	Mapped
372924	Cerebral artery occlusion	Condition	SNOMED	NO	NO	NO
375557	Cerebral embolism	Condition	SNOMED	NO	NO	NO
443454	Cerebral infarction	Condition	SNOMED	NO	YES	NO
441874	Cerebral thrombosis	Condition	SNOMED	NO	NO	NO

6. Sudden cardiac death

Concept Id	Concept Name	Domain	Vocabulary	Excluded	Descendants	Mapped
4048809	Brainstem death	Condition	SNOMED	NO	YES	NO
321042	Cardiac arrest	Condition	SNOMED	NO	YES	NO
442289	Death in less than 24 hours from onset of symptoms	Observation	SNOMED	NO	YES	NO
4317150	Sudden cardiac death	Condition	SNOMED	NO	YES	NO
4132309	Sudden death	Observation	SNOMED	NO	YES	NO
437894	Ventricular fibrillation	Condition	SNOMED	YES	YES	NO

© 2020 Hripcsak G. et al. *JAMA Internal Medicine.*

3.2.15 Cardiovascular-related mortality

Cardiovascular-related mortality

Death record with at least 1 cardiovascular-related condition record (myocardial infarction, ischemic stroke, intracranial hemorrhage, sudden cardiac death, hospitalization for heart failure) in 30 days prior to death

Initial Event Cohort

People having any of the following:

- a death occurrence from Any Death

with continuous observation of at least 0 days prior and 0 days after event index date, and limit initial events to: **earliest event per person.**

For people matching the Primary Events, include:
Having any of the following criteria:

- at least 1 occurrences of a condition occurrence of Acute myocardial Infarction[2]

  where event starts between 30 days Before and 7 days After index start date

- or at least 1 occurrences of a condition occurrence of Ischemic stroke[5]

  where event starts between 30 days Before and 7 days After index start date

- or at least 1 occurrences of a condition occurrence of intracranial bleed Hemorrhagic stroke[4]

  where event starts between all days Before and all days After index start date

- or at least 1 occurrences of a condition occurrence of Sudden cardiac death[6]

  where event starts between 30 days Before and 7 days After index start date

- or at least 1 occurrences of a condition occurrence of Heart Failure [3]

  Having all of the following criteria:

  - at least 1 occurrences of a visit occurrence of Inpatient or ER visit[1]

    where event starts between all days Before and 0 days After index start date and event ends between 0 days Before and all days After index start date

  where event starts between 30 days Before and 7 days After index start date

Limit cohort of initial events to: **earliest event per person.**
Limit qualifying cohort to: **earliest event per person.**
End Date Strategy

© 2020 Hripcsak G. et al. *JAMA Internal Medicine.*

No end date strategy selected. By default, the cohort end date will be the end of the observation period that contains the index event.
Cohort Collapse Strategy:
Collapse cohort by era with a gap size of 0 days.

1. Inpatient or ER visit

Concept Id	Concept Name	Domain	Vocabulary	Excluded	Descendants	Mapped
262	Emergency Room and Inpatient Visit	Visit	Visit	NO	YES	NO
9203	Emergency Room Visit	Visit	Visit	NO	YES	NO
9201	Inpatient Visit	Visit	Visit	NO	YES	NO

2. Acute myocardial Infarction

Concept Id	Concept Name	Domain	Vocabulary	Excluded	Descendants	Mapped
4329847	Myocardial infarction	Condition	SNOMED	NO	YES	NO
314666	Old myocardial infarction	Condition	SNOMED	YES	YES	NO

3. Heart Failure

Concept Id	Concept Name	Domain	Vocabulary	Excluded	Descendants	Mapped
315295	Congestive rheumatic heart failure	Condition	SNOMED	YES	YES	NO
316139	Heart failure	Condition	SNOMED	NO	YES	NO

© 2020 Hripcsak G. et al. *JAMA Internal Medicine.*

4. intracranial bleed Hemorrhagic stroke

Concept Id	Concept Name	Domain	Vocabulary	Excluded	Descendants	Mapped
376713	Cerebral hemorrhage	Condition	SNOMED	NO	NO	NO
439847	Intracranial hemorrhage	Condition	SNOMED	NO	NO	NO
432923	Subarachnoid hemorrhage	Condition	SNOMED	NO	NO	NO

5. Ischemic stroke

Concept Id	Concept Name	Domain	Vocabulary	Excluded	Descendants	Mapped
372924	Cerebral artery occlusion	Condition	SNOMED	NO	NO	NO
375557	Cerebral embolism	Condition	SNOMED	NO	NO	NO
443454	Cerebral infarction	Condition	SNOMED	NO	YES	NO
441874	Cerebral thrombosis	Condition	SNOMED	NO	NO	NO

6. Sudden cardiac death

Concept Id	Concept Name	Domain	Vocabulary	Excluded	Descendants	Mapped
4048809	Brainstem death	Condition	SNOMED	NO	YES	NO
321042	Cardiac arrest	Condition	SNOMED	NO	YES	NO

© 2020 Hripcsak G. et al. *JAMA Internal Medicine.*

Concept Id	Concept Name	Domain	Vocabulary	Excluded	Descendants	Mapped
442289	Death in less than 24 hours from onset of symptoms	Observation	SNOMED	NO	YES	NO
4317150	Sudden cardiac death	Condition	SNOMED	NO	YES	NO
4132309	Sudden death	Observation	SNOMED	NO	YES	NO
437894	Ventricular fibrillation	Condition	SNOMED	YES	YES	NO

3.2.16 Chest pain or angina

Persons with chest pain or angina

Initial Event Cohort

People having any of the following:

- a condition occurrence of Chest pain or angina[1]
  - for the first time in the person's history

with continuous observation of at least 0 days prior and 0 days after event index date, and limit initial events to: **earliest event per person.**

Limit qualifying cohort to: **earliest event per person.**

End Date Strategy

No end date strategy selected. By default, the cohort end date will be the end of the observation period that contains the index event.

Cohort Collapse Strategy:

Collapse cohort by era with a gap size of 0 days.

1. Chest pain or angina

Concept Id	Concept Name	Domain	Vocabulary	Excluded	Descendants	Mapped
321318	Angina pectoris	Condition	SNOMED	NO	YES	NO

© 2020 Hripcsak G. et al. *JAMA Internal Medicine.*

Concept Id	Concept Name	Domain	Vocabulary	Excluded	Descendants	Mapped
77670	Chest pain	Condition	SNOMED	NO	NO	NO

3.2.17 Chronic kidney disease

Persons with chronic kidney disease

The first condition record of chronic kidney disease, which is followed by either another chronic kidney disease condition record or a dialysis procedure or observation

Initial Event Cohort

People having any of the following:

- a condition occurrence of Chronic kidney disease[1]
  - for the first time in the person's history

with continuous observation of at least 0 days prior and 0 days after event index date, and limit initial events to: **earliest event per person.**

For people matching the Primary Events, include:
Having any of the following criteria:

- at least 1 occurrences of a condition occurrence of Chronic kidney disease[1]

  where event starts between 1 days After and all days After index start date

- or at least 1 occurrences of a procedure of Dialysis[2]

  where event starts between 0 days Before and all days After index start date

- or at least 1 occurrences of an observation of Dialysis[2]

  where event starts between 0 days Before and all days After index start date

Limit cohort of initial events to: **earliest event per person.**
Limit qualifying cohort to: **earliest event per person.**
End Date Strategy
No end date strategy selected. By default, the cohort end date will be the end of the observation period that contains the index event.
Cohort Collapse Strategy:
Collapse cohort by era with a gap size of 0 days.

1. Chronic kidney disease

© 2020 Hripcsak G. et al. *JAMA Internal Medicine.*

Concept Id	Concept Name	Domain	Vocabulary	Excluded	Descendants	Mapped
444044	Acute tubular necrosis	Condition	SNOMED	NO	YES	NO
194385	Aneurysm of renal artery	Condition	SNOMED	NO	YES	NO
195834	Atherosclerosis of renal artery	Condition	SNOMED	NO	YES	NO
45769152	Bartter syndrome	Condition	SNOMED	YES	YES	NO
46271022	Chronic kidney disease	Condition	SNOMED	NO	YES	NO
193016	Cystic disease of kidney	Condition	SNOMED	NO	YES	NO
192279	Diabetic renal disease	Condition	SNOMED	NO	YES	NO
4263367	Glomerulonephritis	Condition	SNOMED	NO	YES	NO
261071	Glomerulosclerosis	Condition	SNOMED	NO	YES	NO
195289	Goodpasture's syndrome	Condition	SNOMED	YES	YES	NO
195737	Hemorrhagic nephroso-nephritis	Condition	SNOMED	YES	YES	NO
201313	Hypertensive renal disease	Condition	SNOMED	NO	YES	NO

© 2020 Hripcsak G. et al. *JAMA Internal Medicine.*

Concept Id	Concept Name	Domain	Vocabulary	Excluded	Descendants	Mapped
43530912	Induced termination of pregnancy complicated by renal failure	Condition	SNOMED	YES	YES	NO
4103224	Interstitial nephritis	Condition	SNOMED	NO	YES	NO
193253	Nephritis	Condition	SNOMED	NO	NO	NO
195314	Nephrotic syndrome	Condition	SNOMED	NO	YES	NO
4066005	Post-delivery acute renal failure with postnatal problem	Condition	SNOMED	YES	YES	NO
37116834	Postpartum acute renal failure	Condition	SNOMED	YES	YES	NO
195014	Renal failure following molar AND/OR ectopic pregnancy	Condition	SNOMED	YES	YES	NO
192359	Renal failure syndrome	Condition	SNOMED	NO	YES	NO
197930	Renal hypertension complicating pregnancy, childbirth and the puerperium	Condition	SNOMED	YES	YES	NO

© 2020 Hripcsak G. et al. *JAMA Internal Medicine.*

Concept Id	Concept Name	Domain	Vocabulary	Excluded	Descendants	Mapped
4128219	Urate nephropathy	Condition	SNOMED	NO	YES	NO

2. Dialysis

Concept Id	Concept Name	Domain	Vocabulary	Excluded	Descendants	Mapped
4090651	Dialysis finding	Observation	SNOMED	NO	YES	NO
4032243	Dialysis procedure	Procedure	SNOMED	NO	YES	NO
45889365	Dialysis Services and Procedures	Procedure	CPT4	NO	YES	NO

3.2.18 Cough

Cough events

Cough condition record of any type; successive records with > 90 day gap are considered independent episodes

Initial Event Cohort

People having any of the following:

- a condition occurrence of Cough[1]

with continuous observation of at least 0 days prior and 0 days after event index date, and limit initial events to: **all events per person.**

Limit qualifying cohort to: **all events per person.**

End Date Strategy

Date Offset Exit Criteria

This cohort definition end date will be the index event's start date plus 1 days

Cohort Collapse Strategy:

Collapse cohort by era with a gap size of 90 days.

1. Cough

© 2020 Hripcsak G. et al. *JAMA Internal Medicine.*

Concept Id	Concept Name	Domain	Vocabulary	Excluded	Descendants	Mapped
254761	Cough	Condition	SNOMED	NO	YES	NO

3.2.19 Decreased libido

Persons with decreased libido

The first condition record of decreased libido

Initial Event Cohort

People having any of the following:

- a condition occurrence of Decreased libido[1]
  - for the first time in the person's history

with continuous observation of at least 0 days prior and 0 days after event index date, and limit initial events to: **earliest event per person.**

Limit qualifying cohort to: **earliest event per person.**

End Date Strategy

No end date strategy selected. By default, the cohort end date will be the end of the observation period that contains the index event.

Cohort Collapse Strategy:

Collapse cohort by era with a gap size of 0 days.

1. Decreased libido

Concept Id	Concept Name	Domain	Vocabulary	Excluded	Descendants	Mapped
4087317	Lack of libido	Condition	SNOMED	NO	YES	NO
443262	Lack or loss of sexual desire	Condition	SNOMED	NO	YES	NO
436246	Reduced libido	Condition	SNOMED	NO	YES	NO

3.2.20Dementia

Persons with dementia

Initial Event Cohort

People having any of the following:

© 2020 Hripcsak G. et al. *JAMA Internal Medicine.*

- a condition occurrence of Dementia[1]
    - for the first time in the person's history

with continuous observation of at least 0 days prior and 0 days after event index date, and limit initial events to: **earliest event per person.**

Limit qualifying cohort to: **earliest event per person.**
End Date Strategy
No end date strategy selected. By default, the cohort end date will be the end of the observation period that contains the index event.
Cohort Collapse Strategy:
Collapse cohort by era with a gap size of 0 days.

1. Dementia

Concept Id	Concept Name	Domain	Vocabulary	Excluded	Descendants	Mapped
4182210	Dementia	Condition	SNOMED	NO	YES	NO
377788	General paresis - neurosyphilis	Condition	SNOMED	YES	YES	NO
372610	Postconcussion syndrome	Condition	SNOMED	YES	YES	NO

3.2.21 Depression

Persons with depression
The first condition record of depression, which is followed by another depression condition record, at least two drugs used to treat depression without another indication, or two psychotherapy procedures
Initial Event Cohort
People having any of the following:

- a condition occurrence of Depression[1]
    - for the first time in the person's history

with continuous observation of at least 0 days prior and 0 days after event index date, and limit initial events to: **earliest event per person.**

For people matching the Primary Events, include:
Having any of the following criteria:

- at least 1 occurrences of a condition occurrence of Depression[1]

© 2020 Hripcsak G. et al. *JAMA Internal Medicine.*

where event starts between 1 days After and all days After index start date

- or at least 2 occurrences of a drug exposure of Drugs to treat depression[2]

  Having all of the following criteria:

  - exactly 0 occurrences of a condition occurrence of Other indications for drugs used to treat depression[3]

    where event starts between 30 days Before and 7 days After index start date

  where event starts between 0 days Before and all days After index start date

- or at least 2 occurrences of a procedure of Procedures for depression[4]

  where event starts between 0 days Before and all days After index start date

Limit cohort of initial events to: **earliest event per person.**
Limit qualifying cohort to: **earliest event per person.**
End Date Strategy
No end date strategy selected. By default, the cohort end date will be the end of the observation period that contains the index event.
Cohort Collapse Strategy:
Collapse cohort by era with a gap size of 0 days.

1. Depression

Concept Id	Concept Name	Domain	Vocabulary	Excluded	Descendants	Mapped
442306	Adjustment disorder with depressed mood	Condition	SNOMED	NO	YES	NO
436665	Bipolar disorder	Condition	SNOMED	YES	YES	NO
440383	Depressive disorder	Condition	SNOMED	NO	YES	NO
4175329	Organic mood disorder of depressed type	Condition	SNOMED	NO	YES	NO

2. Drugs to treat depression

© 2020 Hripcsak G. et al. *JAMA Internal Medicine.*

Concept Id	Concept Name	Domain	Vocabulary	Excluded	Descendants	Mapped
21604686	ANTIDEPRESSANTS	Drug	ATC	NO	YES	NO

3. Other indications for drugs used to treat depression

Concept Id	Concept Name	Domain	Vocabulary	Excluded	Descendants	Mapped
438407	Bulimia nervosa	Condition	SNOMED	NO	YES	NO
4311708	Diabetic peripheral neuropathy	Condition	SNOMED	NO	YES	NO
434613	Generalized anxiety disorder	Condition	SNOMED	NO	YES	NO
436962	Insomnia	Condition	SNOMED	NO	YES	NO
440374	Obsessive-compulsive disorder	Condition	SNOMED	NO	YES	NO
436074	Panic disorder	Condition	SNOMED	NO	YES	NO
436676	Posttraumatic stress disorder	Condition	SNOMED	NO	YES	NO
4242733	Premenstrual dysphoric disorder	Condition	SNOMED	NO	YES	NO
440690	Social phobia	Condition	SNOMED	NO	YES	NO

4. Procedures for depression

© 2020 Hripcsak G. et al. *JAMA Internal Medicine.*

Concept Id	Concept Name	Domain	Vocabulary	Excluded	Descendants	Mapped
4030840	Electroconvulsive therapy	Procedure	SNOMED	NO	YES	NO
2795842	Mental Health, Electroconvulsive Therapy	Procedure	ICD10PCS	NO	YES	NO
2795675	Mental Health, Individual Psychotherapy	Procedure	ICD10PCS	NO	YES	NO
4327941	Psychotherapy	Procedure	SNOMED	NO	YES	NO
45887951	Psychotherapy Services and Procedures	Procedure	CPT4	NO	YES	NO

3.2.22 Diarrhea
Diarrhea events

Diarrhea condition record of any type; successive records with > 30 day gap are considered independent episodes

Initial Event Cohort

People having any of the following:

- a condition occurrence of Diarrhea[1]

with continuous observation of at least 0 days prior and 0 days after event index date, and limit initial events to: **all events per person.**

Limit qualifying cohort to: **all events per person.**

End Date Strategy

Date Offset Exit Criteria

This cohort definition end date will be the index event's start date plus 1 days

Cohort Collapse Strategy:

Collapse cohort by era with a gap size of 30 days.

1. Diarrhea

© 2020 Hripcsak G. et al. *JAMA Internal Medicine.*

Concept Id	Concept Name	Domain	Vocabulary	Excluded	Descendants	Mapped
196523	Diarrhea	Condition	SNOMED	NO	YES	NO
4134607	Diarrheal disorder	Condition	SNOMED	NO	YES	NO
201773	Enteritis of small intestine	Condition	SNOMED	NO	NO	NO
80141	Functional diarrhea	Condition	SNOMED	NO	YES	NO
4207688	Infectious enteritis	Condition	SNOMED	NO	NO	NO
4324838	Noninfectious enteritis	Condition	SNOMED	NO	NO	NO
197596	Toxic gastroenteritis	Condition	SNOMED	NO	YES	NO
196620	Viral enteritis	Condition	SNOMED	NO	YES	NO

3.2.23 End stage renal disease

Persons with end stage renal disease

End stage renal disease (ESRD) is defined by at least one diagnosis in any setting, followed by at least one additional diagnosis of a dialysis-related procedure within 90 days

Initial Event Cohort

People having any of the following:

- a condition occurrence of End stage renal disease[2]

with continuous observation of at least 0 days prior and 0 days after event index date, and limit initial events to: **all events per person.**

For people matching the Primary Events, include:
Having any of the following criteria:

- at least 1 occurrences of a condition occurrence of End stage renal disease[2]

© 2020 Hripcsak G. et al. *JAMA Internal Medicine.*

where event starts between 1 days After and 90 days After index start date

- or at least 1 occurrences of a procedure of Dialysis[1]

  where event starts between 0 days After and 90 days After index start date

- or at least 1 occurrences of an observation of Dialysis[1]

  where event starts between 0 days After and 90 days After index start date

Limit cohort of initial events to: **earliest event per person.**
Limit qualifying cohort to: **earliest event per person.**
End Date Strategy
No end date strategy selected. By default, the cohort end date will be the end of the observation period that contains the index event.
Cohort Collapse Strategy:
Collapse cohort by era with a gap size of 0 days.

1. Dialysis

Concept Id	Concept Name	Domain	Vocabulary	Excluded	Descendants	Mapped
4090651	Dialysis finding	Observation	SNOMED	NO	YES	NO
4032243	Dialysis procedure	Procedure	SNOMED	NO	YES	NO
45889365	Dialysis Services and Procedures	Procedure	CPT4	NO	YES	NO

2. End stage renal disease

Concept Id	Concept Name	Domain	Vocabulary	Excluded	Descendants	Mapped
443611	Chronic kidney disease stage 5	Condition	SNOMED	NO	YES	NO
193782	End stage renal disease	Condition	SNOMED	NO	YES	NO

© 2020 Hripcsak G. et al. *JAMA Internal Medicine.*

3.2.24 Fall

Fall events

Fall condition record of any type; successive records with > 180 day gap are considered independent episodes

Initial Event Cohort

People having any of the following:

- an observation of Falls[1]

with continuous observation of at least 0 days prior and 0 days after event index date, and limit initial events to: **all events per person.**

Limit qualifying cohort to: **all events per person.**

End Date Strategy

Date Offset Exit Criteria

This cohort definition end date will be the index event's start date plus 1 days

Cohort Collapse Strategy:

Collapse cohort by era with a gap size of 180 days.

1. Falls

Concept Id	Concept Name	Domain	Vocabulary	Excluded	Descendants	Mapped
436583	Fall	Observation	SNOMED	NO	YES	NO

3.2.25 Gastrointestinal bleeding

Gastrointestinal bleeding events

Gastrointestinal hemorrhage condition record during an inpatient or ER visit; successive records with > 30 day gap are considered independent episodes

Initial Event Cohort

People having any of the following:

- a condition occurrence of Gastrointestinal hemorrhage GI bleeding[2]

with continuous observation of at least 0 days prior and 0 days after event index date, and limit initial events to: **all events per person.**

For people matching the Primary Events, include:
Having any of the following criteria:

- at least 1 occurrences of a visit occurrence of Inpatient or ER visit[1]

  where event starts between all days Before and 0 days After index start date and event ends between 0 days Before and all days After index start date

Limit cohort of initial events to: **all events per person.**

© 2020 Hripcsak G. et al. *JAMA Internal Medicine.*

Limit qualifying cohort to: **all events per person.**
End Date Strategy
Date Offset Exit Criteria
This cohort definition end date will be the index event's start date plus 7 days
Cohort Collapse Strategy:
Collapse cohort by era with a gap size of 30 days.

1. Inpatient or ER visit

Concept Id	Concept Name	Domain	Vocabulary	Excluded	Descendants	Mapped
262	Emergency Room and Inpatient Visit	Visit	Visit	NO	YES	NO
9203	Emergency Room Visit	Visit	Visit	NO	YES	NO
9201	Inpatient Visit	Visit	Visit	NO	YES	NO

2. Gastrointestinal hemorrhage GI bleeding

Concept Id	Concept Name	Domain	Vocabulary	Excluded	Descendants	Mapped
4138962	Acute duodenal ulcer without hemorrhage AND without perforation	Condition	SNOMED	YES	YES	NO
4195231	Acute gastric ulcer without hemorrhage AND without perforation	Condition	SNOMED	YES	YES	NO
4147683	Acute gastrojejunal ulcer without hemorrhage AND without perforation	Condition	SNOMED	NO	NO	NO
4163865	Acute peptic ulcer without hemorrhage AND without perforation	Condition	SNOMED	YES	YES	NO

© 2020 Hripcsak G. et al. *JAMA Internal Medicine.*

Concept Id	Concept Name	Domain	Vocabulary	Excluded	Descendants	Mapped
195584	Acute peptic ulcer without hemorrhage AND without perforation but with obstruction	Condition	SNOMED	YES	YES	NO
40482685	Angiodysplasia of duodenum	Condition	SNOMED	NO	YES	NO
28779	Bleeding esophageal varices	Condition	SNOMED	NO	YES	NO
4222896	Chronic duodenal ulcer without hemorrhage AND without perforation	Condition	SNOMED	YES	YES	NO
4296611	Chronic gastric ulcer without hemorrhage AND without perforation	Condition	SNOMED	YES	YES	NO
200769	Chronic gastric ulcer without hemorrhage, without perforation AND without obstruction	Condition	SNOMED	YES	YES	NO
4177387	Chronic gastrojejunal ulcer without hemorrhage AND without perforation	Condition	SNOMED	YES	YES	NO
434400	Chronic gastrojejunal ulcer without hemorrhage AND without perforation but with obstruction	Condition	SNOMED	YES	YES	NO
438795	Chronic gastrojejunal ulcer without hemorrhage,	Condition	SNOMED	YES	YES	NO

© 2020 Hripcsak G. et al. *JAMA Internal Medicine.*

Concept Id	Concept Name	Domain	Vocabulary	Excluded	Descendants	Mapped
	without perforation AND without obstruction					
4204555	Chronic peptic ulcer without hemorrhage AND without perforation	Condition	SNOMED	YES	YES	NO
24973	Chronic peptic ulcer without hemorrhage AND without perforation but with obstruction	Condition	SNOMED	YES	YES	NO
23808	Chronic peptic ulcer without hemorrhage, without perforation AND without obstruction	Condition	SNOMED	YES	YES	NO
2002608	Control of hemorrhage and suture of ulcer of stomach or duodenum	Procedure	ICD9Proc	NO	YES	NO
198798	Dieulafoy's vascular malformation	Condition	SNOMED	NO	YES	NO
4198381	Duodenal ulcer disease	Condition	SNOMED	NO	YES	NO
4209746	Duodenal ulcer without hemorrhage AND without perforation	Condition	SNOMED	YES	YES	NO
4112183	Esophageal varices with bleeding, associated with another disorder	Condition	SNOMED	NO	YES	NO

© 2020 Hripcsak G. et al. *JAMA Internal Medicine.*

Concept Id	Concept Name	Domain	Vocabulary	Excluded	Descendants	Mapped
2108900	Esophagogastroduodenoscopy, flexible, transoral; with control of bleeding, any method	Procedure	CPT4	NO	YES	NO
2108878	Esophagoscopy, flexible, transoral; with control of bleeding, any method	Procedure	CPT4	NO	YES	NO
4265600	Gastric ulcer	Condition	SNOMED	NO	YES	NO
4248429	Gastric ulcer without hemorrhage AND without perforation	Condition	SNOMED	YES	YES	NO
192671	Gastrointestinal hemorrhage	Condition	SNOMED	NO	YES	NO
4101104	Gastrojejunal ulcer without hemorrhage AND without perforation	Condition	SNOMED	YES	YES	NO
443530	Hematochezia	Condition	SNOMED	YES	YES	NO
197925	Hemorrhage of rectum and anus	Condition	SNOMED	YES	YES	NO
4027663	Peptic ulcer	Condition	SNOMED	NO	YES	NO
4291028	Peptic ulcer without hemorrhage AND without perforation	Condition	SNOMED	YES	YES	NO

© 2020 Hripcsak G. et al. *JAMA Internal Medicine.*

3.2.26 Gout

Persons with gout

The first condition record of gout

Initial Event Cohort

People having any of the following:

- a condition occurrence of Gout[1]
  - for the first time in the person's history

with continuous observation of at least 0 days prior and 0 days after event index date, and limit initial events to: **earliest event per person.**

Limit qualifying cohort to: **earliest event per person.**

End Date Strategy

No end date strategy selected. By default, the cohort end date will be the end of the observation period that contains the index event.

Cohort Collapse Strategy:

Collapse cohort by era with a gap size of 0 days.

1. Gout

Concept Id	Concept Name	Domain	Vocabulary	Excluded	Descendants	Mapped
440674	Gout	Condition	SNOMED	NO	YES	NO
4096347	Gouty arthropathy	Condition	SNOMED	NO	YES	NO
4128219	Urate nephropathy	Condition	SNOMED	NO	YES	NO
80070	Uric acid urolithiasis	Condition	SNOMED	NO	YES	NO

3.2.27 Headache

Headache events

Headache condition record of any type; successive records with > 30 day gap are considered independent episodes

Initial Event Cohort

People having any of the following:

- a condition occurrence of Headache[1]

© 2020 Hripcsak G. et al. *JAMA Internal Medicine.*

with continuous observation of at least 0 days prior and 0 days after event index date, and limit initial events to: **all events per person.**

Limit qualifying cohort to: **all events per person.**
End Date Strategy
Date Offset Exit Criteria
This cohort definition end date will be the index event's start date plus 1 days
Cohort Collapse Strategy:
Collapse cohort by era with a gap size of 30 days.

1. Headache

Concept Id	Concept Name	Domain	Vocabulary	Excluded	Descendants	Mapped
378253	Headache	Condition	SNOMED	NO	YES	NO
375527	Headache disorder	Condition	SNOMED	NO	YES	NO

3.2.28 Heart failure

Persons with heart failure
The first condition record of heart failure, which is followed by at least 1 heart failure condition record in the following year
Initial Event Cohort
People having any of the following:

- a condition occurrence of Heart Failure [1]

with continuous observation of at least 0 days prior and 0 days after event index date, and limit initial events to: **all events per person.**

For people matching the Primary Events, include:
Having all of the following criteria:

- at least 1 occurrences of a condition occurrence of Heart Failure [1]

  where event starts between 1 days After and 365 days After index start date

Limit cohort of initial events to: **earliest event per person.**
Limit qualifying cohort to: **earliest event per person.**
End Date Strategy
Date Offset Exit Criteria
This cohort definition end date will be the index event's start date plus 1 days
Cohort Collapse Strategy:
Collapse cohort by era with a gap size of 0 days.

© 2020 Hripcsak G. et al. *JAMA Internal Medicine.*

1. Heart Failure

Concept Id	Concept Name	Domain	Vocabulary	Excluded	Descendants	Mapped
315295	Congestive rheumatic heart failure	Condition	SNOMED	YES	YES	NO
316139	Heart failure	Condition	SNOMED	NO	YES	NO

3.2.29 Hemorrhagic stroke

Hemorrhagic stroke (intracerebral bleeding) events

Intracranial, cerebral or subarachnoid hemorrhage condition record during an inpatient or ER visit; successive records with > 180 day gap are considered independent episodes

Initial Event Cohort

People having any of the following:

- a condition occurrence of intracranial bleed Hemorrhagic stroke[2]

with continuous observation of at least 0 days prior and 0 days after event index date, and limit initial events to: **all events per person.**

For people matching the Primary Events, include:
Having any of the following criteria:

- at least 1 occurrences of a visit occurrence of Inpatient or ER visit[1]

  where event starts between all days Before and 0 days After index start date and event ends between 0 days Before and all days After index start date

Limit cohort of initial events to: **all events per person.**
Limit qualifying cohort to: **all events per person.**

End Date Strategy

Date Offset Exit Criteria

This cohort definition end date will be the index event's start date plus 7 days

Cohort Collapse Strategy:

Collapse cohort by era with a gap size of 180 days.

1. Inpatient or ER visit

© 2020 Hripcsak G. et al. *JAMA Internal Medicine.*

Concept Id	Concept Name	Domain	Vocabulary	Excluded	Descendants	Mapped
262	Emergency Room and Inpatient Visit	Visit	Visit	NO	YES	NO
9203	Emergency Room Visit	Visit	Visit	NO	YES	NO
9201	Inpatient Visit	Visit	Visit	NO	YES	NO

2. intracranial bleed Hemorrhagic stroke

Concept Id	Concept Name	Domain	Vocabulary	Excluded	Descendants	Mapped
376713	Cerebral hemorrhage	Condition	SNOMED	NO	NO	NO
439847	Intracranial hemorrhage	Condition	SNOMED	NO	NO	NO
432923	Subarachnoid hemorrhage	Condition	SNOMED	NO	NO	NO

3.2.30 Hepatic failure

Persons with hepatic failure

The first condition record of hepatic failure, necrosis, or coma

Initial Event Cohort

People having any of the following:

- a condition occurrence of hepatic failure, necrosis or coma[1]

with continuous observation of at least 0 days prior and 0 days after event index date, and limit initial events to: **earliest event per person.**

Limit qualifying cohort to: **earliest event per person.**

End Date Strategy

No end date strategy selected. By default, the cohort end date will be the end of the observation period that contains the index event.

© 2020 Hripcsak G. et al. *JAMA Internal Medicine.*

Cohort Collapse Strategy:
Collapse cohort by era with a gap size of 0 days.

1. hepatic failure, necrosis or coma

Concept Id	Concept Name	Domain	Vocabulary	Excluded	Descendants	Mapped
377604	Hepatic coma	Condition	SNOMED	NO	NO	NO
4029488	Hepatic encephalopathy	Condition	SNOMED	NO	NO	NO
4245975	Hepatic failure	Condition	SNOMED	NO	YES	NO
4337543	Hepatic necrosis	Condition	SNOMED	NO	YES	NO

3.2.31 Hospitalization with heart failure

Hospitalization with heart failure events
Inpatient or ER visits with heart failure condition record; all qualifying inpatient visits occurring > 7 days apart are considered independent episodes
Initial Event Cohort
People having any of the following:

- a visit occurrence of Inpatient or ER visit[1]

  Having all of the following criteria:

  - at least 1 occurrences of a condition occurrence of Heart Failure [2]

    where event starts between 0 days Before and all days After index start date and event starts between all days Before and 0 days After index end date

with continuous observation of at least 0 days prior and 0 days after event index date, and limit initial events to: **all events per person.**

Limit qualifying cohort to: **all events per person.**
End Date Strategy
Date Offset Exit Criteria
This cohort definition end date will be the index event's end date plus 0 days
Cohort Collapse Strategy:
Collapse cohort by era with a gap size of 7 days.

© 2020 Hripcsak G. et al. *JAMA Internal Medicine.*

1. Inpatient or ER visit

Concept Id	Concept Name	Domain	Vocabulary	Excluded	Descendants	Mapped
262	Emergency Room and Inpatient Visit	Visit	Visit	NO	YES	NO
9203	Emergency Room Visit	Visit	Visit	NO	YES	NO
9201	Inpatient Visit	Visit	Visit	NO	YES	NO

2. Heart Failure

Concept Id	Concept Name	Domain	Vocabulary	Excluded	Descendants	Mapped
315295	Congestive rheumatic heart failure	Condition	SNOMED	YES	YES	NO
316139	Heart failure	Condition	SNOMED	NO	YES	NO

3.2.32 Hospitalization with preinfarction syndrome

Hospitalization with preinfarction syndrome events

Inpatient or ER visits with preinfarction syndrome condition record; all qualifying inpatient visits occurring > 7 days apart are considered independent episodes

Initial Event Cohort

People having any of the following:

- a visit occurrence of Inpatient or ER visit[1]

  Having all of the following criteria:

  - at least 1 occurrences of a condition occurrence of Preinfarction syndrome[2]

    where event starts between 0 days Before and all days After index start date and event starts between all days Before and 0 days After index end date

© 2020 Hripcsak G. et al. *JAMA Internal Medicine.*

with continuous observation of at least 0 days prior and 0 days after event index date, and limit initial events to: **all events per person.**

Limit qualifying cohort to: **all events per person.**
End Date Strategy
Date Offset Exit Criteria
This cohort definition end date will be the index event's end date plus 0 days
Cohort Collapse Strategy:
Collapse cohort by era with a gap size of 7 days.

1. Inpatient or ER visit

Concept Id	Concept Name	Domain	Vocabulary	Excluded	Descendants	Mapped
262	Emergency Room and Inpatient Visit	Visit	Visit	NO	YES	NO
9203	Emergency Room Visit	Visit	Visit	NO	YES	NO
9201	Inpatient Visit	Visit	Visit	NO	YES	NO

2. Preinfarction syndrome

Concept Id	Concept Name	Domain	Vocabulary	Excluded	Descendants	Mapped
315296	Preinfarction syndrome	Condition	SNOMED	NO	YES	NO

3.2.33 Hyperkalemia
Hyperkalemia events
Condition record for hyperkalemia or potassium measurements > 5.6 mmol/L; successive records with >90 day gap are considered independent episodes
Initial Event Cohort
People having any of the following:

- a condition occurrence of Hyperkalemia[1]
- a measurement of Potassium measurement[2]
    - with value as number > 5.6
    - unit is any of: millimole per liter

© 2020 Hripcsak G. et al. *JAMA Internal Medicine.*

with continuous observation of at least 0 days prior and 0 days after event index date, and limit initial events to: **all events per person.**

Limit qualifying cohort to: **all events per person.**
End Date Strategy
Date Offset Exit Criteria
This cohort definition end date will be the index event's start date plus 1 days
Cohort Collapse Strategy:
Collapse cohort by era with a gap size of 90 days.

1. Hyperkalemia

Concept Id	Concept Name	Domain	Vocabulary	Excluded	Descendants	Mapped
434610	Hyperkalemia	Condition	SNOMED	NO	YES	NO

2. Potassium measurement

Concept Id	Concept Name	Domain	Vocabulary	Excluded	Descendants	Mapped
40789893	Potassium | Bld-Ser-Plas	Measurement	LOINC	NO	YES	NO
4245152	Potassium measurement	Measurement	SNOMED	NO	YES	NO
4276440	Potassium level - finding	Condition	SNOMED	NO	YES	NO

3.2.34 Hypokalemia
Hypokalemia events
Hypokalemia condition record of any type; successive records with > 90 day gap are considered independent episodes
Initial Event Cohort
People having any of the following:

- a condition occurrence of Hypokalemia[1]

with continuous observation of at least 0 days prior and 0 days after event index date, and limit initial events to: **all events per person.**

Limit qualifying cohort to: **all events per person.**

© 2020 Hripcsak G. et al. *JAMA Internal Medicine.*

End Date Strategy
Date Offset Exit Criteria
This cohort definition end date will be the index event's start date plus 1 days
Cohort Collapse Strategy:
Collapse cohort by era with a gap size of 90 days.

1. Hypokalemia

Concept Id	Concept Name	Domain	Vocabulary	Excluded	Descendants	Mapped
45769152	Bartter syndrome	Condition	SNOMED	YES	YES	NO
437833	Hypokalemia	Condition	SNOMED	NO	YES	NO

3.2.35 Hypomagnesemia

Hypomagnesemia events
Hypomagnesemia condition record of any type; successive records with > 90 day gap are considered independent episodes
Initial Event Cohort
People having any of the following:

- a condition occurrence of Hypomagnesemia[1]

with continuous observation of at least 0 days prior and 0 days after event index date, and limit initial events to: **all events per person.**

Limit qualifying cohort to: **all events per person.**
End Date Strategy
Date Offset Exit Criteria
This cohort definition end date will be the index event's start date plus 1 days
Cohort Collapse Strategy:
Collapse cohort by era with a gap size of 90 days.

1. Hypomagnesemia

Concept Id	Concept Name	Domain	Vocabulary	Excluded	Descendants	Mapped
438725	Disorder of magnesium metabolism	Condition	SNOMED	NO	YES	NO

© 2020 Hripcsak G. et al. *JAMA Internal Medicine.*

Concept Id	Concept Name	Domain	Vocabulary	Excluded	Descendants	Mapped
4098604	Hypomagnesemia	Condition	SNOMED	NO	YES	NO

3.2.36 Hyponatremia

Hyponatremia events

Hyponatremia condition record of any type; successive records with > 90 day gap are considered independent episodes

Initial Event Cohort

People having any of the following:

- a condition occurrence of Hyponatremia[1]

with continuous observation of at least 0 days prior and 0 days after event index date, and limit initial events to: **all events per person.**

Limit qualifying cohort to: **all events per person.**

End Date Strategy

Date Offset Exit Criteria

This cohort definition end date will be the index event's start date plus 1 days

Cohort Collapse Strategy:

Collapse cohort by era with a gap size of 90 days.

1. Hyponatremia

Concept Id	Concept Name	Domain	Vocabulary	Excluded	Descendants	Mapped
435515	Hypo-osmolality and or hyponatremia	Condition	SNOMED	NO	YES	NO
4232311	Hyponatremia	Condition	SNOMED	NO	YES	NO

3.2.37 Hypotension

Hypotension events

Hypotension condition record of any type; successive records with > 90 day gap are considered independent episodes

Initial Event Cohort

People having any of the following:

© 2020 Hripcsak G. et al. *JAMA Internal Medicine.*

- a condition occurrence of Hypotension[1]

with continuous observation of at least 0 days prior and 0 days after event index date, and limit initial events to: **all events per person.**

Limit qualifying cohort to: **all events per person.**
End Date Strategy
Date Offset Exit Criteria
This cohort definition end date will be the index event's start date plus 1 days
Cohort Collapse Strategy:
Collapse cohort by era with a gap size of 90 days.

1. Hypotension

Concept Id	Concept Name	Domain	Vocabulary	Excluded	Descendants	Mapped
313232	Hemodialysis-associated hypotension	Condition	SNOMED	YES	YES	NO
317002	Low blood pressure	Condition	SNOMED	NO	YES	NO
314432	Maternal hypotension syndrome	Condition	SNOMED	YES	YES	NO

3.2.38 Impotence

Persons with impotence
The first condition record of impotence
Initial Event Cohort
People having any of the following:

- a condition occurrence of Impotence[1]
    - for the first time in the person's history

with continuous observation of at least 0 days prior and 0 days after event index date, and limit initial events to: **earliest event per person.**

Limit qualifying cohort to: **earliest event per person.**
End Date Strategy
No end date strategy selected. By default, the cohort end date will be the end of the observation period that contains the index event.
Cohort Collapse Strategy:
Collapse cohort by era with a gap size of 0 days.

© 2020 Hripcsak G. et al. *JAMA Internal Medicine.*

1. Impotence

Concept Id	Concept Name	Domain	Vocabulary	Excluded	Descendants	Mapped
4250163	Sexual arousal disorder	Condition	SNOMED	NO	YES	NO

3.2.39 Ischemic stroke

Ischemic stroke events

Ischemic stroke condition record during an inpatient or ER visit; successive records with > 180 day gap are considered independent episodes

Initial Event Cohort

People having any of the following:

- a condition occurrence of Ischemic stroke[2]

with continuous observation of at least 0 days prior and 0 days after event index date, and limit initial events to: **all events per person.**

For people matching the Primary Events, include:
Having any of the following criteria:

- at least 1 occurrences of a visit occurrence of Inpatient or ER visit[1] where event starts between all days Before and 1 days After index start date and event ends between 0 days Before and all days After index start date

Limit cohort of initial events to: **all events per person.**
Limit qualifying cohort to: **all events per person.**
End Date Strategy

Date Offset Exit Criteria

This cohort definition end date will be the index event's start date plus 7 days
Cohort Collapse Strategy:

Collapse cohort by era with a gap size of 180 days.

1. Inpatient or ER visit

Concept Id	Concept Name	Domain	Vocabulary	Excluded	Descendants	Mapped
262	Emergency Room and Inpatient Visit	Visit	Visit	NO	YES	NO

© 2020 Hripcsak G. et al. *JAMA Internal Medicine.*

Concept Id	Concept Name	Domain	Vocabulary	Excluded	Descendants	Mapped
9203	Emergency Room Visit	Visit	Visit	NO	YES	NO
9201	Inpatient Visit	Visit	Visit	NO	YES	NO

2. Ischemic stroke

Concept Id	Concept Name	Domain	Vocabulary	Excluded	Descendants	Mapped
372924	Cerebral artery occlusion	Condition	SNOMED	NO	NO	NO
375557	Cerebral embolism	Condition	SNOMED	NO	NO	NO
443454	Cerebral infarction	Condition	SNOMED	NO	YES	NO
441874	Cerebral thrombosis	Condition	SNOMED	NO	NO	NO

3.2.40Malignant neoplasm

Persons with a malignant neoplasm other than non-melanoma skin cancer

First occurrence of maligant neoplasm, followed by at least one additional diagnosis

Initial Event Cohort

People having any of the following:

- a condition occurrence of Malignant neoplasms excluding non-melanoma skin cancer[2]
    - for the first time in the person's history

with continuous observation of at least 0 days prior and 0 days after event index date, and limit initial events to: **earliest event per person.**

For people matching the Primary Events, include:
Having any of the following criteria:

- at least 2 occurrences of a condition occurrence of Malignant neoplasms of breast[5]

© 2020 Hripcsak G. et al. *JAMA Internal Medicine.*

where event starts between 0 days Before and all days After index start date

- or at least 2 occurrences of a condition occurrence of Malignant neoplasms of prostate[15]

  where event starts between 0 days Before and all days After index start date

- or at least 2 occurrences of a condition occurrence of Malignant neoplasms of lung[10]

  where event starts between 0 days Before and all days After index start date

- or at least 2 occurrences of a condition occurrence of Malignant neoplasms of multiple myeloma[12]

  where event starts between 0 days Before and all days After index start date

- or at least 2 occurrences of a condition occurrence of Malignant neoplasms of colon and rectum[6]

  where event starts between 0 days Before and all days After index start date

- or at least 2 occurrences of a condition occurrence of Malignant neoplasms of bladder[3]

  where event starts between 0 days Before and all days After index start date

- or at least 2 occurrences of a condition occurrence of Malignant neoplasms of lymphoma[11]

  where event starts between 0 days Before and all days After index start date

- or at least 2 occurrences of a condition occurrence of Malignant neoplasms of ovary[13]

  where event starts between 0 days Before and all days After index start date

- or at least 2 occurrences of a condition occurrence of Malignant neoplasms of thyroid[16]

  where event starts between 0 days Before and all days After index start date

- or at least 2 occurrences of a condition occurrence of Malignant neoplasms of kidney[7]

  where event starts between 0 days Before and all days After index start date

- or at least 2 occurrences of a condition occurrence of Malignant neoplasms of leukemia[8]

  where event starts between 0 days Before and all days After index start date

- or at least 2 occurrences of a condition occurrence of Malignant neoplasms of brain[4]

  where event starts between 0 days Before and all days After index start date

© 2020 Hripcsak G. et al. *JAMA Internal Medicine.*

- or at least 2 occurrences of a condition occurrence of Malignant neoplasms of pancreas[14]

  where event starts between 0 days Before and all days After index start date
- or at least 2 occurrences of a condition occurrence of Malignant neoplasms of liver[9]

  where event starts between 0 days Before and all days After index start date
- or at least 2 occurrences of a condition occurrence of Malignant neoplasms of uterus[17]

  where event starts between 0 days Before and all days After index start date
- or at least 2 occurrences of a condition occurrence of Malignant melanoma[1]

  where event starts between 0 days Before and all days After index start date
- or at least 2 occurrences of a condition occurrence of Myelodysplastic syndrome[18]

  where event starts between 0 days Before and all days After index start date

Limit cohort of initial events to: **earliest event per person.**
Limit qualifying cohort to: **earliest event per person.**
End Date Strategy
No end date strategy selected. By default, the cohort end date will be the end of the observation period that contains the index event.
Cohort Collapse Strategy:
Collapse cohort by era with a gap size of 0 days.

1. Malignant melanoma

Concept Id	Concept Name	Domain	Vocabulary	Excluded	Descendants	Mapped
4112752	Basal cell carcinoma of skin	Condition	SNOMED	YES	YES	NO
4162276	Malignant melanoma	Condition	SNOMED	NO	YES	NO
432851	Secondary malignant neoplastic disease	Condition	SNOMED	YES	YES	NO

© 2020 Hripcsak G. et al. *JAMA Internal Medicine.*

Concept Id	Concept Name	Domain	Vocabulary	Excluded	Descendants	Mapped
4111921	Squamous cell carcinoma of skin	Condition	SNOMED	YES	YES	NO

2. Malignant neoplasms excluding non-melanoma skin cancer

Concept Id	Concept Name	Domain	Vocabulary	Excluded	Descendants	Mapped
4112752	Basal cell carcinoma of skin	Condition	SNOMED	YES	YES	NO
443392	Malignant neoplastic disease	Condition	SNOMED	NO	YES	NO
432851	Secondary malignant neoplastic disease	Condition	SNOMED	YES	YES	NO
4111921	Squamous cell carcinoma of skin	Condition	SNOMED	YES	YES	NO

3. Malignant neoplasms of bladder

Concept Id	Concept Name	Domain	Vocabulary	Excluded	Descendants	Mapped
4112752	Basal cell carcinoma of skin	Condition	SNOMED	YES	YES	NO
197508	Malignant tumor of urinary bladder	Condition	SNOMED	NO	YES	NO
196360	Primary malignant neoplasm of bladder	Condition	SNOMED	NO	YES	NO

© 2020 Hripcsak G. et al. *JAMA Internal Medicine.*

Concept Id	Concept Name	Domain	Vocabulary	Excluded	Descendants	Mapped
432851	Secondary malignant neoplastic disease	Condition	SNOMED	YES	YES	NO
4111921	Squamous cell carcinoma of skin	Condition	SNOMED	YES	YES	NO

4. Malignant neoplasms of brain

Concept Id	Concept Name	Domain	Vocabulary	Excluded	Descendants	Mapped
4112752	Basal cell carcinoma of skin	Condition	SNOMED	YES	YES	NO
443588	Malignant neoplasm of brain	Condition	SNOMED	NO	YES	NO
432851	Secondary malignant neoplastic disease	Condition	SNOMED	YES	YES	NO
4111921	Squamous cell carcinoma of skin	Condition	SNOMED	YES	YES	NO

5. Malignant neoplasms of breast

Concept Id	Concept Name	Domain	Vocabulary	Excluded	Descendants	Mapped
4112853	Malignant tumor of breast	Condition	SNOMED	NO	YES	NO

© 2020 Hripcsak G. et al. *JAMA Internal Medicine.*

Concept Id	Concept Name	Domain	Vocabulary	Excluded	Descendants	Mapped
432851	Secondary malignant neoplastic disease	Condition	SNOMED	YES	YES	NO

6. Malignant neoplasms of colon and rectum

Concept Id	Concept Name	Domain	Vocabulary	Excluded	Descendants	Mapped
4112752	Basal cell carcinoma of skin	Condition	SNOMED	YES	YES	NO
4180790	Malignant tumor of colon	Condition	SNOMED	NO	YES	NO
443390	Malignant tumor of rectum	Condition	SNOMED	NO	YES	NO
432851	Secondary malignant neoplastic disease	Condition	SNOMED	YES	YES	NO
4111921	Squamous cell carcinoma of skin	Condition	SNOMED	YES	YES	NO

7. Malignant neoplasms of kidney

Concept Id	Concept Name	Domain	Vocabulary	Excluded	Descendants	Mapped
4112752	Basal cell carcinoma of skin	Condition	SNOMED	YES	YES	NO
196653	Malignant tumor of kidney	Condition	SNOMED	NO	YES	NO

© 2020 Hripcsak G. et al. *JAMA Internal Medicine.*

Concept Id	Concept Name	Domain	Vocabulary	Excluded	Descendants	Mapped
198985	Primary malignant neoplasm of kidney	Condition	SNOMED	NO	YES	NO
432851	Secondary malignant neoplastic disease	Condition	SNOMED	YES	YES	NO
4111921	Squamous cell carcinoma of skin	Condition	SNOMED	YES	YES	NO

8. Malignant neoplasms of leukemia

Concept Id	Concept Name	Domain	Vocabulary	Excluded	Descendants	Mapped
4112752	Basal cell carcinoma of skin	Condition	SNOMED	YES	YES	NO
317510	Leukemia	Condition	SNOMED	NO	YES	NO
432851	Secondary malignant neoplastic disease	Condition	SNOMED	YES	YES	NO
4111921	Squamous cell carcinoma of skin	Condition	SNOMED	YES	YES	NO

9. Malignant neoplasms of liver

Concept Id	Concept Name	Domain	Vocabulary	Excluded	Descendants	Mapped
4112752	Basal cell carcinoma of skin	Condition	SNOMED	YES	YES	NO

© 2020 Hripcsak G. et al. *JAMA Internal Medicine.*

Concept Id	Concept Name	Domain	Vocabulary	Excluded	Descendants	Mapped
4246127	Malignant neoplasm of liver	Condition	SNOMED	NO	YES	NO
432851	Secondary malignant neoplastic disease	Condition	SNOMED	YES	YES	NO
4111921	Squamous cell carcinoma of skin	Condition	SNOMED	YES	YES	NO

10. Malignant neoplasms of lung

Concept Id	Concept Name	Domain	Vocabulary	Excluded	Descendants	Mapped
4112752	Basal cell carcinoma of skin	Condition	SNOMED	YES	YES	NO
443388	Malignant tumor of lung	Condition	SNOMED	NO	YES	NO
4311499	Primary malignant neoplasm of respiratory tract	Condition	SNOMED	NO	NO	NO
432851	Secondary malignant neoplastic disease	Condition	SNOMED	YES	YES	NO
4111921	Squamous cell carcinoma of skin	Condition	SNOMED	YES	YES	NO

11. Malignant neoplasms of lymphoma

© 2020 Hripcsak G. et al. *JAMA Internal Medicine.*

Concept Id	Concept Name	Domain	Vocabulary	Excluded	Descendants	Mapped
4082311	B-cell chronic lymphocytic leukemia	Condition	SNOMED	YES	YES	NO
4112752	Basal cell carcinoma of skin	Condition	SNOMED	YES	YES	NO
432571	Malignant lymphoma	Condition	SNOMED	NO	YES	NO
432851	Secondary malignant neoplastic disease	Condition	SNOMED	YES	YES	NO
4111921	Squamous cell carcinoma of skin	Condition	SNOMED	YES	YES	NO

12. Malignant neoplasms of multiple myeloma

Concept Id	Concept Name	Domain	Vocabulary	Excluded	Descendants	Mapped
4112752	Basal cell carcinoma of skin	Condition	SNOMED	YES	YES	NO
437233	Multiple myeloma	Condition	SNOMED	NO	YES	NO
432851	Secondary malignant neoplastic disease	Condition	SNOMED	YES	YES	NO
4111921	Squamous cell carcinoma of skin	Condition	SNOMED	YES	YES	NO

13. Malignant neoplasms of ovary

© 2020 Hripcsak G. et al. *JAMA Internal Medicine.*

Concept Id	Concept Name	Domain	Vocabulary	Excluded	Descendants	Mapped
4112752	Basal cell carcinoma of skin	Condition	SNOMED	YES	YES	NO
4181351	Malignant tumor of ovary	Condition	SNOMED	NO	YES	NO
432851	Secondary malignant neoplastic disease	Condition	SNOMED	YES	YES	NO
4111921	Squamous cell carcinoma of skin	Condition	SNOMED	YES	YES	NO

14. Malignant neoplasms of pancreas

Concept Id	Concept Name	Domain	Vocabulary	Excluded	Descendants	Mapped
4112752	Basal cell carcinoma of skin	Condition	SNOMED	YES	YES	NO
4180793	Malignant tumor of pancreas	Condition	SNOMED	NO	YES	NO
432851	Secondary malignant neoplastic disease	Condition	SNOMED	YES	YES	NO
4111921	Squamous cell carcinoma of skin	Condition	SNOMED	YES	YES	NO

15. Malignant neoplasms of prostate

© 2020 Hripcsak G. et al. *JAMA Internal Medicine.*

Concept Id	Concept Name	Domain	Vocabulary	Excluded	Descendants	Mapped
4112752	Basal cell carcinoma of skin	Condition	SNOMED	YES	YES	NO
4163261	Malignant tumor of prostate	Condition	SNOMED	NO	YES	NO
200962	Primary malignant neoplasm of prostate	Condition	SNOMED	NO	YES	NO
432851	Secondary malignant neoplastic disease	Condition	SNOMED	YES	YES	NO
4111921	Squamous cell carcinoma of skin	Condition	SNOMED	YES	YES	NO

16. Malignant neoplasms of thyroid

Concept Id	Concept Name	Domain	Vocabulary	Excluded	Descendants	Mapped
4112752	Basal cell carcinoma of skin	Condition	SNOMED	YES	YES	NO
4181483	Malignant tumor of parathyroid gland	Condition	SNOMED	NO	YES	NO
4178976	Malignant tumor of thyroid gland	Condition	SNOMED	NO	YES	NO
432851	Secondary malignant neoplastic disease	Condition	SNOMED	YES	YES	NO

© 2020 Hripcsak G. et al. *JAMA Internal Medicine.*

Concept Id	Concept Name	Domain	Vocabulary	Excluded	Descendants	Mapped
4111921	Squamous cell carcinoma of skin	Condition	SNOMED	YES	YES	NO

17. Malignant neoplasms of uterus

Concept Id	Concept Name	Domain	Vocabulary	Excluded	Descendants	Mapped
4112752	Basal cell carcinoma of skin	Condition	SNOMED	YES	YES	NO
197230	Malignant neoplasm of uterus	Condition	SNOMED	NO	YES	NO
432851	Secondary malignant neoplastic disease	Condition	SNOMED	YES	YES	NO
4111921	Squamous cell carcinoma of skin	Condition	SNOMED	YES	YES	NO

18. Myelodysplastic syndrome

Concept Id	Concept Name	Domain	Vocabulary	Excluded	Descendants	Mapped
138994	Myelodysplastic syndrome	Condition	SNOMED	NO	YES	NO
432851	Secondary malignant neoplastic disease	Condition	SNOMED	YES	YES	NO

3.2.41 Measured renal dysfunction

Persons with measured renal dysfunction

The first creatinine measurement with value > 3 mg/dL

© 2020 Hripcsak G. et al. *JAMA Internal Medicine.*

Initial Event Cohort
People having any of the following:

- a measurement of Creatinine measurement[1]
  - with value as number > 3
  - unit is any of: milligram per deciliter

with continuous observation of at least 0 days prior and 0 days after event index date, and limit initial events to: **earliest event per person.**

Limit qualifying cohort to: **earliest event per person.**
End Date Strategy
Date Offset Exit Criteria
This cohort definition end date will be the index event's start date plus 1 days
Cohort Collapse Strategy:
Collapse cohort by era with a gap size of 0 days.

1. Creatinine measurement

Concept Id	Concept Name	Domain	Vocabulary	Excluded	Descendants	Mapped
40796376	Creatinine | Bld-Ser-Plas	Measurement	LOINC	NO	YES	NO

3.2.42 Nausea

Nausea events
Nausea condition record of any type; successive records with > 30 day gap are considered independent episodes
Initial Event Cohort
People having any of the following:

- a condition occurrence of Nausea[1]

with continuous observation of at least 0 days prior and 0 days after event index date, and limit initial events to: **all events per person.**

Limit qualifying cohort to: **all events per person.**
End Date Strategy
Date Offset Exit Criteria
This cohort definition end date will be the index event's start date plus 1 days
Cohort Collapse Strategy:
Collapse cohort by era with a gap size of 30 days.

1. Nausea

© 2020 Hripcsak G. et al. *JAMA Internal Medicine.*

Concept Id	Concept Name	Domain	Vocabulary	Excluded	Descendants	Mapped
30284	Motion sickness	Condition	SNOMED	YES	YES	NO
31967	Nausea	Condition	SNOMED	NO	YES	NO

3.2.43 Neutropenia or agranulocytosis

Persons with neutropenia or agranulocytosis

The first condition record of neutropenia or agranulocytosis

Initial Event Cohort

People having any of the following:

- a condition occurrence of Neutropenia and agranulocytosis[1]
  - for the first time in the person's history

with continuous observation of at least 0 days prior and 0 days after event index date, and limit initial events to: **earliest event per person.**

Limit qualifying cohort to: **earliest event per person.**

End Date Strategy

No end date strategy selected. By default, the cohort end date will be the end of the observation period that contains the index event.

Cohort Collapse Strategy:

Collapse cohort by era with a gap size of 0 days.

1. Neutropenia and agranulocytosis

Concept Id	Concept Name	Domain	Vocabulary	Excluded	Descendants	Mapped
4150156	Agranulocytopenic disorder	Condition	SNOMED	NO	NO	NO
440689	Agranulocytosis	Condition	SNOMED	NO	NO	NO
4322386	Chemotherapy-induced neutropenia	Condition	SNOMED	NO	NO	NO

© 2020 Hripcsak G. et al. *JAMA Internal Medicine.*

Concept Id	Concept Name	Domain	Vocabulary	Excluded	Descendants	Mapped
4174297	Chloramphenicol-induced neutropenia	Condition	SNOMED	NO	NO	NO
4211401	Dose-related drug-induced neutropenia	Condition	SNOMED	NO	NO	NO
432289	Drug-induced neutropenia	Condition	SNOMED	NO	NO	NO
320073	Neutropenia	Condition	SNOMED	NO	NO	NO
4119158	Neutropenic disorder	Condition	SNOMED	NO	NO	NO
4190716	Non dose-related drug-induced neutropenia	Condition	SNOMED	NO	NO	NO
4135712	Toxic neutropenia	Condition	SNOMED	NO	NO	NO

3.2.44 Rash

Rash events

Initial Event Cohort

People having any of the following:

- a condition occurrence of Rash[1]

with continuous observation of at least 0 days prior and 0 days after event index date, and limit initial events to: **all events per person.**

Limit qualifying cohort to: **all events per person.**

End Date Strategy

Date Offset Exit Criteria

This cohort definition end date will be the index event's start date plus 1 days

Cohort Collapse Strategy:

© 2020 Hripcsak G. et al. *JAMA Internal Medicine.*

Collapse cohort by era with a gap size of 90 days.

1. Rash

Concept Id	Concept Name	Domain	Vocabulary	Excluded	Descendants	Mapped
140214	Eruption	Condition	SNOMED	NO	YES	NO
139900	Urticaria	Condition	SNOMED	NO	YES	NO

3.2.45 Rhabdomyolysis

Rhabdomyolysis events

Rhabdomyolysis condition record or muscle disorder condition record with creatine measurement 5*ULN during an inpatient or ER visit; successive records with >90 day gap are considered independent episodes

Initial Event Cohort

People having any of the following:

- a condition occurrence of Rhabdomyolysis narrow[4]
- a condition occurrence of Rhabdomyolysis broad[2]

Having all of the following criteria:

- at least 1 occurrences of a measurement of Rhabdomyolysis measurement of creatine kinase[3]
  - with value as number > 0
  - with high range ratio > 5

  where event starts between 7 days Before and 7 days After index start date

with continuous observation of at least 0 days prior and 0 days after event index date, and limit initial events to: **all events per person.**

For people matching the Primary Events, include:
Having all of the following criteria:

- at least 1 occurrences of a visit occurrence of Inpatient or ER visit[1]

  where event starts between all days Before and 0 days After index start date and event ends between 0 days Before and all days After index start date

Limit cohort of initial events to: **all events per person.**
Limit qualifying cohort to: **all events per person.**
End Date Strategy
Date Offset Exit Criteria

© 2020 Hripcsak G. et al. *JAMA Internal Medicine.*

This cohort definition end date will be the index event's start date plus 1 days
Cohort Collapse Strategy:
Collapse cohort by era with a gap size of 90 days.

1. Inpatient or ER visit

Concept Id	Concept Name	Domain	Vocabulary	Excluded	Descendants	Mapped
262	Emergency Room and Inpatient Visit	Visit	Visit	NO	YES	NO
9203	Emergency Room Visit	Visit	Visit	NO	YES	NO
9201	Inpatient Visit	Visit	Visit	NO	YES	NO

2. Rhabdomyolysis broad

Concept Id	Concept Name	Domain	Vocabulary	Excluded	Descendants	Mapped
137967	Muscle, ligament and fascia disorders	Condition	SNOMED	NO	NO	NO
439142	Myoglobinuria	Condition	SNOMED	NO	YES	NO
4345578	Rhabdomyolysis	Condition	SNOMED	NO	YES	NO

3. Rhabdomyolysis measurement of creatine kinase

Concept Id	Concept Name	Domain	Vocabulary	Excluded	Descendants	Mapped
40782593	Creatine kinase	Measurement	LOINC	NO	YES	NO

© 2020 Hripcsak G. et al. *JAMA Internal Medicine.*

Concept Id	Concept Name	Domain	Vocabulary	Excluded	Descendants	Mapped
4265595	Creatine kinase measurement	Measurement	SNOMED	NO	YES	NO

4. Rhabdomyolysis narrow

Concept Id	Concept Name	Domain	Vocabulary	Excluded	Descendants	Mapped
439142	Myoglobinuria	Condition	SNOMED	NO	YES	NO
4345578	Rhabdomyolysis	Condition	SNOMED	NO	YES	NO

3.2.46 Stroke

Stroke (ischemic or hemorrhagic) events

Initial Event Cohort

People having any of the following:

- a condition occurrence of Stroke (ischemic or hemorrhagic)[2]

with continuous observation of at least 0 days prior and 0 days after event index date, and limit initial events to: **all events per person.**

For people matching the Primary Events, include:
Having any of the following criteria:

- at least 1 occurrences of a visit occurrence of Inpatient or ER visit[1]

  where event starts between all days Before and 1 days After index start date and event ends between 0 days Before and all days After index start date

Limit cohort of initial events to: **all events per person.**
Limit qualifying cohort to: **all events per person.**
End Date Strategy
Date Offset Exit Criteria
This cohort definition end date will be the index event's start date plus 7 days
Cohort Collapse Strategy:
Collapse cohort by era with a gap size of 180 days.

1. Inpatient or ER visit

© 2020 Hripcsak G. et al. *JAMA Internal Medicine.*

Concept Id	Concept Name	Domain	Vocabulary	Excluded	Descendants	Mapped
262	Emergency Room and Inpatient Visit	Visit	Visit	NO	YES	NO
9203	Emergency Room Visit	Visit	Visit	NO	YES	NO
9201	Inpatient Visit	Visit	Visit	NO	YES	NO

2. Stroke (ischemic or hemorrhagic)

Concept Id	Concept Name	Domain	Vocabulary	Excluded	Descendants	Mapped
372924	Cerebral artery occlusion	Condition	SNOMED	NO	NO	NO
375557	Cerebral embolism	Condition	SNOMED	NO	NO	NO
376713	Cerebral hemorrhage	Condition	SNOMED	NO	NO	NO
443454	Cerebral infarction	Condition	SNOMED	NO	YES	NO
441874	Cerebral thrombosis	Condition	SNOMED	NO	NO	NO
439847	Intracranial hemorrhage	Condition	SNOMED	NO	NO	NO
432923	Subarachnoid hemorrhage	Condition	SNOMED	NO	NO	NO

3.2.47 Sudden cardiac death

Sudden cardiac death events

© 2020 Hripcsak G. et al. *JAMA Internal Medicine.*

Sudden cardiac death condition record during an inpatient or ER visit; successive records with > 180 day gap are considered independent episodes

Initial Event Cohort

People having any of the following:

- a condition occurrence of Sudden cardiac death[2]

with continuous observation of at least 0 days prior and 0 days after event index date, and limit initial events to: **all events per person.**

For people matching the Primary Events, include:
Having all of the following criteria:

- at least 1 occurrences of a visit occurrence of Inpatient or ER visit[1]

  where event starts between all days Before and 0 days After index start date and event ends between 0 days Before and all days After index start date

Limit cohort of initial events to: **all events per person.**
Limit qualifying cohort to: **all events per person.**

End Date Strategy

Date Offset Exit Criteria

This cohort definition end date will be the index event's start date plus 7 days

Cohort Collapse Strategy:

Collapse cohort by era with a gap size of 180 days.

1. Inpatient or ER visit

Concept Id	Concept Name	Domain	Vocabulary	Excluded	Descendants	Mapped
262	Emergency Room and Inpatient Visit	Visit	Visit	NO	YES	NO
9203	Emergency Room Visit	Visit	Visit	NO	YES	NO
9201	Inpatient Visit	Visit	Visit	NO	YES	NO

2. Sudden cardiac death

© 2020 Hripcsak G. et al. *JAMA Internal Medicine.*

Concept Id	Concept Name	Domain	Vocabulary	Excluded	Descendants	Mapped
4048809	Brainstem death	Condition	SNOMED	NO	YES	NO
321042	Cardiac arrest	Condition	SNOMED	NO	YES	NO
442289	Death in less than 24 hours from onset of symptoms	Observation	SNOMED	NO	YES	NO
4317150	Sudden cardiac death	Condition	SNOMED	NO	YES	NO
4132309	Sudden death	Observation	SNOMED	NO	YES	NO
437894	Ventricular fibrillation	Condition	SNOMED	YES	YES	NO

3.2.48Syncope

Syncope events

Syncope condition record of any type; successive records with > 180 day gap are considered independent episodes

Initial Event Cohort

People having any of the following:

- a condition occurrence of Syncope[1]

with continuous observation of at least 0 days prior and 0 days after event index date, and limit initial events to: **all events per person.**

Limit qualifying cohort to: **all events per person.**

End Date Strategy

Date Offset Exit Criteria

This cohort definition end date will be the index event's start date plus 1 days

Cohort Collapse Strategy:

Collapse cohort by era with a gap size of 180 days.

1. Syncope

© 2020 Hripcsak G. et al. *JAMA Internal Medicine.*

Concept Id	Concept Name	Domain	Vocabulary	Excluded	Descendants	Mapped
140586	Heat syncope	Condition	SNOMED	YES	YES	NO
135360	Syncope	Condition	SNOMED	NO	YES	NO
38001140	Syncope & collapse	Observation	DRG	NO	NO	NO

3.2.49 Thrombocytopenia

Persons with thrombocytopenia

The first condition record of thrombocytopenia

Initial Event Cohort

People having any of the following:

- a condition occurrence of Thrombocytopenia[1]
  - for the first time in the person's history

with continuous observation of at least 0 days prior and 0 days after event index date, and limit initial events to: **earliest event per person.**

Limit qualifying cohort to: **earliest event per person.**

End Date Strategy

No end date strategy selected. By default, the cohort end date will be the end of the observation period that contains the index event.

Cohort Collapse Strategy:

Collapse cohort by era with a gap size of 0 days.

1. Thrombocytopenia

Concept Id	Concept Name	Domain	Vocabulary	Excluded	Descendants	Mapped
432870	Thrombocytopenic disorder	Condition	SNOMED	NO	YES	NO

3.2.50 Transient ischemic attack

Transient ischemic attack events

Transient ischemic attack condition record during an inpatient or ER visit; successive records with > 30 day gap are considered independent episodes

Initial Event Cohort

People having any of the following:

© 2020 Hripcsak G. et al. *JAMA Internal Medicine.*

- a condition occurrence of Transient ischemic attack (TIA) Transient cerebral Ischemia (TCA)[2]

with continuous observation of at least 0 days prior and 0 days after event index date, and limit initial events to: **all events per person.**

For people matching the Primary Events, include:
Having any of the following criteria:

- at least 1 occurrences of a visit occurrence of Inpatient or ER visit[1]

  where event starts between all days Before and 0 days After index start date and event ends between 0 days Before and all days After index start date

Limit cohort of initial events to: **all events per person.**
Limit qualifying cohort to: **all events per person.**
End Date Strategy
Date Offset Exit Criteria
This cohort definition end date will be the index event's start date plus 7 days
Cohort Collapse Strategy:
Collapse cohort by era with a gap size of 30 days.

1. Inpatient or ER visit

Concept Id	Concept Name	Domain	Vocabulary	Excluded	Descendants	Mapped
262	Emergency Room and Inpatient Visit	Visit	Visit	NO	YES	NO
9203	Emergency Room Visit	Visit	Visit	NO	YES	NO
9201	Inpatient Visit	Visit	Visit	NO	YES	NO

2. Transient ischemic attack (TIA) Transient cerebral Ischemia (TCA)

Concept Id	Concept Name	Domain	Vocabulary	Excluded	Descendants	Mapped
373503	Transient cerebral ischemia	Condition	SNOMED	NO	YES	NO

© 2020 Hripcsak G. et al. *JAMA Internal Medicine.*

3.2.51 Type 2 diabetes mellitus

Persons with type 2 diabetes mellitus

The first condition record of Type 2 Diabetes Mellitus, which is followed by another Type 2 Diabetes Mellitus condition record, at least 2 drugs used to treat Type 2 diabetes, or at least 2 HbA1c measurements with value > 6.5%

Initial Event Cohort

People having any of the following:

- a condition occurrence of Type 2 Diabetes Mellitus[3]
  - for the first time in the person's history

with continuous observation of at least 0 days prior and 0 days after event index date, and limit initial events to: **earliest event per person.**

For people matching the Primary Events, include:
Having any of the following criteria:

- at least 1 occurrences of a condition occurrence of Type 2 Diabetes Mellitus[3]

  where event starts between 1 days After and all days After index start date

- or at least 2 occurrences of a drug exposure of Drugs to treat Type 2 Diabetes Mellitus excluding insulin[1]

  where event starts between 0 days Before and all days After index start date

- or at least 2 occurrences of a measurement of Hemoglobin A1c measurement[2]
  - with value as number between 6.5 and 30 (inclusive)
  - unit is any of: percent

  where event starts between 7 days Before and all days After index start date

Limit cohort of initial events to: **earliest event per person.**
Limit qualifying cohort to: **earliest event per person.**

End Date Strategy

No end date strategy selected. By default, the cohort end date will be the end of the observation period that contains the index event.

Cohort Collapse Strategy:

Collapse cohort by era with a gap size of 0 days.

1. Drugs to treat Type 2 Diabetes Mellitus excluding insulin

Concept Id	Concept Name	Domain	Vocabulary	Excluded	Descendants	Mapped
21600744	BLOOD GLUCOSE LOWERING DRUGS, EXCL. INSULINS	Drug	ATC	NO	YES	NO

© 2020 Hripcsak G. et al. *JAMA Internal Medicine.*

2. Hemoglobin A1c measurement

Concept Id	Concept Name	Domain	Vocabulary	Excluded	Descendants	Mapped
4197971	HbA1c measurement	Measurement	SNOMED	NO	NO	NO
44786901	HEDIS 2014 Value Set - HbA1c Tests	Measurement	LOINC	NO	NO	NO
3004410	Hemoglobin A1c (Glycated)	Measurement	LOINC	NO	NO	NO
3034639	Hemoglobin A1c [Mass/volume] in Blood	Measurement	LOINC	NO	NO	NO
40758583	Hemoglobin A1c in Blood	Measurement	LOINC	NO	NO	NO
3007263	Hemoglobin A1c/Hemoglobin.total in Blood by calculation	Measurement	LOINC	NO	NO	NO
3003309	Hemoglobin A1c/Hemoglobin.total in Blood by Electrophoresis	Measurement	LOINC	NO	NO	NO
3005673	Hemoglobin A1c/Hemoglobin.total in Blood by HPLC	Measurement	LOINC	NO	NO	NO
40762352	Hemoglobin A1c/Hemoglobin.total in Blood by IFCC protocol	Measurement	LOINC	NO	NO	NO

© 2020 Hripcsak G. et al. *JAMA Internal Medicine.*

Concept Id	Concept Name	Domain	Vocabulary	Excluded	Descendants	Mapped
2212392	Hemoglobin; glycosylated (A1C)	Measurement	CPT4	NO	NO	NO

3. Type 2 Diabetes Mellitus

Concept Id	Concept Name	Domain	Vocabulary	Excluded	Descendants	Mapped
443735	Coma associated with diabetes mellitus	Condition	SNOMED	NO	YES	NO
4225656	Diabetic cataract associated with type 1 diabetes mellitus	Condition	SNOMED	YES	YES	NO
443767	Diabetic oculopathy	Condition	SNOMED	NO	YES	NO
192279	Diabetic renal disease	Condition	SNOMED	NO	YES	NO
4227210	Diabetic retinopathy associated with type 1 diabetes mellitus	Condition	SNOMED	YES	YES	NO
435216	Disorder due to type 1 diabetes mellitus	Condition	SNOMED	YES	YES	NO
443732	Disorder due to type 2 diabetes mellitus	Condition	SNOMED	NO	YES	NO

© 2020 Hripcsak G. et al. *JAMA Internal Medicine.*

Concept Id	Concept Name	Domain	Vocabulary	Excluded	Descendants	Mapped
37016355	Hyperosmolar coma due to secondary diabetes mellitus	Condition	SNOMED	YES	YES	NO
4228112	Hypoglycemic coma in type 1 diabetes mellitus	Condition	SNOMED	YES	YES	NO
4224254	Ketoacidotic coma in type 1 diabetes mellitus	Condition	SNOMED	YES	YES	NO
376065	Neurologic disorder associated with type 2 diabetes mellitus	Condition	SNOMED	NO	YES	NO
443729	Peripheral circulatory disorder associated with type 2 diabetes mellitus	Condition	SNOMED	NO	YES	NO
200687	Renal disorder associated with type 1 diabetes mellitus	Condition	SNOMED	YES	YES	NO
201254	Type 1 diabetes mellitus	Condition	SNOMED	YES	YES	NO
201531	Type 1 diabetes mellitus with hyperosmolar coma	Condition	SNOMED	YES	YES	NO

© 2020 Hripcsak G. et al. *JAMA Internal Medicine.*

Concept Id	Concept Name	Domain	Vocabulary	Excluded	Descendants	Mapped
4295011	Type 1 diabetes mellitus with persistent microalbuminuria	Condition	SNOMED	YES	YES	NO
201826	Type 2 diabetes mellitus	Condition	SNOMED	NO	YES	NO

3.2.52 Vasculitis

Persons with vasculitis

The first condition record of vasculitis, which is followed by another vasculitis condition record or drug to treat vasculitis

Initial Event Cohort

People having any of the following:

- a condition occurrence of Vasculitis[2]
    - for the first time in the person's history

with continuous observation of at least 0 days prior and 0 days after event index date, and limit initial events to: **earliest event per person.**

For people matching the Primary Events, include:
Having any of the following criteria:

- at least 1 occurrences of a condition occurrence of Vasculitis[2]

  where event starts between 1 days After and all days After index start date

- or at least 1 occurrences of a drug exposure of Drugs to treat vasculitis[1]

  where event starts between 0 days Before and 30 days After index start date

Limit cohort of initial events to: **earliest event per person.**
Limit qualifying cohort to: **earliest event per person.**

End Date Strategy

No end date strategy selected. By default, the cohort end date will be the end of the observation period that contains the index event.

Cohort Collapse Strategy:

Collapse cohort by era with a gap size of 0 days.

1. Drugs to treat vasculitis

© 2020 Hripcsak G. et al. *JAMA Internal Medicine.*

Concept Id	Concept Name	Domain	Vocabulary	Excluded	Descendants	Mapped
920458	Betamethasone	Drug	RxNorm	NO	YES	NO
1507705	Cortisone	Drug	RxNorm	NO	YES	NO
1310317	Cyclophosphamide	Drug	RxNorm	NO	YES	NO
1518254	Dexamethasone	Drug	RxNorm	NO	YES	NO
1305058	Methotrexate	Drug	RxNorm	NO	YES	NO
1506270	Methylprednisolone	Drug	RxNorm	NO	YES	NO
1550557	prednisolone	Drug	RxNorm	NO	YES	NO
1551099	Prednisone	Drug	RxNorm	NO	YES	NO
903963	Triamcinolone	Drug	RxNorm	NO	YES	NO

2. Vasculitis

Concept Id	Concept Name	Domain	Vocabulary	Excluded	Descendants	Mapped
314381	Acute febrile mucocutaneous lymph node syndrome	Condition	SNOMED	NO	YES	NO
4324395	Aortitis	Condition	SNOMED	NO	YES	NO
436642	Behcet's syndrome	Condition	SNOMED	NO	YES	NO

© 2020 Hripcsak G. et al. *JAMA Internal Medicine.*

Concept Id	Concept Name	Domain	Vocabulary	Excluded	Descendants	Mapped
4096220	Cryoglobulinemic vasculitis	Condition	SNOMED	NO	YES	NO
314963	Giant cell arteritis	Condition	SNOMED	NO	YES	NO
195289	Goodpasture's syndrome	Condition	SNOMED	NO	YES	NO
313223	Granulomatosis with polyangiitis	Condition	SNOMED	NO	YES	NO
4101602	Henoch-Schönlein purpura	Condition	SNOMED	NO	YES	NO
320749	Polyarteritis nodosa	Condition	SNOMED	NO	YES	NO
44783716	Primary angiitis of central nervous system	Condition	SNOMED	NO	YES	NO

3.2.53 Venous thromboembolic events

Venous thromboembolic (pulmonary embolism and deep vein thrombosis) events

Venous thromboembolism condition record of any type; successive records with > 180 day gap are considered independent episodes

Initial Event Cohort

People having any of the following:

- a condition occurrence of Venous thromboembolism (pulmonary embolism and deep vein thrombosis)[1]

with continuous observation of at least 0 days prior and 0 days after event index date, and limit initial events to: **all events per person.**

Limit qualifying cohort to: **all events per person.**

End Date Strategy

Date Offset Exit Criteria

© 2020 Hripcsak G. et al. *JAMA Internal Medicine.*

This cohort definition end date will be the index event's start date plus 1 days
Cohort Collapse Strategy:
Collapse cohort by era with a gap size of 180 days.

1. Venous thromboembolism (pulmonary embolism and deep vein thrombosis)

Concept Id	Concept Name	Domain	Vocabulary	Excluded	Descendants	Mapped
435616	Amniotic fluid embolism	Condition	SNOMED	YES	YES	NO
435887	Antepartum deep vein thrombosis	Condition	SNOMED	YES	YES	NO
196715	Budd-Chiari syndrome	Condition	SNOMED	YES	YES	NO
4062269	Cerebral venous thrombosis in pregnancy	Condition	SNOMED	YES	YES	NO
442055	Obstetric air pulmonary embolism	Condition	SNOMED	YES	YES	NO
433832	Obstetric blood-clot pulmonary embolism	Condition	SNOMED	YES	YES	NO
435026	Obstetric pulmonary embolism	Condition	SNOMED	YES	YES	NO
440477	Obstetric pyemic and septic pulmonary embolism	Condition	SNOMED	YES	YES	NO
318137	Phlebitis and thrombophlebitis	Condition	SNOMED	YES	YES	NO

© 2020 Hripcsak G. et al. *JAMA Internal Medicine.*

Concept Id	Concept Name	Domain	Vocabulary	Excluded	Descendants	Mapped
	of intracranial sinuses					
199837	Portal vein thrombosis	Condition	SNOMED	YES	YES	NO
438820	Postpartum deep phlebothrombosis	Condition	SNOMED	YES	YES	NO
440417	Pulmonary embolism	Condition	SNOMED	NO	YES	NO
254662	Pulmonary infarction	Condition	SNOMED	NO	YES	NO
4235812	Septic thrombophlebitis	Condition	SNOMED	YES	YES	NO
195294	Thrombosed hemorrhoids	Condition	SNOMED	YES	YES	NO
4187790	Thrombosis of retinal vein	Condition	SNOMED	YES	YES	NO
444247	Venous thrombosis	Condition	SNOMED	NO	YES	NO

3.2.54 Vertigo

Persons with vertigo

The first condition record of vertigo

Initial Event Cohort

People having any of the following:

- a condition occurrence of Vertigo[1]
  - for the first time in the person's history

© 2020 Hripcsak G. et al. *JAMA Internal Medicine.*

with continuous observation of at least 0 days prior and 0 days after event index date, and limit initial events to: **earliest event per person.**

Limit qualifying cohort to: **earliest event per person.**
End Date Strategy
No end date strategy selected. By default, the cohort end date will be the end of the observation period that contains the index event.
Cohort Collapse Strategy:
Collapse cohort by era with a gap size of 0 days.

1. Vertigo

Concept Id	Concept Name	Domain	Vocabulary	Excluded	Descendants	Mapped
437496	Epidemic vertigo	Condition	SNOMED	NO	YES	NO
78162	Peripheral vertigo	Condition	SNOMED	NO	YES	NO
439383	Vertigo	Condition	SNOMED	NO	YES	NO
381035	Vertigo of central origin	Condition	SNOMED	NO	YES	NO

3.2.55 Vomiting

Vomiting events
Vomiting condition record of any type; successive records with > 30 day gap are considered independent episodes
Initial Event Cohort
People having any of the following:

- a condition occurrence of Vomiting[1]

with continuous observation of at least 0 days prior and 0 days after event index date, and limit initial events to: **all events per person.**

Limit qualifying cohort to: **all events per person.**
End Date Strategy
Date Offset Exit Criteria
This cohort definition end date will be the index event's start date plus 1 days
Cohort Collapse Strategy:
Collapse cohort by era with a gap size of 30 days.

1. Vomiting

© 2020 Hripcsak G. et al. *JAMA Internal Medicine.*

Concept Id	Concept Name	Domain	Vocabulary	Excluded	Descendants	Mapped
40480291	Hyperemesis	Condition	SNOMED	YES	YES	NO
4216862	Postoperative vomiting	Condition	SNOMED	YES	YES	NO
441408	Vomiting	Condition	SNOMED	NO	YES	NO
440785	Vomiting of pregnancy	Condition	SNOMED	YES	YES	NO

4 eReferences

1. Schuemie M, Ryan P, Pratt N, You SC, Hripcsak G, Suchard MA. OHDSI Large-Scale Evidence Generation and Evaluation in a Network of Databases (LEGEND): Study of the Effects of Treatments for Hypertension. https://github.com/OHDSI/Legend/blob/master/Documents/OHDSI%20Legend%20Protocol%20Hypertension%20V02.docx; accessed February 8, 2019
2. OHDSI. Common Data Model. (https://github.com/OHDSI/CommonDataModel, accessed February 20, 2019).
3. U.S. National Library of Medicine. DailyMed. (https://dailymed.nlm.nih.gov/dailymed, accessed February 20, 2019).
4. PheKB. Public Phenoytpes. (https://phekb.org/phenotypes, accessed February 20, 2019).
5. Reboussin, D.M., et al., Systematic Review for the 2017 ACC/AHA/AAPA/ABC/ACPM/AGS/APhA/ASH/ASPC/NMA/PCNA Guideline for the Prevention, Detection, Evaluation, and Management of High Blood Pressure in Adults: A Report of the American College of Cardiology/American Heart Association Task Force on Clinical Practice Guidelines. Hypertension., 2018. 71(6): p. e116-e135. doi: 10.1161/HYP.0000000000000067. Epub 2017 Nov 13.
6. Whelton, P.K., et al., 2017 ACC/AHA/AAPA/ABC/ACPM/AGS/APhA/ASH/ASPC/NMA/PCNA Guideline for the Prevention, Detection, Evaluation, and Management of High Blood Pressure in Adults: A Report of the American College of Cardiology/American Heart Association Task Force on Clinical Practice Guidelines. Hypertension., 2018. 71(6): p. e13-e115. doi: 10.1161/HYP.0000000000000065. Epub 2017 Nov 13.
7. Williams, B., et al., 2018 ESC/ESH Guidelines for the management of arterial hypertension. Eur Heart J., 2018. 39(33): p. 3021-3104. doi: 10.1093/eurheartj/ehy339.
8. OHDSI. Atlas. (https://github.com/OHDSI/atlas, accessed February 20, 2019).
9. Voss EA, Boyce RD, Ryan PB, van der Lei J, Rijnbeek PR, Schuemie MJ. Accuracy of an automated knowledge base for identifying drug adverse reactions. J Biomed Inform, 2017. 66: p. 72-81.

© 2020 Hripcsak G. et al. *JAMA Internal Medicine.*

10. Winnenburg R, Sorbello A, Ripple A, Harpaz R, Tonning J, Szarfman A, Francis H, Bodenreider O. Leveraging MEDLINE indexing for pharmacovigilance - Inherent limitations and mitigation strategies. J Biomed Inform, 2015. 57: p. 425-35.
11. Duke, J., J. Friedlin, X. Li, Consistency in the safety labeling of bioequivalent medications. Pharmacoepidemiol Drug Saf, 2013. 22(3): p. 294-301.
12. Evans, S.J., P.C. Waller, and S. Davis, Use of proportional reporting ratios (PRRs) for signal generation from spontaneous adverse drug reaction reports. Pharmacoepidemiol Drug Saf, 2001. 10(6): p. 483-6.
13. Banda JM, Evans L, Vanguri RS, Tatonetti NP, Ryan PB, Shah NH. A curated and standardized adverse drug event resource to accelerate drug safety research. Sci Data, 2016. 3: p. 160026.
14. Schuemie MJ, Hripcsak G, Ryan PB, Madigan D, Suchard MA. Empirical confidence interval calibration for population-level effect estimation studies in observational healthcare data. Proc Natl Acad Sci U S A, 2018. 115(11): p. 2571-2577.
15. Schuemie MJ, Suchard MA, Ryan PB (2018). CohortMethod: New-user cohort method with large scale propensity and outcome models. R package version 2.5.1.
16. Suchard M, Simpson S, Zorych I, Ryan P, Madigan D (2013). “Massive parallelization of serial inference algorithms for a complex generalized linear model.” Transactions on Modeling and Computer Simulation, 23, 10.
17. Rosenbaum PR, Rubin DB (1983). “The central role of the propensity score in observational studies for causal effects.” Biometrika, 70(1), 41–55.
18. Schuemie MJ, Suchard MA, Ryan PB, Reps J (2018). FeatureExtraction: Generating Features for a Cohort. R package version 2.1.5.
19. Schuemie MJ, Ryan PB, DuMouchel W, Suchard MA, Madigan D. Interpreting observational studies: why empirical calibration is needed to correct p-values. Stat Med, 2014. 33(2): p. 209-18.
20. Schuemie MJ, Hripcsak G, Ryan PB, Madigan D, Suchard MA (2016). “Robust empirical calibration of p-values using observational data.” Statistics in Medicine, 35(22), 3883–3888.
21. Schuemie M, Ryan P, Hripcsak G, Madigan D, Suchard M. Improving reproducibility by using high-throughput observational studies with empirical calibration. Philos Trans A Math Phys Eng Sci, 2018. 376(2128).
22. OHDSI LEGEND Workgroup. LEGEND basic viewer. http://data.ohdsi.org/LegendBasicViewer/; accessed February 10, 2019
23. Walker AM, Patrick AR, Lauer MS, Hornbrook MC, Marin MG, Platt R, Roger VL, Stang P, Schneeweiss S. A tool for assessing the feasibility of comparative effectiveness research. Comparative Effectiveness Research 2013;3:11-20.
24. Overhage JM, Ryan PB, Reich CG, Hartzema AG, Stang PE. Validation of a common data model for active safety surveillance research. J Am Med Inform Assoc. 2012 Jan-Feb;19(1):54-60.
25. Goldstein, J.L., et al., Incidence of outpatient physician claims for upper gastrointestinal symptoms among new users of celecoxib, ibuprofen, and naproxen in an insured population in the United States. Am J Gastroenterol., 2003. 98(12): p. 2627-34. doi: 10.1111/j.1572-0241.2003.08722.x.
26. Rao, G., et al., Identifying, Analyzing, and Visualizing Diagnostic Paths for Patients with Nonspecific Abdominal Pain. Appl Clin Inform., 2018. 9(4): p. 905-913. doi: 10.1055/s-0038-1676338. Epub 2018 Dec 19.
27. Saps, M., et al., Seasonal patterns of abdominal pain consultations among adults and children. J Pediatr Gastroenterol Nutr., 2013. 56(3): p. 290-6. doi: 10.1097/MPG.0b013e3182769796.

© 2020 Hripcsak G. et al. *JAMA Internal Medicine.*

28. Broder, M.S., et al., Identification of Potential Markers for Cushing Disease. Endocr Pract., 2016. 22(5): p. 567-74. doi: 10.4158/EP15914.OR. Epub 2016 Jan 20.
29. Williams, B.A., The clinical epidemiology of fatigue in newly diagnosed heart failure. BMC Cardiovasc Disord., 2017. 17(1): p. 122. doi: 10.1186/s12872-017-0555-9.
30. Ammann, E.M., et al., Chart validation of inpatient ICD-9-CM administrative diagnosis codes for acute myocardial infarction (AMI) among intravenous immune globulin (IGIV) users in the Sentinel Distributed Database. Pharmacoepidemiol Drug Saf., 2018. 27(4): p. 398-404. doi: 10.1002/pds.4398. Epub 2018 Feb 15.
31. Floyd, J.S., et al., Validation of methods for assessing cardiovascular disease using electronic health data in a cohort of Veterans with diabetes. Pharmacoepidemiol Drug Saf., 2016. 25(4): p. 467-71. doi: 10.1002/pds.3921. Epub 2015 Nov 11.
32. Rubbo, B., et al., Use of electronic health records to ascertain, validate and phenotype acute myocardial infarction: A systematic review and recommendations. Int J Cardiol, 2015. 187:705-11.(doi): p. 10.1016/j.ijcard.2015.03.075. Epub 2015 Mar 5.
33. Singh, S., et al., Diagnostic Algorithms for Cardiovascular Death in Administrative Claims Databases: A Systematic Review. Drug Saf, 2018. 23(10): p. 018-0754.
34. Wahl, P.M., et al., Validation of claims-based diagnostic and procedure codes for cardiovascular and gastrointestinal serious adverse events in a commercially-insured population. Pharmacoepidemiol Drug Saf., 2010. 19(6): p. 596-603. doi: 10.1002/pds.1924.
35. Normand, S.L., et al., Development and validation of a claims based index for adjusting for risk of mortality: the case of acute myocardial infarction. J Clin Epidemiol., 1995. 48(2): p. 229-43.
36. Dore, D.D., et al., Stratum-specific positive predictive values of claims for acute pancreatitis among commercial health insurance plan enrollees with diabetes mellitus. Pharmacoepidemiol Drug Saf., 2011. 20(2): p. 209-13. doi: 10.1002/pds.2077. Epub 2010 Dec 23.
37. Dore, D.D., et al., A pooled analysis of exenatide use and risk of acute pancreatitis. Curr Med Res Opin., 2013. 29(12): p. 1577-86. doi: 10.1185/03007995.2013.838550. Epub 2013 Sep 13.
38. Yabe, D., et al., Use of the Japanese health insurance claims database to assess the risk of acute pancreatitis in patients with diabetes: comparison of DPP-4 inhibitors with other oral antidiabetic drugs. Diabetes Obes Metab., 2015. 17(4): p. 430-4. doi: 10.1111/dom.12381. Epub 2014 Sep 17.
39. Chen, H.J., et al., Epidemiology and outcome of acute pancreatitis in end-stage renal disease dialysis patients: a 10-year national cohort study. Nephrol Dial Transplant., 2017. 32(10): p. 1731-1736. doi: 10.1093/ndt/gfw400.
40. Afzal, Z., et al., Improving sensitivity of machine learning methods for automated case identification from free-text electronic medical records. BMC Med Inform Decis Mak., 2013. 13:30.(doi): p. 10.1186/1472-6947-13-30.
41. Lenihan, C.R., et al., Trends in acute kidney injury, associated use of dialysis, and mortality after cardiac surgery, 1999 to 2008. Ann Thorac Surg., 2013. 95(1): p. 20-8. doi: 10.1016/j.athoracsur.2012.05.131. Epub 2012 Dec 25.
42. Winkelmayer, W.C., et al., Identification of individuals with CKD from Medicare claims data: a validation study. Am J Kidney Dis., 2005. 46(2): p. 225-32. doi: 10.1053/j.ajkd.2005.04.029.
43. Grams, M.E., et al., Performance and limitations of administrative data in the identification of AKI. Clin J Am Soc Nephrol., 2014. 9(4): p. 682-9. doi: 10.2215/CJN.07650713. Epub 2014 Jan 23.

© 2020 Hripcsak G. et al. *JAMA Internal Medicine.*

44. Arnold, J., et al., Incidence and impact on outcomes of acute kidney injury after a stroke: a systematic review and meta-analysis. BMC Nephrol., 2018. 19(1): p. 283. doi: 10.1186/s12882-018-1085-0.
45. Sutherland, S.M., et al., AKI in hospitalized children: comparing the pRIFLE, AKIN, and KDIGO definitions. Clin J Am Soc Nephrol., 2015. 10(4): p. 554-61. doi: 10.2215/CJN.01900214. Epub 2015 Feb 3.
46. Waikar, S.S., et al., Validity of International Classification of Diseases, Ninth Revision, Clinical Modification Codes for Acute Renal Failure. J Am Soc Nephrol., 2006. 17(6): p. 1688-94. doi: 10.1681/ASN.2006010073. Epub 2006 Apr 26.
47. Rhee, C., et al., Improving documentation and coding for acute organ dysfunction biases estimates of changing sepsis severity and burden: a retrospective study. Crit Care., 2015. 19:338.(doi): p. 10.1186/s13054-015-1048-9.
48. Ooba, N., et al., Claims-based definition of death in Japanese claims database: validity and implications. PLoS One., 2013. 8(5): p. e66116. doi: 10.1371/journal.pone.0066116. Print 2013.
49. Robinson, T.E., et al., Development and validation of a predictive risk model for all-cause mortality in type 2 diabetes. Diabetes Res Clin Pract., 2015. 108(3): p. 482-8. doi: 10.1016/j.diabres.2015.02.015. Epub 2015 Mar 16.
50. Schneider, G., et al., A systematic review of validated methods for identifying anaphylaxis, including anaphylactic shock and angioneurotic edema, using administrative and claims data. Pharmacoepidemiol Drug Saf., 2012. 21(Suppl 1): p. 240-7. doi: 10.1002/pds.2327.
51. Walsh, K.E., et al., Validation of anaphylaxis in the Food and Drug Administration's Mini-Sentinel. Pharmacoepidemiol Drug Saf., 2013. 22(11): p. 1205-13. doi: 10.1002/pds.3505. Epub 2013 Sep 5.
52. Han, C., et al., A case-control study of anaemia in patients with rheumatoid arthritis treated with disease-modifying antirheumatic drugs in an adult population in the US: prevalence and impact on healthcare utilisation. J Med Econ, 2008. 11(2): p. 255-64. doi: 10.3111/13696990802066469.
53. Michalik, D.E., B.W. Taylor, and J.A. Panepinto, Identification and Validation of a Sickle Cell Disease Cohort Within Electronic Health Records. Acad Pediatr., 2017. 17(3): p. 283-287. doi: 10.1016/j.acap.2016.12.005. Epub 2016 Dec 13.
54. Tuck, M.G., et al., A Comprehensive Index for Predicting Risk of Anemia from Patients' Diagnoses. Big Data., 2017. 5(1): p. 42-52. doi: 10.1089/big.2016.0073.
55. Cherepanov, D., et al., Validation of an ICD-9-based claims algorithm for identifying patients with chronic idiopathic/spontaneous urticaria. Ann Allergy Asthma Immunol., 2015. 114(5): p. 393-8. doi: 10.1016/j.anai.2015.02.003. Epub 2015 Mar 12.
56. Bushnell, G.A., et al., Psychotherapy Claims Surrounding Pharmacotherapy Initiation in Children and Adolescents with Anxiety Disorders. J Child Adolesc Psychopharmacol, 2018. 19(10).
57. John, A., et al., Case-finding for common mental disorders of anxiety and depression in primary care: an external validation of routinely collected data. BMC Med Inform Decis Mak., 2016. 16:35.(doi): p. 10.1186/s12911-016-0274-7.
58. Marrie, R.A., et al., Performance of administrative case definitions for depression and anxiety in inflammatory bowel disease. J Psychosom Res., 2016. 89:107-13.(doi): p. 10.1016/j.jpsychores.2016.08.014. Epub 2016 Sep 3.
59. Castro, V.M., et al., Validation of electronic health record phenotyping of bipolar disorder cases and controls. Am J Psychiatry., 2015. 172(4): p. 363-72. doi: 10.1176/appi.ajp.2014.14030423. Epub 2014 Dec 12.
60. Shin, J., M. Gonzales, and M.J. Pletcher, Risk of emergent bradycardia associated with initiation of immediate- or slow-release metoprolol. Pharmacotherapy., 2013. 33(12): p. 1353-61. doi: 10.1002/phar.1319. Epub 2013 Jun 27.

© 2020 Hripcsak G. et al. *JAMA Internal Medicine.*

61. Turgeon, R.D., et al., Ticagrelor and bradycardia: a nested case-control study. Pharmacoepidemiol Drug Saf., 2015. 24(12): p. 1281-5. doi: 10.1002/pds.3884. Epub 2015 Oct 7.
62. Hennessy, S., et al., Validation of diagnostic codes for outpatient-originating sudden cardiac death and ventricular arrhythmia in Medicaid and Medicare claims data. Pharmacoepidemiol Drug Saf., 2010. 19(6): p. 555-62. doi: 10.1002/pds.1869.
63. Tamariz, L., T. Harkins, and V. Nair, A systematic review of validated methods for identifying ventricular arrhythmias using administrative and claims data. Pharmacoepidemiol Drug Saf., 2012. 21(Suppl 1): p. 148-53. doi: 10.1002/pds.2340.
64. Gage, B.F., et al., Validation of clinical classification schemes for predicting stroke: results from the National Registry of Atrial Fibrillation. JAMA., 2001. 285(22): p. 2864-70.
65. Jensen, P.N., et al., A systematic review of validated methods for identifying atrial fibrillation using administrative data. Pharmacoepidemiol Drug Saf., 2012. 21(Suppl 1): p. 141-7. doi: 10.1002/pds.2317.
66. Karnik, S., et al., Predicting atrial fibrillation and flutter using electronic health records. Conf Proc IEEE Eng Med Biol Soc, 2012. 2012:5562-5.(doi): p. 10.1109/EMBC.2012.6347254.
67. Navar-Boggan, A.M., et al., Accuracy and validation of an automated electronic algorithm to identify patients with atrial fibrillation at risk for stroke. Am Heart J., 2015. 169(1): p. 39-44.e2. doi: 10.1016/j.ahj.2014.09.014. Epub 2014 Oct 22.
68. Yahi, A. and N.P. Tatonetti, A knowledge-based, automated method for phenotyping in the EHR using only clinical pathology reports. AMIA Jt Summits Transl Sci Proc., 2015. 2015: p. 64-8. eCollection 2015.
69. Zaher, C., G.A. Goldberg, and P. Kadlubek, Estimating angina prevalence in a managed care population. Am J Manag Care., 2004. 10(11 Suppl): p. S339-46.
70. Chase, H.S., et al., Under-documentation of chronic kidney disease in the electronic health record in outpatients. J Am Med Inform Assoc., 2010. 17(5): p. 588-94. doi: 10.1136/jamia.2009.001396.
71. Fraccaro, P., et al., An external validation of models to predict the onset of chronic kidney disease using population-based electronic health records from Salford, UK. BMC Med., 2016. 14:104.(doi): p. 10.1186/s12916-016-0650-2.
72. Frigaard, M., et al., Validating laboratory defined chronic kidney disease in the electronic health record for patients in primary care. BMC Nephrol., 2019. 20(1): p. 3. doi: 10.1186/s12882-018-1156-2.
73. Holzmann, M.J., et al., Chronic kidney disease and 10-year risk of cardiovascular death. Eur J Prev Cardiol., 2016. 23(11): p. 1187-94. doi: 10.1177/2047487315614491. Epub 2015 Nov 5.
74. Luong, D.T.A., et al., Extracting Deep Phenotypes for Chronic Kidney Disease Using Electronic Health Records. EGEMS (Wash DC). 2017. 5(1): p. 9. doi: 10.5334/egems.226.
75. Muntner, P., et al., Validation study of medicare claims to identify older US adults with CKD using the Reasons for Geographic and Racial Differences in Stroke (REGARDS) Study. Am J Kidney Dis., 2015. 65(2): p. 249-58. doi: 10.1053/j.ajkd.2014.07.012. Epub 2014 Sep 19.
76. Nadkarni, G.N., et al., Development and validation of an electronic phenotyping algorithm for chronic kidney disease. AMIA Annu Symp Proc., 2014. 2014: p. 907-16. eCollection 2014.
77. Robertson, L.M., et al., Is routine hospital episode data sufficient for identifying individuals with chronic kidney disease? A comparison study with laboratory data. Health Informatics J., 2016. 22(2): p. 383-96. doi: 10.1177/1460458214562286. Epub 2014 Dec 31.

© 2020 Hripcsak G. et al. *JAMA Internal Medicine.*

78. Chen, C.C., et al., Estimated incidence of pertussis in people aged <50 years in the United States. Hum Vaccin Immunother., 2016. 12(10): p. 2536-2545. doi: 10.1080/21645515.2016.1186313. Epub 2016 May 31.
79. Fathima, S., et al., How well do ICD-9 physician claim diagnostic codes identify confirmed pertussis cases in Alberta, Canada? A Canadian Immunization Research Network (CIRN) Study. BMC Health Serv Res., 2017. 17(1): p. 479. doi: 10.1186/s12913-017-2321-1.
80. Lazarus, R., et al., Using automated medical records for rapid identification of illness syndromes (syndromic surveillance): the example of lower respiratory infection. BMC Public Health, 2001. 1: p. 9. Epub 2001 Oct 22.
81. Masterson, J.M., et al., Elevated Body Mass Index Is Associated with Secondary Hypogonadism Among Men Presenting to a Tertiary Academic Medical Center. World J Mens Health., 2019. 37(1): p. 93-98. doi: 10.5534/wjmh.180047. Epub 2018 Oct 10.
82. Ostbye, T., et al., Identification of dementia: agreement among national survey data, medicare claims, and death certificates. Health Serv Res., 2008. 43(1 Pt 1): p. 313-26. doi: 10.1111/j.1475-6773.2007.00748.x.
83. Sibbett, R.A., et al., Dementia ascertainment using existing data in UK longitudinal and cohort studies: a systematic review of methodology. BMC Psychiatry., 2017. 17(1): p. 239. doi: 10.1186/s12888-017-1401-4.
84. Amra, S., et al., Derivation and validation of the automated search algorithms to identify cognitive impairment and dementia in electronic health records. J Crit Care., 2017. 37:202-205.(doi): p. 10.1016/j.jcrc.2016.09.026. Epub 2016 Oct 8.
85. Jaakkimainen, R.L., et al., Identification of Physician-Diagnosed Alzheimer's Disease and Related Dementias in Population-Based Administrative Data: A Validation Study Using Family Physicians' Electronic Medical Records. J Alzheimers Dis., 2016. 54(1): p. 337-49. doi: 10.3233/JAD-160105.
86. Kosteniuk, J.G., et al., Incidence and prevalence of dementia in linked administrative health data in Saskatchewan, Canada: a retrospective cohort study. BMC Geriatr., 2015. 15:73.(doi): p. 10.1186/s12877-015-0075-3.
87. McGuinness, L.A., et al., The validity of dementia diagnoses in routinely collected electronic health records in the United Kingdom: A systematic review. Pharmacoepidemiol Drug Saf., 2019. 28(2): p. 244-255. doi: 10.1002/pds.4669. Epub 2019 Jan 22.
88. Williamson, T., et al., Validating the 8 CPCSSN case definitions for chronic disease surveillance in a primary care database of electronic health records. Ann Fam Med., 2014. 12(4): p. 367-72. doi: 10.1370/afm.1644.
89. Lin, P.J., et al., An examination of Alzheimer's disease case definitions using Medicare claims and survey data. Alzheimers Dement., 2010. 6(4): p. 334-41. doi: 10.1016/j.jalz.2009.09.001.
90. Alaghehbandan, R., et al., Using administrative databases in the surveillance of depressive disorders--case definitions. Popul Health Manag., 2012. 15(6): p. 372-80. doi: 10.1089/pop.2011.0084. Epub 2012 Jul 12.
91. Cepeda, M.S., et al., Finding treatment-resistant depression in real-world data: How a data-driven approach compares with expert-based heuristics. Depress Anxiety., 2018. 35(3): p. 220-228. doi: 10.1002/da.22705. Epub 2017 Dec 15.
92. Davidson, A.J., et al., Monitoring Depression Rates in an Urban Community: Use of Electronic Health Records. J Public Health Manag Pract., 2018. 24(6): p. E6-E14. doi: 10.1097/PHH.0000000000000751.
93. Doktorchik, C., et al., Validation of a case definition for depression in administrative data against primary chart data as a reference standard. BMC Psychiatry., 2019. 19(1): p. 9. doi: 10.1186/s12888-018-1990-6.

© 2020 Hripcsak G. et al. *JAMA Internal Medicine.*

94. Buono, J.L., et al., Economic Burden of Irritable Bowel Syndrome with Diarrhea: Retrospective Analysis of a U.S. Commercially Insured Population. J Manag Care Spec Pharm., 2017. 23(4): p. 453-460. doi: 10.18553/jmcp.2016.16138. Epub 2016 Nov 21.
95. Krishnarajah, G., et al., Public Health Impact of Complete and Incomplete Rotavirus Vaccination among Commercially and Medicaid Insured Children in the United States. PLoS One., 2016. 11(1): p. e0145977. doi: 10.1371/journal.pone.0145977. eCollection 2016.
96. Panozzo, C.A., et al., Direct, indirect, total, and overall effectiveness of the rotavirus vaccines for the prevention of gastroenteritis hospitalizations in privately insured US children, 2007-2010. Am J Epidemiol., 2014. 179(7): p. 895-909. doi: 10.1093/aje/kwu001. Epub 2014 Feb 26.
97. Shen, A.K., et al., Beneficiary characteristics and vaccinations in the end-stage renal disease Medicare beneficiary population, an analysis of claims data 2006-2015. Vaccine., 2017. 35(52): p. 7302-7308. doi: 10.1016/j.vaccine.2017.10.105. Epub 2017 Nov 10.
98. Homer, M.L., et al., Predicting Falls in People Aged 65 Years and Older from Insurance Claims. Am J Med., 2017. 130(6): p. 744.e17-744.e23. doi: 10.1016/j.amjmed.2017.01.003. Epub 2017 Jan 20.
99. Kim, D.H., et al., Measuring Frailty in Medicare Data: Development and Validation of a Claims-Based Frailty Index. J Gerontol A Biol Sci Med Sci., 2018. 73(7): p. 980-987. doi: 10.1093/gerona/glx229.
100. McCoy, T.H., Jr., et al., Validation of a risk stratification tool for fall-related injury in a state-wide cohort. BMJ Open., 2017. 7(2): p. e012189. doi: 10.1136/bmjopen-2016-012189.
101. Curtis, J.R., et al., Validation of ICD-9-CM codes to identify gastrointestinal perforation events in administrative claims data among hospitalized rheumatoid arthritis patients. Pharmacoepidemiol Drug Saf., 2011. 20(11): p. 1150-8. doi: 10.1002/pds.2215. Epub 2011 Aug 27.
102. Lanza, L.L., et al., Peptic ulcer and gastrointestinal hemorrhage associated with nonsteroidal anti-inflammatory drug use in patients younger than 65 years. A large health maintenance organization cohort study. Arch Intern Med., 1995. 155(13): p. 1371-7.
103. Lin, K.J., L.A. Garcia Rodriguez, and S. Hernandez-Diaz, Systematic review of peptic ulcer disease incidence rates: do studies without validation provide reliable estimates? Pharmacoepidemiol Drug Saf., 2011. 20(7): p. 718-28. doi: 10.1002/pds.2153. Epub 2011 May 27.
104. Patorno, E., et al., The Role of Hemoglobin Laboratory Test Results for the Detection of Upper Gastrointestinal Bleeding Outcomes Resulting from the Use of Medications in Observational Studies. Drug Saf., 2017. 40(1): p. 91-100. doi: 10.1007/s40264-016-0472-3.
105. Valkhoff, V.E., et al., Validation study in four health-care databases: upper gastrointestinal bleeding misclassification affects precision but not magnitude of drug-related upper gastrointestinal bleeding risk. J Clin Epidemiol., 2014. 67(8): p. 921-31. doi: 10.1016/j.jclinepi.2014.02.020. Epub 2014 May 1.
106. Cadzow, M., T.R. Merriman, and N. Dalbeth, Performance of gout definitions for genetic epidemiological studies: analysis of UK Biobank. Arthritis Res Ther., 2017. 19(1): p. 181. doi: 10.1186/s13075-017-1390-1.
107. Harrold, L.R., et al., Validity of gout diagnoses in administrative data. Arthritis Rheum., 2007. 57(1): p. 103-8. doi: 10.1002/art.22474.
108. MacFarlane, L.A., et al., Validation of claims-based algorithms for gout flares. Pharmacoepidemiol Drug Saf., 2016. 25(7): p. 820-6. doi: 10.1002/pds.4044. Epub 2016 May 27.
109. Malik, A., et al., Poor validation of medical record ICD-9 diagnoses of gout in a veterans affairs database. J Rheumatol., 2009. 36(6): p. 1283-6. doi: 10.3899/jrheum.081195. Epub 2009 May 15.

© 2020 Hripcsak G. et al. *JAMA Internal Medicine.*

110. Hurwitz, E.L., et al., Variations in Patterns of Utilization and Charges for the Care of Headache in North Carolina, 2000-2009: A Statewide Claims' Data Analysis. J Manipulative Physiol Ther., 2016. 39(4): p. 229-39. doi: 10.1016/j.jmpt.2016.02.008.
111. Rizzoli, P., E. Loder, and S. Joshi, Validity of cluster headache diagnoses in an electronic health record data repository. Headache., 2016. 56(7): p. 1132-6. doi: 10.1111/head.12850. Epub 2016 Jun 6.
112. Floyd, J.S., et al., Use of Electronic Health Data to Estimate Heart Failure Events in a Population-Based Cohort with CKD. Clin J Am Soc Nephrol., 2016. 11(11): p. 1954-1961. doi: 10.2215/CJN.03900416. Epub 2016 Aug 9.
113. Gini, R., et al., Automatic identification of type 2 diabetes, hypertension, ischaemic heart disease, heart failure and their levels of severity from Italian General Practitioners' electronic medical records: a validation study. BMJ Open., 2016. 6(12): p. e012413. doi: 10.1136/bmjopen-2016-012413.
114. Kaspar, M., et al., Underestimated prevalence of heart failure in hospital inpatients: a comparison of ICD codes and discharge letter information. Clin Res Cardiol., 2018. 107(9): p. 778-787. doi: 10.1007/s00392-018-1245-z. Epub 2018 Apr 17.
115. Li, Q., et al., Validity of claims-based definitions of left ventricular systolic dysfunction in Medicare patients. Pharmacoepidemiol Drug Saf., 2011. 20(7): p. 700-8. doi: 10.1002/pds.2146. Epub 2011 May 14.
116. Patel, Y.R., et al., Development and validation of a heart failure with preserved ejection fraction cohort using electronic medical records. BMC Cardiovasc Disord., 2018. 18(1): p. 128. doi: 10.1186/s12872-018-0866-5.
117. Saczynski, J.S., et al., A systematic review of validated methods for identifying heart failure using administrative data. Pharmacoepidemiol Drug Saf., 2012. 21(Suppl 1): p. 129-40. doi: 10.1002/pds.2313.
118. Schultz, S.E., et al., Identifying cases of congestive heart failure from administrative data: a validation study using primary care patient records. Chronic Dis Inj Can., 2013. 33(3): p. 160-6.
119. Feder, S.L., et al., Validation of the ICD-9 Diagnostic Code for Palliative Care in Patients Hospitalized With Heart Failure Within the Veterans Health Administration. Am J Hosp Palliat Care., 2018. 35(7): p. 959-965. doi: 10.1177/1049909117747519. Epub 2017 Dec 18.
120. Rosenman, M., et al., Database queries for hospitalizations for acute congestive heart failure: flexible methods and validation based on set theory. J Am Med Inform Assoc., 2014. 21(2): p. 345-52. doi: 10.1136/amiajnl-2013-001942. Epub 2013 Oct 10.
121. Quach, S., C. Blais, and H. Quan, Administrative data have high variation in validity for recording heart failure. Can J Cardiol., 2010. 26(8): p. 306-12.
122. Andrade, S.E., et al., A systematic review of validated methods for identifying cerebrovascular accident or transient ischemic attack using administrative data. Pharmacoepidemiol Drug Saf., 2012. 21(Suppl 1): p. 100-28. doi: 10.1002/pds.2312.
123. Park, T.H. and J.C. Choi, Validation of Stroke and Thrombolytic Therapy in Korean National Health Insurance Claim Data. J Clin Neurol., 2016. 12(1): p. 42-8. doi: 10.3988/jcn.2016.12.1.42. Epub 2015 Sep 11.
124. Gon, Y., et al., Validation of an algorithm that determines stroke diagnostic code accuracy in a Japanese hospital-based cancer registry using electronic medical records. BMC Med Inform Decis Mak., 2017. 17(1): p. 157. doi: 10.1186/s12911-017-0554-x.
125. Sung, S.F., et al., Validation of algorithms to identify stroke risk factors in patients with acute ischemic stroke, transient ischemic attack, or intracerebral hemorrhage in an administrative claims database. Int J Cardiol., 2016. 215:277-82.(doi): p. 10.1016/j.ijcard.2016.04.069. Epub 2016 Apr 14.

© 2020 Hripcsak G. et al. *JAMA Internal Medicine.*

126. Tu, K., et al., Validity of administrative data for identifying patients who have had a stroke or transient ischemic attack using EMRALD as a reference standard. Can J Cardiol., 2013. 29(11): p. 1388-94. doi: 10.1016/j.cjca.2013.07.676. Epub 2013 Sep 26.
127. Bui, C.L., et al., Validation of acute liver injury cases in a population-based cohort study of oral antimicrobial users. Curr Drug Saf., 2014. 9(1): p. 23-8.
128. Cheetham, T.C., et al., An automated causality assessment algorithm to detect drug-induced liver injury in electronic medical record data. Pharmacoepidemiol Drug Saf., 2014. 23(6): p. 601-8. doi: 10.1002/pds.3531. Epub 2013 Oct 21.
129. Jinjuvadia, K., W. Kwan, and R.J. Fontana, Searching for a needle in a haystack: use of ICD-9-CM codes in drug-induced liver injury. Am J Gastroenterol., 2007. 102(11): p. 2437-43. doi: 10.1111/j.1572-0241.2007.01456.x. Epub 2007 Jul 27.
130. Lo Re, V., 3rd, et al., Validity of diagnostic codes and laboratory tests of liver dysfunction to identify acute liver failure events. Pharmacoepidemiol Drug Saf., 2015. 24(7): p. 676-83. doi: 10.1002/pds.3774. Epub 2015 Apr 10.
131. Lo Re, V., 3rd, et al., Validity of diagnostic codes to identify cases of severe acute liver injury in the US Food and Drug Administration's Mini-Sentinel Distributed Database. Pharmacoepidemiol Drug Saf., 2013. 22(8): p. 861-72. doi: 10.1002/pds.3470. Epub 2013 Jun 25.
132. Overby, C.L., et al., A collaborative approach to developing an electronic health record phenotyping algorithm for drug-induced liver injury. J Am Med Inform Assoc., 2013. 20(e2): p. e243-52. doi: 10.1136/amiajnl-2013-001930. Epub 2013 Jul 9.
133. Udo, R., et al., Validity of diagnostic codes and laboratory measurements to identify patients with idiopathic acute liver injury in a hospital database. Pharmacoepidemiol Drug Saf., 2016. 25(Suppl 1): p. 21-8. doi: 10.1002/pds.3824. Epub 2015 Jul 5.
134. Wing, K., et al., Optimising case detection within UK electronic health records: use of multiple linked databases for detecting liver injury. BMJ Open., 2016. 6(9): p. e012102. doi: 10.1136/bmjopen-2016-012102.
135. Ryan, P.B., et al., Comparative effectiveness of canagliflozin, SGLT2 inhibitors and non-SGLT2 inhibitors on the risk of hospitalization for heart failure and amputation in patients with type 2 diabetes mellitus: A real-world meta-analysis of 4 observational databases (OBSERVE-4D). Diabetes Obes Metab., 2018. 20(11): p. 2585-2597. doi: 10.1111/dom.13424. Epub 2018 Jun 25.
136. Voors, A.A., et al., Development and validation of multivariable models to predict mortality and hospitalization in patients with heart failure. Eur J Heart Fail., 2017. 19(5): p. 627-634. doi: 10.1002/ejhf.785. Epub 2017 Mar 1.
137. Saver, B.G., et al., No pain, but no gain? The disappearance of angina hospitalizations, 1992-1999. Med Care., 2009. 47(10): p. 1106-10. doi: 10.1097/MLR.0b013e31819e1f53.
138. Varas-Lorenzo, C., et al., Positive predictive value of ICD-9 codes 410 and 411 in the identification of cases of acute coronary syndromes in the Saskatchewan Hospital automated database. Pharmacoepidemiol Drug Saf., 2008. 17(8): p. 842-52. doi: 10.1002/pds.1619.
139. Abbas, S., et al., Risk of hyperkalemia and combined use of spironolactone and long-term ACE inhibitor/angiotensin receptor blocker therapy in heart failure using real-life data: a population- and insurance-based cohort. Pharmacoepidemiol Drug Saf., 2015. 24(4): p. 406-13. doi: 10.1002/pds.3748. Epub 2015 Feb 12.
140. Betts, K.A., et al., The prevalence of hyperkalemia in the United States. Curr Med Res Opin., 2018. 34(6): p. 971-978. doi: 10.1080/03007995.2018.1433141. Epub 2018 Feb 21.
141. Fitch, K., et al., The Clinical and Economic Burden of Hyperkalemia on Medicare and Commercial Payers. Am Health Drug Benefits., 2017. 10(4): p. 202-210.

© 2020 Hripcsak G. et al. *JAMA Internal Medicine.*

142. Krogager, M.L., et al., Short-term mortality risk of serum potassium levels in hypertension: a retrospective analysis of nationwide registry data. Eur Heart J., 2017. 38(2): p. 104-112. doi: 10.1093/eurheartj/ehw129.
143. Koulouridis, I., et al., Out-of-hospital use of proton pump inhibitors and hypomagnesemia at hospital admission: a nested case-control study. Am J Kidney Dis., 2013. 62(4): p. 730-7. doi: 10.1053/j.ajkd.2013.02.373. Epub 2013 May 10.
144. Markovits, N., et al., The association of proton pump inhibitors and hypomagnesemia in the community setting. J Clin Pharmacol., 2014. 54(8): p. 889-95. doi: 10.1002/jcph.316. Epub 2014 May 6.
145. Movig, K.L., et al., Validity of hospital discharge International Classification of Diseases (ICD) codes for identifying patients with hyponatremia. J Clin Epidemiol., 2003. 56(6): p. 530-5.
146. Shea, A.M., et al., Sensitivity of International Classification of Diseases codes for hyponatremia among commercially insured outpatients in the United States. BMC Nephrol., 2008. 9:5.(doi): p. 10.1186/1471-2369-9-5.
147. Chrischilles, E., et al., Initiation of nonselective alpha1-antagonist therapy and occurrence of hypotension-related adverse events among men with benign prostatic hyperplasia: a retrospective cohort study. Clin Ther., 2001. 23(5): p. 727-43.
148. Bekelman, J.E., et al., Outcomes after intensity-modulated versus conformal radiotherapy in older men with nonmetastatic prostate cancer. Int J Radiat Oncol Biol Phys., 2011. 81(4): p. e325-34. doi: 10.1016/j.ijrobp.2011.02.006. Epub 2011 Apr 16.
149. Frederick, L.R., et al., Undertreatment of erectile dysfunction: claims analysis of 6.2 million patients. J Sex Med., 2014. 11(10): p. 2546-53. doi: 10.1111/jsm.12647. Epub 2014 Jul 24.
150. McVary, K., et al., Identifying patients with benign prostatic hyperplasia through a diagnosis of, or treatment for, erectile dysfunction. Curr Med Res Opin., 2008. 24(3): p. 775-84. doi: 10.1185/030079908X260916. Epub 2008 Jan 30.
151. Mulhall, J.P., et al., Relationship between age and erectile dysfunction diagnosis or treatment using real-world observational data in the USA. Int J Clin Pract., 2016. 70(12): p. 1012-1018. doi: 10.1111/ijcp.12908.
152. Yuan, Z., et al., Risk Prediction for Ischemic Stroke and Transient Ischemic Attack in Patients Without Atrial Fibrillation: A Retrospective Cohort Study. J Stroke Cerebrovasc Dis., 2017. 26(8): p. 1721-1731. doi: 10.1016/j.jstrokecerebrovasdis.2017.03.036. Epub 2017 Apr 6.
153. Czwikla, J., K. Jobski, and T. Schink, The impact of the lookback period and definition of confirmatory events on the identification of incident cancer cases in administrative data. BMC Med Res Methodol., 2017. 17(1): p. 122. doi: 10.1186/s12874-017-0407-4.
154. Abraha, I., et al., Accuracy of administrative databases in detecting primary breast cancer diagnoses: a systematic review. BMJ Open., 2018. 8(7): p. e019264. doi: 10.1136/bmjopen-2017-019264.
155. Abraha, I., et al., Sensitivity and specificity of breast cancer ICD-9-CM codes in three Italian administrative healthcare databases: a diagnostic accuracy study. BMJ Open., 2018. 8(7): p. e020627. doi: 10.1136/bmjopen-2017-020627.
156. Baldi, I., et al., A high positive predictive value algorithm using hospital administrative data identified incident cancer cases. J Clin Epidemiol., 2008. 61(4): p. 373-9. doi: 10.1016/j.jclinepi.2007.05.017. Epub 2007 Oct 22.
157. Cea Soriano, L., M. Soriano-Gabarro, and L.A. Garcia Rodriguez, Validity and completeness of colorectal cancer diagnoses in a primary care database in the United Kingdom. Pharmacoepidemiol Drug Saf., 2016. 25(4): p. 385-91. doi: 10.1002/pds.3877. Epub 2015 Oct 5.

© 2020 Hripcsak G. et al. *JAMA Internal Medicine.*

158. Chawla, N., et al., Limited validity of diagnosis codes in Medicare claims for identifying cancer metastases and inferring stage. Ann Epidemiol, 2014. 2014 Sep;24(9): p. 666-72.
159. Creighton, N., et al., Validation of administrative hospital data for identifying incident pancreatic and periampullary cancer cases: a population-based study using linked cancer registry and administrative hospital data in New South Wales, Australia. BMJ Open., 2016. 6(7): p. e011161. doi: 10.1136/bmjopen-2016-011161.
160. Dregan, A., et al., Validity of cancer diagnosis in a primary care database compared with linked cancer registrations in England. Population-based cohort study. Cancer Epidemiol., 2012. 36(5): p. 425-9. doi: 10.1016/j.canep.2012.05.013. Epub 2012 Jun 21.
161. Goldsbury, D., et al., Identifying incident colorectal and lung cancer cases in health service utilisation databases in Australia: a validation study. BMC Med Inform Decis Mak., 2017. 17(1): p. 23. doi: 10.1186/s12911-017-0417-5.
162. Gupta, S., et al., Validity of Administrative Data in Identifying Cancer-related Events in Adolescents and Young Adults: A Population-based Study Using the IMPACT Cohort. Med Care., 2018. 56(6): p. e32-e38. doi: 10.1097/MLR.0000000000000777.
163. Hassett, M.J., et al., Validating billing/encounter codes as indicators of lung, colorectal, breast, and prostate cancer recurrence using 2 large contemporary cohorts. Med Care., 2014. 52(10): p. e65-73. doi: 10.1097/MLR.0b013e318277eb6f.
164. Kim, S.C., et al., Validation of claims-based algorithms for identification of high-grade cervical dysplasia and cervical cancer. Pharmacoepidemiol Drug Saf., 2013. 22(11): p. 1239-44. doi: 10.1002/pds.3520. Epub 2013 Sep 12.
165. Nordstrom, B.L., et al., Identification of metastatic cancer in claims data. Pharmacoepidemiol Drug Saf., 2012. 21(Suppl 2): p. 21-8. doi: 10.1002/pds.3247.
166. Penberthy, L., et al., Using hospital discharge files to enhance cancer surveillance. Am J Epidemiol., 2003. 158(1): p. 27-34.
167. Stavrou, E., N. Pesa, and S.A. Pearson, Hospital discharge diagnostic and procedure codes for upper gastro-intestinal cancer: how accurate are they? BMC Health Serv Res., 2012. 12:331.(doi): p. 10.1186/1472-6963-12-331.
168. Goldberg, D.S., et al., Validation of a coding algorithm to identify patients with hepatocellular carcinoma in an administrative database. Pharmacoepidemiol Drug Saf., 2013. 22(1): p. 103-7. doi: 10.1002/pds.3367. Epub 2012 Nov 4.
169. Donga, P.Z., et al., Comparative treatment-related adverse event cost burden in immune thrombocytopenic purpura. J Med Econ., 2017. 20(11): p. 1200-1206. doi: 10.1080/13696998.2017.1370425. Epub 2017 Sep 8.
170. Marrett, E., et al., Health Care Utilization and Costs Associated with Nausea and Vomiting in Patients Receiving Oral Immediate-Release Opioids for Outpatient Acute Pain Management. Pain Ther., 2016. 5(2): p. 215-226. doi: 10.1007/s40122-016-0057-y. Epub 2016 Oct 4.
171. Kim, S.Y., et al., Accuracy of identifying neutropenia diagnoses in outpatient claims data. Pharmacoepidemiol Drug Saf., 2011. 20(7): p. 709-13. doi: 10.1002/pds.2157. Epub 2011 May 12.
172. Weycker, D., et al., Technical evaluation of methods for identifying chemotherapy-induced febrile neutropenia in healthcare claims databases. BMC Health Serv Res., 2013. 13:60.(doi): p. 10.1186/1472-6963-13-60.
173. Schneider, G., et al., A systematic review of validated methods for identifying hypersensitivity reactions other than anaphylaxis (fever, rash, and lymphadenopathy), using administrative and claims data. Pharmacoepidemiol Drug Saf., 2012. 21(Suppl 1): p. 248-55. doi: 10.1002/pds.2333.
174. Andrade, S.E., et al., Health plan administrative databases can efficiently identify serious myopathy and rhabdomyolysis. J Clin Epidemiol., 2005. 58(2): p. 171-4. doi: 10.1016/j.jclinepi.2004.10.004.

© 2020 Hripcsak G. et al. *JAMA Internal Medicine.*

175. Chan, S.L., et al., Development and validation of algorithms for the detection of statin myopathy signals from electronic medical records. Clin Pharmacol Ther., 2017. 101(5): p. 667-674. doi: 10.1002/cpt.526. Epub 2017 Jan 21.
176. Wahl, P.M., et al., Validation of claims-based diagnostic codes for idiopathic thrombotic thrombocytopenic purpura in a commercially-insured population. Thromb Haemost., 2010. 103(6): p. 1203-9. doi: 10.1160/TH09-08-0595. Epub 2010 Mar 29.
177. Moulis, G., et al., Validation of immune thrombocytopenia diagnosis code in the French hospital electronic database. Eur J Intern Med., 2016. 32:e21-2.(doi): p. 10.1016/j.ejim.2016.02.021. Epub 2016 Mar 21.
178. Khokhar, B., et al., Systematic review of validated case definitions for diabetes in ICD-9-coded and ICD-10-coded data in adult populations. BMJ Open., 2016. 6(8): p. e009952. doi: 10.1136/bmjopen-2015-009952.
179. Leong, A., et al., Systematic review and meta-analysis of validation studies on a diabetes case definition from health administrative records. PLoS One., 2013. 8(10): p. e75256. doi: 10.1371/journal.pone.0075256. eCollection 2013.
180. Chen, G., et al., Validating ICD coding algorithms for diabetes mellitus from administrative data. Diabetes Res Clin Pract., 2010. 89(2): p. 189-95. doi: 10.1016/j.diabres.2010.03.007. Epub 2010 Apr 2.
181. Thorpe, C.T., et al., Healthcare utilization and expenditures for United States Medicare beneficiaries with systemic vasculitis. Semin Arthritis Rheum., 2018. 47(4): p. 507-519. doi: 10.1016/j.semarthrit.2017.08.005. Epub 2017 Aug 10.
182. England, B.R., et al., Herpes Zoster as a Risk Factor for Incident Giant Cell Arteritis. Arthritis Rheumatol., 2017. 69(12): p. 2351-2358. doi: 10.1002/art.40236. Epub 2017 Nov 9.
183. Tamariz, L., T. Harkins, and V. Nair, A systematic review of validated methods for identifying venous thromboembolism using administrative and claims data. Pharmacoepidemiol Drug Saf., 2012. 21(Suppl 1): p. 154-62. doi: 10.1002/pds.2341.
184. Burwen, D.R., et al., Venous thromboembolism incidence, recurrence, and mortality based on Women's Health Initiative data and Medicare claims. Thromb Res., 2017. 150:78-85.(doi): p. 10.1016/j.thromres.2016.11.015. Epub 2016 Nov 15.
185. Coleman, C.I., et al., External validation of a multivariable claims-based rule for predicting in-hospital mortality and 30-day post-pulmonary embolism complications. BMC Health Serv Res., 2016. 16(1): p. 610. doi: 10.1186/s12913-016-1855-y.
186. Ammann, E.M., et al., Chart validation of inpatient International Classification of Diseases, Ninth Revision, Clinical Modification (ICD-9-CM) administrative diagnosis codes for venous thromboembolism (VTE) among intravenous immune globulin (IGIV) users in the Sentinel Distributed Database. Medicine (Baltimore). 2018. 97(8): p. e9960. doi: 10.1097/MD.0000000000009960.
187. Koo, M., J.C. Chen, and J.H. Hwang, Risk of Peripheral Artery Occlusive Disease in Patients with Vertigo, Tinnitus, or Sudden Deafness: A Secondary Case-Control Analysis of a Nationwide, Population-Based Health Claims Database. PLoS One., 2016. 11(9): p. e0162629. doi: 10.1371/journal.pone.0162629. eCollection 2016.

© 2020 Hripcsak G. et al. *JAMA Internal Medicine.*
